# Catalytic Hydrodefluorination via Oxidative Addition, Ligand Metathesis, and Reductive Elimination at Bi(I)/Bi(III) Centers

**DOI:** 10.1021/jacs.1c06735

**Published:** 2021-08-06

**Authors:** Yue Pang, Markus Leutzsch, Nils Nöthling, Felix Katzenburg, Josep Cornella

**Affiliations:** Max-Planck-Institut für Kohlenforschung, Kaiser-Wilhelm-Platz 1, Mülheim an der Ruhr 45470, Germany

## Abstract

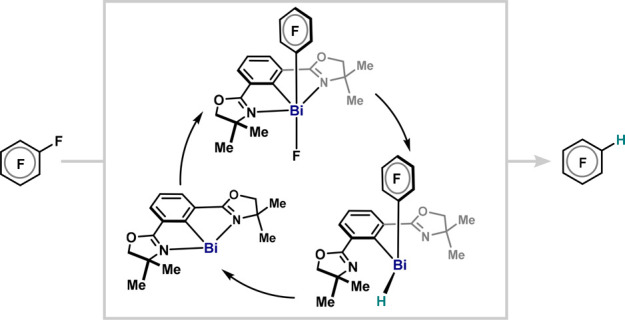

Herein, we report a hydrodefluorination reaction of polyfluoroarenes catalyzed by bismuthinidenes, Phebox-Bi(I) and OMe-Phebox-Bi(I). Mechanistic studies on the elementary steps support a Bi(I)/Bi(III) redox cycle that comprises C(sp^2^)–F oxidative addition, F/H ligand metathesis, and C(sp^2^)–H reductive elimination. Isolation and characterization of a cationic Phebox-Bi(III)(4-tetrafluoropyridyl) triflate manifests the feasible oxidative addition of Phebox-Bi(I) into the C(sp^2^)–F bond. Spectroscopic evidence was provided for the formation of a transient Phebox-Bi(III)(4-tetrafluoropyridyl) hydride during catalysis, which decomposes at low temperature to afford the corresponding C(sp^2^)–H bond while regenerating the propagating Phebox-Bi(I). This protocol represents a distinct catalytic example where a main-group center performs three elementary organometallic steps in a low-valent redox manifold.

The elementary organometallic steps, oxidative addition (OA), ligand metathesis (LM), and reductive elimination (RE), define the innate capacity of transition-metal centers to revolve between different oxidation states in numerous catalytic processes (Figure 1A).^1^ With the aim of mimicking such reactivity by elements beyond the d-block, the past decades have witnessed prominent progress in low-valent main-group compounds exhibiting transition-metal-like reactivity, in particular, the cleavage of strong chemical bonds (e.g., N–H, O–H, H–H, C–H, C–F) through OA.^2^ However, the intrinsic difficulties posed by the regeneration of low-valent species via RE limited the development of efficient catalytic redox processes based on main-group catalysts.^2b,2c^ Located in the middle of the p-block, group 15 elements have recently been identified as privileged candidates to unfold redox catalysis,^3^ as exemplified by the success of redox cycling using P and Bi redox couples in various catalytic reactions.^4−6^ In this endeavor, our group reported catalytic C(sp^2^)–F and C(sp^2^)–OTf/ONf bond formation proceeding through canonical cross-coupling steps in a Bi(III)/Bi(V) manifold (Figure 1B).^5^ However, in contrast to other pnictogens, Bi possesses additional low-valent redox manifolds to be exploited. Indeed, the Bi(I)/(III) redox couple has recently emerged and found applications in catalytic transfer hydrogenation of azo- and nitro-arenes, as well as in the catalytic activation of N_2_O.^6^ The low-valent Bi(I)/(III) redox manifold distinguishes itself from the high-valent and radical processes^7^ by its superior catalytic efficiency, and achieving catalytic redox transformations via the full triad of three elementary organometallic steps would be highly desirable.

**Figure 1:**
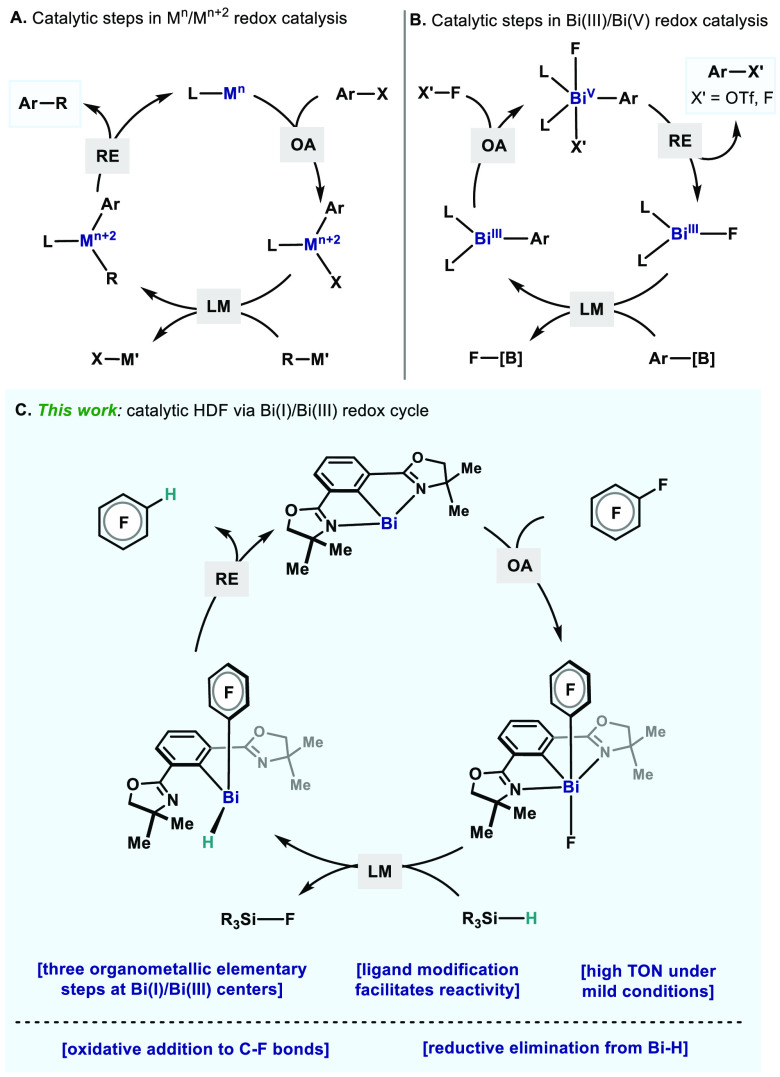
(A) Well-established transition-metal catalytic cycle. (B) Bi(III)/Bi(V) redox catalysis including elementary organometallic steps of OA/LM/RE. (C) HDF via Bi(I)/Bi(III) catalysis: elementary organometallic steps at low-valent main-group centers.

Hydrodefluorination (HDF) of polyfluoroarenes is a fundamental reaction that enables access to partially fluorinated building blocks from perfluorinated bulk chemicals.^8^ HDFs have largely been dominated by transition-metal catalysis,^9,10^ and a considerable number of these systems proceed through the catalytic steps depicted in Figure 1A.^10^ Recent progress in HDFs extended the available strategies to photoredox catalysis^11^ and main-group catalysis,^12,13^ which proceed through mechanistically distinct catalytic steps. In addition to its synthetic potential, HDF serves as a model reaction for studying the performance of main-group compounds in the elementary organometallic steps of a catalytic cycle. In this regard, C–F OA has been established for low-valent group 13/14 elements,^14^ and recently Radosevich has further shown an elegant synthetic cycle for HDF at a phosphorus triamide.^15^ Herein, we report that bismuthinidenes with a rationally designed *N,C,N*-pincer ligand scaffold unlock the *catalytic* HDF of a variety of polyfluoroarenes (Figure 1C). Mechanistic studies suggest a Bi(I)/Bi(III) cycle comprising C–F OA, F/H LM, and C–H RE steps, in a manner akin to a canonical catalytic cycle of transition-metal congeners.

Initially, we attempted the HDF of hexafluorobenzene (**1a**) using 5 mol% of Dostál’s bismuthinidene **3**(16) as catalyst and 2.4 equiv. of Et_2_SiH_2_ as hydrogen source in THF at 60 °C (Figure 2A). Unfortunately, only a trace amount of HDF product (**2a**, <1%) was detected after 20 h. With the aim of tuning the electronics of the Bi(I) center, an alternative *N*,*C*,*N*-pincer scaffold was envisaged, where the imine arms are replaced with oxazoline groups. In this manner, two new bismuthinidenes supported by a 2,6-bis(oxazolinyl)phenyl (Phebox) ligand scaffold,^17^ Phebox-Bi(I) (**4**) and OMe-Phebox-Bi(I) (**5**), were synthesized via cobaltocene reduction of the parent bismuth chlorides **6** and **7**.^6b,18^ When **4** and **5** were tested as catalysts for the HDF of **1a**, 40% and 74% of **2a** were obtained, respectively. In the case of **5**, two-fold HDF (**2a′**) could also be detected in 16% yield. To gain more insights on the boosted reactivity, X-ray crystal structures of **4** and **5** were compared with that of **3**, showing considerably more elongated Bi1–C1 distances [2.193(6) Å for **4**,^19^ 2.201(2) Å for **5**, cf. 2.146(18) Å for **3**,^19^Figure 2B]. These data suggest that electron delocalization of the 6p_*z*^2^_ lone pair of Bi to the *ipso* C(sp^2^) is diminished in the new bismuthinidenes, leading to the enhanced reactivity of the Phebox-based Bi(I) in HDFs.^16,20^

**Figure 2:**
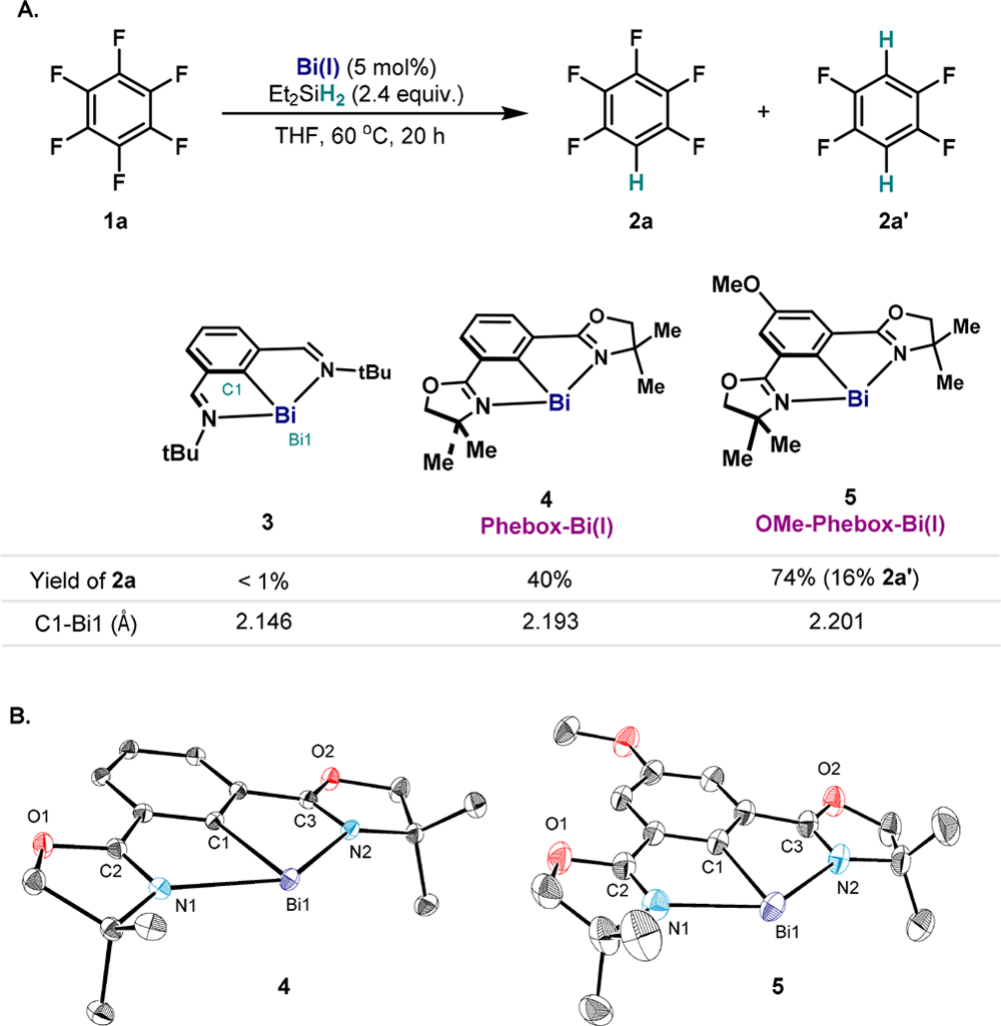
(A) HDF of **1a**; ^19^F NMR yields are given. (B) ORTEP drawings of **4** and **5**, with ellipsoids drawn at the 50% probability level. H atoms of **4** and **5**, the second molecule in the asymmetric unit of **4**, and disordered parts of **5** are omitted for clarity. Selected bond lengths (Å): for **4** (the bond lengths for the second molecule of **4** are given in brackets), Bi1–C1 2.189(3) [2.196(3)], Bi1–N1 2.525(3) [2.523(3)], Bi1–N2 2.503(3) [2.502(3)], N1–C2 1.288(4) [1.287(5)], N2–C3 1.288(4) [1.291(4)]; for **5**, Bi1–C1 2.201(2), Bi1–N1 2.5359(19), Bi1–N2 2.5142(18), N1–C2 1.282(3), N2–C3 1.284(3).

With these Bi(I) catalysts in hand, HDFs of other polyfluoroarenes were evaluated (**1b**–**1n**, Table 1). In general, HDF proceeds in high yields; however, the reaction parameters varied significantly depending on the substituents of the substrates.^18^ Pentafluoropyridine (**1b**) and pentafluorobenzenes with strong electron-withdrawing groups (CF_3_, CO_2_Me, and CN, **1c**–**1f**) underwent HDF readily at ambient temperature. Whereas **1b** reached full conversion in 1 h using **3**, the reaction finished within 2 min using **4** as catalyst. The high reactivity of **4** permitted lowering the catalyst loading to a remarkable 0.05 mol% while maintaining a high yield of **2b** (1640 TON). Di-, tri-, and tetra-HDFs occurred for **1f**–**1h**, **1j**, and **1k** when higher amount of Et_2_SiH_2_ (1.2–2.4 equiv.) were used. Several highly fluorinated phosphine compounds (**1i**–**1k**) utilized in various catalytic processes could be electronically fine-tuned through this HDF process.^21^ Partially fluorinated substrates (**2a** and **1n**) and substrate with electron-neutral functionality (**1l**) were also amenable to HDF using **5** as catalyst. No directing effect was observed in HDF of **1m**, thus providing orthogonal selectivity to transition-metal-catalyzed systems.^10f^ It should be mentioned that, similar to the reported systems based on transition metals, the HDF becomes sluggish when applied to polyfluoroarenes bearing electron-donating groups.^9e,10d,10i^ For instance, reaction of 2,3,4,5,6-pentafluorotoluene (**1o**) only delivered 2,3,5,6-tetrafluorotoluene (**2o**) in 3.5% yield after 3 days.

**Table 1:**
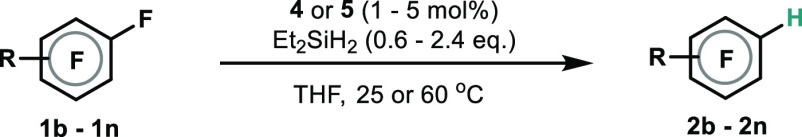

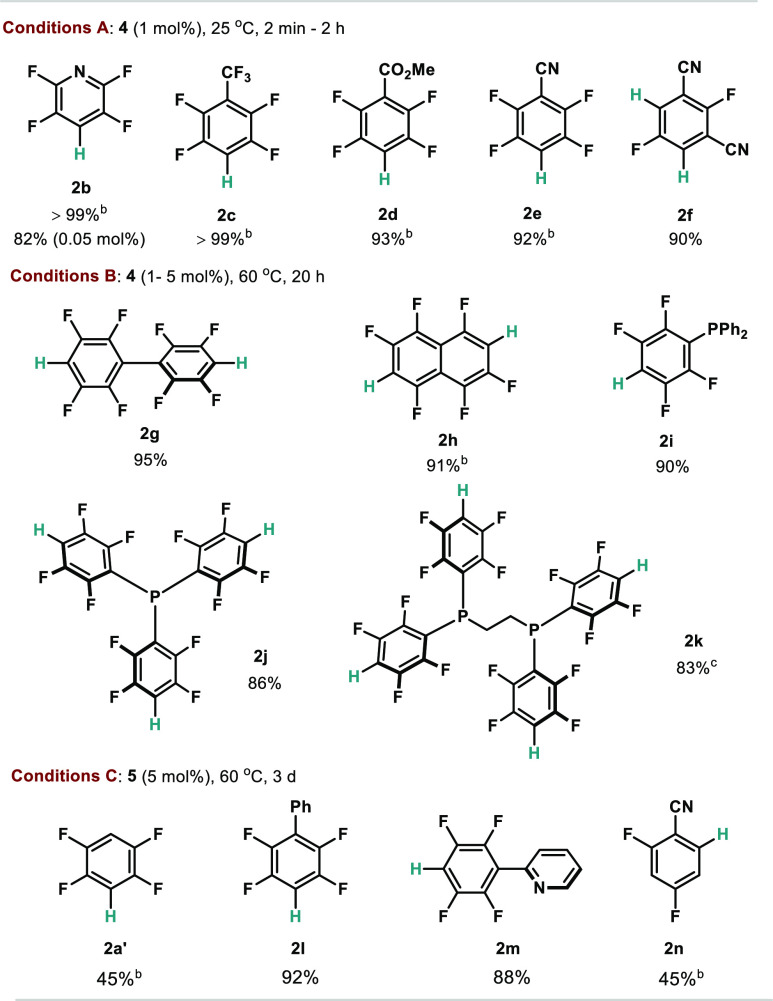
Scope of the Bi(I)-Catalyzed HDF^a^

a Reactions performed on 0.25 mmol scale of **1b**–**1n**.

b Yields calculated by quantitative ^19^F NMR using 4-fluorotoluene as internal standard.

c 0.20 mmol scale of **1k**.

In light of its demonstrated high reactivity, **1b** was chosen as the model compound to study the mechanism of the Bi(I)-catalyzed HDF reaction. First, Phebox-Bi(I) (**4**) was subjected to 1.0 equiv. of **1b** in THF-*d*_8_ (Figure 3A, *path a*). After 5 min, ^19^F NMR at 25 °C showed a distinct multiplet at −125.6 ppm, which is shifted dramatically compared to the *meta*-fluorines of **1b** and **2b** (**1b**, −163.0 ppm; **2b**, −141.7 ppm). However, such chemical shift is consistent with the *ortho*-fluorines of 4-tetrafluoropyridyl attached to Bi in the reported Bi(4-C_5_F_4_N)_3_ (−120.7 ppm)^22^ and to other electropositive centers (e.g., Mg,^14f^ Ni^23^). ^1^H–^19^F HOESY data at −40 °C further revealed the spatial proximity between these fluorines and two of the methyl groups of the Phebox backbone, suggesting the formation of Phebox-Bi(III)(4-tetrafluoropyridyl) fluoride (**8a**) via OA. However, the complex interconversions observed between **8a** and other Bi species precluded its complete characterization.^18^ Nevertheless, when this mixture was treated with 2.0 equiv. of Et_2_SiH_2_, regeneration of **4** (>99%) and formation of the HDF product **2b** (77%) were observed, manifesting the capacity of forging a C(sp^2^)–H bond through a Bi(I)/Bi(III) redox event.

**Figure 3:**
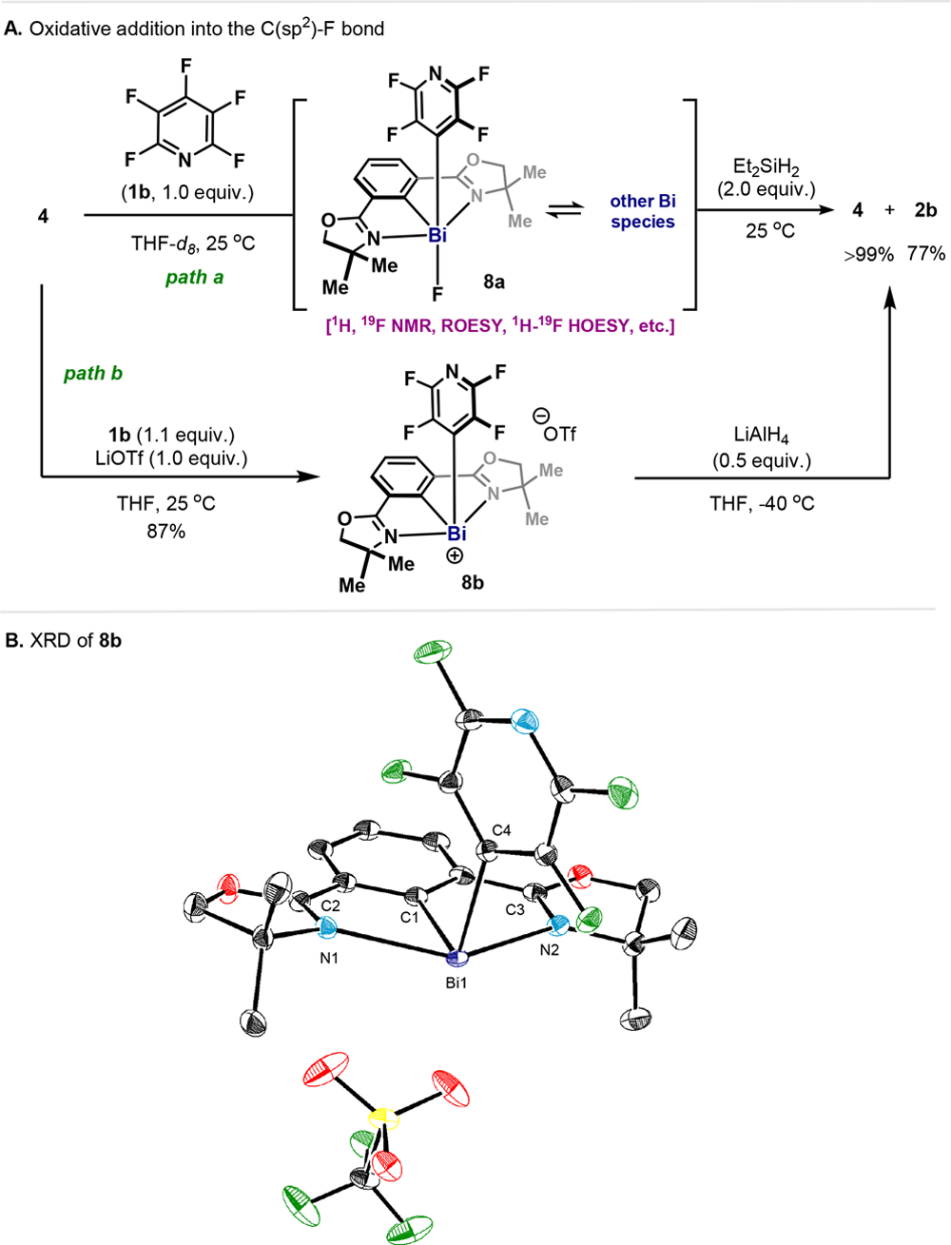
(A) OA of **4** with **1b**; *path a*: **4** (20.8 μmol) and **1b** (1.0 equiv.) in 0.5 mL of THF-*d*_8_ at 25 °C; *path b*: **4** (2.08 mmol), **1b** (1.1 equiv.) and LiOTf (1.0 equiv.) in 7.0 mL of THF at 25 °C. (B) ORTEP drawing of **8b**, with ellipsoids drawn at the 50% probability level. H atoms of **8b** are omitted for clarity. Selected bond lengths (Å) and angles (°): Bi1–C1 2.225(2), Bi1–C4 2.294(2), Bi1–N1 2.450(2), Bi1–N2 2.4779(19), N1–C2 1.280(3), N2–C3 1.286(3); C1–Bi1–C4 93.60(8).

It was reasoned that the reactivity of the fluoride after C–F cleavage played an important role in the observed equilibriums. Hence, it was envisaged that fluoride abstraction after OA would lead to a well-defined cationic bismuth species with higher stability. Indeed, when the same reaction was performed in the presence of 1.0 equiv. of LiOTf, the triflate salt **8b** was isolated in 87% yield (Figure 3A, *path b*). The attachment of the 4-tetrafluoropyridyl group to the Bi center results in the ^19^F signals of the *ortho*-fluorines appearing in a region (−121.4 ppm) similar to the observed shift of **8a**. Moreover, the observation of diastereotopic methyl groups and methylene protons in the oxazolines of **8b** by ^1^H NMR (CH_3_, 1.60 and 1.27 ppm; CH_2_, 4.59 and 4.56 ppm) confirms that the symmetry through the plane of Phebox ligand has been broken in **8b**. The X-ray crystal structure of **8b** confirms the weak interaction between the cationic Bi center and the triflate anion, as shown by the large distance between the closest oxygens of triflate and the Bi center (2.974 Å, ∑_cov_(Bi–O) = 2.14 Å,^24^Figure 3B). In spite of the cationic nature of **8b**, the Bi1–C4 bond is still polarized [2.294(2) Å].^25^ As a result, **8b** is highly moisture-sensitive, yielding [Phebox-Bi(OTf)]_2_O, **2b**, and other oxo-bismuth species upon hydrolysis.^18^ Similar reactivity has been observed for Bi(4-C_5_F_4_N)_3_^22^ and other perfluoro-aryl^26^ or -alkyl^27^ Bi(III) compounds. Although **8b** showed no reactivity toward hydrosilanes due to the absence of fluoride anion, reduction of **8b** with stronger metal hydrides (e.g., LiAlH_4_) readily yielded **4** and **2b** (Figure 3A, *path b*).

At this point, it was hypothesized that a Ar_2_Bi(III)-H was generated via LM of **8a** or **8b** with hydrosilanes or metal hydrides. Organobismuth(III) hydrides are usually unstable species,^28^ prone to H_2_ release and formation of metallic Bi,^29^ Bi(I),^6a,16^ or dimetallic Bi(II)–Bi(II) compounds.^7,30^ Reported by Power in 2000, (2,6-Mes_2_H_3_C_6_)_2_BiH represents the only stable and well-defined organobismuth hydride until now.^31^ This compound indicated an alternative reaction pathway, namely C–H/D bond formation, yielding stable dibismuthene [Ar–Bi(I)=Bi(I)–Ar] and Ar-H/D (Ar = 2,6-Mes_2_H_3_C_6_). Later, the hydride signal of this bismuth hydride was located at a remarkably deshielded position (19.39 ppm),^32^ which resulted from the spin-orbital heavy-atom effect on the light atom (SO-HALA effect).^30,33−35^ Treatment of **8b** with 0.5 equiv. of LiAlH_4_ at −78 °C resulted in instant formation of a new organobismuth species (Figure 4A, top). A broad singlet at 24.52 ppm in ^1^H NMR was detected (Figure 4B, top), suggesting that this species corresponds to Phebox-Bi(III)(4-tetrafluoropyridyl) hydride (**9**) with an electronic environment around Bi–H similar to that of the reported (2,6-Mes_2_H_3_C_6_)_2_BiH. In addition, **9** has an asymmetric and dynamic structure, as revealed by the considerably broadened NMR signals of the oxazolines (e.g., H-4, Figure 4B) and *ortho*-fluorines of the 4-tetrafluoropyridyl (−117.9 ppm).^18^ At –40 °C, **9** rapidly decayed into Phebox-Bi(I) (**4**) and HDF product (**2b**) in ca. 90% and 80% yields, indicating C(sp^2^)–H RE at the Bi center. Under catalytic conditions, **9** was the major species and remained relatively stable in concentration (Figure 4A and 4B, middle). Structural information on **9** was gathered from 2D NMR data of the reaction mixture. Particularly, C-5 (157.8 ppm) of **9** is noticeably more shielded than those of **4**, **6**, and **8b** (**4**, 172.7 ppm; **6**, 181.9 ppm; **8b**, 182.3 ppm), but similar to that of the precursor Phebox-Br (**10**, 161.9 ppm). These electronic differences suggest that the oxazolines remain uncoordinated to the Bi center in **9**, permitting the Bi center to adopt a trigonal pyramidal geometry. To further interrogate the nature of the unusual downfield proton signal, the catalytic reaction was performed using Et_2_SiD_2_. While all the signals assigned to **9** could be observed, the signal at 24.52 ppm did not appear in ^1^H NMR, suggesting the formation of corresponding bismuth deuteride **9-D** (Figure 4A and 4B, bottom). As expected, decomposition of **9-D** results in formation of **2b-D**. It is important to point out that this is a distinct example where NMR spectroscopic data supports the involvement of an organobismuth hydride in a catalytic process, resulting in the formation of a C–H bond.

**Figure 4:**
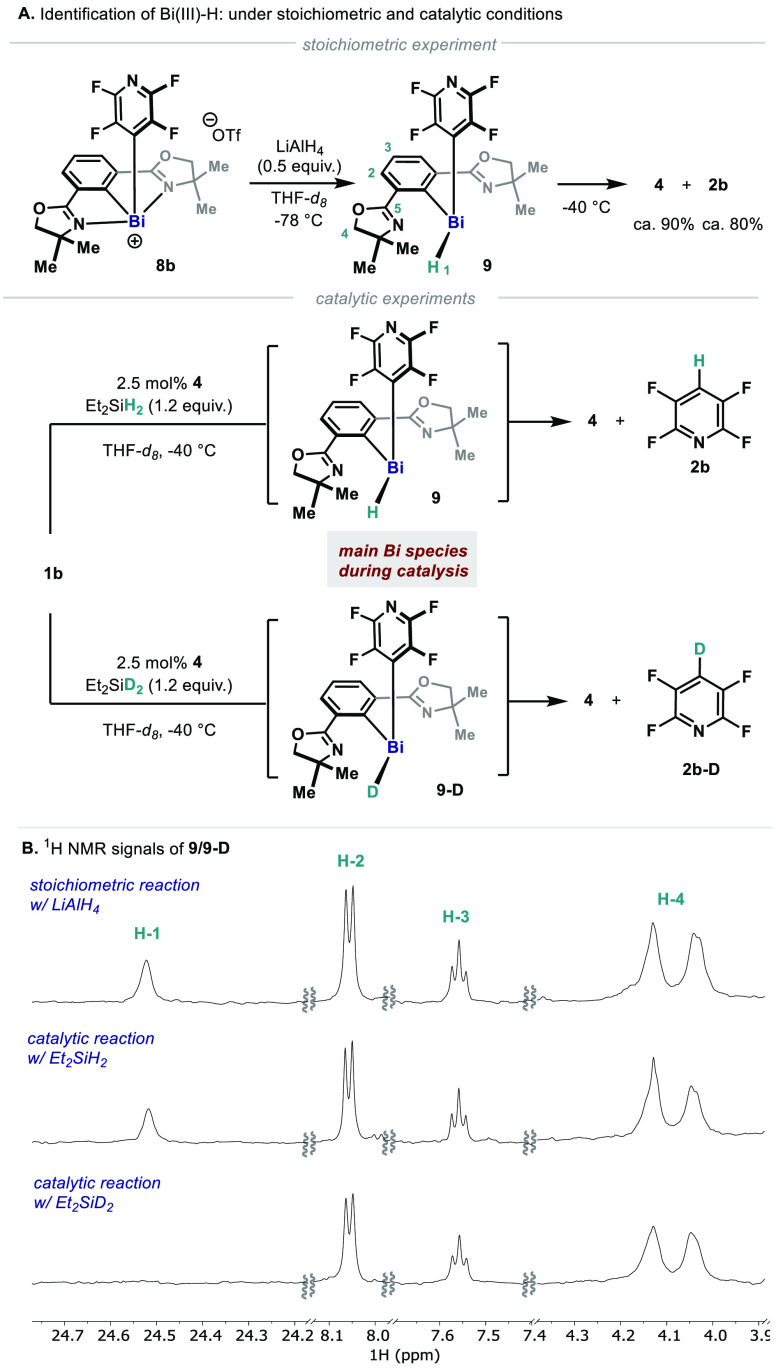
(A) Proposed Bi-H/D intermediates (**9**/**9-D**) and C-H/D reductive elimination. (B) ^1^H NMR spectra of **9**/**9-D** at −40 °C; top: LiAlH_4_ reduction of **8b**; middle: catalytic HDF of **1b**; bottom: catalytic HDF of **1b** using Et_2_SiD_2_.

Taking **1b** as an example, a Bi(I)/Bi(III) catalytic cycle can be proposed (Figure 5). Bismuthinidene **4** undergoes OA to **1b**, delivering the Bi(III) intermediate **8a**. Subsequent F/H LM between **8a** and Et_2_SiH_2_ leads to the formation of diorganobismuth hydride (**9**) and fluorosilane. The catalytic redox loop is closed with RE from **9**, releasing HDF product (**2b**) and regenerating Bi(I) (**4**).

**Figure 5:**
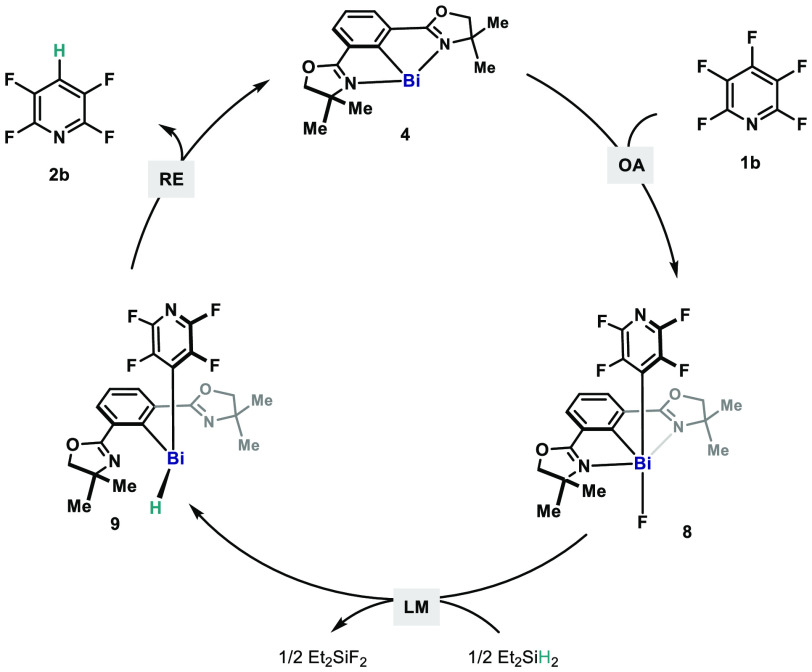
Proposed catalytic cycle for Bi(I)-catalyzed HDF.

In conclusion, we present that bismuthinidenes supported by a Phebox ligand scaffold facilitate catalytic HDF reaction of a variety of polyfluoroarenes under mild conditions. Mechanistic investigations enabled the identification of the intermediates involved, both after C–F cleavage (**8b**) and prior to C–H bond formation (**9**). These findings support a distinct Bi(I)/Bi(III) redox cycle where Bi centers manifest oxidative addition, ligand metathesis, and reductive elimination steps, conventionally exploited in transition-metal catalysis. The facile cycling through three elementary organometallic steps in the Bi(I)/Bi(III) redox manifold serves as a response to the long-standing challenge in the field of redox catalysis using low-valent main-group compounds, potentially enabling a myriad of catalytic redox processes beyond HDF.

## Supporting Information Available

Experimental procedures and analytical data (^1^H, ^13^C, ^19^F, and ^31^P NMR, HRMS and X-ray crystallographic details) for new compounds (PDF)

## Accession Codes

CCDC 2091964–2091968 contain the supplementary crystallographic data for this paper. These data can be obtained free of charge via www.ccdc.cam.ac.uk/data_request/cif, or by emailing data_request@ccdc.cam.ac.uk, or by contacting The Cambridge Crystallographic Data Centre, 12 Union Road, Cambridge CB2 1EZ, UK; fax: +44 1223 336033.
