# Supplementary material for: Catalytic Hydrodefluorination via Oxidative Addition, Ligand Metathesis, and Reductive Elimination at Bi(I)/Bi(III) Centers
Source: J Am Chem Soc. 2021 Aug 6;143(32):12487–93. doi: 10.1021/jacs.1c06735 (PMC8377712; doi:10.1021/jacs.1c06735)
Supplement: Supplementary file 1 — ja1c06735_si_001.pdf [file ja1c06735_si_001.pdf]

Supporting Information

Catalytic Hydrodefluorination via Oxidative Addition, Ligand Metathesis and Reductive Elimination at Bi(I)/Bi(III) Centers

Yue Pang, Markus Leutzsch, Nils Nöthling, Felix Katzenburg and Josep Cornella*

Max-Planck-Institut für Kohlenforschung, D-45470 Mülheim an der Ruhr, Germany

cornella@kofo.mpg.de

Table of Contents

1. General Information 3

2. Synthesis and Characterization Data of Bismuth Catalysts 4

2.1. Synthesis of Phebox-Bi(I) (**4**) 4

2.2. Synthesis of OMe-Phebox-Bi(I) (**5**) 8

3. Bi(I)-catalyzed Hydrodefluorination Reactions 11

3.1. Optimization of the Reaction Conditions 11

3.2. General Procedure for HDF 12

3.3. Results and Characterization Data of HDF Products 12

4. Mechanistic Studies of Bi(I)-catalyzed HDF Reactions 23

4.1. Mechanistic Proposal: Bi(I)/Bi(III) Redox Catalytic Cycle 23

4.2. Oxidative Addition of Phebox-Bi(I) (**4**) with Pentafluoropyridine (**1b**) 24

4.2.1. Procedure of OA and Stoichiometric HDF Reactivity 24

4.2.2. NMR Data of **8a**, **S4** and **S5** 25

4.2.3. NMR Analysis of the OA Reaction 29

4.3. Characterization and Reactivities of Phebox-Bi(4-tetrafluoropyridyl) triflate (**8b**) . 41

4.4. Phebox-Bi(4-tetrafluoropyridyl) hydride (**9**) and C-H Reductive Elimination 44

4.4.1. Generation of **9** via LAH reduction of **8b** 44

4.4.2. NMR Characterization of **9** under the Catalytic Condition 48

4.4.3. Characterization Data of **9** and Structural Information of **9** Obtained from NMR Studies 53

4.4.4. Deuterium Kinetic Experiment 56

5. X-ray Crystallographic Studies 58

5.1. Single Crystal Structure Analysis of Complex **4** 58

5.2. Single Crystal Structure Analysis of Complex **5** 63

5.3. Single Crystal Structure Analysis of Complex **6** 68

5.4. Single Crystal Structure Analysis of Complex **7 · 0.5 THF** 72

5.5. Single Crystal Structure Analysis of Complex **8b** 80

5.6. Clarification for Compound **3**, **4** and **5** 85

6. NMR Spectra 87

6.1. NMR Spectra of Bismuth Compounds and the Precursors 87

6.2. ^{19}F NMR Spectra for Determining the NMR Yields of HDF Reactions 100

6.3. NMR Spectra of the Isolated HDF Products 104

7. References 122

1. General Information

Unless otherwise stated, all manipulations were performed under argon using standard Schlenk line techniques or in a MBraun argon-filled glove box.

Instruments: Flash chromatography: Merck silica gel 60 (40-63 µm). ESI-MS: ESQ 3000 (Bruker). Accurate mass determinations: Bruker APEX III FT-MS (7 T magnet) or MAT 95 (Finnigan). Melting points were measured with an EZ-Melt Automated Melting Point Apparatus from Standford Research Systems.

NMR data were recorded using a Bruker AVIII HD 300 MHz, Bruker AVIII HD 400 MHz, Bruker AVIII 500 MHz or Bruker AVNeo 600 MHz NMR spectrometer. ^{1}H and ^{13}C chemical shifts are reported relative to the solvent residual peaks as an internal reference. For ^{1}H NMR the following residual proton peaks of the deuterated solvents were used: $CDCl_3$, $\delta_H(CHCl_3)$ 7.260; THF-d_8, $\delta_H((CD_2)_3CHDO)$ 3.580; CD_3CN, $\delta_H(CHD_2CN)$ 1.940; DMSO-d_6, $\delta_H(CD_2HSOCD_3)$ 2.500. For ^{13}C NMR: $CDCl_3$, δ 77.16; THF-d_8, δ 67.57; CD_3CN, δ 1.32; DMSO-d_6, δ 39.52. ^{13}C and ^{31}P NMR spectra were acquired with broadband ^{1}H decoupling unless mentioned otherwise. ^{19}F NMR spectra measured at 282 MHz and 470 MHz were generally acquired with broadband ^{1}H decoupling. ^{19}F NMR spectra at 565 MHz were measured without ^{1}H decoupling due to probe limitations. The chemical shifts of ^{19}F NMR spectra extracted from reaction mixtures in pure THF with a C_6D_6 capillary for locking are reported relative (4-fluorotoluene: δ -119.8). All other ^{19}F spectra as well as ^{31}P NMR data was referenced indirectly with the Bruker 'xiref' macro to a calibrated ^{1}H NMR spectrum.[1] ^{19}F shifts are reported relative to $\delta(CFCl_3)$ = 0 ppm ($\Xi(^{19}F)$= 94.094011%) and ^{31}P shifts are reported relative to $\delta(H_3PO_4)$ = 0 ppm ($\Xi(^{31}P)$= 40.480742%).

^{19}F NMR yields were determined by using 4-fluorotoluene as internal standard.

For quantitative ^{1}H and $^{19}F\{^1H\}$ data, the short pulse angles (30°) were used and the offset was set in the center of the NMR spectrum. For each spectrum 8 (^{1}H) or 16 (for ^{19}F) FIDs were averaged to obtain a good SNR. Between scans, a long relaxation delay was used (60s for ^{1}H, 70s for ^{19}F) to ensure full relaxation between the scans.

Chemicals: THF, *n*-pentane, THF-d_8 and other solvents were distilled from the proper drying agents (THF and THF-d_8, Na/benzophenone; *n*-pentane, CaH_2) and stored over 4 Å molecular sieves under argon prior to use. 4 Å molecular sieves were activated at 200 °C under high vacuum (1×10^{-4} bar) for 3 days. Anhydrous $BiCl_3$ (99.9%, trace metal basis) was purchased from Alfa Aesar. Cobaltocene (min. 98%) was purchased from Strem Chemicals and stored in the freezer of the glove box prior to use. Bismuthinidene **1** was prepared by the reported method.[2] Unless otherwise noted, all reagents were obtained from commercial suppliers and used without further purification. Et_2SiH_2, pentafluoropyridine (**1b**) and LiOTf were purchased from Sigma-Aldrich Chemie GmbH. All liquid hydrosilanes and polyfluoroarenes were degassed by repeated freeze-pump-thaw cycles and stored over 4 Å molecular sieves under argon prior to use.

2. Synthesis and Characterization Data of Bismuth Catalysts

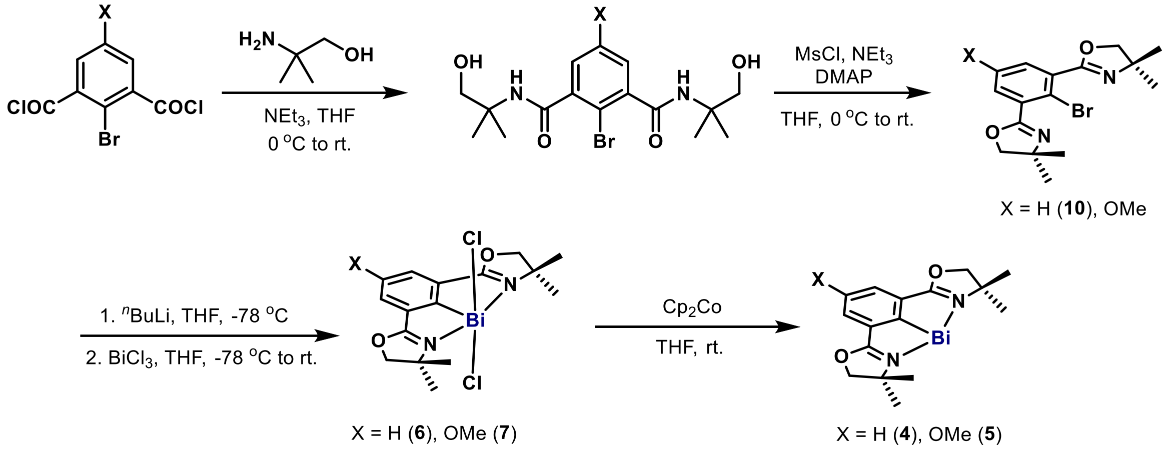

Scheme S1. The synthetic route to Phebox-Bi(I) (**4**) and OMe-Phebox-Bi(I) (**5**)

2.1. Synthesis of Phebox-Bi(I) (**4**)

2-Bromo-*N,N'*-bis(1-hydroxy-2-methylpropan-2-yl)isophthalamide (**S1**)

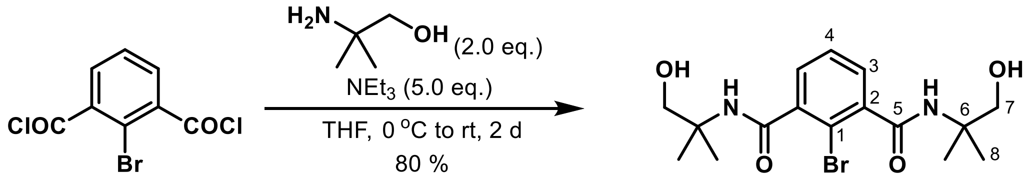

Procedure: To a 250 mL argon-filled Schlenk flask 2-amino-2-methylpropan-1-ol (1.9 g, 21.3 mmol, 2.0 equiv.), anhydrous triethylamine (7.4 mL, 53.2 mmol, 5.0 equiv.) and 50 mL anhydrous THF were added under argon. At 0 °C, a solution of 2-bromoisophthaloyl dichloride[3] in 20 mL THF (3.0 g, 10.6 mmol) was added dropwise to the reaction mixture. After stirring at room temperature for 2 days, the mixture was filtered and the crude product was washed with ca. 400 mL DCM until NEt_3-HCl was removed completely as judged by ^{1}H NMR. After drying under high vacuum, **S1** (3.3 g, 80 %) was obtained as a white solid.

In the previous procedures, the title compound was used in the next step without further purification.[4]

^{1}H NMR (400 MHz, DMSO-*d*$_6$): δ 7.73 (s, 2H, NH), 7.39 (dd, *J* = 8.5, 6.2 Hz, 1H, H-4), 7.35 – 7.29 (m, 2H, H-3), 4.80 (t, *J* = 6.1 Hz, 2H, OH), 3.47 (d, *J* = 6.1 Hz, 4H, H-7), 1.29 (s, 12H, H-8).

^{13}C NMR (101 MHz, DMSO-*d*$_6$): δ 167.1 (C-5), 140.7 (C-2), 128.2 (C-3), 127.2 (C-4), 115.8 (C-1), 67.5 (C-7), 55.2 (C-6), 23.4 (C-8).

M.p.: 193.1 – 194.5 °C.

HRMS (ESI): calc'd for $C_{16}H_{23}BrN_2O_4Na^+$ $[M+Na]^+$ 409.07334; found 409.07340.

Phebox-Br (**10**)

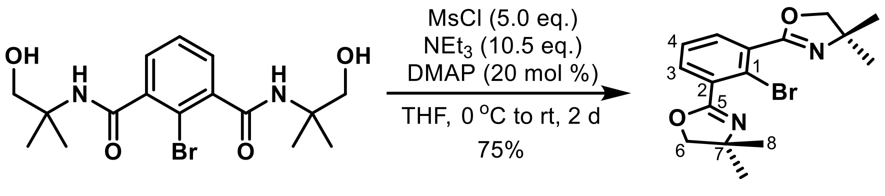

Phebox-Br (**10**)

Procedure: A 250 mL flame-dried Schlenk flask was charged with **S1** (2.5 g, 6.46 mmol) and DMAP (158 mg, 1.29 mmol, 20 mol %), evacuated and backfilled with argon. Anhydrous triethylamine (9.4 mL, 67.8 mmol, 10.5 equiv.) and 120 mL anhydrous THF were added under argon. At 0 °C, freshly distilled methanesulfonyl chloride (MsCl, 2.5 mL, 32.3 mmol, 5.0 equiv.) was added to the reaction mixture dropwise. After stirring at room temperature for 2 days, the mixture was filtered through a Celite pad and the pad was washed with 200 mL EtOAc. The filtrate was evaporated to dryness, re-dissolved in 200 mL EtOAc and washed with saturated Na_2CO_3 solution and brine. The organic layer was dried over anhydrous Na_2SO_4 and concentrated *in vacuo*. The residue was purified by column chromatography on silica gel with MTBE as eluent to afford 1.70 g **Phebox-Br** as a white crystalline solid in 75% yield.

^{1}H NMR (300 MHz, $CDCl_3$): δ 7.61 (d, *J* = 7.7 Hz, 2H, H-3), 7.35 (t, *J* = 7.7 Hz, 1H, H-4), 4.13 (s, 4H, H-6), 1.40 (s, 12H, H-8).

^{13}C NMR (75 MHz, $CDCl_3$): δ 161.9 (C-5), 132.6, 132.4, 127.0, 121.5, 79.7 (C-6), 68.2 (C-7), 28.3 (C-8).

M.p.: 105.4 – 107.8 °C.

HRMS (ESI): calc'd for $C_{16}H_{20}BrN_2O_2^+$ $[M+H]^+$ 351.07027; found 351.07024.

The spectral data matched with those reported in the literature.[4]

Phebox-BiCl$_2$ (**6**)

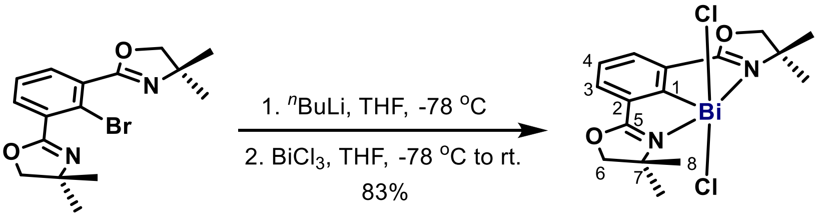

Phebox-Br (**10**) Phebox-BiCl$_2$ (**6**)

Phebox-BiCl$_2$ (**6**) was prepared by using a modified literature procedure.[3, 5]

Procedure: To a solution of **10** (1.60 g, 4.56 mmol, 80 mL anhydrous THF) at -78 °C, ^{n}BuLi (1.84 mL, 2.6 M in hexane, 4.78 mmol, 1.05 equiv.) was added dropwise under argon. The resulting bright orange solution was stirred at this temperature for 2 h and then transferred to a precooled solution of $BiCl_3$ (1.51 g, 4.78 mmol, 1.05 equiv., in 60 mL anhydrous THF) via a

cannula at -78 °C under argon. The resulting yellow solution was stirred at room temperature for 1 day, during which it turned into a white muddy suspension.

The work-up was carried out without the protection of argon. The reaction mixture was evaporated to dryness, re-dissolved in DCM and filtered through a Celite-pad. The filtrate was concentrated *in vacuo* to ca. 20 mL and hexane was used for precipitation of **6**. The crude product was filtered and washed with EtOAc and Et_2O to give Phebox-$BiCl_2$ (**6**) as a white powder (2.09 g, 83%). The titled compound contains DCM residue which cannot be removed under high vacuum (**6**/DCM = 10:1).

^{1}H NMR (400 MHz, $CDCl_3$): δ 8.24 (d, *J* = 7.7 Hz, 2H, H-3), 7.70 (t, *J* = 7.7 Hz, 1H, H-4), 4.50 (s, 4H, H-6), 1.60 (s, 12H, H-8).

^{13}C NMR (101 MHz, $CDCl_3$): δ 206.1 (C-1), 181.9 (C-5), 134.8 (C-3), 131.4 (C-2), 129.2 (C-4), 83.0 (C-6), 67.5 (C-7), 28.8 (C-8).

M.p.: > 300 °C.

HRMS (ESI): calc'd for $C_{16}H_{19}N_2O_2BiCl^+$ $[M-Cl]^+$ 515.09334; found 515.09339.

Single crystals of **6** suitable for X-ray crystallographic analysis were obtained by slow diffusion of *n*-pentane into a concentrated $CHCl_3$ solution of **6** at 0 °C.

Phebox-Bi(I) (**4**)

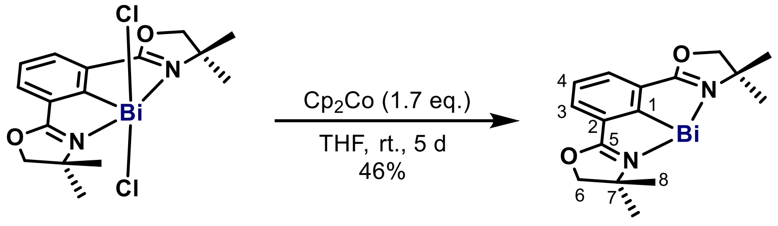

Phebox-BiCl2 (**6**) Phebox-Bi(I) (**4**)

Phebox-Bi(I) (**4**) was prepared by a modified literature method.[3] Due to *the limited solubility of **6** in THF*, full consumption of Cp_2Co was not achieved within two days as judged by quantitative ^{1}H NMR with 1,3,5-trimethoxylbenzene as internal standard. Therefore, the reaction was prolonged to 5 days.

Procedure: In a glove box, Phebox-$BiCl_2$ (**6**, 800 mg, 1.45 mmol) and THF (anhydrous and degassed, 50 mL) were added to a 100 mL flame-dried Schlenk flask. At room temperature, cobaltocene (467 mg, 2.47 mmol, 1.7 equiv.) was added to this suspension under stirring. The Schlenk flask was taken out of the glove box. The reaction mixture was allowed to react for 5 days in the darkness, during which the solution turned dark green and yellow cobaltocenium chloride precipitated. The solvent was removed *in vacuo* and the residue was re-dissolved in *n*-pentane (anhydrous and degassed, ca. 70 mL). The insoluble salts were filtered off under argon and the filtrate was concentrated *in vacuo* to ca. 15 mL. Crystallization at -35 °C overnight gave Phebox-Bi(I) (**4**) as a dark green crystalline solid (321 mg, 46%).

^{1}H NMR (400 MHz, THF-*d_8*): δ 7.91 (d, *J* = 7.5 Hz, 2H, H-3), 6.73 (t, *J* = 7.5 Hz, 1H, H-4), 4.48 (s, 4H, H-6), 1.49 (s, 12H, H-8).

^{13}C NMR (101 MHz, THF-*d*$_8$): δ 193.9 (C-1), 172.7 (C-5), 131.5 (C-2), 130.8 (C-3), 124.2 (C-4), 80.8 (C-6), 69.2 (C-7), 30.8 (C-8).

M.p.: > 160 ^{o}C (dec.).

HRMS (ESI): calc'd for $C_{16}H_{19}BiN_2O_2^+$ $[M]^+$ 480.12449; found 480.12451.

Single crystals of **4** suitable for X-ray crystallographic analysis were obtained by slow cooling of a concentrate *n*-pentane solution of **4** to -35 ^{o}C over 40 h.

2.2. Synthesis of OMe-Phebox-Bi(I) (**5**)

2-Bromo-*N,N'*-bis(1-hydroxy-2-methylpropan-2-yl)-5-methoxyisophthalamide (**S2**)

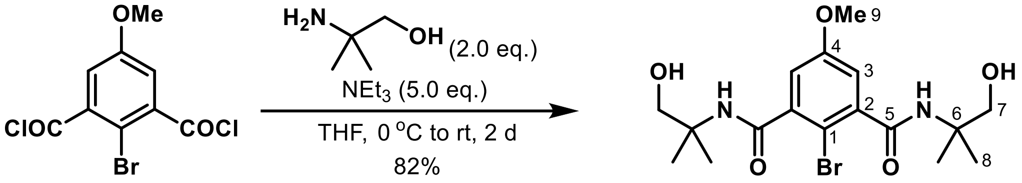

2-bromo-5-methoxyisophthaloyl dichloride was prepared according to literature procedures and used without further purification.[3, 6]

Procedure: To a 250 mL argon-filled Schlenk flask 2-amino-2-methylpropan-1-ol (0.82 g, 9.23 mmol, 2.0 equiv.), dry triethylamine (3.2 mL, 23.1 mmol, 5.0 equiv.) and 20 mL anhydrous THF were added. At 0 °C, a solution of 2-bromo-5-methoxyisophthaloyl dichloride (1.44 g, 4.62 mmol, in 10 mL THF) was added dropwise to the reaction mixture. After stirring at room temperature for 2 days, the mixture was evaporated to dryness. The residue was purified by column chromatography on silica gel with acetone as eluent to afford 1.57 g **S2** as a white solid in 82% yield.

^{1}H NMR (300 MHz, DMSO-*d*$_6$): δ 7.68 (s, 2H, N-H), 6.91 (s, 2H, H-3), 4.78 (d, *J* = 6.5 Hz, 2H, O-H), 3.79 (s, 3H, H-9), 3.47 (d, *J* = 6.1 Hz, 4H, H-7), 1.29 (s, 12H, H-8).

^{13}C NMR (75 MHz, DMSO-*d*$_6$): δ 166.7, 157.9, 141.6, 114.0, 106.2, 67.4, 55.8, 55.2, 23.4.

M.p.: 209.3 – 214.2 °C.

HRMS (ESI): calc'd for $C_{17}H_{25}BrN_2O_5Na^+$ $[M+Na]^+$ 439.08391; found 439.08408.

OMe-Phebox-Br (**S3**)

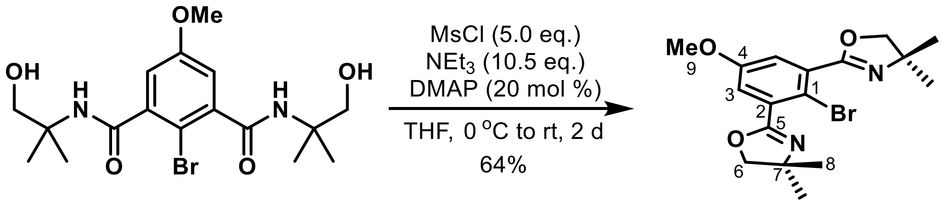

OMe-Phebox-Br (**S3**)

A 250 mL flame-dried Schlenk flask was charged with **S2** (1.56 g, 3.74 mmol) and DMAP (91 mg, 0.75 mmol, 20 mol %), evacuated and backfilled with argon. Anhydrous triethylamine (5.5 mL, 39.3 mmol, 10.5 equiv.) and 70 mL anhydrous THF were added under argon. At 0 °C, freshly distilled MsCl (1.45 mL, 18.7 mmol, 5.0 equiv.) was added to the reaction mixture dropwise. After stirring at room temperature for 2 days, the mixture was filtered through a Celite pad and the pad was washed with EtOAc. The filtrate was evaporated to dryness, re-dissolved in EtOAc and washed with saturated Na_2CO_3 solution and brine. The organic layer was dried over anhydrous Na_2SO_4 and concentrated *in vacuo*. The residue was purified by column chromatography on silica gel (hexane/EtOAc = 2:1, *v*/*v*) to afford 0.91 g **S3** as a white crystalline solid in 64% yield.

^{1}H NMR (300 MHz, $CDCl_3$): δ 7.17 (s, 2H, H-3), 4.13 (s, 4H, H-6), 3.81 (s, 3H, H-9), 1.40 (s, 12H, H-8).

^{13}C NMR (75 MHz, $CDCl_3$): δ 161.9, 158.3, 133.1, 118.5, 111.7, 79.7, 68.2, 56.0, 28.3.

M.p.: 77.4 – 79.0 °C.

HRMS (ESI): calc'd for $C_{17}H_{22}BrN_2O_3^+$ $[M+H]^+$ 381.08083; found 381.08063.

The spectral data matched with those reported in the literature.[6b]

OMe-Phebox-$BiCl_2$ (**7**)

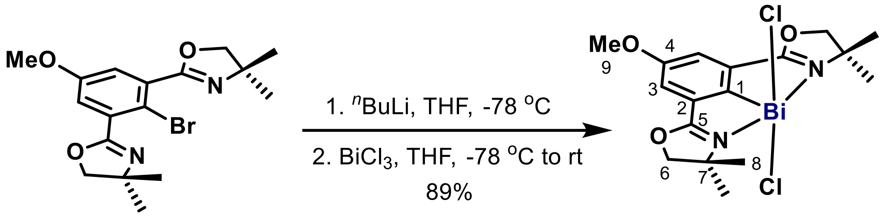

OMe-Phebox-Br (**S3**)

OMe-Phebox-$BiCl_2$ (**7**)

OMe-Phebox-$BiCl_2$ (**7**) was prepared by using a modified literature procedure.[3, 5]

Procedure: To a solution of **S3** (800 mg, 2.10 mmol, in 50 mL anhydrous THF) at -78 °C, ^{n}BuLi (0.85 mL, 2.6 M in hexane, 2.20 mmol, 1.05 equiv.) was added dropwise under argon. The resulting bright yellow solution was stirred at this temperature for 2 h and then transferred to a solution of $BiCl_3$ (695 mg, 2.20 mmol, 1.05 equiv., in 30 mL anhydrous THF) via a cannula at -78 °C under argon. The resulting yellow solution was stirred at room temperature for one day, during which it turned into a colorless solution with black precipitates.

The work-up was carried out without the protection of argon, however, **7** is moisture-sensitive to some degree. The reaction mixture was evaporated to dryness, re-dissolved in DCM and filtered through a Celite bed. The filtrate was concentrated *in vacuo* to ca. 8 mL and hexane was used for the precipitation of **7**. The crude product was filtered and washed with hexane and Et_2O. To remove the DCM residue, the product was re-dissolved in anhydrous THF and evaporated to dryness. **7** was obtained as a pale yellow powder (1.08 g, 89%). **7** contains THF residues which cannot be removed under high vacuum (**7**/THF = 2.5:1).

^{1}H NMR (400 MHz, $CDCl_3$): δ 7.65 (s, 2H, H-3), 4.48 (s, 4H, H-6), 3.86 (s, 3H, H-9), 1.57 (s, 12H, H-8).

^{13}C NMR (101 MHz, $CDCl_3$): δ 198.2 (C-1), 181.2 (C-5), 160.7 (C-4), 133.3 (C-2), 121.1 (C-3), 83.0 (C-6), 67.5 (C-7), 56.2 (C-9), 28.7 (C-8).

M.p.: > 260 °C (dec.).

HRMS (ESI): calc'd for $C_{17}H_{21}N_2O_3BiCl^+$ $[M-Cl]^+$ 545.10390; found 545.10382.

Single crystals of **7** suitable for X-ray crystallographic analysis were obtained by slow diffusion of *n*-pentane into a THF solution of **7** under argon at room temperature.

OMe-Phebox-Bi(I) (**5**)

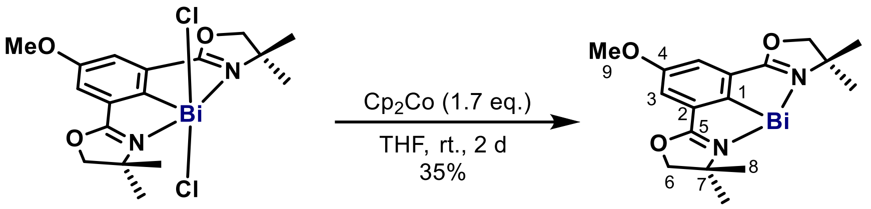

OMe-Phebox-BiCl$_2$ (**7**) OMe-Phebox-Bi(I) (**5**)

OMe-Phebox-Bi(I) (**5**) was prepared by the literature methods with modifications.[3]

Procedure: In a glove box, **7** (300 mg, 0.52 mmol) and THF (anhydrous and degassed, 15 mL) were added to a 50 mL flame-dried Schlenk flask. At room temperature, cobaltocene (166 mg, 0.88 mmol, 1.7 equiv.) was added to the solution under stirring. The Schlenk flask was taken out of the glove box. The reaction mixture was allowed to react for 2 days in the darkness, during which the solution turned dark green and yellow cobaltocenium chloride precipitated. The solvent was removed *in vacuo* and the residue was re-dissolved in *n*-pentane (anhydrous and degassed, ca. 30 mL). The insoluble salts were filtered off under argon and the filtrate was concentrated *in vacuo* to ca. 10 mL. Crystallization at -35 ^{o}C overnight gave **5** as a dark blue crystalline solid (92 mg, 35%).

^{1}H NMR (400 MHz, THF-*d$_8$*): δ 7.66 (s, 2H, H-3), 4.48 (s, 4H, H-6), 3.85 (s, 3H, H-9), 1.49 (s, 12H, H-8).

^{13}C NMR (101 MHz, THF-*d$_8$*): δ 179.7 (C-1), 172.0 (C-5), 160.1 (C-4), 132.0 (C-2), 117.4 (C-3), 80.8 (C-6), 69.5 (C-7), 56.4 (C-9), 30.8 (C-8).

M.p.: > 150 ^{o}C (dec.).

HRMS (ESI): calc'd for $C_{17}H_{21}BiN_2O_3^+$ $[M]^+$ 510.13505; found 510.13492.

Single crystals of **5** suitable for X-ray crystallographic analysis were obtained by slow cooling of a concentrate *n*-pentane solution of **5** to -35 ^{o}C over 20 h.

3. Bi(I)-catalyzed Hydrodefluorination Reactions

3.1. Optimization of the Reaction Conditions

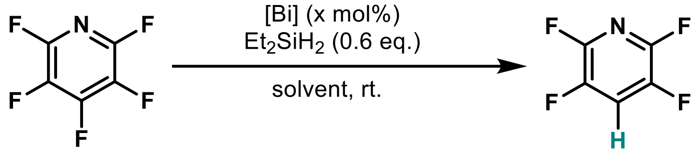

1b 2b

Table S1. HDF of **1b** using different Bi(I) and solvents[a]

Entry	[Bi] (mol %)	solv.	Time	Yield (%)[b]
1	--	THF	5 d	0
2	**3** (1)	THF	1 h (2 h)	88 (> 99)
3	**4** (1)	THF	< 2 min	> 99
4	**5** (1)	THF	< 2 min	> 99
5	**4** (1)	DMA	< 2 min	53
6	**4** (1)	MeCN	< 2 min	98
7	**4** (1)	toluene	< 1 h	> 99
8	**4** (1)	Et_2O	< 1 h	> 99
9	**4** (1)	*n*-pentane	< 1 h	> 99
10	**4** (0.1)	THF	< 1 h	> 99
11	**4** (0.05)	THF	14 h	82

[a]**1b** (0.25 mmol), 1.25 mL solvent;

[b]Yields calculated by quantitative ^{19}F NMR using 1.0 equiv. 4-fluorotoluene as internal standard.

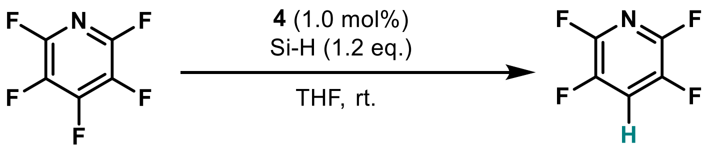

1b 2b

Table S2. HDF of **1b** using different hydrosilanes[a]

Entry	Si-H (equiv.)	Time	Yield (%)[b]
1	--	17 h	0
2	Et_2SiH_2 (0.6)	< 2 min	> 99
3	Ph_3SiH (1.2)	< 2 min	> 99
4	$MePh_2SiH$ (1.2)	< 10 min	> 99
5	Me_2PhSiH (1.2)	10 (30) min	89 (> 99)
6	Et_3SiH (1.2)	17 h	98
7	$(EtO)_3SiH$ (1.2)	< 10 min	> 99
8	$PhSiH_3$ (0.4)	17 h	> 99
9	PMHS (1.2)	17 h	> 99

[a]**1b** (0.25 mmol), 1.25 mL THF;

[b]Yields calculated by quantitative ^{19}F NMR using 1.0 equiv. 4-fluorotoluene as internal standard.

3.2. General Procedure for HDF

In a glovebox, polyfluoroarenes (**1a** – **1o**, 0.25 mmol), Et_2SiH_2 (0.6 – 2.4 equiv., 0.15 – 0.60 mmol) and THF (dry and degassed) were added to a 10 mL reaction vial. To the reaction mixture a THF stock solution of Bi(I) (**4** or **5**, 0.025 M, 100 – 500 µL, 1 – 5 mol%) was added upon stirring. The total volume of the THF solution is 1.25 mL. The vial was taken out of the glovebox and sealed with parafilm. After reaction at room temperature or 60 °C, the HDF product was purified by column chromatography. If HDF product is volatile, 4-fluorotoluene (27.5 mg, 0.25 mmol, 1.0 equiv.) was added as internal standard and the reaction mixture was analyzed by quantitative ^{19}F NMR with a sealed glass capillary containing C_6D_6.

3.3. Results and Characterization Data of HDF Products

HDF of hexafluorobenzene (**1a**)

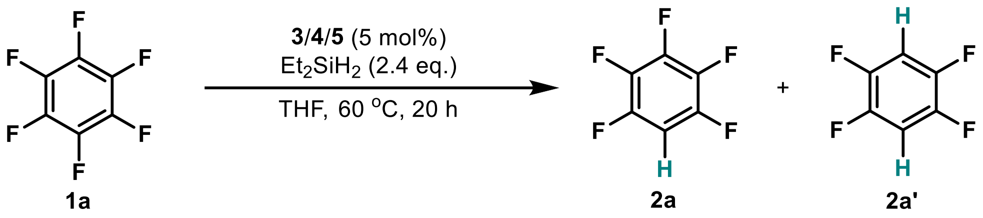

Bi(I)	2a	2a'
**3**	<1%	0
**4**	40%	1%
**5**	74%	16%

Following the general procedure, the reaction was performed with hexafluorobenzene (46.5 mg, 0.25 mmol), Et_2SiH_2 (52.9 mg, 0.60 mmol, 2.4 equiv.) and Bi(I) **3**/**4**/**5** (5.7/6.0/6.4 mg, 12.5 µmol, 5 mol%) at 60 °C for 20 h. Quantitative ^{19}F NMR revealed that pentafluorobenzene (**2a**) and 1,2,4,5-tetrafluorobenzene (**2a'**) were formed in <1 %/40%/74% and 0/1%/16% yields using **3**/**4**/**5** as catalysts.

Pentafluorobenzene (**2a**)

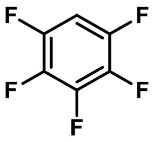

^{19}F NMR (470 MHz, in THF with a sealed glass capillary containing C_6D_6): δ -140.2 (dd, J = 22.2, 8.6 Hz, 2F), -156.3 (t, J = 20.0 Hz, 1F), -164.1 – -164.3 (m, 2F).

The spectral data matched with those reported in the literature.[7]

1,2,4,5-Tetrafluorobenzene (**2a'**)

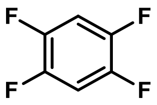

^{19}F NMR (470 MHz, in THF with a sealed glass capillary containing C_6D_6): δ -140.9 (m, 4F).

The spectral data matched with those reported in the literature.[7]

HDF of pentafluoropyridine (**1b**)

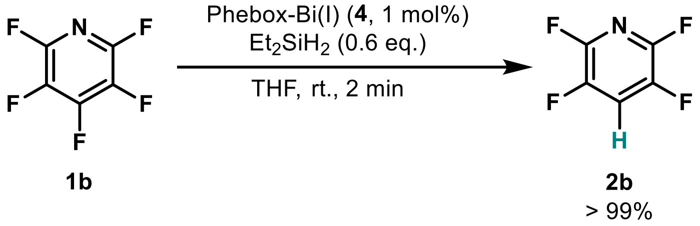

Following the general procedure, the reaction was performed with pentafluoropyridine (**1b**, 42.3 mg, 0.25 mmol), Et_2SiH_2 (13.2 mg, 0.15 mmol, 0.6 equiv.), 4-fluorotoluene (27.5 mg, 0.25 mmol, 1.0 equiv.) and Phebox-Bi(I) (**4**, 1.2 mg, 2.5 µmol, 1 mol%) at room temperature within 2 min. Quantitative ^{19}F NMR revealed that 2,3,5,6-tetrafluoropyridine (**2b**) was formed in > 99% yield.

2,3,5,6-Tetrafluoropyridine (**2b**)

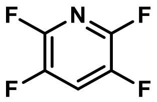

^{19}F NMR (470 MHz, in THF with a sealed glass capillary containing C_6D_6): δ -93.4 – -93.7 (m, 2F, *o*-F), -141.6 – -141.8 (m, 2F, *m*-F).

HRMS (GC-CI): calc'd for $C_5H_2N_1F_4^+$ $[M+H]^+$ 152.011788; found 152.012030.

The spectral data matched with those reported in the literature.[7-8]

HDF of octafluorotoluene (**1c**)

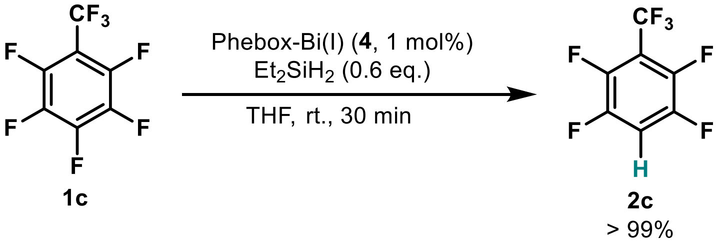

Following the general procedure, the reaction was performed with octafluorotoluene (**1c**, 59.0 mg, 0.25 mmol), Et_2SiH_2 (13.2 mg, 0.15 mmol, 0.6 equiv.), 4-fluorotoluene (27.5 mg, 0.25 mmol, 1.0 equiv.) and Phebox-Bi(I) (**4**, 1.2 mg, 2.5 µmol, 1 mol%) at room temperature within 30 min. Quantitative ^{19}F NMR revealed that 1,2,4,5-tetrafluoro-3-trifluoromethylbenzene (**2c**) was formed in > 99% yield.

1,2,4,5-Tetrafluoro-3-(trifluoromethyl)benzene (**2c**)

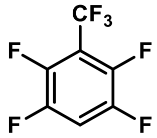

^{19}F NMR (470 MHz, in THF with a sealed glass capillary containing C_6D_6): δ -57.0 – -58.1 (m, 3F), -138.0 – -138.8 (m, 2F), -142.3 – -143.2 (m, 2F).

HRMS (EI): calc'd for $C_7HF_7^+$ [M]$^+$ 217.996099; found 217.996120.

The spectral data matched with those reported in the literature.[7-8]

HDF of methyl 2,3,4,5,6-pentafluorobenzoate (**1d**)

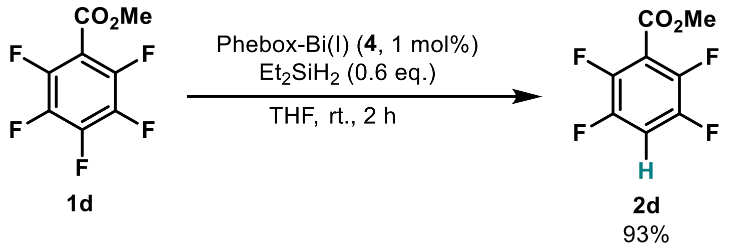

Following the general procedure, the reaction was performed with methyl 2,3,4,5,6-pentafluorobenzoate (**1d**, 56.5 mg, 0.25 mmol), Et_2SiH_2 (13.2 mg, 0.15 mmol, 0.6 equiv.), 4-fluorotoluene (27.5 mg, 0.25 mmol, 1.0 equiv.) and Phebox-Bi(I) (**4**, 1.2 mg, 2.5 μmol, 1 mol%) at room temperature. Quantitative ^{19}F NMR revealed that methyl 2,3,5,6-tetrafluorobenzoate (**2d**) was formed in 64% (93%) yield after 30 min (2 h).

Methyl 2,3,5,6-tetrafluorobenzoate (**2d**)

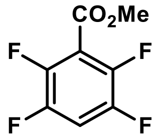

^{19}F NMR (470 MHz, in THF with a sealed glass capillary containing C_6D_6): δ -139.1 – -140.0 (m, 2F), -141.3 – -142.2 (m, 2F).

HRMS (EI): calc'd for $C_8H_4O_2F_4^+$ [M]$^+$ 208.014194; found 208.014190.

The spectral data matched with those reported in the literature.[7b, c, 8]

HDF of 2,3,4,5,6-pentafluorocyanobenzene (**1e**)

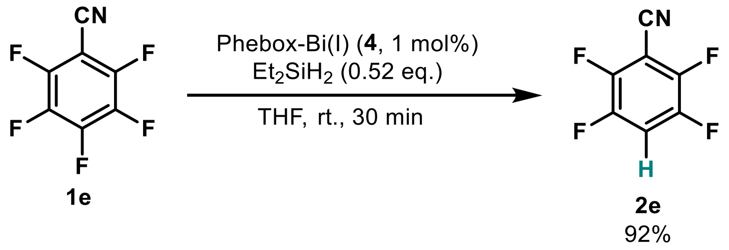

Following the general procedure, the reaction was performed with 2,3,4,5,6-pentafluorocyanobenzene (**1e**, 48.3 mg, 0.25 mmol), Et_2SiH_2 (11.5 mg, 0.13 mmol, 0.52 equiv.), 4-fluorotoluene (27.5 mg, 0.25 mmol, 1.0 equiv.) and Phebox-Bi(I) (**4**, 1.2 mg, 2.5 µmol, 1 mol%) at room temperature within 30 min. Quantitative ^{19}F NMR revealed that 2,3,5,6-tetrafluorocyanobenzene (**2e**) was formed in 92% yield.

2,3,5,6-Tetrafluorobenzonitrile (**2e**)

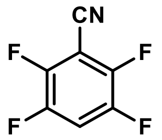

^{19}F NMR (470 MHz, in THF with a sealed glass capillary containing C_6D_6): δ -135.1 – -135.8 (m, 2F), -137.6 – -138.3 (m, 2F).

HRMS (EI): calc'd for $C_7HNF_4^+$ $[M]^+$ 175.003963; found 175.004210.

The spectral data matched with those reported in the literature.[7-8]

HDF of 2,4,5,6-tetrafluoroisophthalonitrile (**1f**)

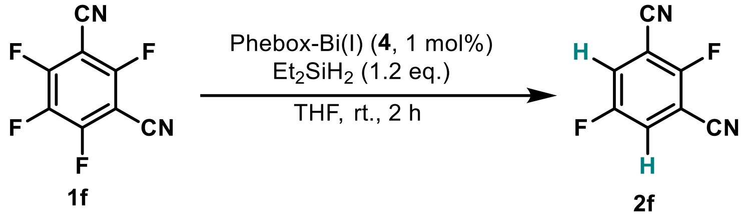

Following the general procedure, the reaction was performed with 2,4,5,6-tetrafluoroisophthalonitrile (**1f**, 50.0 mg, 0.25 mmol), Et_2SiH_2 (26.5 mg, 0.30 mmol, 1.2 equiv.) and Phebox-Bi(I) (**4**, 1.2 mg, 2.5 µmol, 1 mol%) at room temperature for 2 h. 2,5-difluoroisophthalonitrile (**2f**, 36.8 mg, 90%) was obtained as a white crystalline solid after purification by column chromatography on silica gel (*n*-pentane/EtOAc = 20:1 to 8:1, *v*/*v*).

2,5-Difluoroisophthalonitrile (**2f**)

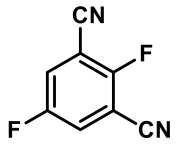

^{1}H NMR (400 MHz, $CDCl_3$): δ 7.63 (dd, J = 6.8, 4.8 Hz, 2H).

^{13}C NMR (101 MHz, $CDCl_3$): δ 161.8 – 156.1 (m), 125.1 (d, J = 26.6 Hz), 111.0 (d, J = 2.5 Hz), 104.7 (dd, J = 16.8, 9.8 Hz).

^{19}F NMR (282 MHz, $CDCl_3$): δ -105.4 (d, J = 16.5 Hz, 1F), -111.4 (d, J = 16.5 Hz, 1F).

M.p.: 108.5 – 110.6 °C.

HRMS (EI): calc'd for $C_8H_2N_2F_2^+$ $[M]^+$ 164.018055; found 164.018210.

HDF of decafluorobiphenyl (**1g**)

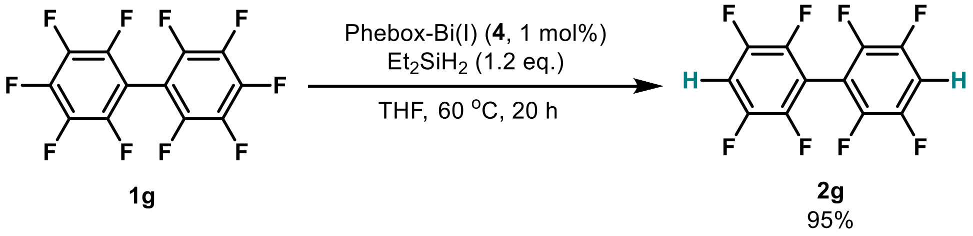

Following the general procedure, the reaction was performed with decafluorobiphenyl (**1g**, 83.5 mg, 0.25 mmol), Et_2SiH_2 (26.5 mg, 0.30 mmol, 1.2 equiv.) and Phebox-Bi(I) (**4**, 1.2 mg, 2.5 µmol, 1 mol%) at 60 °C for 20 h. 2,2',3,3',5,5',6,6'-Octafluoro-1,1'-biphenyl (**2g**, 71.0 mg, 95%) was obtained as a white crystalline solid after purification by column chromatography on silica gel with *n*-pentane as eluent.

2,2',3,3',5,5',6,6'-Octafluoro-1,1'-biphenyl (**2g**)

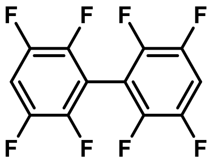

^{1}H NMR (400 MHz, $CDCl_3$): δ 7.57 – 6.93 (m, 2H).

^{13}C NMR (151 MHz, $CDCl_3$): δ 147.3 – 145.2 (m, 2C), 145.1 – 143.2 (m, 2C), 108.3 – 107.8 (m, 4C).

^{13}C{^{19}F} NMR (151 MHz, $CDCl_3$): δ 146.2 (d, J = 7.1 Hz), 144.2 (d, J = 9.3 Hz), 108.1 (d, J = 1.4 Hz), 108.0 (d, J = 168.7 Hz).

^{19}F NMR (565 MHz, $CDCl_3$): δ -135.9 – -136.0 (m, 2F), -136.4 – -136.6 (m, 2F).

M.p.: 83.0 – 84.0 °C.

HRMS (EI): calc'd for $C_{12}H_2F_8^+$ $[M]^+$ 298.002328; found 298.002650.

The spectral data matched with those reported in the literature.[7c, 9]

HDF of octafluoronaphthalene (**1h**)

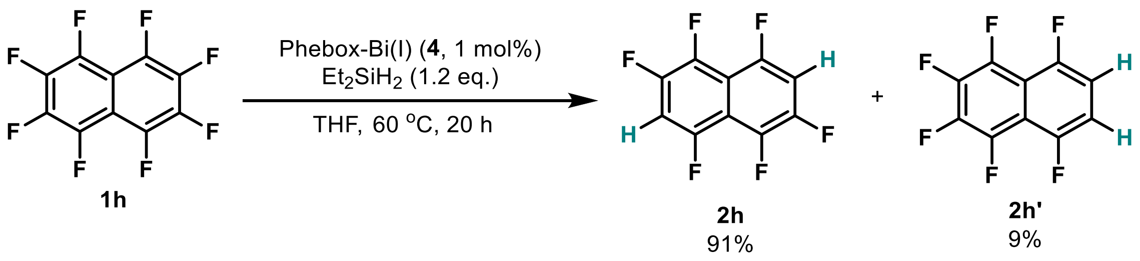

Following the general procedure, the reaction was performed with octafluoronaphthalene (**1h**, 68.0 mg, 0.25 mmol), Et_2SiH_2 (26.5 mg, 0.30 mmol, 1.2 equiv.), 4-fluorotoluene (27.5 mg,

0.25 mmol, 1.0 equiv.) and Phebox-Bi(I) (**4**, 1.2 mg, 2.5 µmol, 1 mol%) at 60 °C for 20 h. Quantitative ^{19}F NMR revealed that 1,2,4,5,6,8-hexafluoronaphthalene (**2h**) and 1,2,3,4,5,8-hexafluoronaphthalene (**2h'**) were formed in 91% and 9% yield, respectively.

1,2,4,5,6,8-Hexafluoronaphthalene (**2h**)

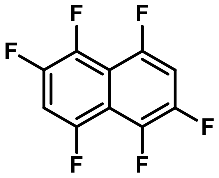

^{19}F NMR (470 MHz, in THF with a sealed glass capillary containing C_6D_6): δ -118.2 – -119.0 (m, 2F), -137.5 – -138.3 (m, 2F), -150.3 – -151.2 (m, 2F).

HRMS (EI): calc'd for $C_{10}H_2F_6^+$ $[M]^+$ 236.005521; found 236.005790.

1,2,3,4,5,8-Hexafluoronaphthalene (**2h'**)

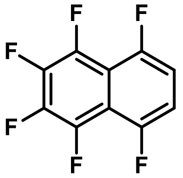

^{19}F NMR (470 MHz, in THF with a sealed glass capillary containing C_6D_6): δ -116.3 – -116.6 (m, 2F), -134.8 – -135.2 (m, 2F), -152.2 – -152.4 (m, 2F).

The spectral data matched with those reported in the literature.[7c]

HDF of (pentafluorophenyl)diphenylphosphane (**1i**)

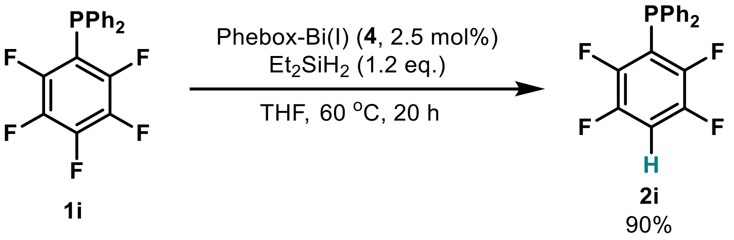

Following the general procedure, the reaction was performed with (pentafluorophenyl)diphenylphosphane (**1i**, 88.1 mg, 0.25 mmol), Et_2SiH_2 (26.5 mg, 0.30 mmol, 1.2 equiv.) and Phebox-Bi(I) (**4**, 3.0 mg, 6.25 µmol, 2.5 mol%) at 60 °C for 20 h. Diphenyl(2,3,5,6-tetrafluorophenyl)phosphane (**2i**, 75.0 mg, 90%) was obtained as a white solid after purification by column chromatography on silica gel (*n*-pentane/EtOAc = 20:1, *v*/*v*).

Diphenyl(2,3,5,6-tetrafluorophenyl)phosphane (**2i**)

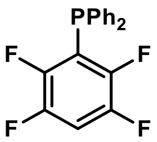

^{1}H NMR (400 MHz, CDCl$_3$): δ 7.49 – 7.41 (m, 4H), 7.41 – 7.34 (m, 6H), 7.12 (tt, J = 9.4, 7.3 Hz, 1H).

^{13}C NMR (101 MHz, CDCl$_3$): δ 148.0 (dm, J = 247.5 Hz), 146.2 (dm, J = 250.3 Hz), 133.7 (dt, J = 10.5, 2.8 Hz), 133.2 (d, J = 21.1 Hz), 129.4, 128.8 (d, J = 7.0 Hz), 117.33 (dt, J = 39.7, 20.6 Hz), 108.2 (tt, J = 22.6, 1.5 Hz).

^{31}P NMR (162 MHz, CDCl$_3$): δ -24.1 (t, J = 38.4 Hz).

^{19}F NMR (282 MHz, CDCl$_3$): δ -128.1 – -128.4 (m), -137.6 – -137.8 (m).

M.p.: 60.5 – 62.7 °C.

HRMS (EI): calc'd for $C_{18}H_{11}F_4P^+$ $[M]^+$ 334.052903; found 334.053510.

The spectral data matched with those reported in the literature.[7b, 10]

HDF of tris(pentafluorophenyl)phosphine (**1j**)

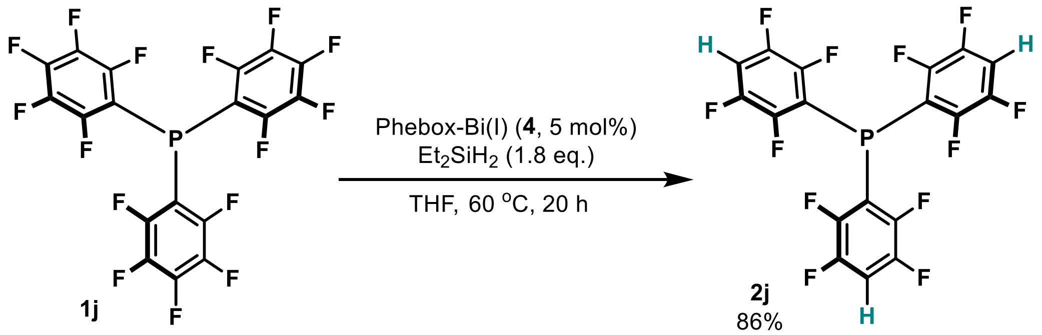

Following the general procedure, the reaction was performed with tris(pentafluorophenyl)phosphine (**1j**, 133.0 mg, 0.25 mmol), Et_2SiH_2 (39.7 mg, 0.45 mmol, 1.8 equiv.) and Phebox-Bi(I) (**4**, 6.0 mg, 12.5 µmol, 5.0 mol%) at 60 °C for 20 h. Tris(2,3,5,6-tetrafluorophenyl)phosphine (**2j**, 103.3 mg, 86%) was obtained as a white crystalline solid after purification by column chromatography on silica gel with *n*-pentane as eluent.

Tris(2,3,5,6-tetrafluorophenyl)phosphane (**2j**)

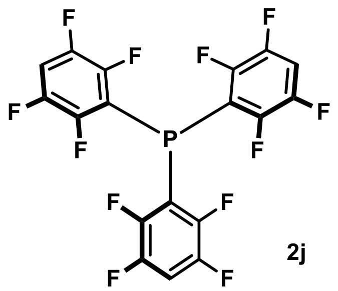

2j

^{1}H NMR (400 MHz, CDCl$_3$): δ 7.25 – 7.13 (m, 3H).

^{13}C NMR (101 MHz, CDCl$_3$): δ 149.1 – 144.4 (m), 111.8 – 110.8 (m), 109.1 (t, J = 22.6 Hz).

^{31}P NMR (162 MHz, CDCl$_3$): δ -71.1 – -72.7 (m).

^{19}F NMR (282 MHz, CDCl$_3$): δ -130.0 – -131.3 (m, 6F), -136.7 – -138.0 (m, 6F).

M.p.: 83.0 – 85.5 °C.

HRMS (CI): calc'd for $C_{18}H_4F_{12}P^+$ $[M+H]^+$ 478.985357; found 478.986110.

The spectral data matched with those reported in the literature.[7b]

HDF of 1,2-Bis-[bis-(pentafluorphenyl)-phosphin]-ethane (dfppe, **1k**)

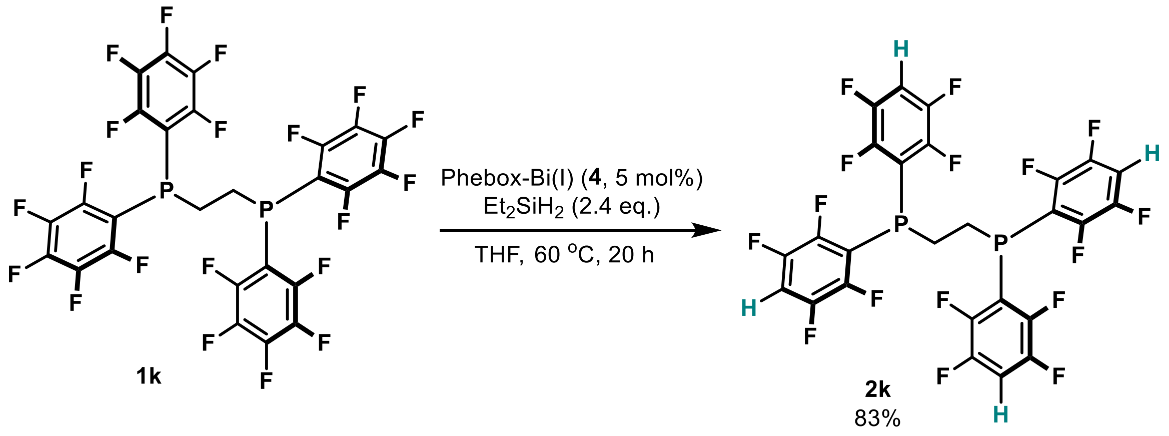

Following the general procedure, the reaction was performed with 1,2-bis-[bis-(pentafluorphenyl)-phosphino]-ethane (dfppe, **1k**, 151.6 mg, 0.20 mmol), Et_2SiH_2 (42.3 mg, 0.48 mmol, 2.4 equiv.) and Phebox-Bi(I) (**4**, 4.8 mg, 10.0 µmol, 5.0 mol%) at 60 °C for 20 h. 1,2-Bis-[bis-(2,3,5,6-trifluorophenyl)phosphino]ethane (**2k**, 114.0 mg, 83%) was obtained as a white crystalline solid after purification by column chromatography on silica gel (*n*-pentane/EtOAc = 1:1, *v*/*v*).

1,2-Bis-[bis-(2,3,5,6-trifluorophenyl)phosphino]ethane (**2k**)

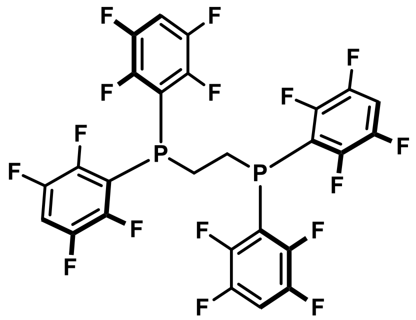

¹H NMR (600 MHz, CDCl3): δ 7.17 – 7.09 (m, 4H), 2.58 (t, *J* = 6.6 Hz, 4H).

¹³C NMR (151 MHz, CDCl3): δ 148.5 – 145.0 (m), 114.8 – 114.0 (m), 108.5 (t, *J* = 22.6 Hz, 4C), 20.5 – 20.1 (m).

¹³C{¹⁹F} NMR (151 MHz, CDCl3): δ 147.4 (dt, *J* = 9.3, 4.6 Hz), 146.0 (d, *J* = 6.8 Hz), 114.8 – 113.9 (m), 108.5 (d, *J* = 168.9 Hz), 20.3 (t, *J* = 135.6 Hz).

³¹P NMR (243 MHz, CDCl3): δ -41.6 – -42.7 (m).

¹⁹F NMR (565 MHz, CDCl3): δ -128.9 – -129.3 (m, 8F), -134.9 – -135.8 (m, 8F).

M.p.: 176.4 – 177.6 °C.

HRMS (ESI): calc'd for $C_{26}H_8NaF_{16}P_2^+$ $[M+Na]^+$ 708.973804; found 708.973910.

The spectral data matched with those reported in the literature.[11]

HDF of pentafluorobenzene (**2a**)

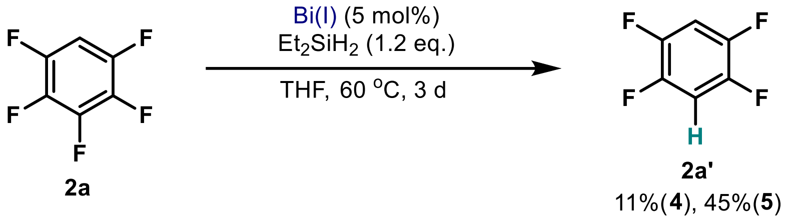

Following the general procedure, the reaction was performed with hexafluorobenzene (42.0 mg, 0.25 mmol), Et_2SiH_2 (26.5 mg, 0.30 mmol, 1.2 equiv.), 4-fluorotoluene (27.5 mg, 0.25 mmol, 1.0 equiv.) and Bi(I) (**4**, 6.0 mg; **5**, 6.4 mg, 12.5 µmol, 5 mol%) at 60 °C for 3 days. Quantitative ^{19}F NMR revealed that 1,2,3,5-tetrafluorobenzene (**2a**) was formed in 11% and 45% yield using **4** and **5** as catalysts, respectively.

HDF of 2,3,4,5,6-pentafluoro-1,1'-biphenyl (**1l**)

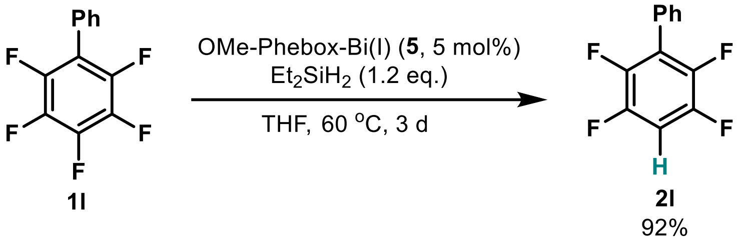

Following the general procedure, the reaction was performed with 2,3,4,5,6-pentafluoro-1,1'-biphenyl (**1l**, 61.0 mg, 0.25 mmol), Et_2SiH_2 (26.5 mg, 0.30 mmol, 1.2 equiv.) and OMe-Phebox-Bi(I) (**5**, 6.4 mg, 12.5 µmol, 5 mol%) at 60 °C for 3 days. 2,3,5,6-Tetrafluoro-1,1'-biphenyl (**2l**, 52.1 mg, 92%) was obtained as a white crystalline solid after purification by column chromatography on silica gel with *n*-pentane as eluent.

2,3,5,6-Tetrafluoro-1,1'-biphenyl (**2l**)

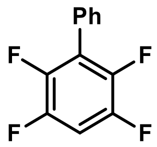

^{1}H NMR (400 MHz, $CDCl_3$): δ 7.55 – 7.41 (m, 5H), 7.13 – 7.00 (m, 1H).

^{13}C NMR (101 MHz, $CDCl_3$): δ 147.9 – 142.4 (m), 130.2 (t, J = 2.3 Hz), 129.3, 128.7, 127.6 (t, J = 2.5 Hz), 121.6 (t, J = 16.6 Hz), 105.0 (t, J = 22.8 Hz).

^{19}F NMR (282 MHz, $CDCl_3$): δ -139.2 (dd, J = 22.3, 12.9 Hz, 2F), -143.9 (dd, J = 22.3, 12.9 Hz, 2F).

M.p.: 106.6 – 108.2 °C.

HRMS (EI): calc'd for $C_{12}H_6F_4^+$ $[M]^+$ 226.040014; found 226.040180.

The spectral data matched with those reported in the literature.[12]

HDF of 2-(perfluorophenyl)pyridine (**1m**)

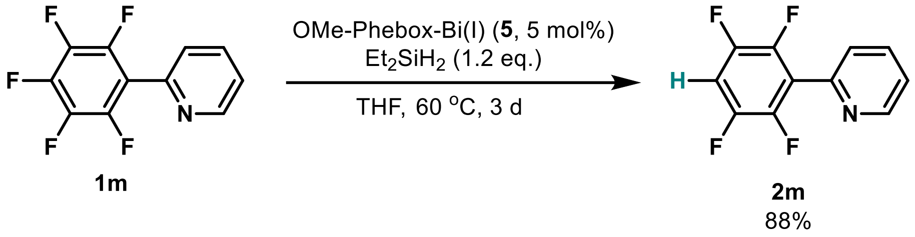

2-(Perfluorophenyl)pyridine (**1m**) was prepared according to a reported method.[13]

Following the general procedure, the reaction was performed with 2-(perfluorophenyl)pyridine (**1m**, 61.3 mg, 0.25 mmol), Et_2SiH_2 (26.5 mg, 0.30 mmol, 1.2 equiv.) and OMe-Phebox-Bi(I) (**5**, 6.4 mg, 12.5 μmol, 5 mol%) at 60 °C for 3 days. 2-(2,3,5,6-Tetrafluorophenyl)pyridine (**2m**, 49.7 mg, 88%) was obtained as a white crystalline solid after purification by column chromatography on silica gel (*n*-pentane/EtOAc = 20:1, *v*/*v*).

2-(Perfluorophenyl)pyridine (**1m**)

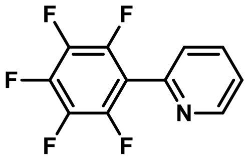

^{1}H NMR (400 MHz, CDCl$_3$): δ 8.77 (ddd, *J* = 4.9, 1.8, 1.0 Hz, 1H), 7.84 (td, *J* = 7.7, 1.8 Hz, 1H), 7.52 – 7.44 (m, 1H), 7.39 (ddd, *J* = 7.6, 4.8, 1.1 Hz, 1H).

^{13}C NMR (101 MHz, CDCl$_3$): δ 150.4, 147.0, 144.8 (dm, *J* = 250.5 Hz), 141.4 (dm, *J* = 255.0 Hz), 138.0 (dm, *J* = 252.7 Hz), 136.9, 126.1, 124.0, 116.0 – 115.0 (m).

^{19}F NMR (282 MHz, CDCl$_3$): δ -143.1 – -143.4 (m, 2F), -153.8 (tt, *J* = 20.9, 2.2 Hz, 1F), -161.7 – -162.1 (m, 2F).

M.p.: 62.7 – 64.5 °C.

HRMS (CI): calc'd for $C_{11}H_5NF_5^+$ $[M+H]^+$ 246.033666; found 246.033360.

The spectral data matched with those reported in the literature.[13a, 14]

2-(2,3,5,6-Tetrafluorophenyl)pyridine (**2m**)

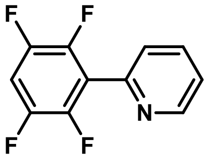

^{1}H NMR (400 MHz, CDCl$_3$): δ 8.78 (ddd, *J* = 4.9, 1.9, 1.0 Hz, 1H), 7.84 (td, *J* = 7.7, 1.8 Hz, 1H), 7.53 – 7.47 (m, 1H), 7.38 (ddd, *J* = 7.7, 4.9, 1.2 Hz, 1H), 7.19 – 7.07 (m, 1H).

^{13}C NMR (101 MHz, $CDCl_3$): δ 150.2, 147.8 (t, J = 3.3 Hz), 146.3 (dddd, , J = 248.5, 14.6, 10.0, 4.0 Hz), 144.3 (ddt, J = 249.4, 14.1, 4.0 Hz), 136.8, 126.0 (t, J = 2.3 Hz), 123.9, 120.9 (t, J = 16.1 Hz), 106.2 (t, J = 22.6 Hz).

^{19}F NMR (282 MHz, $CDCl_3$): δ -138.8 (dd, J = 22.0, 12.7 Hz, 2F), -143.9 (dd, J = 22.0, 13.2 Hz, 2F).

M.p.: 74.1 – 75.3 °C.

HRMS (EI): calc'd for $C_{11}H_5NF_4^+$ $[M]^+$ 227.035263; found 227.035170.

The spectral data matched with those reported in the literature.[13b]

HDF of 2,4,6-trifluorobenzonitrile (**1n**)

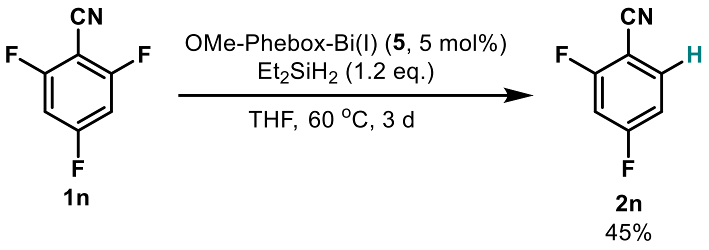

Following the general procedure, the reaction was performed with 2,4,6-trifluorobenzonitrile (**1n**, 39.3 mg, 0.25 mmol), Et_2SiH_2 (26.5 mg, 0.30 mmol, 1.2 equiv.), 4-fluorotoluene (27.5 mg, 0.25 mmol, 1.0 equiv.) and OMe-Phebox-Bi(I) (**5**, 6.4 mg, 12.5 µmol, 5 mol%) at 60 °C for 3 days. Quantitative ^{19}F NMR revealed that 2,4-difluorobenzonitrile (**2n**) was formed in 45% yield.

2,4-Difluorobenzonitrile (**2n**)

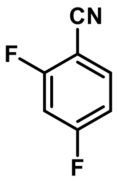

^{19}F NMR (470 MHz, in THF with a sealed glass capillary containing C_6D_6): δ -100.3 (d, J = 11.4 Hz), -104.5 (d, J = 11.4 Hz).

HRMS (EI): calc'd for $C_7H_3NF_2^+$ $[M]^+$ 139.022806; found 139.022940.

^{19}F NMR spectra matched with pure **2n** purchased from Sigma Aldrich.

4. Mechanistic Studies of Bi(I)-catalyzed HDF Reactions

4.1. Mechanistic Proposal: Bi(I)/Bi(III) Redox Catalytic Cycle

A Bi(I)/Bi(III) catalytic redox cycle is proposed for the HDF reactions (Figure S1), comprising oxidative addition of Bi(I) to the C(sp^2)–F bonds of polyfluoroarenes, H/F ligand exchange between Phebox-Bi(III)(polyfluoroaryl) fluoride and hydrosilanes, and C-H reductive elimination from Phebox-Bi(III)(polyfluoroaryl) hydride.

In light of the demonstrated high reactivity, pentafluoropyridine (**1b**) was chosen as the model compound to probe the mechanism of the Bi(I)-catalyzed HDF reactions. Mechanistic studies directly using Phebox-Bi(III)(4-tetrafluoropyridyl) fluoride (**8a**) were not feasible, since **8a** is unstable and further complicated by complex equilibriums (*vide infra*). Therefore, Phebox-Bi(III)(4-tetrafluoropyridyl) triflate (**8b**) was used in the stoichiometric studies and a synthetic HDF cycle was realized (Figure S1). Stoichiometric LAH reduction and kinetic studies using Et_2SiH_2/Et_2SiD_2 confirm the bismuth hydride nature of **9**, its capacity of C-H reductive elimination and involvement in the catalytic reaction.

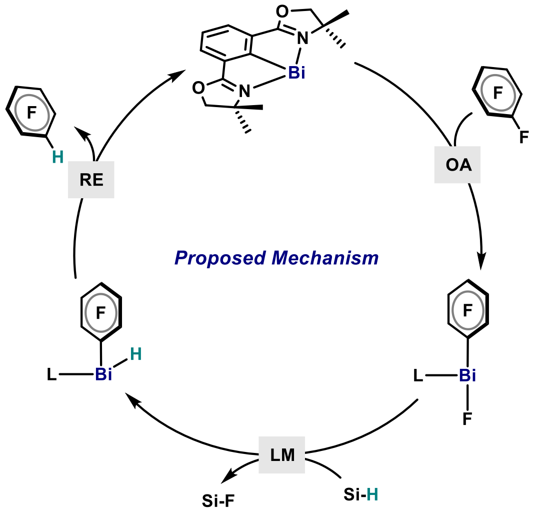

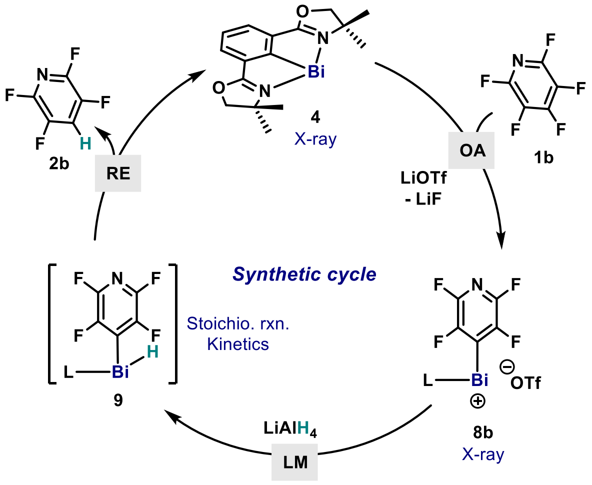

Figure S1. Proposed mechanism and model synthetic cycle for mechanistic studies

4.2. Oxidative Addition of Phebox-Bi(I) (**4**) with Pentafluoropyridine (**1b**)

4.2.1. Procedure of OA and Stoichiometric HDF Reactivity

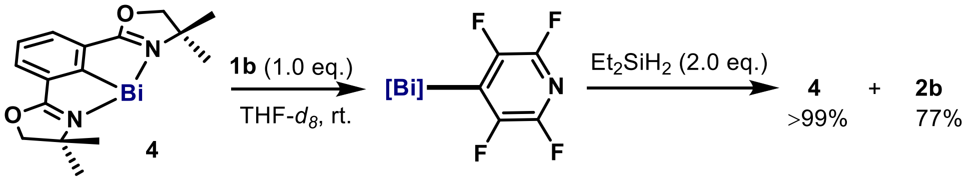

Procedure: In a glovebox, Phebox-Bi(I) (**4**, 10.0 mg, 20.8 μmol) and 0.4 mL anhydrous THF-d_8 were added to a flame-dried vial. Upon rapid stirring, a stock solution of pentafluoropyridine (**1b**, 0.208 M, 100 μL, 1.0 equiv.) in THF-d_8 was added dropwise. The mixture was allowed to react for 5 min and the resulting pale green solution was transferred into a J-Young NMR tube and analyzed by NMR spectroscopy.

Based on the comprehensive NMR data (*vide infra*), it was concluded that Phebox-Bi(4-tetrafluoropyridyl) fluoride (**8a**) was formed via OA and is involved in equilibriums with Phebox-Bi(I) (**4**), Phebox-Bi(4-tetrafluoropyridyl)$_2$ (**S4**) and Phebox-BiF$_2$ (**S5**) (Figure S2). The detailed mechanism of the equilibriums remains elusive, which might involve reversible OA and disproportionation processes.

Stoichiometric HDF: Treatment of the mixture with a stock solution of Et_2SiH_2 (0.208 M, 200 μL, 2.0 equiv.) in THF-d_8 at 25 °C leads to immediate regeneration of Phebox-Bi(I) (**4**, >99%) and the formation of HDF product **2b** (77%), *demonstrating the stoichiometric HDF reactivity of the mixture*.

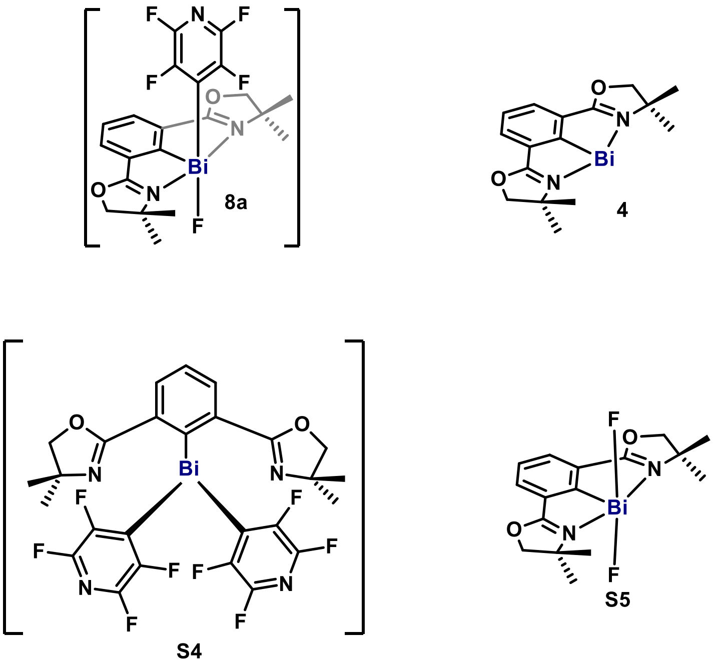

Figure S2. The compounds that are in equilibriums in the OA

4.2.2. NMR Data of **8a**, **S4** and **S5**

The 1D and 2D NMR spectra data of the reaction mixture allowed $^{1}H/^{13}C/^{19}F$ signals of **8a** and **S4** to be largely assigned (Figure S3).

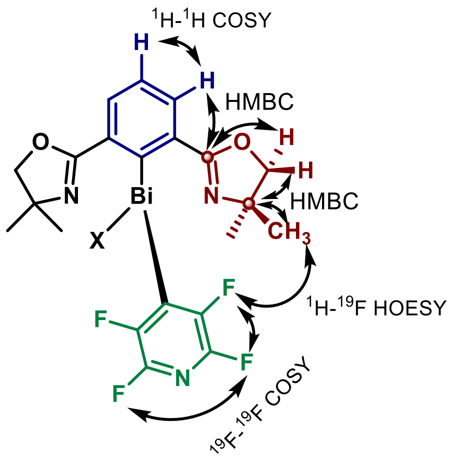

Figure S3. The NMR assignment of **8a** and **S4**

Phebox-Bi(4-tetrafluoropyridyl) fluoride (**8a**)

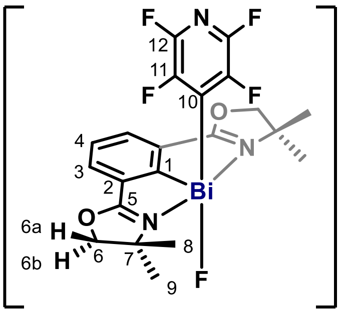

NMR data of **8a** at 25 °C:

^{1}H NMR (600 MHz, THF-*d*8, 25 °C): δ 8.09 (d, br., *J* = 7.7 Hz, 2H, H-3), 7.62 (t, br., *J* = 7.7 Hz, 1H, H-4), 4.21 (s, br., 4H, H-6a/b), 1.36 (s, br., 6H, H-9), 1.20 (s, br., 6H, H-8).

^{19}F NMR (565 MHz, THF-*d*8, 25 °C): δ -95.8 – -96.2 (m, 2F, F-12), -125.4 – -125.8 (m, 2F, F-11).

NMR data of **8a** at -40 °C:

^{1}H NMR (600 MHz, THF-*d*8, -40 °C): δ 8.02 (d, *J* = 7.6 Hz, 2H, H-3), 7.64 (t, *J* = 7.6 Hz, 1H, H-4), 4.25 (d, *J* = 8.2 Hz, 2H, H-6a), 4.22 (d, *J* = 8.2 Hz, 2H, H-6b), 1.38 (s, 6H, H-9), 1.14 (s, 6H, H-8).

^{13}C NMR (151 MHz, THF-*d*8, -40 °C): δ 198.5 (br., C-1), 174.1 (br., C-5), 136.4 (C-2), 133.4 (C-3), 128.9 (C-4), 81.6 (C-6), 68.6 (C-7), 28.8 (C-9), 28.2 (C-8).

^{19}F NMR (565 MHz, THF-*d*8, -40 °C): δ -97.1 (br., 2F, F-12), -124.5 (br., 2F, F-11).

C-11 and C-12 could not be assigned. The signal of fluoride could not be assigned unambiguously due to broadening or exchange (see VT NMR data). C-10s for **8a** and **S4** [δ

182.4 (t, br., J = 56.2 Hz) and 163.0 (t, J = 51.3 Hz)] were found in ^{13}C NMR at -40 °C but could not be assigned to **8a** and **S4** unambiguously.

Phebox-Bi(4-tetrafluoropyridyl)$_2$ (**S4**)

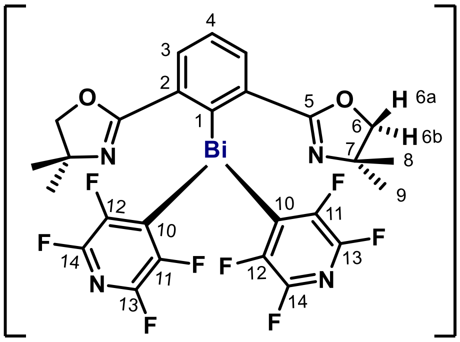

NMR data of **S4** at 25 °C:

^{1}H NMR (600 MHz, THF-d_8, 25 °C): δ 8.19 (d, J = 7.7 Hz, 2H, H-3), 7.69 (t, J = 7.7 Hz, 1H, H-4), 4.05 (s, br., 4H, H-6a/b), 1.03 (s, br., 12H, H-8/9).

^{19}F NMR (565 MHz, THF-d_8, 25 °C): δ -94.9 (br., 4F, F-13/14). The signals of F-11, F-12 and fluoride anions of **8a** and **S5** overlap with each other and are considerably broadened (ca. -119.4 – -124.0 ppm).

NMR data of **S4** at -40 °C:

^{1}H NMR (600 MHz, THF-d_8, -40 °C): δ 8.20 (d, J = 7.6 Hz, 2H, H-3), 7.75 (t, J = 7.6 Hz, 1H, H-4), 4.18 (d, J = 8.4 Hz, 2H, H-6a), 4.01 (d, J = 8.4 Hz, 2H, H-6b), 1.26 (s, 6H, H-9), 0.77 (s, 6H, H-8).

^{13}C NMR (151 MHz, THF-d_8, -40 °C): δ 169.8 (br., C-5), 139.1 (C-2), 132.9 (C-3), 130.2 (C-4), 80.6 (C-6), 69.2 (C-7), 29.3 (C-9), 27.0 (C-8).

^{19}F NMR (565 MHz, THF-d_8, -40 °C): δ -94.2 – -94.6 (m, br., 2F, F-13), -95.4 – -95.9 (m, br., 2F, F-14), -122.1 – -122.6 (m, br., 2F, F-12), -125.6 (t, J = 27.3 Hz, 2F, F-11).

C-11, C-12, C-13 and C-14 could not be assigned. C-1 (C_{ipso}) could not be found in HMBC due to dynamic behavior or residual coupling effects from ^{209}Bi.

MS studies: the attempts to detect any molecular fragment containing 4-tetrafluoropyridyl failed using mass spectroscopic method, due to highly moisture-sensitivity of the Bi-C bonds in **8a** and **S4**. Instead, [Phebox-Bi-OH]$^+$ and [Phebox-Bi-F]$^+$ were found.

Phebox-BiF$_2$ (**S5**)

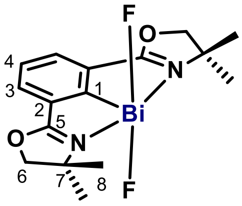

NMR data of **S5** (extracted from NMR spectra of the reaction mixture):

^{1}H NMR (600 MHz, THF-d_8, 25 °C): δ 8.04 (d, *J* = 7.6 Hz, 2H, H-3), 7.53 (t, *J* = 7.6 Hz, 1H, H-4), 4.32 (s, 4H, H-6), 1.42 (s, 12H, H-8).

^{1}H NMR (600 MHz, THF-d_8, -40 °C): δ 7.96 (d, *J* = 7.5 Hz, 2H, H-3), 7.55 (t, *J* = 7.6 Hz, 1H, H-4), 4.30 (s, 4H, H-6), 1.36 (s, 12H, H-8).

^{13}C NMR (151 MHz, THF-d_8, -40 °C): δ 222.4 (C-1), 177.6 (C-5), 134.0 (C-2), 133.6 (C-3), 128.3 (C-4), 82.3 (C-6), 67.9 (C-7), 28.6 (C-8).

The fluoride signal could not be unambiguously assigned likely due to exchange with of other fluorides or broadening due to residual coupling to ^{209}Bi (see VT NMR data).

S5 was independently prepared by the following procedure for comparison with the reaction mixture.

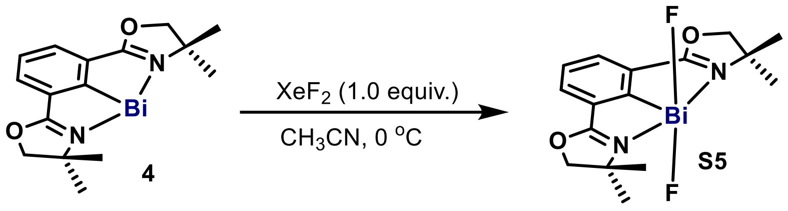

Procedure: In a glovebox, a flame-dried 50 mL Schlenk flask was charged with **4** (100 mg, 208 µmol) and CH_3CN (anhydrous and degassed, 10 mL). The Schlenk flask was taken out of the glovebox. At 0 °C, XeF_2 (38.8 mg, 229 µmol, 1.1 equiv.) was added portionwise under argon and the solution rapidly decolorized. After stirring for 5 min, CH_3CN was removed *in vacuo*. In the glovebox, the crude product was re-dissolved by THF (anhydrous and degassed, 5 mL). *n*-Pentane (anhydrous and degassed, 10 mL) was used to reprecipitate the product and the mother liquor was decanted. After drying under high vacuum, **S5** was obtained as an off-white powder (83 mg, 77%).

S5 is soluble in CH_3CN but has low solubility in THF. **S5** is very moisture-sensitive, and reacts with CH_3CN and THF slowly, leading to Phebox-H and unidentifiable by-products.

The ^{1}H NMR data of **S5** in the reaction mixture and pure Phebox-BiF_2 are slightly different, however, the ^{13}C NMR data match well. The deviation of the ^{1}H NMR chemical shifts could be attributed to fluoride anion exchange with **8a**.

NMR data of pure **S5**:

^{1}H NMR (600 MHz, CD_3CN, 25 °C): δ 8.22 (d, *J* = 7.6 Hz, 2H, H-3), 7.71 (t, *J* = 7.6 Hz, 1H, H-4), 4.51 (s, 4H, H-6), 1.50 (s, 12H, H-8).

^{13}C NMR (151 MHz, CD_3CN, 25 °C): δ 216.8 (C-1), 182.0 (C-5), 134.6 (C-3), 132.2 (C-2), 129.8 (C-4), 83.6 (C-6), 67.9 (C-7), 28.4 (C-8).

^{19}F NMR (565 MHz, CD_3CN, 25 °C): δ -115.2 (br. s).

^{1}H NMR (600 MHz, THF-d_8, 25 °C): δ 8.10 (d, *J* = 7.6 Hz, 2H, H-3), 7.56 (t, *J* = 7.6 Hz, 1H, H-4), 4.37 (s, 4H, H-6), 1.46 (s, 12H, H-8).

^{19}F NMR (282 MHz, THF-d_8, 25 °C): δ -118.1 (br., 2F).

^{1}H NMR (600 MHz, THF-d_8, -40 ºC): δ 8.07 (d, *J* = 7.5 Hz, 2H), 7.59 (t, *J* = 7.6 Hz, 1H), 4.38 (s, 4H), 1.44 (s, 12H).

^{13}C NMR (151 MHz, , THF-d_8, -40 ºC): δ 220.3 (C-1), 177.9 (C-5), 134.0 (C-2), 133.6 (C-3), 128.6 (C-4), 82.4 (C-6), 68.0 (C-7), 28.6 (C-8).

^{19}F NMR (282 MHz, THF-d_8, -40 ºC): δ -114.8 (br., 2F).

M.p.: > 220 ^{o}C (dec.).

HRMS (ESI): calc'd for $C_{16}H_{19}N_2O_2BiF^+$ [M-F]$^+$ 499.12289; found 499.12317.

4.2.3. NMR Analysis of the OA Reaction

^{1}H NMR (THF-d_8, 600 MHz, 25 °C)

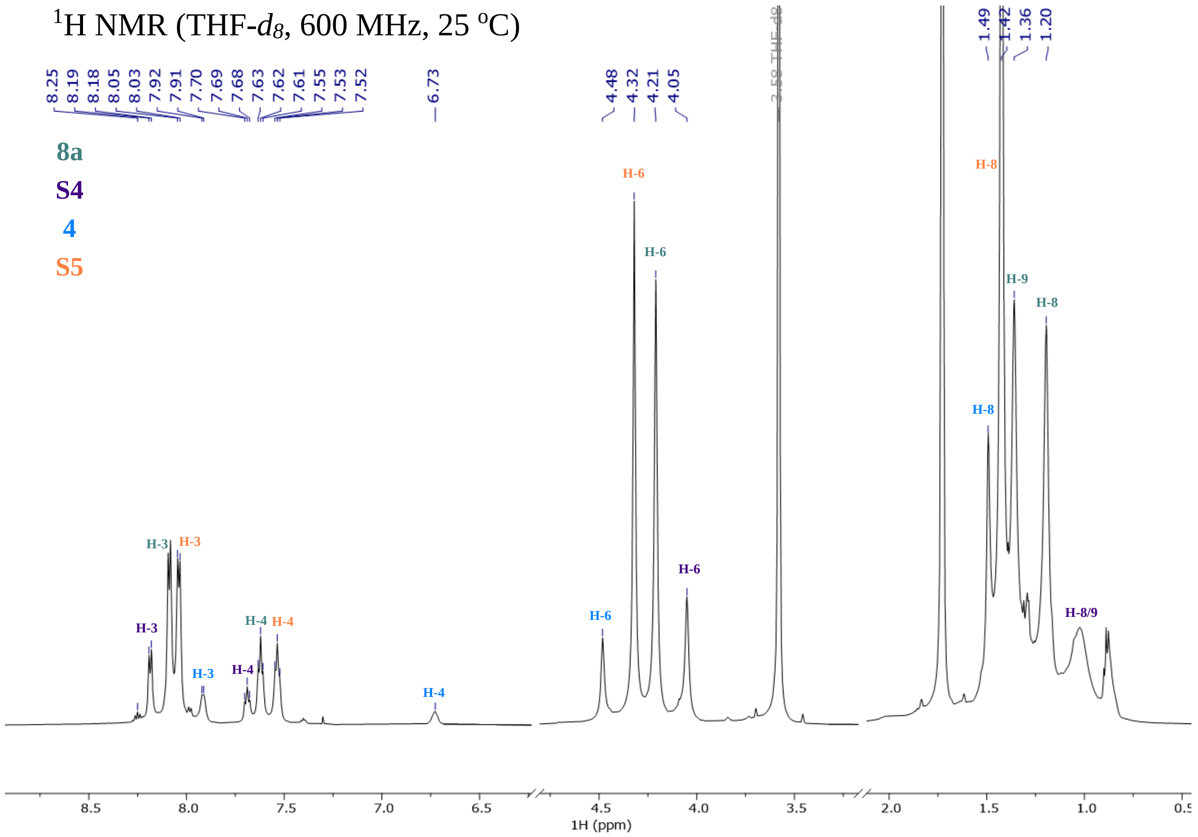

^{1}H NMR at 25 °C reveals that **8a**, **S4** and **S5** were formed in the stoichiometric OA reaction while a small portion of **4** remained unreacted. The noticeable signal broadening of **4** and other species indicates the chemical interconversions within this mixture.

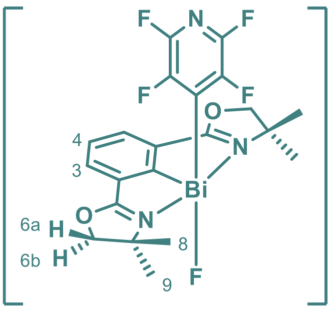

8a

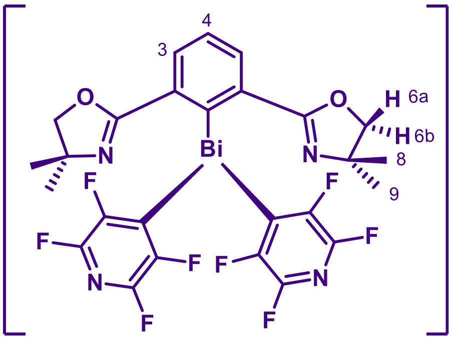

S4

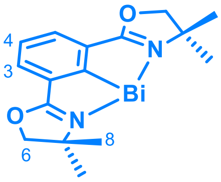

Phebox-Bi(I) (**4**)

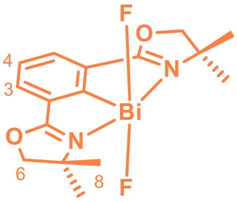

Phebox-BiF$_2$ (**S5**)

ROESY (25 °C)

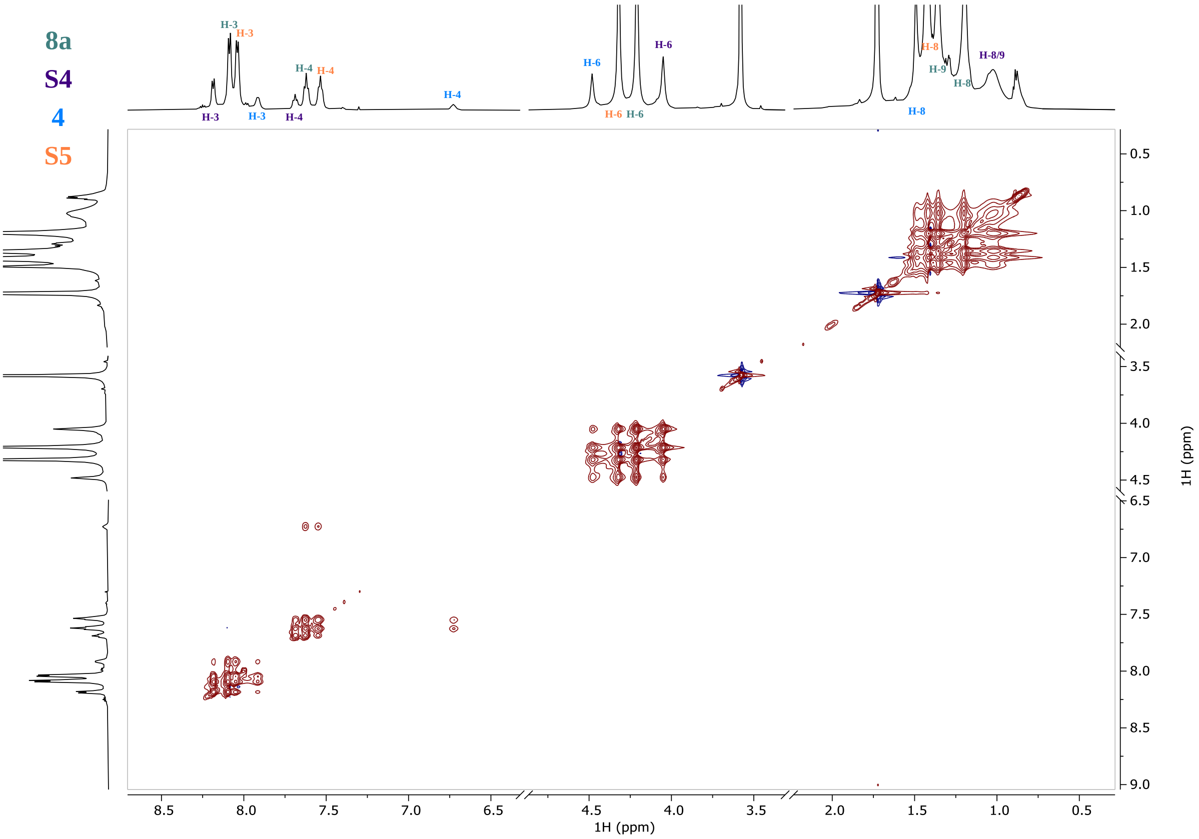

EXSY crosspeaks (red) in the ROESY spectrum at 25 °C show a rapid chemical interconversion between **4**, **8a**, **S4** and **S5**.

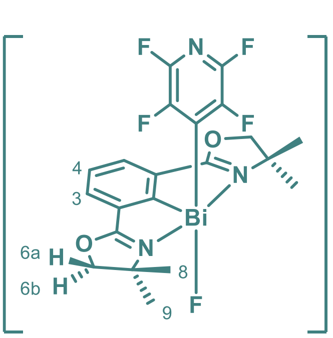

8a

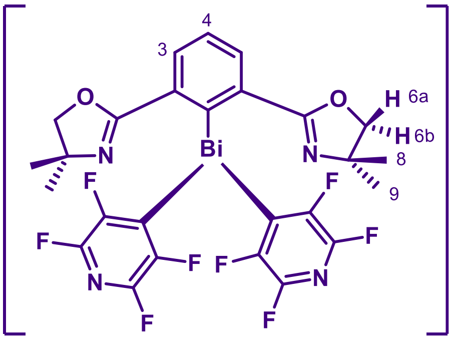

S4

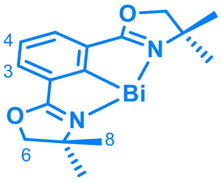

Phebox-Bi(I) (**4**)

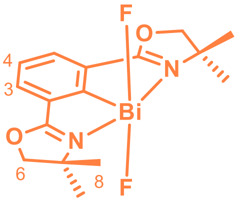

Phebox-BiF$_2$ (**S5**)

^{1}H NMR spectra at different temperatures (25 ^{o}C to -40 ^{o}C)

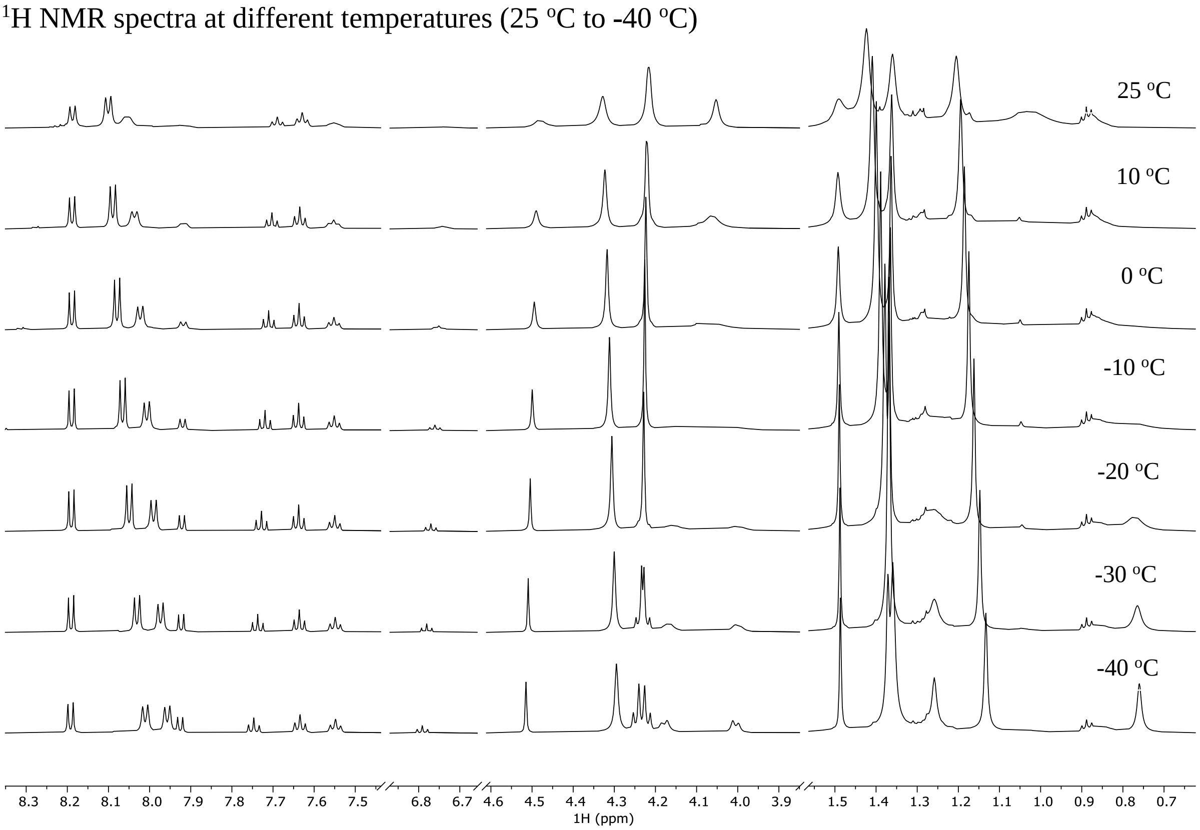

Variable temperature ^{1}H NMR data (25 ^{o}C to -40 ^{o}C) shows the signal sharpening of all species at lower temperatures.

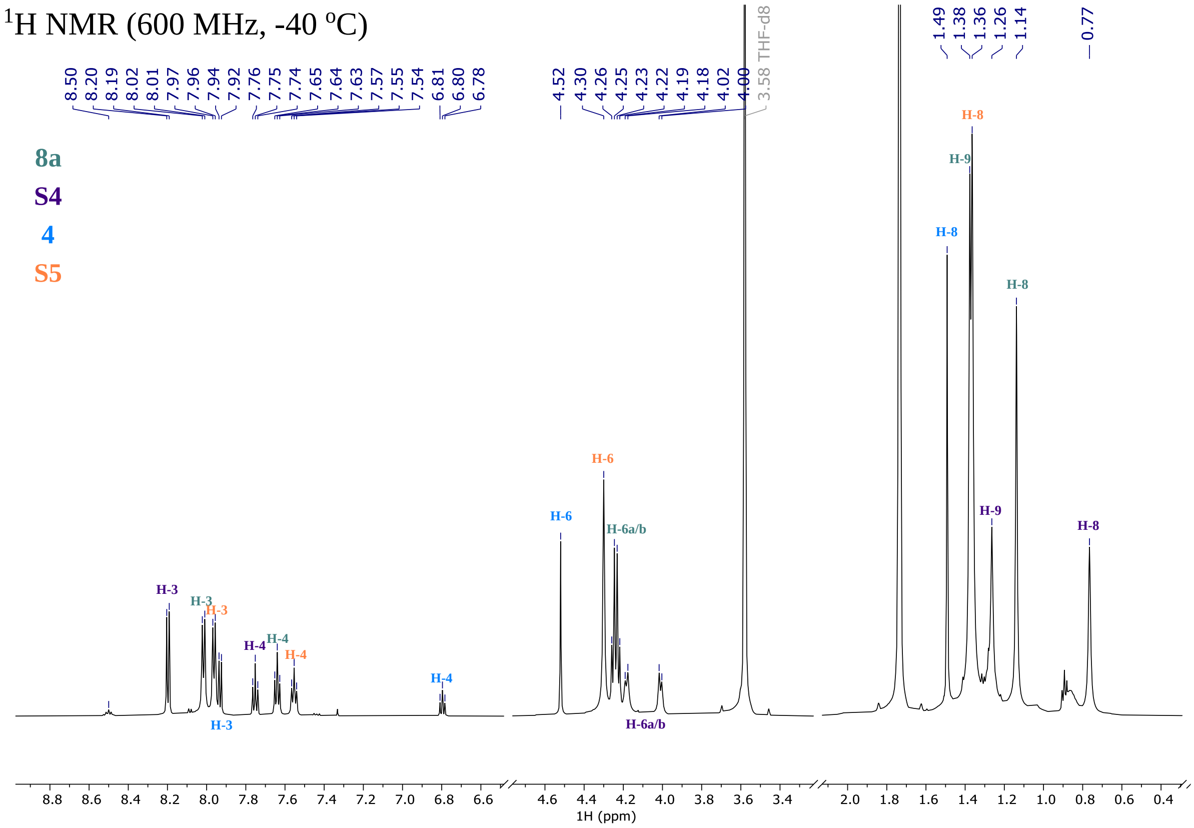

At -40 °C, **8a** and **S4** have asymmetric Phebox backbones, as indicated by the inequivalence of CH_2 and CH_3 groups of the oxazolines.

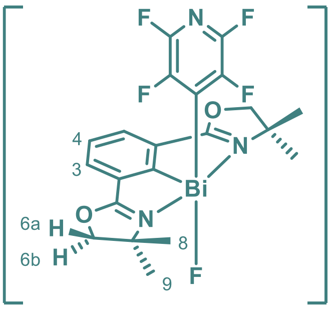

8a

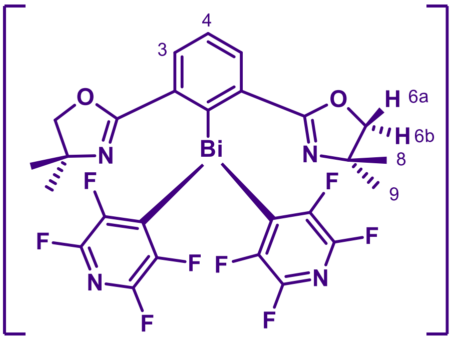

S4

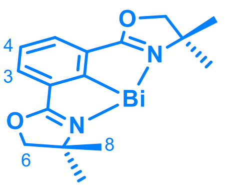

Phebox-Bi(I) (**4**)

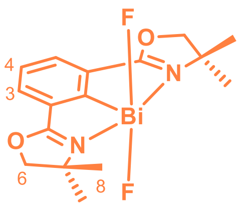

Phebox-BiF$_2$ (**S5**)

ROESY (600 MHz, -40 °C)

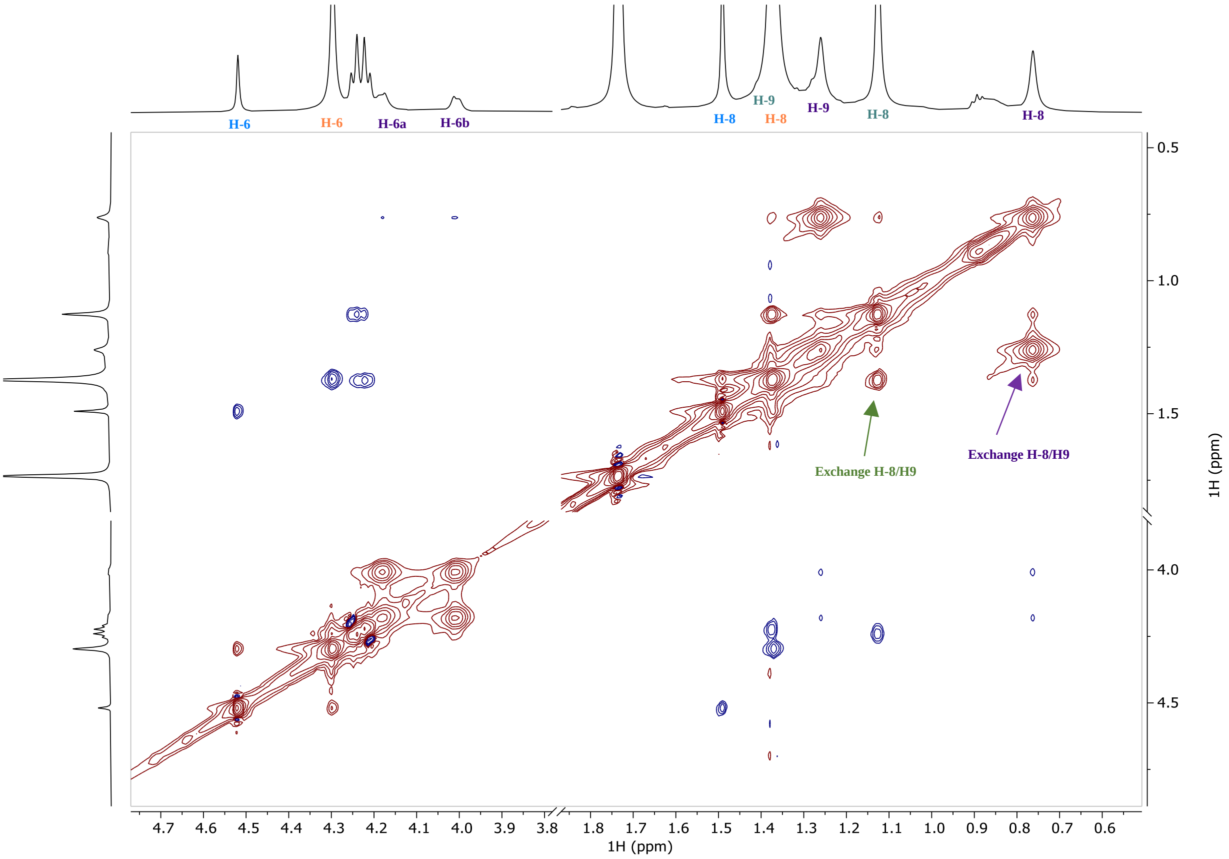

EXSY cross peaks (red) in the ROESY spectrum show a reduced chemical interconversion between the bismuth species at -40 °C, however, **8a** and **S4** are still structurally fluxional, as shown by more intense EXSY correlations between CH_2 and CH_3 groups of the oxazolines.

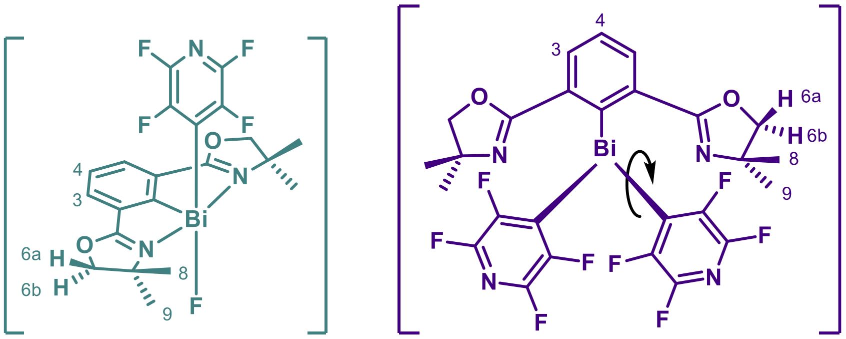

8a

S4

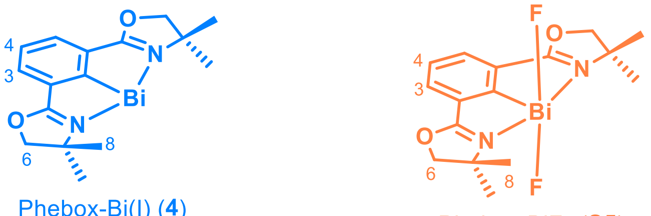

Phebox-Bi(I) (**4**)

Phebox-BiF_2 (**S5**)

^{19}F NMR (THF-d_8, 565 MHz, 25 °C)

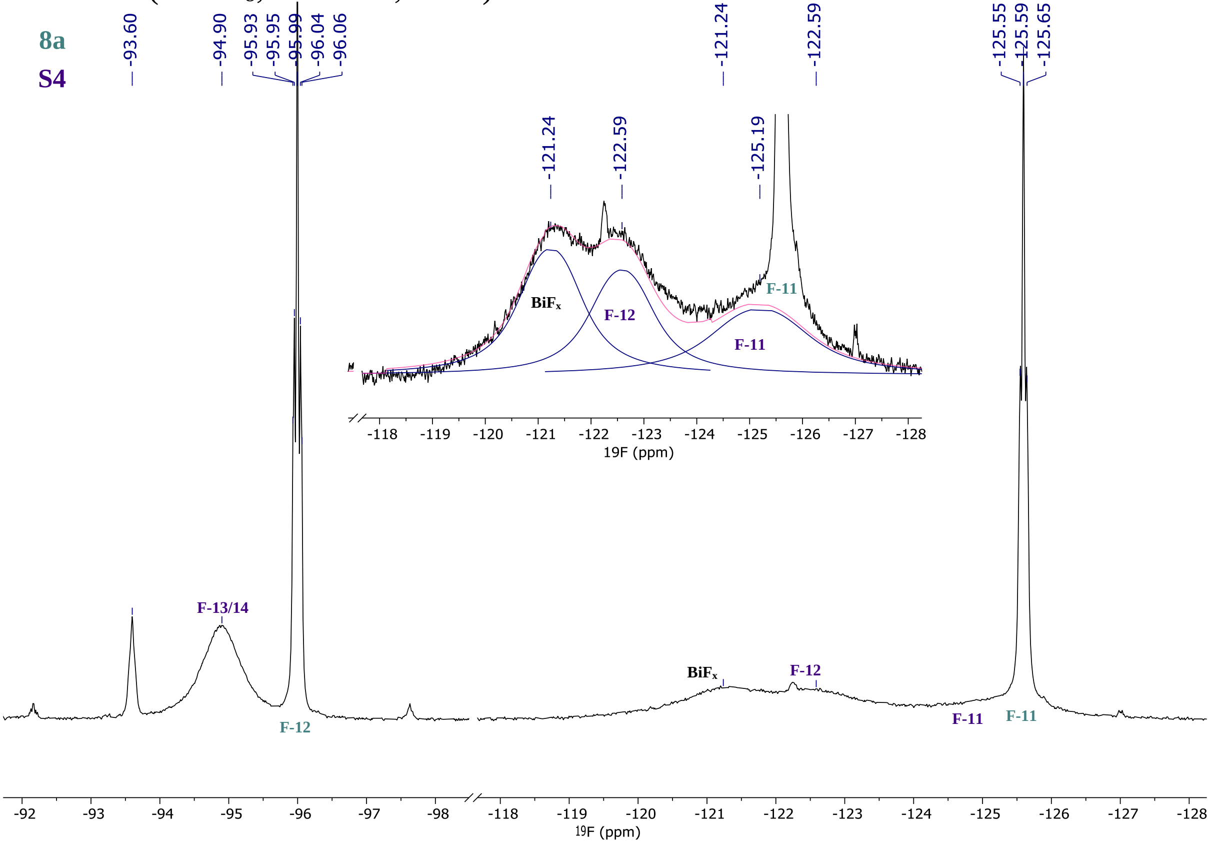

^{19}F NMR at 25 °C shows that **8a** and **S4** contain 4-tetrafluoropyridyl groups. The ^{19}F chemical shifts of *ortho*-fluorines (-120 to -127 ppm) are similar to that of the reported Bi(4-C_5F_4N)$_3$ (-120.7 ppm).[15] **8a** has sharp ^{19}F NMR signals (-96.0 and -125.6 ppm) while the signals (especially the *ortho*-fluorines) of **S4** are considerably broadened, presumably due to restricted rotation of two 4-tetrafluoropyridyls in **S4**.

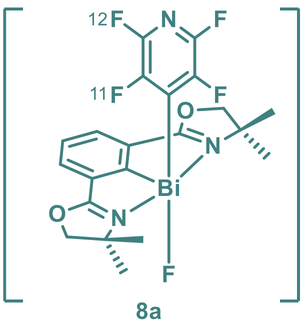

8a

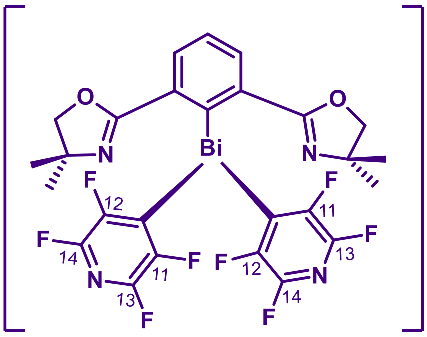

S4

^{19}F NMR spectra at different temperatures (25 °C to -40 °C)

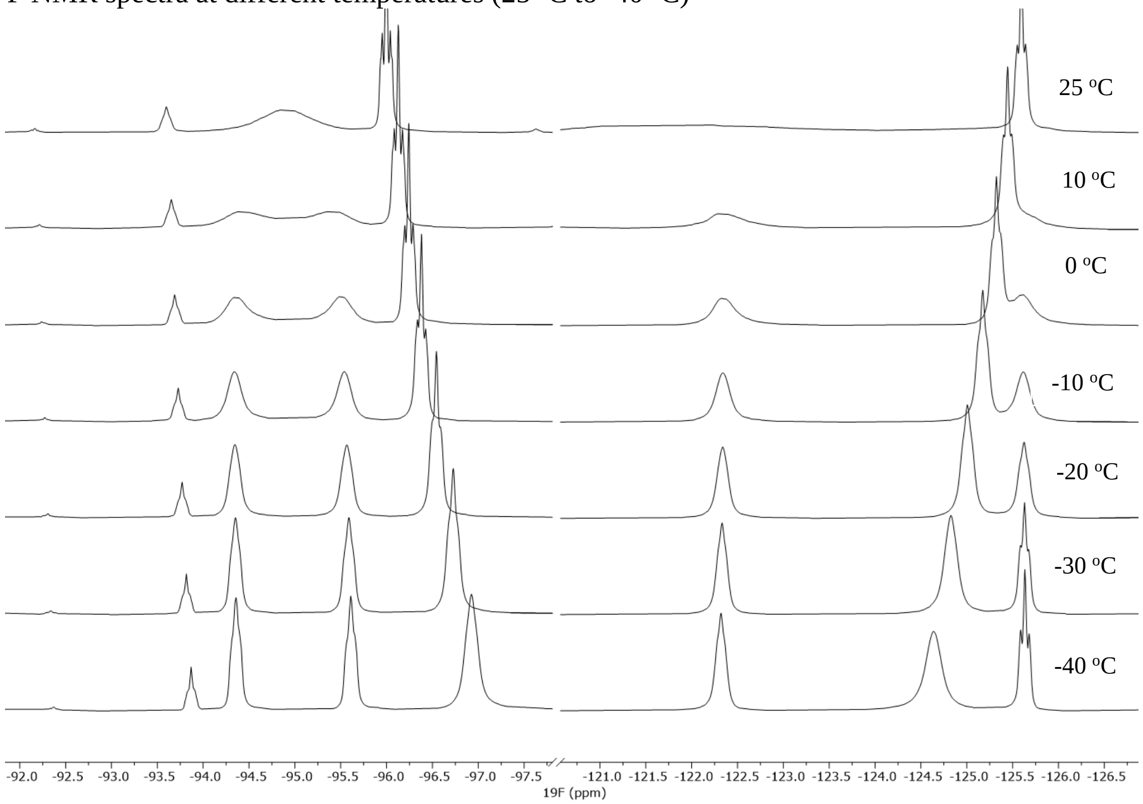

Variable temperature ^{19}F NMR data (25 °C to –40 °C) shows that the ^{19}F signals of **S4** sharpen at lower temperatures and all *ortho*-fluorines and *meta*-fluorines of **S4** become inequivalent.

^{19}F NMR spectra at different temperatures (25 °C to -40 °C)

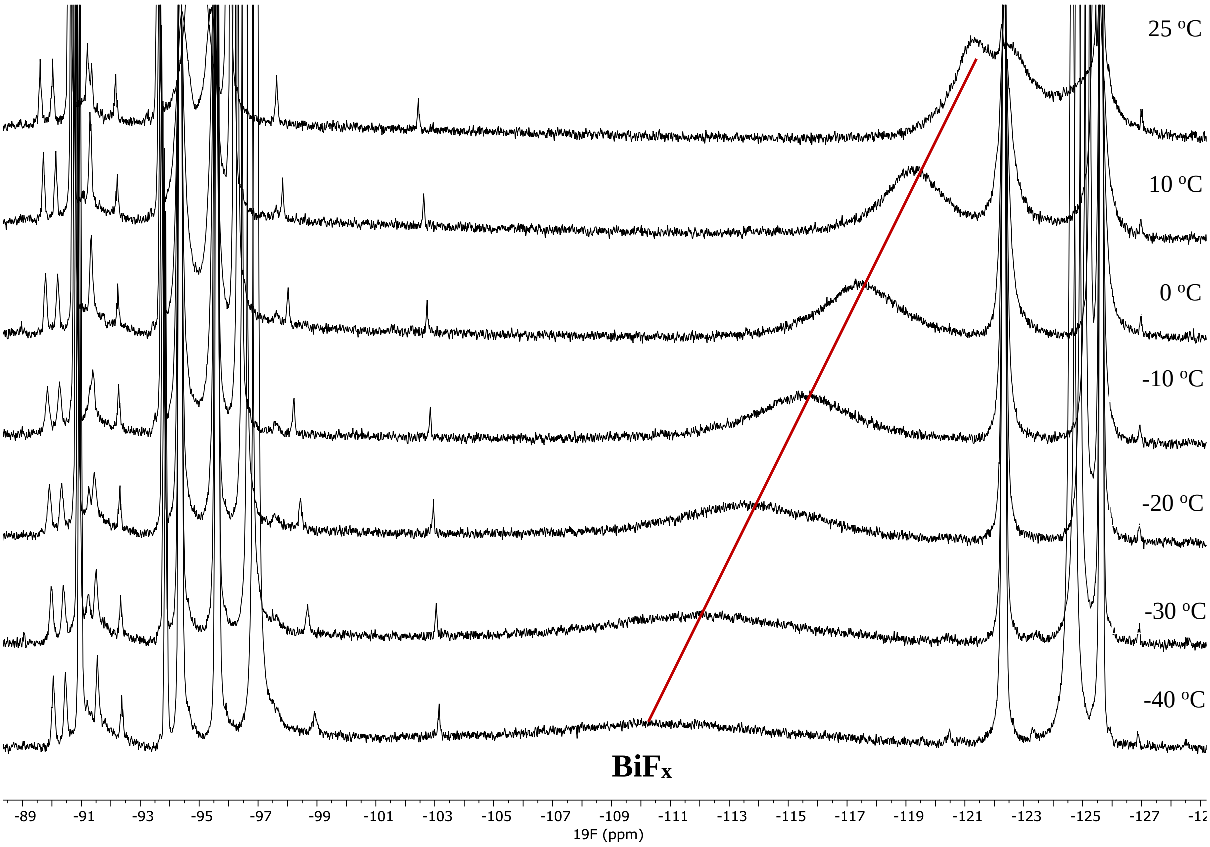

An additional broad component is visible at 25°C. At lower temperatures a strong broadening and rather strong temperature dependence is observed. This signal could be attributed to a fluorine with an increased ionic character and which is in chemical exchange within multiple Bi-species (**8a** and **S5**)

^{19}F NMR (THF-d_8, 565 MHz, -40 °C)

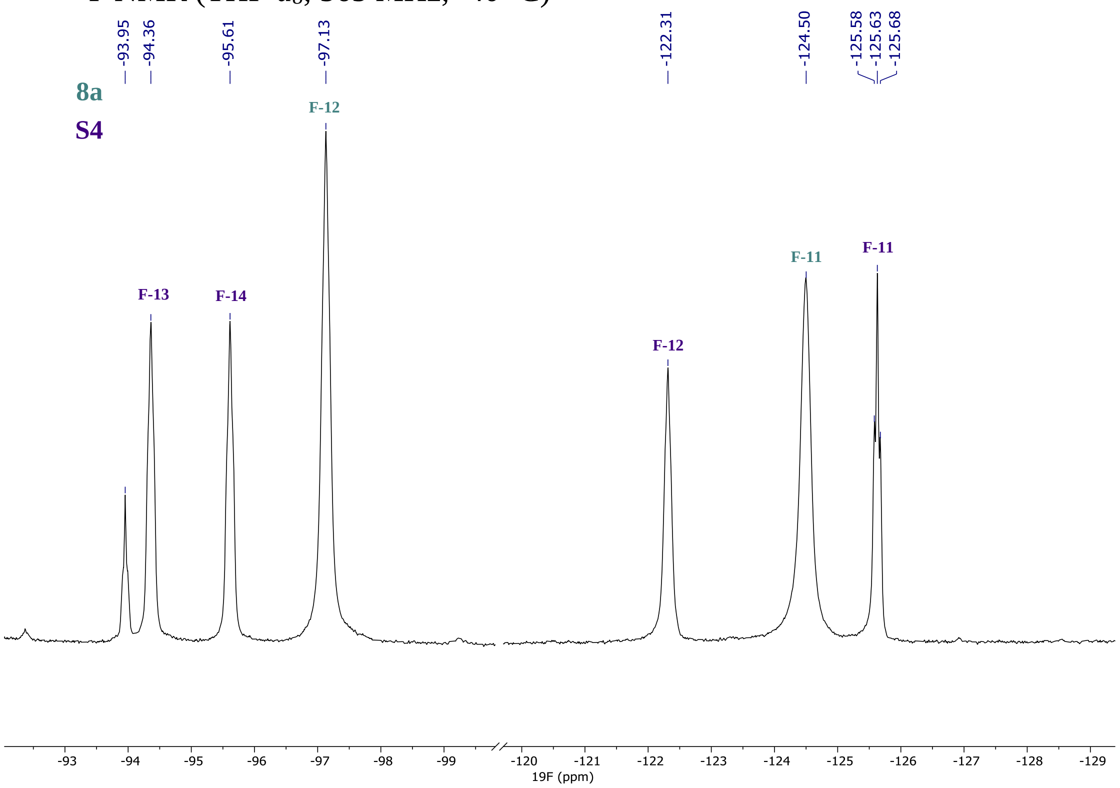

^{19}F NMR at -40 °C shows that the *ortho*-fluorines (F-11 and F-12) and *meta*-fluorines (F-13 and F-14) of **S4** are inequivalent, probably because of the rotation of two 4-tetrafluoropyridyls being frozen.

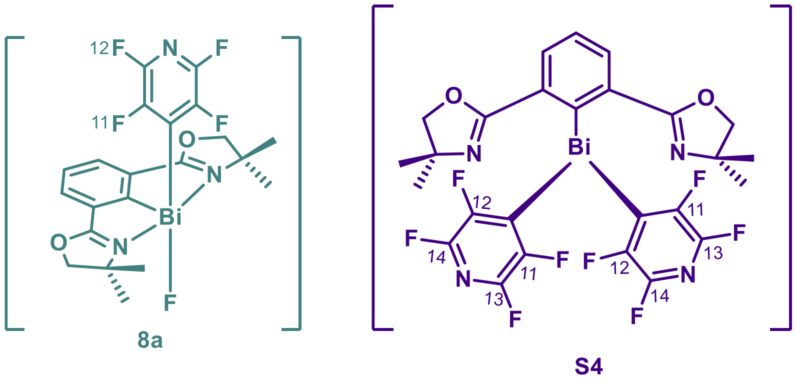

^{1}H-^{19}F HOESY (-40 ^{o}C)

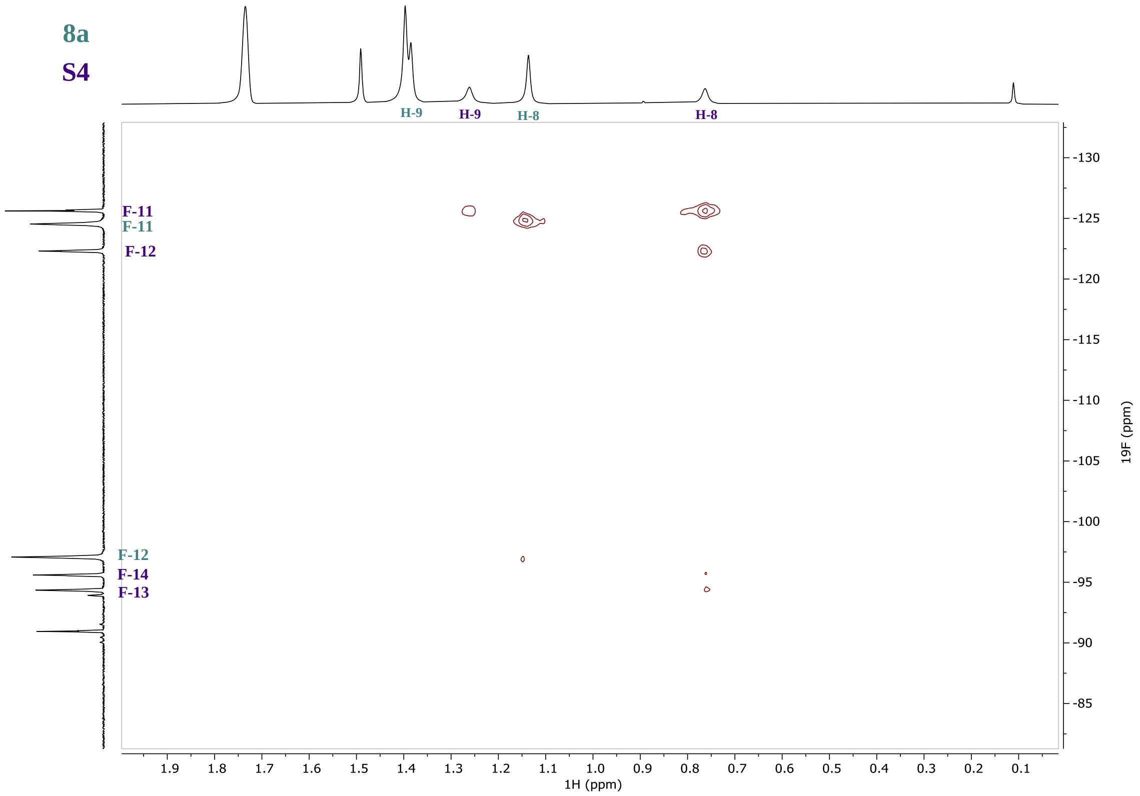

The ^{1}H-^{19}F HOESY at -40 ^{o}C reveals the spatial proximity of the 4-tetrafluoropyridyls to the same side CH_3 (H-8) of oxazoline ligands, confirming the connectivity of 4-tetrafluoropyridyls and Phebox ligands through Bi centers in **8a** and **S4**.

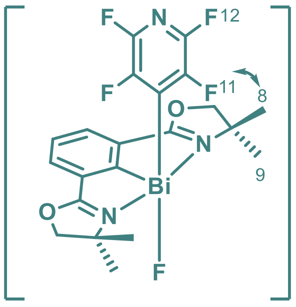

8a

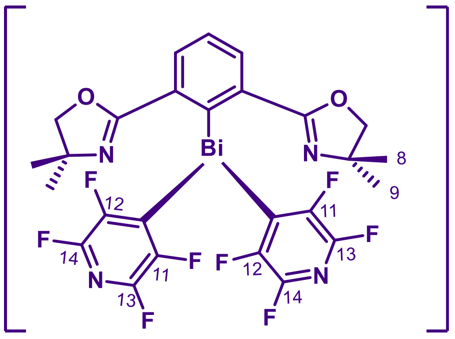

S4

^{19}F-^{19}F NOESY (-40 ^{o}C)

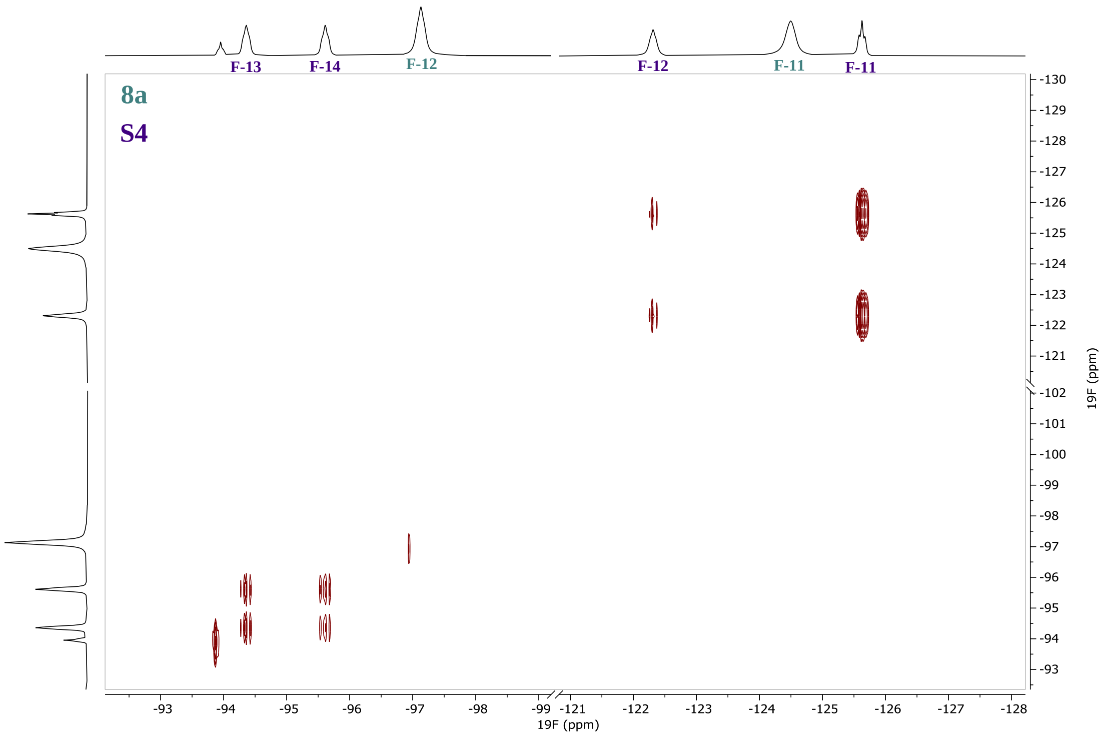

The ^{19}F-^{19}F NOESY at -40 ^{o}C reveals the dynamic structure of **S4**, which is consistent with ^{1}H-^{1}H-ROESY experiment.

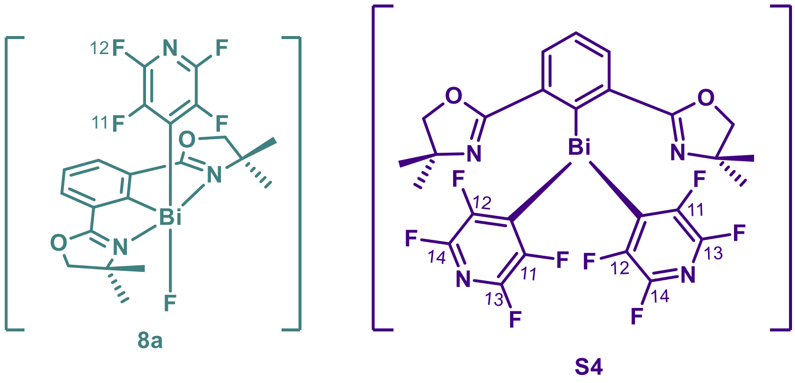

^{1}H NMR (THF-d_8, 500 MHz, -40 ^{o}C)

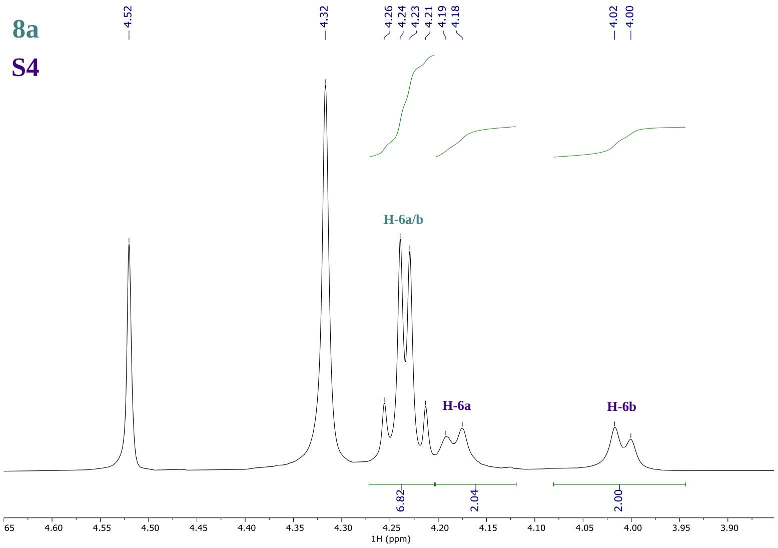

^{19}F NMR (THF-d_8, 470 MHz, -40 ^{o}C)

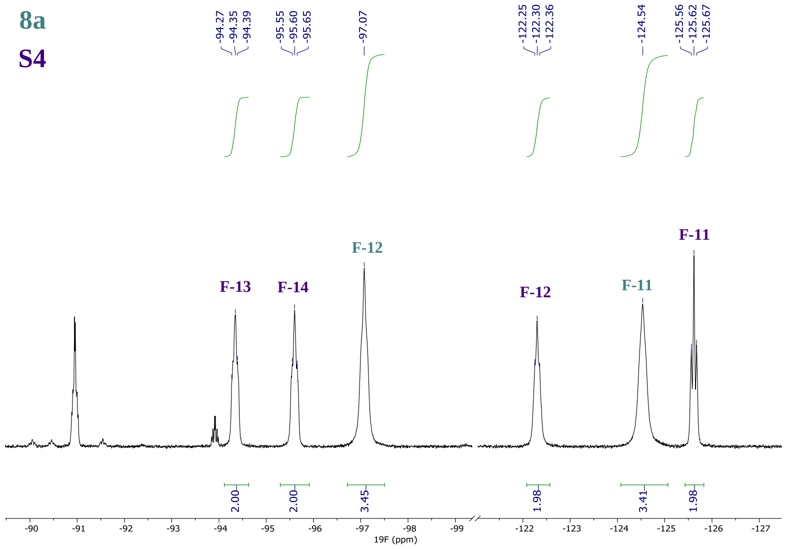

By comparing the integration ratios between **8a** and **S4** in ^{1}H and ^{19}F spectra, it isconcluded that ***S4*** *contains two 4-tetrafluoropyridyls*, which is consistent with its dynamic behavior.

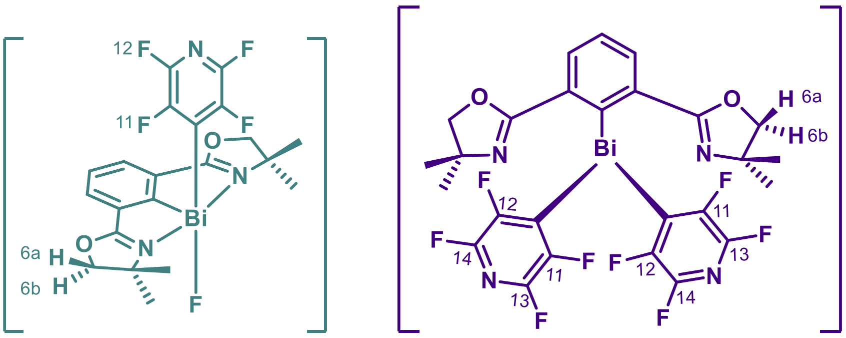

8a **S4**

4.3. Characterization and Reactivities of Phebox-Bi(4-tetrafluoropyridyl) triflate (**8b**)

The complex equilibriums in the OA reaction mixture impedes the full characterizations of the initial Phebox-Bi(4-tetrafluoropyridyl) fluoride (**8a**). It is reasoned that fluoride abstraction of the mixture with LiOTf might lead to a single cationic species Phebox-Bi(4-tetrafluoropyridyl) triflate (**8b**) with enhanced stability, which in turn sheds light on the structure of **8a**.

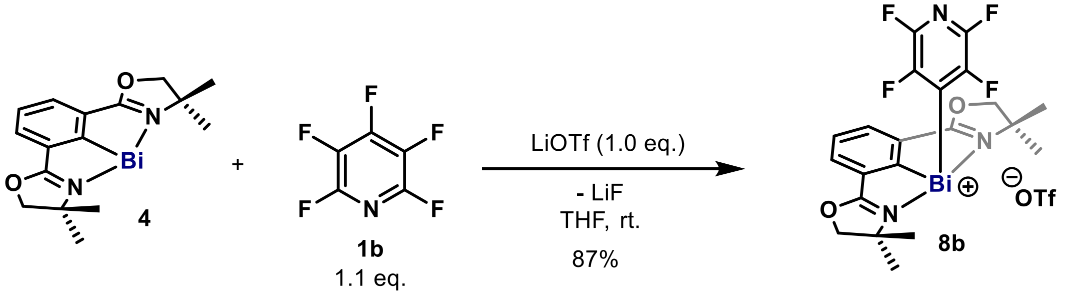

Procedure: In a glovebox, Phebox-Bi(I) (**4**, 100.0 mg, 208.2 µmol), LiOTf (32.5 mg, 208.2 µmol, 1.0 equiv.) and 7 mL anhydrous THF were added to a flame-dried 25 mL Schlenk flask. Upon rapid stirring, a THF stock solution of pentafluoropyridine (0.208 M, 1.1 mL, 1.1 equiv.) was added dropwise, resulting in a pale yellow solution after 10 min. The solution was filtered and the filtrate was layered with 10 mL dry *n*-pentane. After recrystallization for 2 days, the mother liquor was decanted and the product was washed with 5 mL anhydrous *n*-pentane. After drying on the Schlenk line, **8b** was afforded as a white crystalline solid (140.8 mg, 87%).

Note: due to high moisture-sensitivity of **8b**, *all reagents, solvents and equipment were rigorously dried prior to use*. Particularly, LiOTf was dried by diffusion pump (10^{-4} mbar) at 160 °C for 3 days.

Phebox-Bi(4-tetrafluoropyridyl) triflate (**8b**)

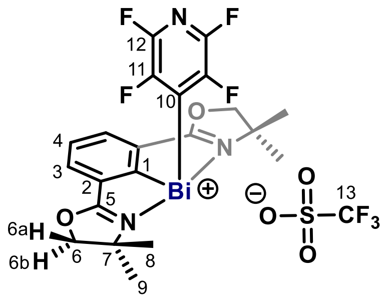

^{1}H NMR (600 MHz, THF-*d*$_8$): δ 8.30 (d, *J* = 7.6 Hz, 2H, H-3), 7.85 (t, *J* = 7.7 Hz, 1H, H-4), 4.59 (d, *J* = 8.6 Hz, 2H, H-6a), 4.56 (d, *J* = 8.6 Hz, 2H, H-6b), 1.60 (s, 6H, H-9), 1.27 (s, 6H, H-8).

^{13}C NMR (151 MHz, THF-*d*$_8$): δ 191.1 (C-1), 182.3 (C-5), 171.3 (t, *J* = 45.7 Hz, C-10), 147.2 – 143.7 (m, C-11/12), 134.7 (C-2), 134.6 (C-3), 130.9 (C-4), 121.6 (q, *J* = 321.2 Hz, C-13), 84.1 (C-6), 68.3 (C-7), 28.4 (C-9), 27.9 (C-8).

^{19}F NMR (565 MHz, THF-*d*$_8$): δ -77.5 (s, 3F, F-13), -92.0 – -92.4 (m, 2F, F-12), -121.2 – -121.7 (m, 2F, F-11).

M.p.: > 200 °C (dec.).

Positive-ion ESI-HRMS: calc'd for $C_{21}H_{19}BiF_4N_3O_2^+$ [M-OTf]$^+$ 630.12117; found 630.12215.

Negative-ion ESI-MS: [OTf]$^-$ 149.0.

Single crystals of **8b** suitable for X-ray crystallographic analysis were obtained by slow diffusion of *n*-pentane into a THF solution of **8b** at room temperature under argon over 2 days.

The reactivities:

a. Redox reactivity

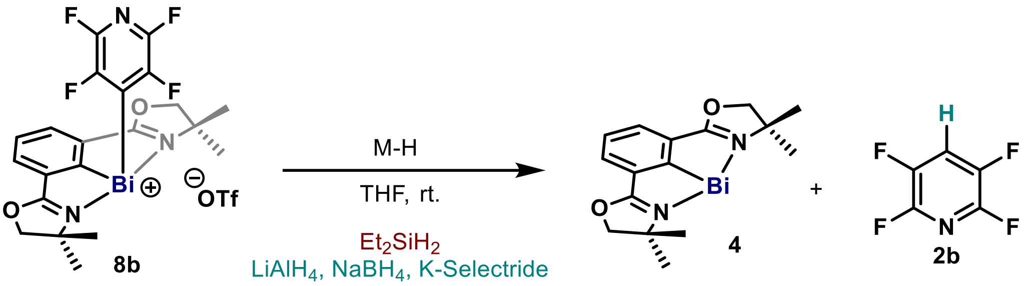

Due to the absence of fluoride anion, **8b** shows no reactivity towards hydrosilanes (eg. Et_2SiH_2) while metal hydrides ($LiAlH_4$, $NaBH_4$ and K-Selectride) are able to reduce **8b** to **4** instantaneously at room temperature with the formation of **2b**.

The mechanism of reduction is proposed to be C-H reductive elimination from Phebox-Bi(4-tetrafluoropyridul) hydride (**9**, *vide infra*).

b. Moisture-sensitivity

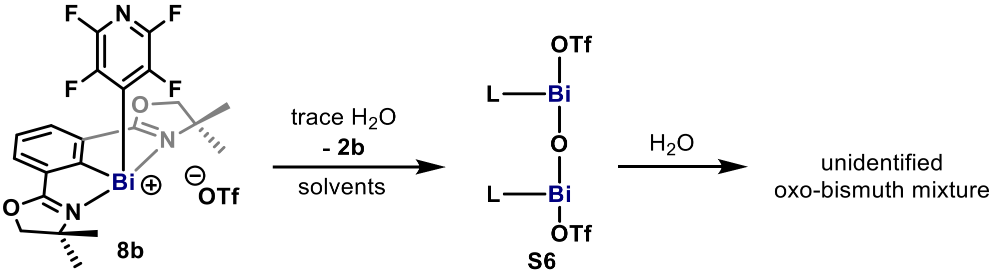

The perfluoro-aryl and –alkyl bismuth compounds are known to be highly moisture-sensitive.[15-16] Although **8b** is more stable than the original **8a**, partial hydrolysis of **8b** occurred in slightly moist solvents and the oxo-bismuth byproduct gradually crushed out as single crystals. X-ray crystallographic analysis revealed that it is μ-O bridging [Phebox-Bi(OTf)]$_2$O (**S6**), however, further discussion was not allowed because of the poor quality of the crystals. Correspondingly, quantitative ^{1}H NMR showed the formation of equal amount of **2b**. It is proposed that the hydrolysis of the highly polarized Bi-C bond leads to Phebox-Bi(OH)OTf and releases **2b**. Dehydration of Phebox-Bi(OH)OTf in anhydrous solvents gives **S6**.

Addition of H_2O (ca. 20 equiv.) to the THF-d_8 solution of **S6** leads to a mixture of oxo-bismuth species.

The NMR data of **S6** was extracted from the NMR spectra of partially hydrolyzed **8b**.

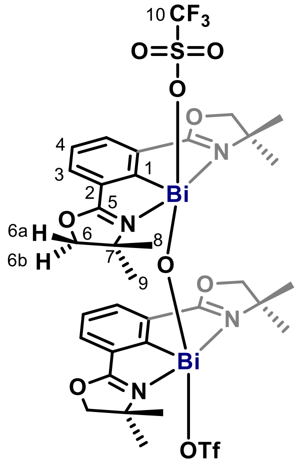

^{1}H NMR (400 MHz, CD$_3$CN): δ 8.23 (d, *J* = 7.6 Hz, 2H, H-3), 7.80 (t, *J* = 7.7 Hz, 1H, H-4), 4.48 (d, *J* = 8.9 Hz, 2H, H-6a), 4.39 (d, *J* = 8.9 Hz, 2H, H-6b), 1.44 (s, 6H, H-9), 1.32 (s, 6H, H-8).

^{13}C NMR (101 MHz, CD$_3$CN): δ 214.4 (C-1), 182.3 (C-5), 135.3 (C-3), 133.2 (C-2), 131.2 (C-4), 121.9 (q, *J* = 320.7 Hz, C-10), 83.7 (C-6), 68.0 (C-7), 28.7 (C-9), 28.0 (C-8).

^{19}F NMR (565 MHz, THF-*d$_8$*): δ -77.5 (s, 3F, F-10).

HRMS (ESI): calc'd for $C_{32}H_{38}N_4O_5Bi_2^{2+}$ $[M\text{-}2OTf]^{2+}$ 488.121983; found 488.121560.

4.4. Phebox-Bi(4-tetrafluoropyridyl) hydride (**9**) and C-H Reductive Elimination

4.4.1. Generation of **9** via LAH reduction of **8b**

Known reactivities of reported Bi-H

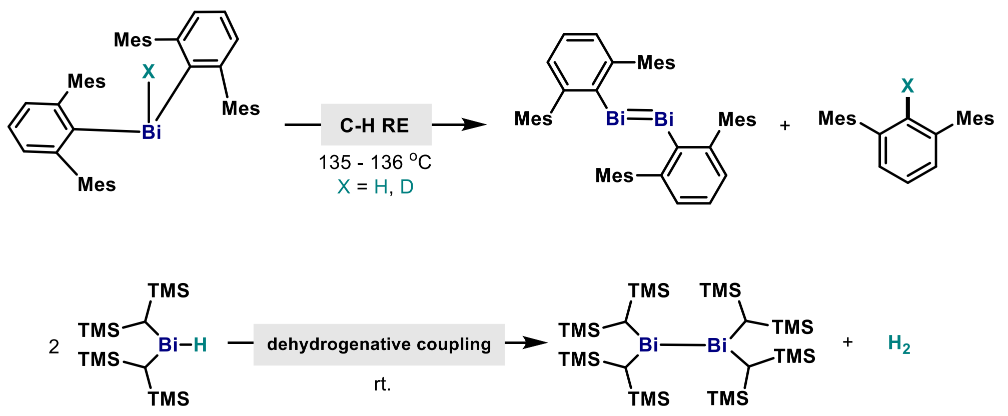

Bismuth hydrides are highly unstable and elusive species,[17] which are prone to release H_2 and form metallic Bi,[18] Bi(I)[2, 19] or Bi(II)-Bi(II) bonds[20]. Reported by Power in 2000, (2,6-$Mes_2H_3C_6)_2BiH$ is the only stable and well-defined bismuth hydride until now.[21] This compound indicated an alternative reaction pathway, C-H/D reductive elimination, yielding stable dibismuthene (Ar-Bi(I)=Bi(I)-Ar) and Ar-H/D. By analogy, the formation of relatively stable PheboxBi(I) (**4**) could also serve as the driving force for C-H reductive elimination.

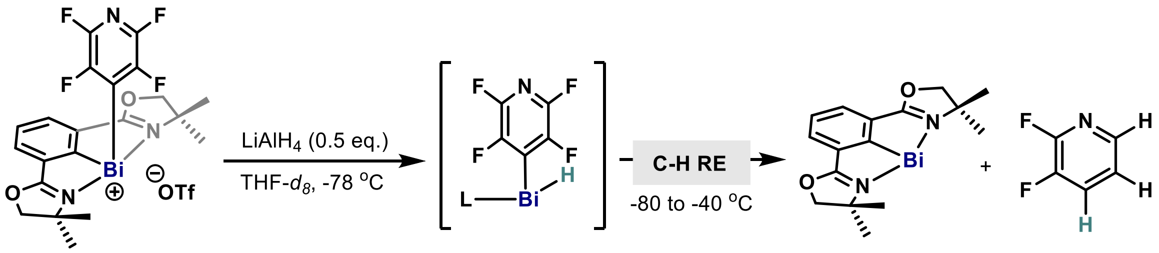

8b **9** **4** (ca. 90%) **2b** (ca. 80%)

Procedure: In a glovebox, **8b** (5.0 mg, 6.4 µmol) and 0.5 mL anhydrous THF-d_8 were added to a J-Young NMR tube. The NMR tube was taken out of glovebox and cooled by dry ice. A THF-d_8 stock solution of $LiAlH_4$ (0.0321 M, 100 µL, 0.5 equiv.) was added under argon. The J-Young NMR tube was kept under -78 °C and quickly shaked once before NMR measurement (-80 °C). The sample turned pale green after shaking, indicating the partial formation of **4** in the beginning of the NMR study.

In the NMR study, the sample was allowed to warm up from -80 °C to -60 °C and then to -40 °C.

^{1}H NMR spectra at different temperatures (-80 °C to -40 °C)

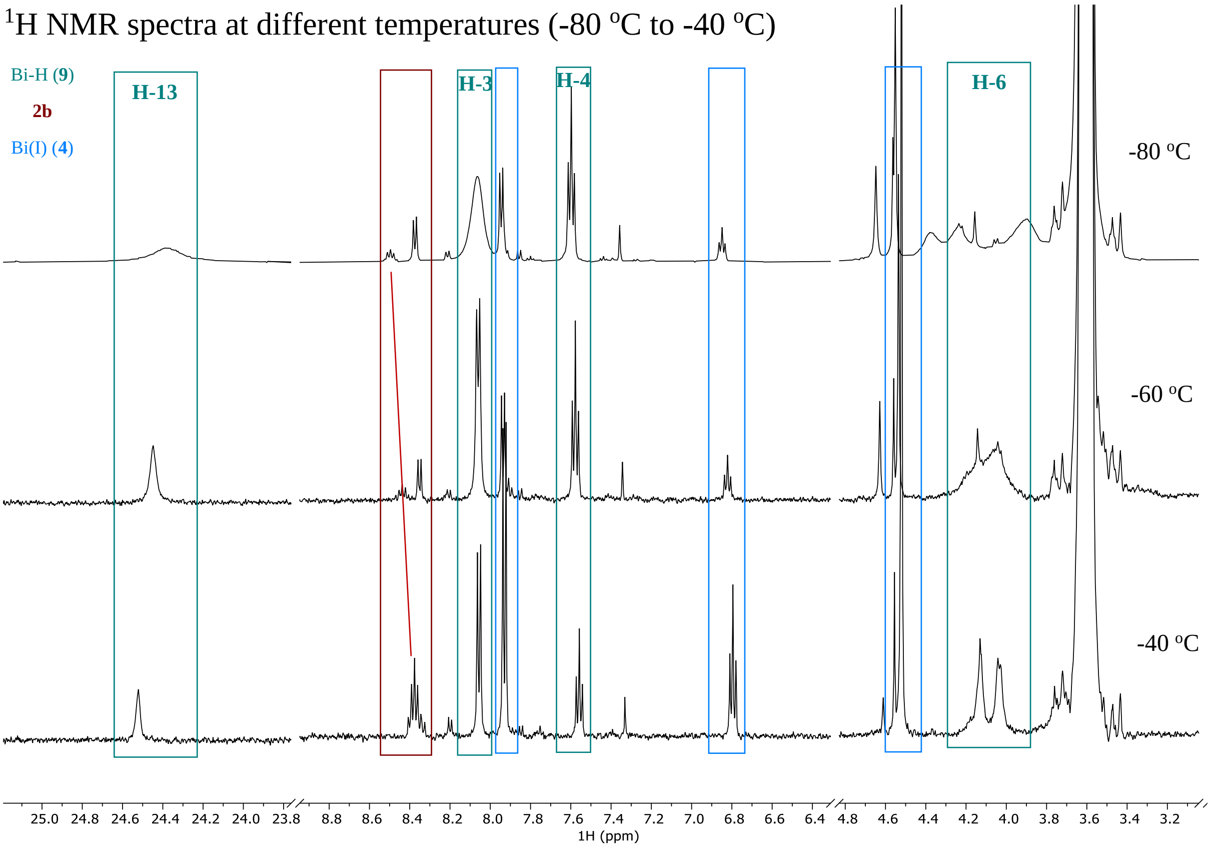

Variable temperature ^{1}H NMR data (-80 °C to –40 °C) showed that **8b** reacted rapidly with $LiAlH_4$ to give a new species **9**. According to the distinct hydride signal (H-13, br., 24.52 ppm, -40 °C), **9** is Phebox-Bi(4-tetrafluoropyridyl) hydride and the chemical shift of this hydride is comparable to the reported value for $(2,6\text{-}Mes_2H_3C_6)_2BiH$ (19.39 ppm). However, the chemical shifts of the hydrides of alkylbismuth analogues, Me_2BiH[22] and $(TMS_2CH)_2BiH$[20a], are very different (2.10 and 3.24 ppm, respectively). Apparently, heavy Bi atoms exert a drastic influence on the attached H atoms and this influence is highly dependent on the supporting ligands, which could be explained by the spin-orbital heavy-atom effect on the light-atom (SO-HALA effect). [23]

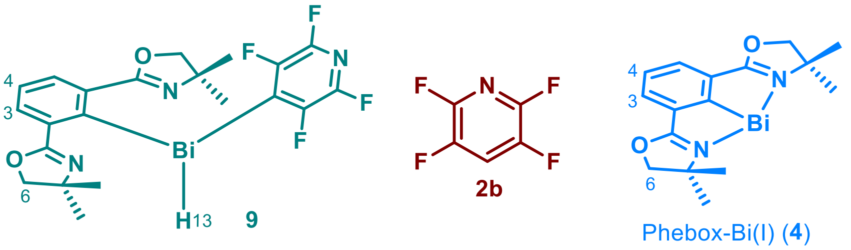

^{1}H NMR (THF-d_8, 500 MHz, -40 ^{o}C)

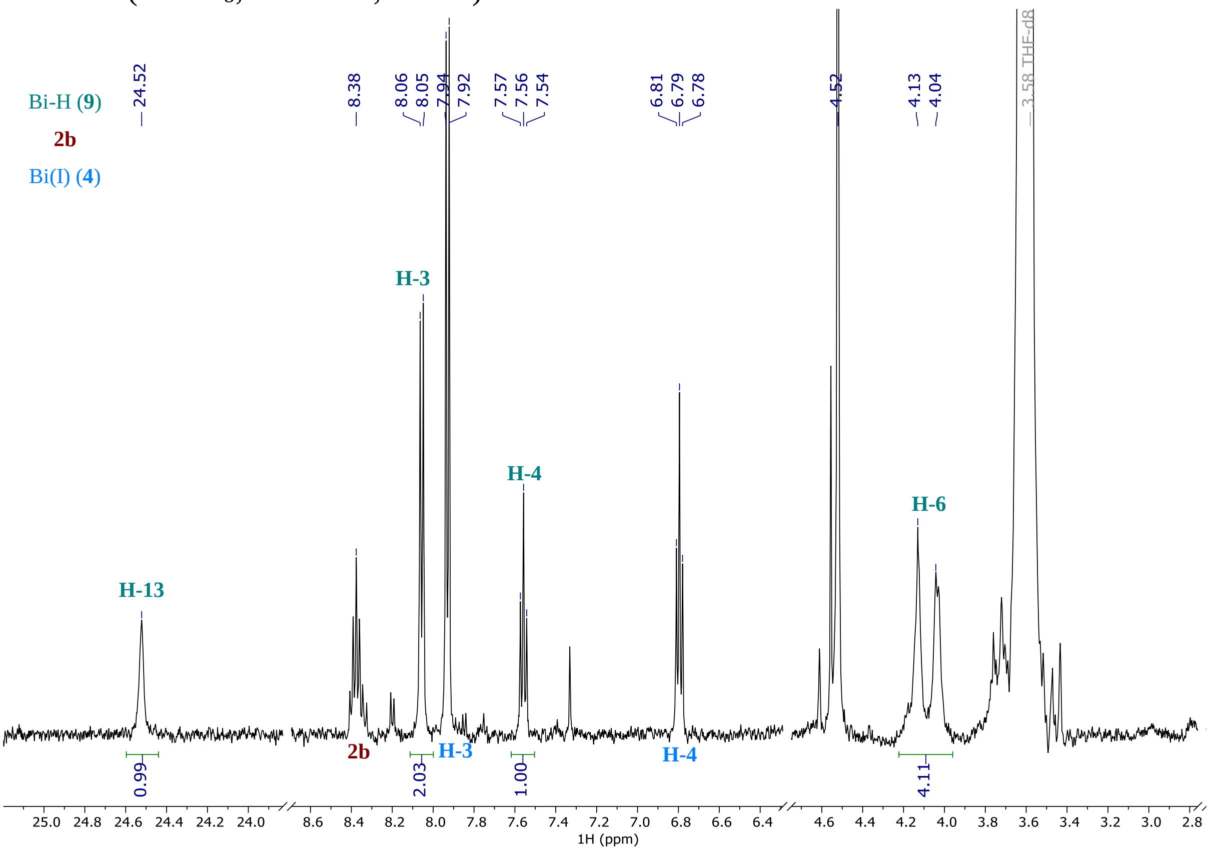

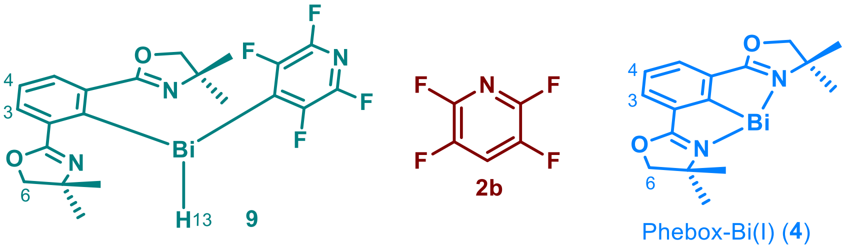

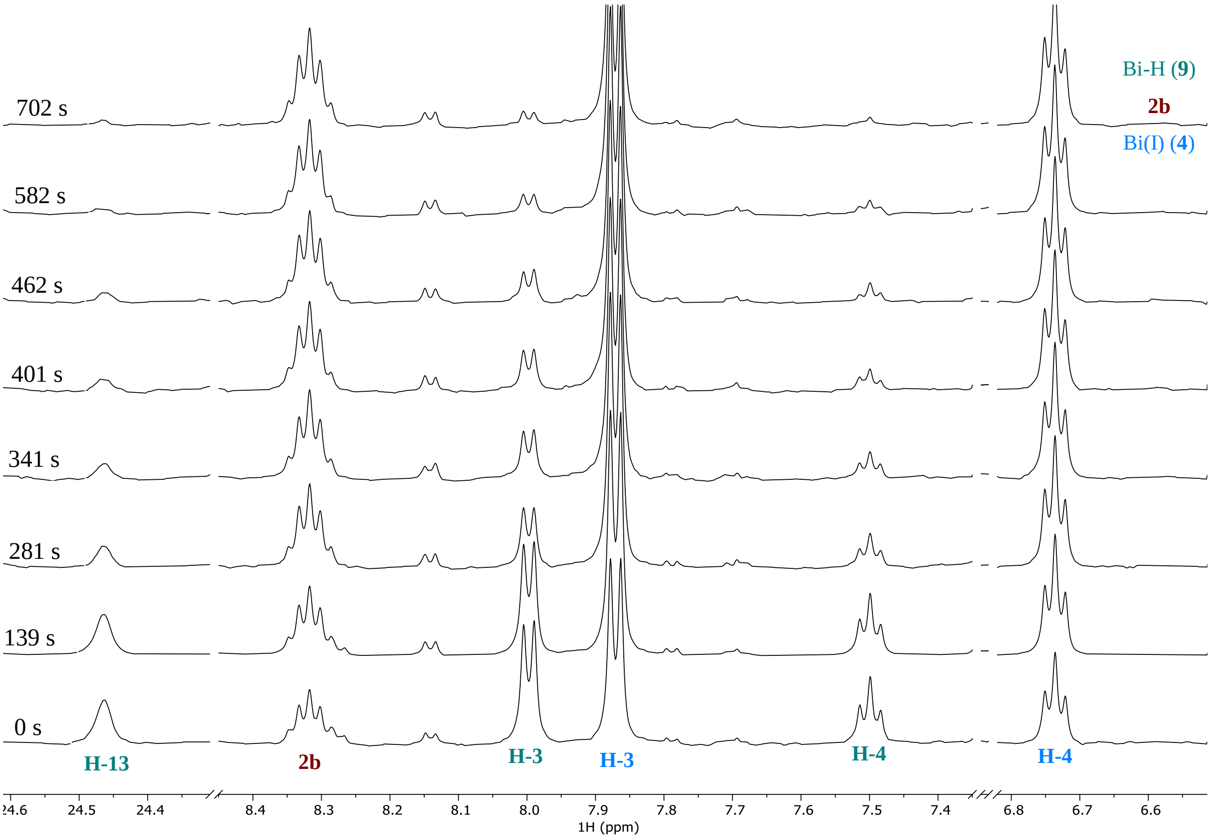

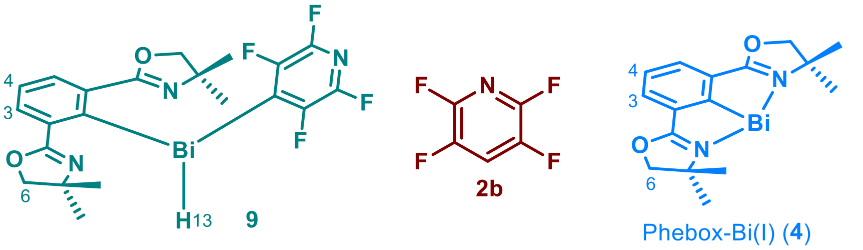

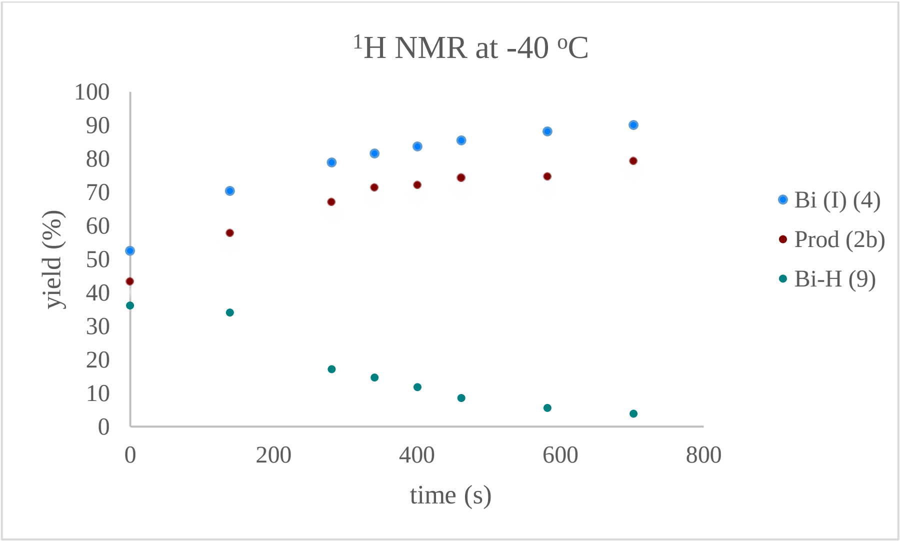

At -40 °C, Bi-H (**9**) rapidly decayed while Bi(I) (**4**) and **2b** increased by approximately the same magnitude in 700 s.

4.4.2. NMR Characterization of **9** under the Catalytic Condition

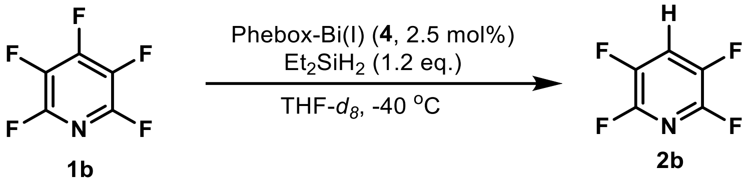

Under the catalytic condition, Bi-H (**9**) was found to be the major Bi species and remained relatively stable in concentration before the late stage of the catalytic HDF (*vide infra*), which allowed further characterization of the short-lived **9** under the catalytic condition using 600 MHz NMR with CryoProbe.

Procedure: In a glovebox, **4** (1.2 mg, 2.5 µmol, 2.5 mol%) and 100 µL anhydrous THF-d_8 were added to a J-Young NMR tube. The NMR tube was taken out of glovebox and cooled by dry ice. A 0.5 mL THF-d_8 solution of **1b** (16.9 mg, 0.1 mmol) and Et_2SiH_2 (10.6 mg, 0.12 mmol, 1.2 equiv.) was added under argon. The J-Young NMR tube was kept at -78 °C and quickly shaken once before insertion into the NMR probe which was precooled to -40 °C. After shimming, the NMR measurements were immediately started.

^{1}H NMR spectra at different time points during the reaction (THF-d_8, 600 MHz, -40 °C)

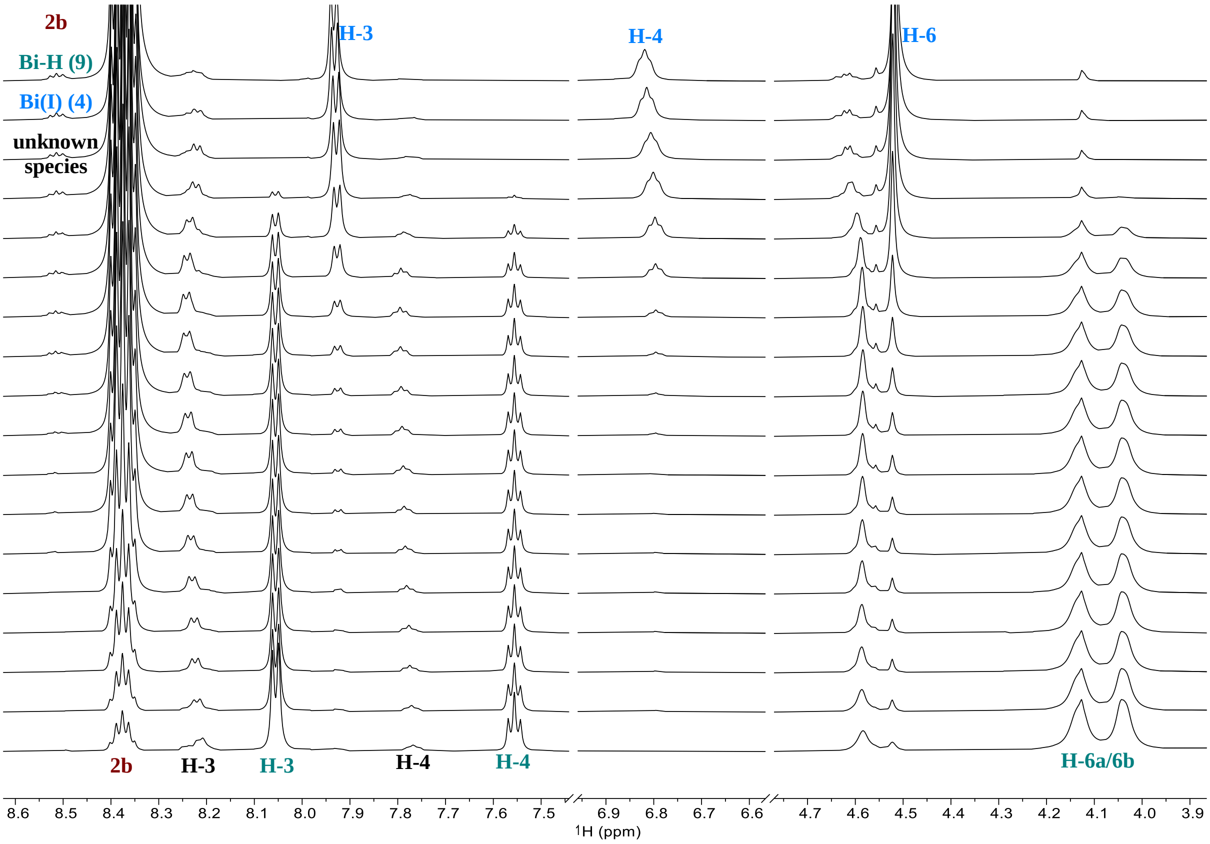

^{1}H NMR spectra taken at different time points during the reaction showed that Bi-H (**9**) was the major species and its concentration remained relatively stable during the catalytic reaction. Bi(I) (**4**) remained at low concentration and was regenerated after the reaction. Besides this, there was another unidentified Bi species which carried 4-tetrafluoropyridyl and remained at low concentration. The kinetic profiles of **2b**, **4** and **9** are shown as below.

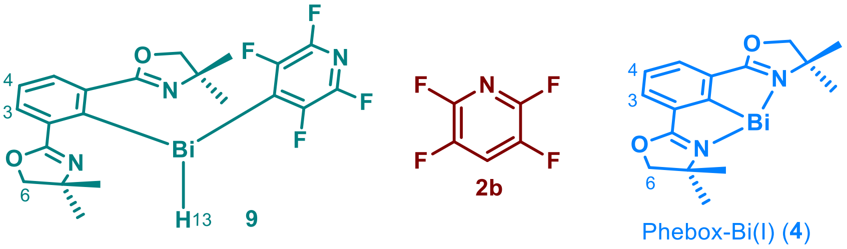

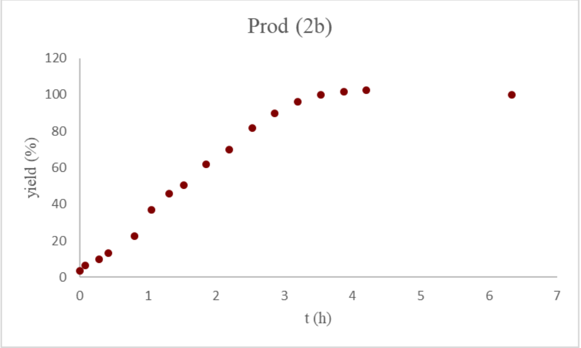

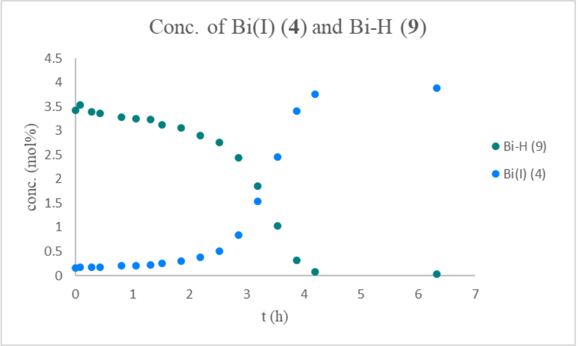

^{1}H NMR kinetic profile including Bi-H region (THF-d_8, 500 MHz, -40 °C)

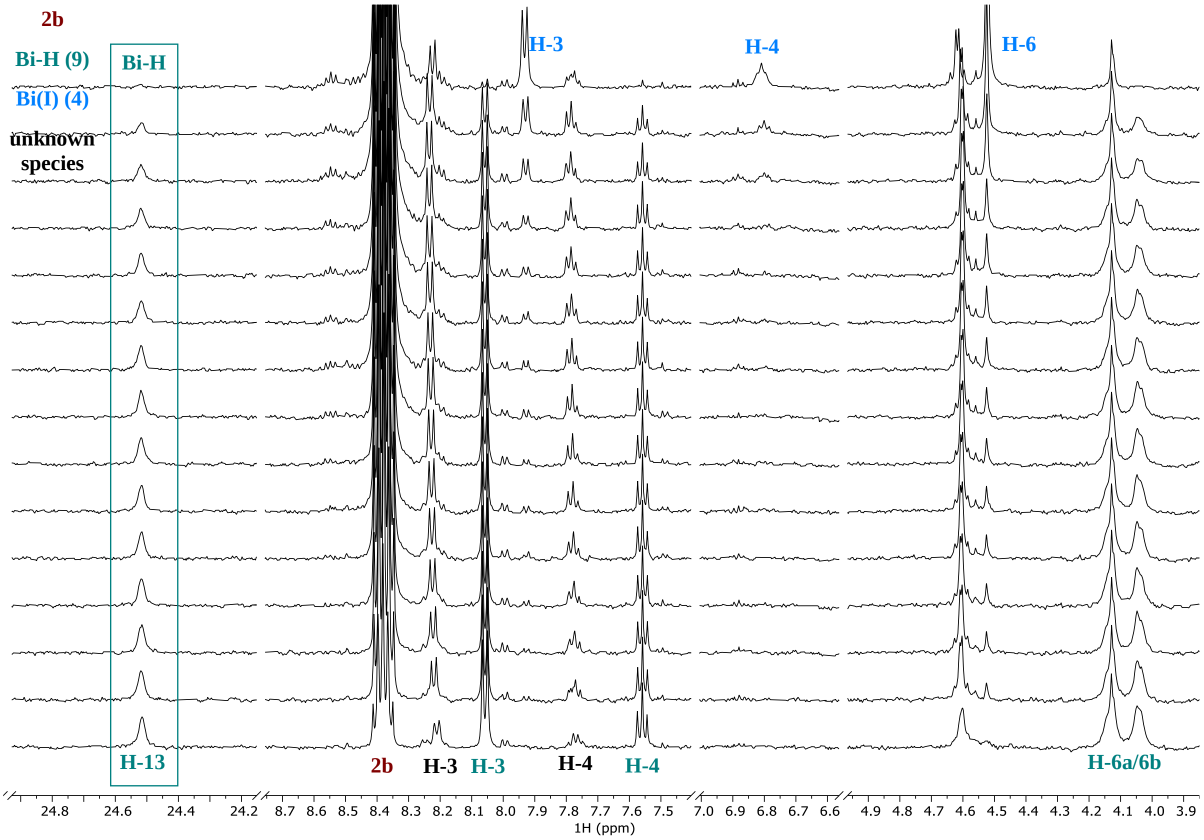

The catalytic reaction was repeated and the ^{1}H NMR kinetic profile was recorded for this sample using 500 MHz NMR instrument. The Bi-H region was monitored in this NMR experiment, from which the NMR spectra of Figure 4B middle was extracted.

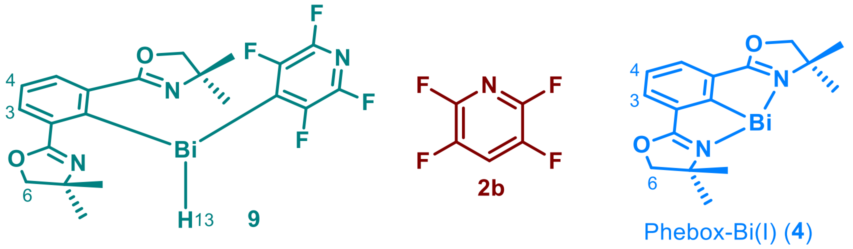

Time-stacked ^{19}F NMR spectra (THF-d_8, 565 MHz, -40 °C)

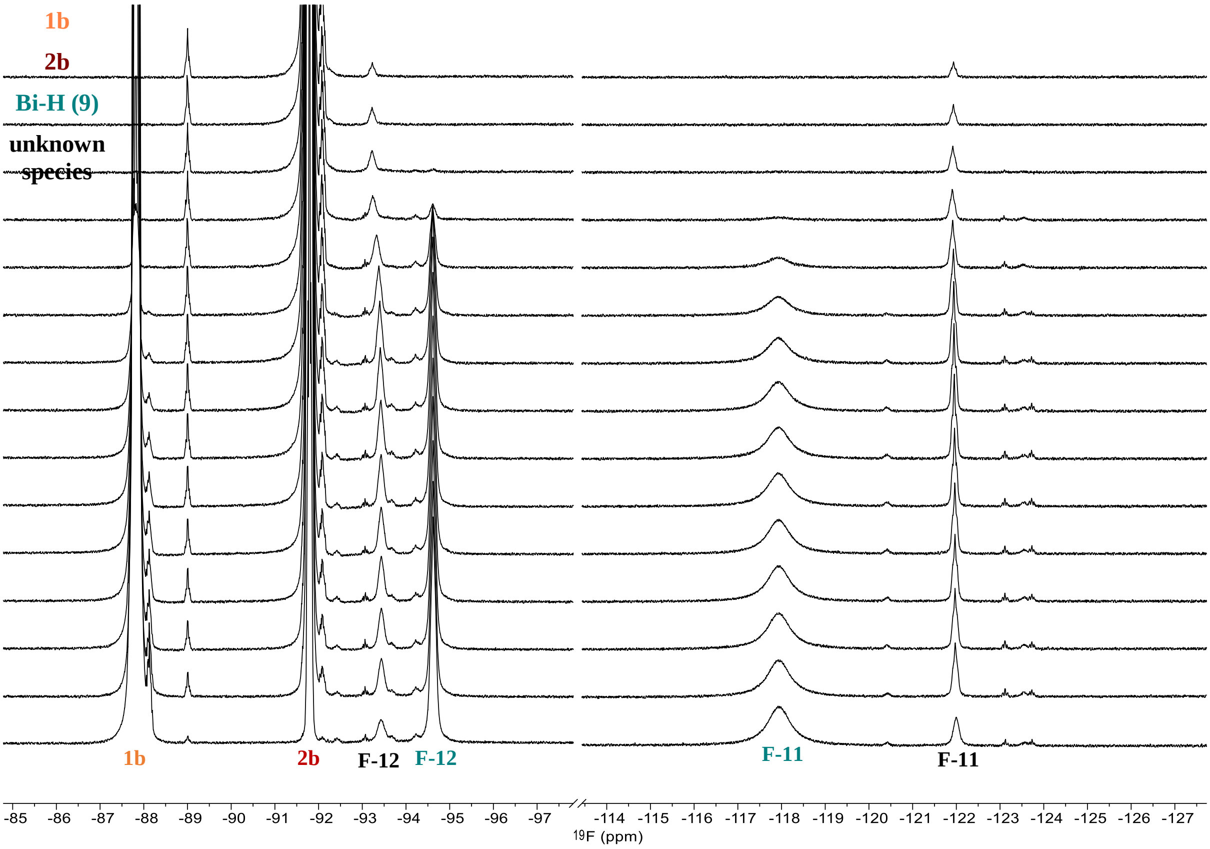

In ^{19}F NMR, *o*-Fs (F-11) and *m*-Fs (F-12) of Bi-H (**9**) were found at -117.9 and -94.6 ppm. *o*-Fs are considerably broadended probably due to restricted rotation of the 4-tetrafluoropyridyl of **9**. Besides this, ^{19}F NMR signals at -122.0 and -93.4 ppm were assigned to the *o*-Fs (F-11) and *m*-Fs (F-12) of the unidentified Bi species.

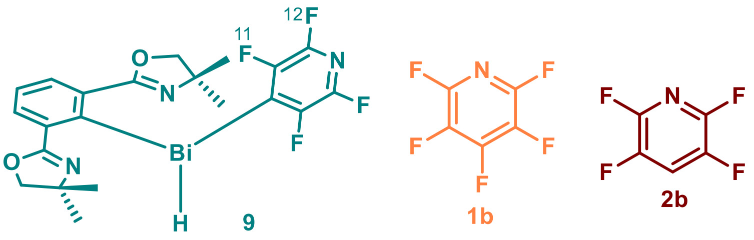

^{1}H-^{13}C-*edited*-HSQC acquired during the catalytic reaction

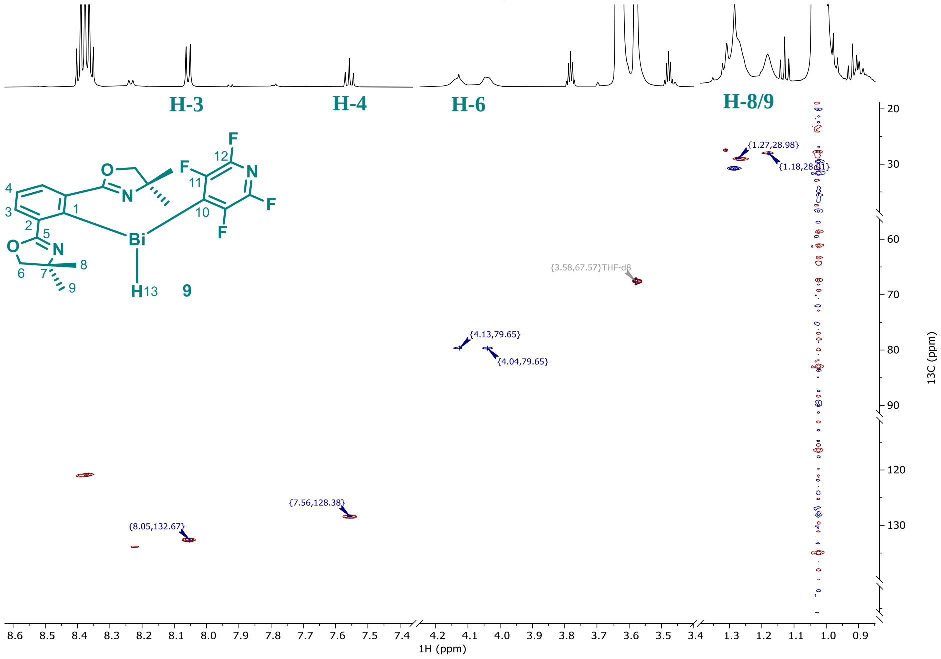

^{1}H-^{13}C HMBC acquired during the catalytic reaction

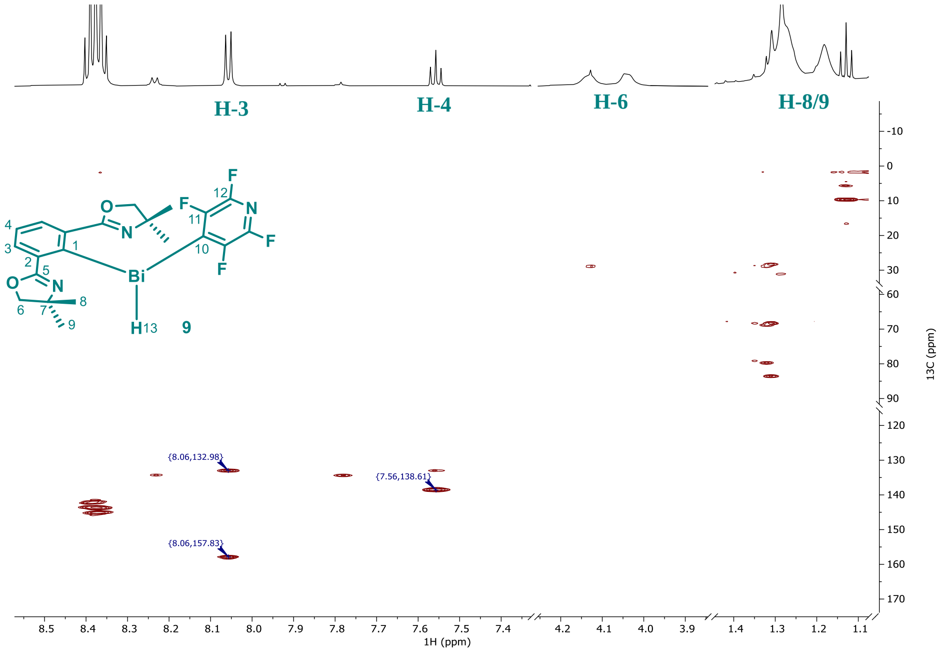

4.4.3. Characterization Data of **9** and Structural Information of **9** Obtained from NMR Studies

^{1}H/^{13}C/^{19}F signals of **9** were largely assigned based on NMR data of stoichiometric and catalytic reactions.

Phebox-Bi(4-tetrafluoropyridyl) hydride (**9**)

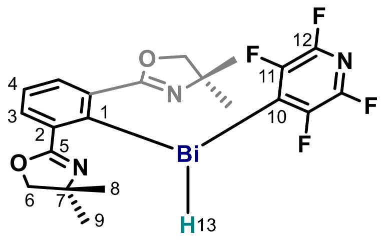

^{1}H NMR (500 MHz, THF-d_8, -40 ºC): δ 24.52 (s, br., 1H, H-13), 8.06 (d, *J* = 7.8 Hz, 2H, H-3), 7.56 (t, *J* = 7.7 Hz, 1H, H-4), 4.20 – 4.09 (m, br., 2H, H-6), 4.08 – 3.98 (m, br., 2H, H-6'), 1.27 (s, br., 6H, H-9), 1.17 (s, br., 6H, H-8).

^{1}H NMR shifts were extracted from ^{1}H NMR spectra of the reduction of **8b** with $LiAlH_4$.

^{13}C NMR (151 MHz, THF-d_8, -40 ºC): δ 157.8 (C-5), 138.6 (C-2), 132.7 (C-3), 128.4 (C-4), 79.7 (C-6), 29.0 (C-9), 28.0 (C-8).

^{13}C NMR shifts were extracted from HSQC and HMBC data of the catalytic HDF. The chemical shifts of C-1, C-7, C-10, C-11 and C-12 could not be determined.

^{19}F NMR (565 MHz, THF-d_8, -40 ºC): δ -94.6 (2F, F-12), -117.9 (br., 2F, F-11).

^{19}F NMR shifts were extracted from the ^{19}F NMR spectrum of the catalytic HDF.

MS studies: mass determination of **9** was attempted under both stoichiometric and catalytic conditions. The reaction systems were very sensitive towards temperature. As a result, **9** was reduced back to Phebox-Bi(I) (**4**) during the injections of the samples and only **4** was detected.

Structural information of **9** obtained from NMR studies

1. Non-coordinated oxazolines and trigonal pyramidal geometry of the Bi center

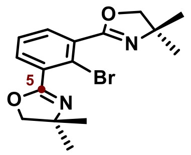

Phebox-Br (**10**)

161.9

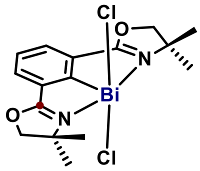

Phebox-BiCl$_2$ (**6**)

181.9

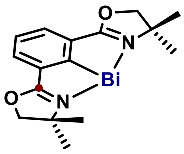

Phebox-Bi(I) (**4**)

172.7

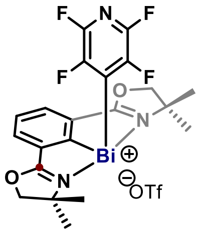

8b

182.3

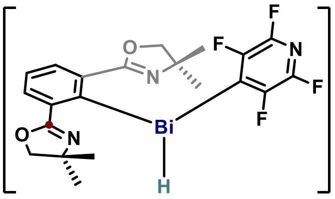

9

157.8

Table S3. A comparison of C-5 chemical shifts (ppm)

By comparing the chemical shifts of the C-5 of **9** and other Bi compounds where *oxazolines are coordinated to Bi centers in their X-ray single crystal structures*, it was found that the C-5 of **9** is noticeably more shielded than that of other Bi compounds, but similar to that of Phebox-Br (**10**). Together with the signal broadening of the elements around Bi (H-6, H-9, H-8, F-11), it is suggested that *the oxazolines in* ***9*** *remain non-coordinated to Bi and the Bi center adopts a trigonal pyramidal geometry*.

2. 4-Tetrafluoropyridyl is attached to the Bi center

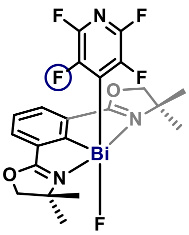

8a

-125.6

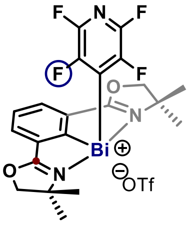

8b

-121.4

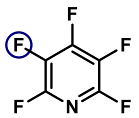

1b

-163.0

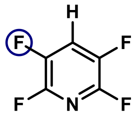

2b

-141.7

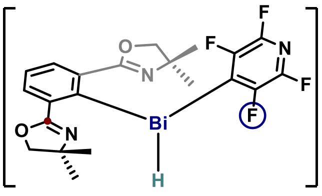

9

-117.9 (-40 °C)

Table S4. A comparison of F-11 chemical shifts (ppm)

By comparing the chemical shifts of *m*-Fs of **1b** and **2b** and F-11 of **8a**, **8b** and **9**, it is suggested that 4-tetrafluoropyridyl is attached to Bi in **9**.

4.4.4. Deuterium Kinetic Experiment

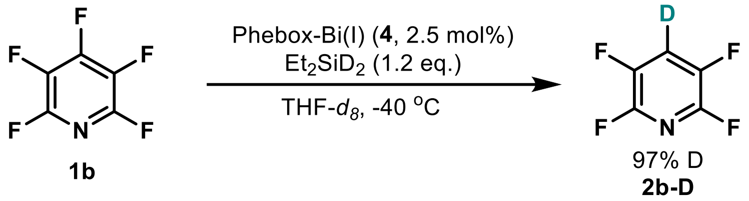

Et_2SiD_2 was prepared according to the reported procedure containing > 99% D.[24]

Diethylsilane-d_2

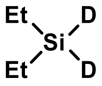

^{1}H NMR (300 MHz, $CDCl_3$): δ 1.02 (t, J = 7.8 Hz, 6H), 0.66 (q, J = 7.9 Hz, 4H).

^{13}C NMR (75 MHz, $CDCl_3$) δ 9.2, 1.0.

Procedure: In a glovebox, a 100 µL THF-d_8 stock solution of **4** (1.2 mg, 2.5 µmol, 2.5 mol%) was added to a J-Young NMR tube. The NMR tube was taken out of glovebox and cooled by dry ice. A 0.5 mL THF-d_8 stock solution of **1b** (16.9 mg, 0.1 mmol) and Et_2SiD_2 (10.8 mg, 0.12 mmol, 1.2 equiv.) was added under argon. The J-Young NMR tube was kept at -78 °C and quickly shaken once before insertion into the NMR probe which was precooled to -40 °C. After shimming, the NMR measurements were immediately started.

Time-stacked ^{1}H NMR spectra (THF-d_8, 500 MHz, -40 ^{o}C)

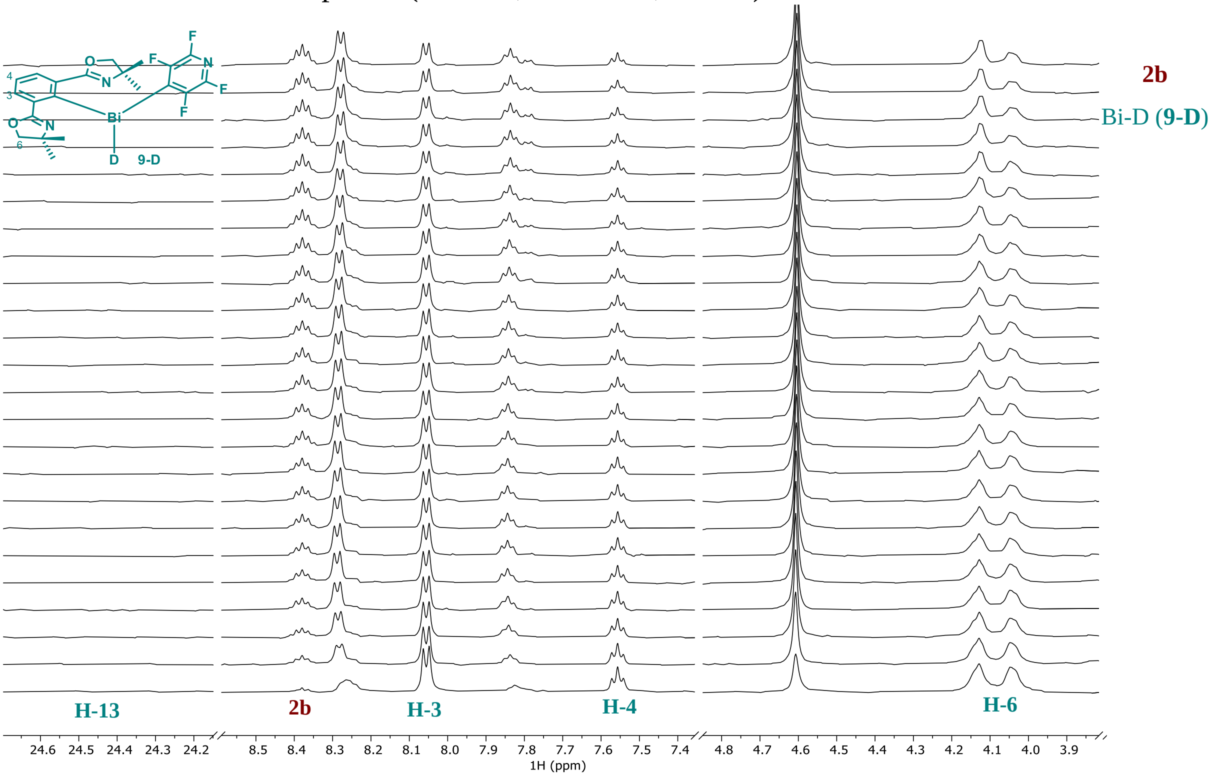

In the deuterium kinetic experiment, the formation of Phebox-Bi(4-tetrafluoropyridyl) deuteride (**9-D**) was indicated by the observation of the ligand backbone signals (H-3/4/6) and disappearance of hydride signal (H-13).

Time-stacked ^{19}F NMR spectra (THF-d_8, 470 MHz, -40 ^{o}C)

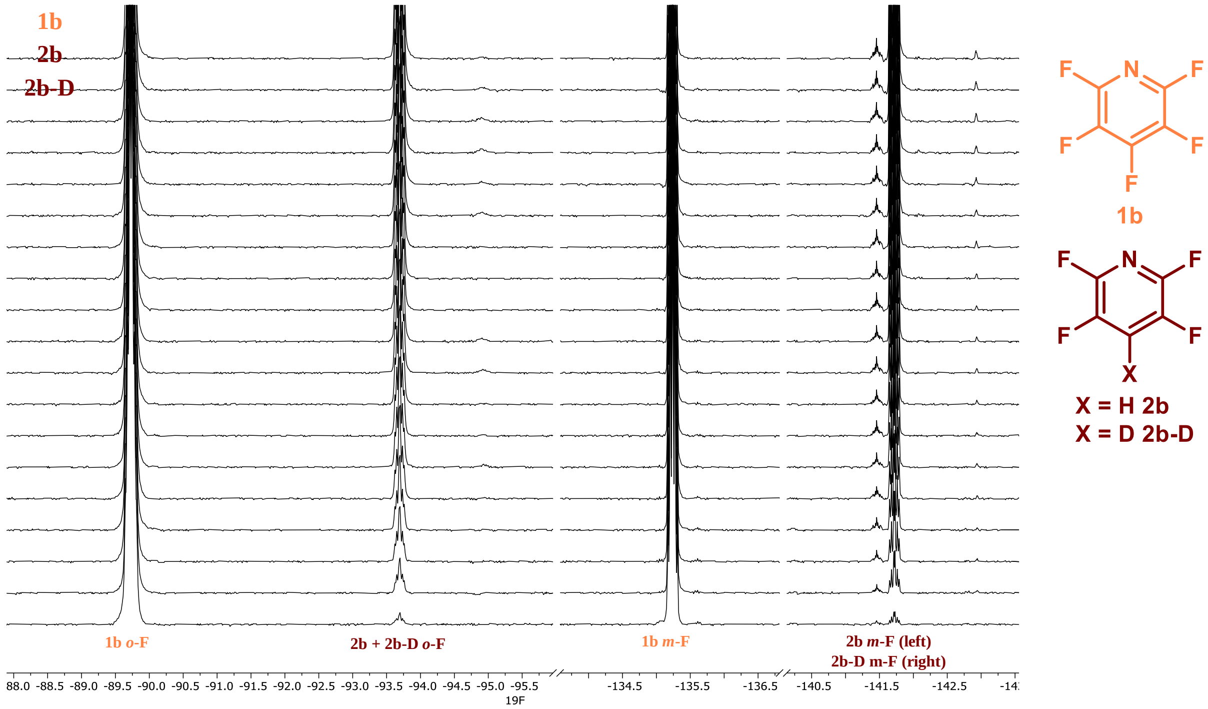

5. X-ray Crystallographic Studies

5.1. Single Crystal Structure Analysis of Complex **4**

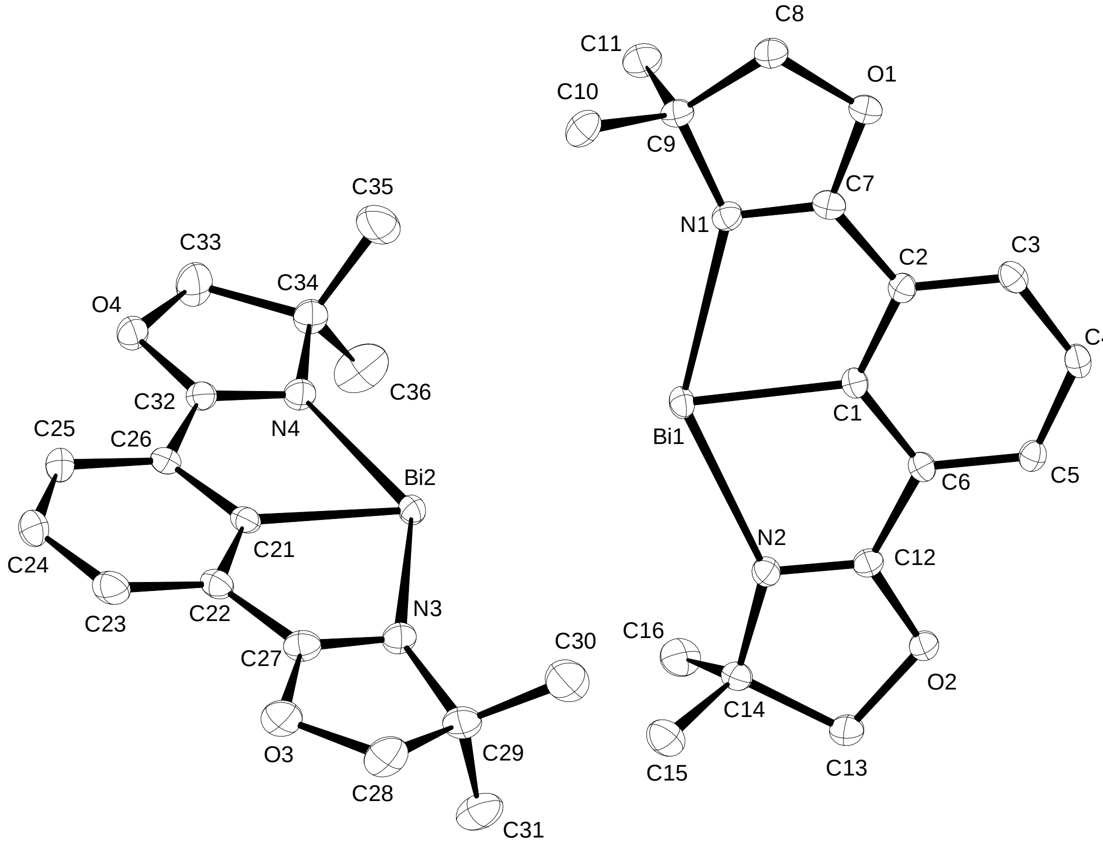

Figure S4. The molecular structure of complex **4**. H atoms have been removed for clarity.

X-ray Crystal Structure Analysis of complex 4: $C_{16}H_{19}BiN_2O_2$, M_r = 480.31 g mol^{-1}, dark-blue block, crystal size 0.13 x 0.12 x 0.11 mm^3, orthorhombic, $P2_12_12_1$ [19], a = 10.4582(13) Å, b = 11.8438(10) Å, c = 27.187(6) Å, V = 3367.5(9) Å^3, T = 100(2) K, Z = 8, D_{calc} = 1.895 g·cm^3, λ = 0.71073 Å, μ(*Mo-K$_\alpha$*)= 10.477 mm^{-1}, Gaussian absorption correction ($T_{\min}$ = 0.27804, $T_{\max}$ = 0.40911), Bruker AXS Enraf-Nonius KappaCCD diffractometer with a FR591 rotating Mo-anode X-ray source, 2.704 < θ < 38.062°, 163558 measured reflections, 18415 independent reflections, 16672 reflections with $I > 2\sigma(I)$, R_{int} = 0.0477]. The structure was solved by *SHELXT* and refined by full-matrix least-squares (*SHELXL*) against F^2 to R_1 = 0.0247 [$I > 2\sigma(I)$], wR_2 = 0.0424, 387 parameters, absolute structure parameter Flack (x) = -0.0267(19).

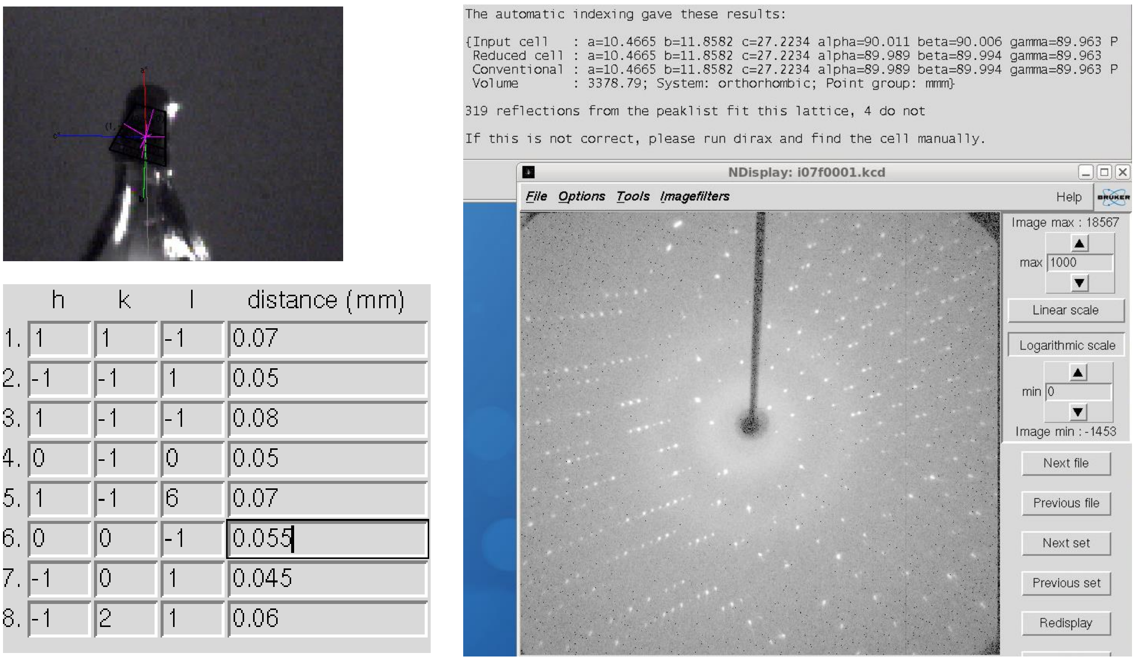

Figure S5. Crystal faces and unit cell determination of complex **4**.

INTENSITY STATISTICS FOR DATASET

Resolution	#Data	#Theory	%Complete	Redundancy	Mean I	Mean I/s	Rmerge	Rsigma
Inf - 2.35	277	287	96.5	12.79	127.89	90.95	0.0346	0.0105
2.35 - 1.56	660	660	100.0	15.94	86.09	93.22	0.0304	0.0092
1.56 - 1.24	927	927	100.0	15.47	58.34	79.14	0.0323	0.0104
1.24 - 1.08	952	952	100.0	14.01	44.18	66.51	0.0321	0.0123
1.08 - 0.98	945	945	100.0	12.81	34.76	55.86	0.0356	0.0148
0.98 - 0.91	946	946	100.0	11.86	29.08	47.47	0.0396	0.0172
0.91 - 0.86	851	851	100.0	11.12	24.70	41.92	0.0454	0.0200
0.86 - 0.81	1104	1104	100.0	10.43	19.84	35.04	0.0543	0.0241
0.81 - 0.78	795	795	100.0	9.89	17.83	30.18	0.0603	0.0273
0.78 - 0.75	911	911	100.0	9.43	15.24	26.55	0.0691	0.0319
0.75 - 0.72	1114	1114	100.0	8.21	15.05	23.85	0.0748	0.0358
0.72 - 0.70	809	809	100.0	6.82	12.60	19.03	0.0876	0.0455
0.70 - 0.68	959	959	100.0	6.59	11.14	16.79	0.0974	0.0519
0.68 - 0.66	1032	1032	100.0	6.27	9.34	14.09	0.1153	0.0626
0.66 - 0.64	1214	1214	100.0	5.95	8.42	12.20	0.1331	0.0742
0.64 - 0.63	638	638	100.0	5.72	8.25	11.31	0.1463	0.0803
0.63 - 0.62	693	693	100.0	5.66	7.62	10.24	0.1511	0.0892
0.62 - 0.60	1544	1544	100.0	5.48	6.84	8.75	0.1639	0.1073
0.60 - 0.59	823	823	100.0	5.17	5.86	6.79	0.1997	0.1448
0.59 - 0.58	1269	1273	99.7	4.99	5.16	5.25	0.2310	0.1920
0.68 - 0.58	7213	7217	99.9	5.59	7.26	9.62	0.1549	0.1021
Inf - 0.58	18463	18477	99.9	8.87	22.19	30.93	0.0471	0.0285

Nine poorly measured reflections were removed from the data set before the final refinement cycles. Complete .cif-data of the compound are available under the CCDC number **CCDC-2091966**.

Table S5. Crystal data and structure refinement of complex **4**.

Identification code	13754	
Empirical formula	$C_{16} H_{19} Bi N_2 O_2$	
Color	dark-blue	
Formula weight	480.31 g · mol^{-1}	
Temperature	100(2) K	
Wavelength	0.71073 Å	
Crystal system	orthorhombic	
Space group	$P2_12_12_1$, (No. 19)	
Unit cell dimensions	a = 10.4582(13) Å	α= 90°.
	b = 11.8438(10) Å	β= 90°.
	c = 27.187(6) Å	γ = 90°.
Volume	3367.5(9) $Å^3$	
Z	8	
Density (calculated)	1.895 Mg · m^{-3}	
Absorption coefficient	10.477 mm^{-1}	
F(000)	1824 e	
Crystal size	0.13 x 0.12 x 0.11 mm^3	
θ range for data collection	2.704 to 38.062°.	
Index ranges	-18 ≤ h ≤ 18, -20≤ k ≤ 20, -47 ≤ l ≤ 47	
Reflections collected	163558	
Independent reflections	18415 [R_{int} = 0.0477]	
Reflections with I>2σ(I)	16672	
Completeness to θ = 25.242°	99.7 %	
Absorption correction	Gaussian	
Max. and min. transmission	0.41 and 0.28	
Refinement method	Full-matrix least-squares on F^2	
Data / restraints / parameters	18415 / 0 / 387	
Goodness-of-fit on F^2	1.123	
Final R indices [I>2σ(I)]	R_1 = 0.0247	wR^2 = 0.0424
R indices (all data)	R_1 = 0.0326	wR^2 = 0.0442
Absolute structure parameter	-0.0267(19)	
Largest diff. peak and hole	1.3 and -1.1 e · $Å^{-3}$	

Table S6. Bond lengths [Å] and angles [°] of complex **4**.

Bi(1)-N(1)	2.525(3)	Bi(1)-N(2)	2.503(3)
Bi(1)-C(1)	2.189(3)	O(1)-C(7)	1.357(4)
O(1)-C(8)	1.457(4)	O(2)-C(12)	1.347(4)
O(2)-C(13)	1.457(4)	N(1)-C(7)	1.288(4)
N(1)-C(9)	1.482(4)	N(2)-C(12)	1.288(4)
N(2)-C(14)	1.484(4)	C(1)-C(2)	1.410(4)
C(1)-C(6)	1.410(4)	C(2)-C(3)	1.399(4)
C(2)-C(7)	1.446(4)	C(3)-C(4)	1.391(5)
C(4)-C(5)	1.395(5)	C(5)-C(6)	1.396(4)
C(6)-C(12)	1.453(4)	C(8)-C(9)	1.548(5)
C(9)-C(10)	1.519(5)	C(9)-C(11)	1.531(5)
C(13)-C(14)	1.550(5)	C(14)-C(15)	1.536(5)
C(14)-C(16)	1.520(5)	Bi(2)-N(3)	2.523(3)
Bi(2)-N(4)	2.502(3)	Bi(2)-C(21)	2.196(3)
O(3)-C(27)	1.357(4)	O(3)-C(28)	1.460(5)
O(4)-C(32)	1.354(4)	O(4)-C(33)	1.459(5)
N(3)-C(27)	1.287(5)	N(3)-C(29)	1.482(4)
N(4)-C(32)	1.291(4)	N(4)-C(34)	1.480(4)
C(21)-C(22)	1.413(4)	C(21)-C(26)	1.405(5)
C(22)-C(23)	1.400(5)	C(22)-C(27)	1.446(5)
C(23)-C(24)	1.391(6)	C(24)-C(25)	1.399(5)
C(25)-C(26)	1.399(5)	C(26)-C(32)	1.448(5)
C(28)-C(29)	1.541(5)	C(29)-C(30)	1.520(6)
C(29)-C(31)	1.523(6)	C(33)-C(34)	1.541(5)
C(34)-C(35)	1.516(6)	C(34)-C(36)	1.522(6)
N(2)-Bi(1)-N(1)	141.77(8)	C(1)-Bi(1)-N(1)	70.72(10)
C(1)-Bi(1)-N(2)	71.07(10)	C(7)-O(1)-C(8)	104.9(3)
C(12)-O(2)-C(13)	105.7(2)	C(7)-N(1)-Bi(1)	110.5(2)
C(7)-N(1)-C(9)	107.9(3)	C(9)-N(1)-Bi(1)	141.5(2)
C(12)-N(2)-Bi(1)	111.1(2)	C(12)-N(2)-C(14)	108.0(3)
C(14)-N(2)-Bi(1)	140.3(2)	C(2)-C(1)-Bi(1)	121.1(2)
C(2)-C(1)-C(6)	118.1(3)	C(6)-C(1)-Bi(1)	120.7(2)
C(1)-C(2)-C(7)	115.3(3)	C(3)-C(2)-C(1)	120.7(3)
C(3)-C(2)-C(7)	124.0(3)	C(4)-C(3)-C(2)	120.3(3)
C(3)-C(4)-C(5)	119.9(3)	C(4)-C(5)-C(6)	120.1(3)

C(1)-C(6)-C(12)	115.4(3)	C(5)-C(6)-C(1)	120.9(3)
C(5)-C(6)-C(12)	123.7(3)	O(1)-C(7)-C(2)	120.6(3)
N(1)-C(7)-O(1)	117.2(3)	N(1)-C(7)-C(2)	122.1(3)
O(1)-C(8)-C(9)	104.4(3)	N(1)-C(9)-C(8)	101.4(3)
N(1)-C(9)-C(10)	110.8(3)	N(1)-C(9)-C(11)	108.4(3)
C(10)-C(9)-C(8)	113.2(3)	C(10)-C(9)-C(11)	111.1(3)
C(11)-C(9)-C(8)	111.6(3)	O(2)-C(12)-C(6)	120.6(3)
N(2)-C(12)-O(2)	117.8(3)	N(2)-C(12)-C(6)	121.6(3)
O(2)-C(13)-C(14)	104.9(2)	N(2)-C(14)-C(13)	102.1(2)
N(2)-C(14)-C(15)	110.3(3)	N(2)-C(14)-C(16)	108.4(3)
C(15)-C(14)-C(13)	111.5(3)	C(16)-C(14)-C(13)	112.8(3)
C(16)-C(14)-C(15)	111.2(3)	N(4)-Bi(2)-N(3)	141.73(9)
C(21)-Bi(2)-N(3)	70.92(11)	C(21)-Bi(2)-N(4)	70.84(11)
C(27)-O(3)-C(28)	104.7(3)	C(32)-O(4)-C(33)	105.6(3)
C(27)-N(3)-Bi(2)	110.5(2)	C(27)-N(3)-C(29)	108.0(3)
C(29)-N(3)-Bi(2)	140.2(2)	C(32)-N(4)-Bi(2)	111.0(2)
C(32)-N(4)-C(34)	108.5(3)	C(34)-N(4)-Bi(2)	140.2(2)
C(22)-C(21)-Bi(2)	120.6(2)	C(26)-C(21)-Bi(2)	121.0(2)
C(26)-C(21)-C(22)	118.4(3)	C(21)-C(22)-C(27)	115.5(3)
C(23)-C(22)-C(21)	120.5(3)	C(23)-C(22)-C(27)	124.0(3)
C(24)-C(23)-C(22)	120.0(3)	C(23)-C(24)-C(25)	120.5(3)
C(24)-C(25)-C(26)	119.4(3)	C(21)-C(26)-C(32)	115.1(3)
C(25)-C(26)-C(21)	121.2(3)	C(25)-C(26)-C(32)	123.7(3)
O(3)-C(27)-C(22)	120.3(3)	N(3)-C(27)-O(3)	117.2(3)
N(3)-C(27)-C(22)	122.4(3)	O(3)-C(28)-C(29)	104.9(3)
N(3)-C(29)-C(28)	101.3(3)	N(3)-C(29)-C(30)	111.7(3)
N(3)-C(29)-C(31)	108.2(3)	C(30)-C(29)-C(28)	112.5(4)
C(30)-C(29)-C(31)	111.1(4)	C(31)-C(29)-C(28)	111.6(3)
O(4)-C(32)-C(26)	120.7(3)	N(4)-C(32)-O(4)	117.3(3)
N(4)-C(32)-C(26)	122.0(3)	O(4)-C(33)-C(34)	105.6(3)
N(4)-C(34)-C(33)	102.3(3)	N(4)-C(34)-C(35)	108.9(3)
N(4)-C(34)-C(36)	110.7(3)	C(35)-C(34)-C(33)	112.6(3)
C(35)-C(34)-C(36)	110.0(4)	C(36)-C(34)-C(33)	111.9(3)

5.2. Single Crystal Structure Analysis of Complex **5**

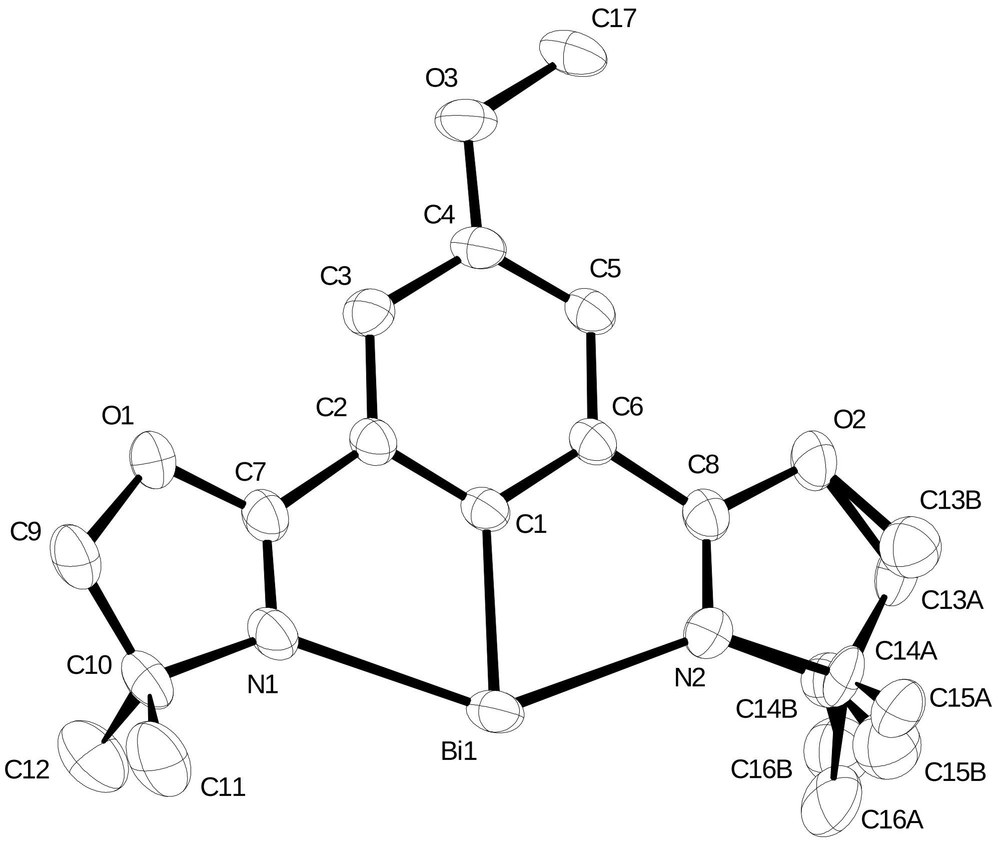

Figure S6. The molecular structure of complex **5**. H atoms have been removed for clarity.

X-ray Crystal Structure Analysis of complex 5: $C_{17} H_{21} Bi N_2 O_3$, M_r = 510.34 g mol^{-1}, dark-blue prism, crystal size 0.185 x 0.11 x 0.09 mm^3, monoclinic, *C*2/c [15], a = 20.6780(6) Å, b = 9.3278(6) Å, c = 19.0486(5) Å, β = 94.812(4)°, *V* = 3661.1(3) Å^3, *T* = 100(2) K, *Z* = 8, D_{calc} = 1.852 g·cm^3, λ = 0.71073 Å, μ(*Mo-K*$_\alpha$) = 9.646 mm^{-1}, Gaussian absorption correction (T_{min} = 0.25484, T_{max} = 0.49420), Bruker AXS Enraf-Nonius KappaCCD diffractometer with a FR591 rotating Mo-anode X-ray source, 2.660 < θ < 33.133°, 39748 measured reflections, 6971 independent reflections, 5709 reflections with $I > 2\sigma(I)$, R_{int} = 0.0354. The structure was solved by *SHELXT* and refined by full-matrix least-squares (*SHELXL*) against F^2 to R_1 = 0.0227 [$I > 2\sigma(I)$], wR_2 = 0.0429, 231 parameters.

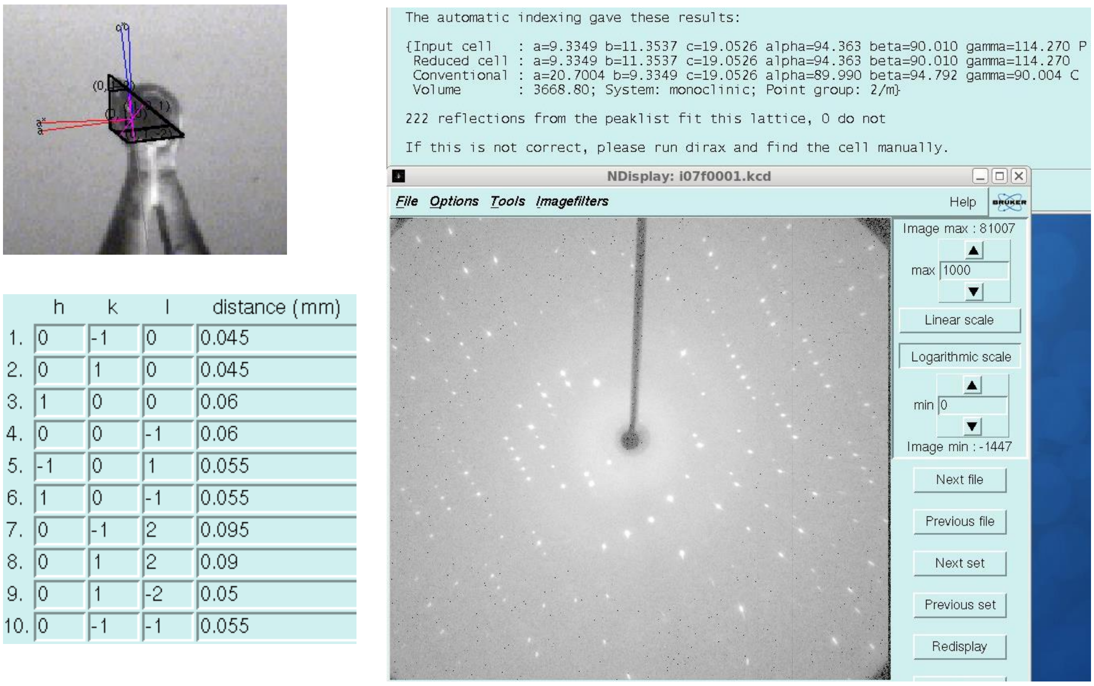

Figure S7. Crystal faces and unit cell determination of complex **5**.

INTENSITY STATISTICS FOR DATASET

Resolution	#Data	#Theory	%Complete	Redundancy	Mean I	Mean I/s	Rmerge	Rsigma
Inf - 2.67	111	123	90.2	8.95	207.11	73.04	0.0379	0.0143
2.67 - 1.79	260	260	100.0	8.35	174.01	67.93	0.0342	0.0120
1.79 - 1.42	361	361	100.0	7.73	115.36	61.39	0.0279	0.0126
1.42 - 1.24	366	366	100.0	7.36	73.37	52.43	0.0263	0.0138
1.24 - 1.12	380	380	100.0	7.05	62.89	48.75	0.0227	0.0151
1.12 - 1.04	354	354	100.0	6.82	46.40	42.41	0.0264	0.0166
1.04 - 0.97	425	425	100.0	6.45	33.38	35.75	0.0302	0.0195
0.97 - 0.92	369	369	100.0	6.15	27.69	31.75	0.0330	0.0222
0.92 - 0.88	373	373	100.0	5.93	23.92	28.43	0.0370	0.0245
0.88 - 0.85	327	327	100.0	5.61	20.45	25.65	0.0401	0.0285
0.85 - 0.82	373	374	99.7	5.42	14.62	20.84	0.0507	0.0344
0.82 - 0.79	414	415	99.8	5.21	13.00	18.64	0.0570	0.0394
0.79 - 0.77	336	336	100.0	4.92	10.58	15.77	0.0652	0.0478
0.77 - 0.75	355	355	100.0	4.75	9.67	14.14	0.0709	0.0539
0.75 - 0.73	406	408	99.5	4.47	9.19	13.15	0.0779	0.0596
0.73 - 0.71	422	424	99.5	4.30	7.89	11.25	0.0888	0.0689
0.71 - 0.70	262	264	99.2	4.28	7.12	10.35	0.0952	0.0757
0.70 - 0.68	518	520	99.6	4.07	5.15	7.98	0.1260	0.1032
0.68 - 0.67	275	278	98.9	3.74	4.54	7.00	0.1360	0.1215
0.67 - 0.66	323	323	100.0	3.91	4.17	6.43	0.1590	0.1314
0.66 - 0.65	314	324	96.9	3.61	2.86	4.84	0.2122	0.1908
0.75 - 0.65	2520	2541	99.2	4.08	5.99	8.91	0.1090	0.0907
Inf - 0.65	7324	7359	99.5	5.54	34.13	26.60	0.0350	0.0218

One low-angle reflection [-1 1 3] was likely affected by the beamstop and therefore removed from the data set before final refinement cycles. C13, C14, C15 and C16 are disordered over two positions with occupancy of 60:40. The minor disordered part is described by isotropic displacement parameters. Complete .cif-data of the compound are available under the CCDC number **CCDC-2091965**.

Table S7. Crystal data and structure refinement of complex **5**.

Identification code	13682	
Empirical formula	$C_{17} H_{21} Bi N_2 O_3$	
Color	dark blue / black	
Formula weight	510.34 $g \cdot mol^{-1}$	
Temperature	100(2) K	
Wavelength	0.71073 Å	
Crystal system	MONOCLINIC	
Space group	*C*2/c, (No. 15)	
Unit cell dimensions	a = 20.6780(6) Å	α= 90°.
	b = 9.3278(6) Å	β= 94.812(4)°.
	c = 19.0486(5) Å	γ = 90°.
Volume	3661.1(3) $Å^3$	
Z	8	
Density (calculated)	1.852 $Mg \cdot m^{-3}$	
Absorption coefficient	9.646 mm^{-1}	
F(000)	1952 e	
Crystal size	0.185 x 0.11 x 0.09 mm^3	
θ range for data collection	2.660 to 33.133°.	
Index ranges	$-31 \leq h \leq 31, -14 \leq k \leq 14, -29 \leq l \leq 29$	
Reflections collected	39748	
Independent reflections	6971 [R_{int} = 0.0354]	
Reflections with $I>2\sigma(I)$	5709	
Completeness to θ = 25.242°	99.7 %	
Absorption correction	Gaussian	
Max. and min. transmission	0.49 and 0.25	
Refinement method	Full-matrix least-squares on F^2	
Data / restraints / parameters	6971 / 0 / 231	
Goodness-of-fit on F^2	1.083	
Final R indices [$I>2\sigma(I)$]	R_1 = 0.0227	wR^2 = 0.0429
R indices (all data)	R_1 = 0.0344	wR^2 = 0.0468
Largest diff. peak and hole	0.4 and -1.1 $e \cdot Å^{-3}$	

Table S8. Bond lengths [Å] and angles [°] of complex **5**.

Bi(1)-N(1)	2.5359(19)	Bi(1)-N(2)	2.5142(18)
Bi(1)-C(1)	2.201(2)	O(1)-C(7)	1.355(3)
O(1)-C(9)	1.455(3)	O(2)-C(8)	1.352(2)
O(2)-C(13A)	1.520(5)	O(2)-C(13B)	1.395(9)
O(3)-C(4)	1.368(2)	O(3)-C(17)	1.415(3)
N(1)-C(7)	1.282(3)	N(1)-C(10)	1.480(3)
N(2)-C(8)	1.284(3)	N(2)-C(14A)	1.535(10)
N(2)-C(14B)	1.401(17)	C(1)-C(2)	1.405(3)
C(1)-C(6)	1.403(3)	C(2)-C(3)	1.390(3)
C(2)-C(7)	1.455(3)	C(3)-C(4)	1.394(3)
C(4)-C(5)	1.386(3)	C(5)-C(6)	1.401(3)
C(6)-C(8)	1.446(3)	C(9)-C(10)	1.540(4)
C(10)-C(11)	1.519(4)	C(10)-C(12)	1.518(4)
C(13A)-C(14A)	1.437(15)	C(13B)-C(14B)	1.68(2)
C(14A)-C(15A)	1.550(15)	C(14A)-C(16A)	1.571(16)
C(14B)-C(15B)	1.45(2)	C(14B)-C(16B)	1.52(2)

N(2)-Bi(1)-N(1)	140.66(6)	C(1)-Bi(1)-N(1)	70.06(7)
C(1)-Bi(1)-N(2)	70.60(7)	C(7)-O(1)-C(9)	104.68(18)
C(8)-O(2)-C(13A)	102.8(2)	C(8)-O(2)-C(13B)	107.7(4)
C(4)-O(3)-C(17)	116.88(19)	C(7)-N(1)-Bi(1)	111.13(14)
C(7)-N(1)-C(10)	108.20(19)	C(10)-N(1)-Bi(1)	140.66(15)
C(8)-N(2)-Bi(1)	110.99(14)	C(8)-N(2)-C(14A)	106.7(6)
C(8)-N(2)-C(14B)	109.8(9)	C(14A)-N(2)-Bi(1)	142.3(6)
C(14B)-N(2)-Bi(1)	139.1(9)	C(2)-C(1)-Bi(1)	121.91(14)
C(6)-C(1)-Bi(1)	120.83(15)	C(6)-C(1)-C(2)	117.22(18)
C(1)-C(2)-C(7)	114.93(18)	C(3)-C(2)-C(1)	121.69(18)
C(3)-C(2)-C(7)	123.33(19)	C(2)-C(3)-C(4)	119.7(2)
O(3)-C(4)-C(3)	115.70(19)	O(3)-C(4)-C(5)	124.03(18)
C(5)-C(4)-C(3)	120.27(18)	C(4)-C(5)-C(6)	119.43(18)
C(1)-C(6)-C(8)	115.44(18)	C(5)-C(6)-C(1)	121.66(19)
C(5)-C(6)-C(8)	122.89(18)	O(1)-C(7)-C(2)	120.25(19)
N(1)-C(7)-O(1)	117.75(19)	N(1)-C(7)-C(2)	122.0(2)
O(2)-C(8)-C(6)	120.52(18)	N(2)-C(8)-O(2)	117.39(19)

N(2)-C(8)-C(6)	122.07(18)	O(1)-C(9)-C(10)	105.54(18)
N(1)-C(10)-C(9)	101.67(17)	N(1)-C(10)-C(11)	109.7(2)
N(1)-C(10)-C(12)	108.8(2)	C(11)-C(10)-C(9)	113.2(2)
C(12)-C(10)-C(9)	112.3(2)	C(12)-C(10)-C(11)	110.6(2)
C(14A)-C(13A)-O(2)	106.1(5)	O(2)-C(13B)-C(14B)	101.2(8)
N(2)-C(14A)-C(15A)	106.1(8)	N(2)-C(14A)-C(16A)	105.0(8)
C(13A)-C(14A)-N(2)	102.5(8)	C(13A)-C(14A)-C(15A)	117.9(10)
C(13A)-C(14A)-C(16A)	115.7(10)	C(15A)-C(14A)-C(16A)	108.2(9)
N(2)-C(14B)-C(13B)	101.1(13)	N(2)-C(14B)-C(15B)	114.9(13)
N(2)-C(14B)-C(16B)	113.8(13)	C(15B)-C(14B)-C(13B)	106.0(13)
C(15B)-C(14B)-C(16B)	113.3(14)	C(16B)-C(14B)-C(13B)	106.2(11)

5.3. Single Crystal Structure Analysis of Complex **6**

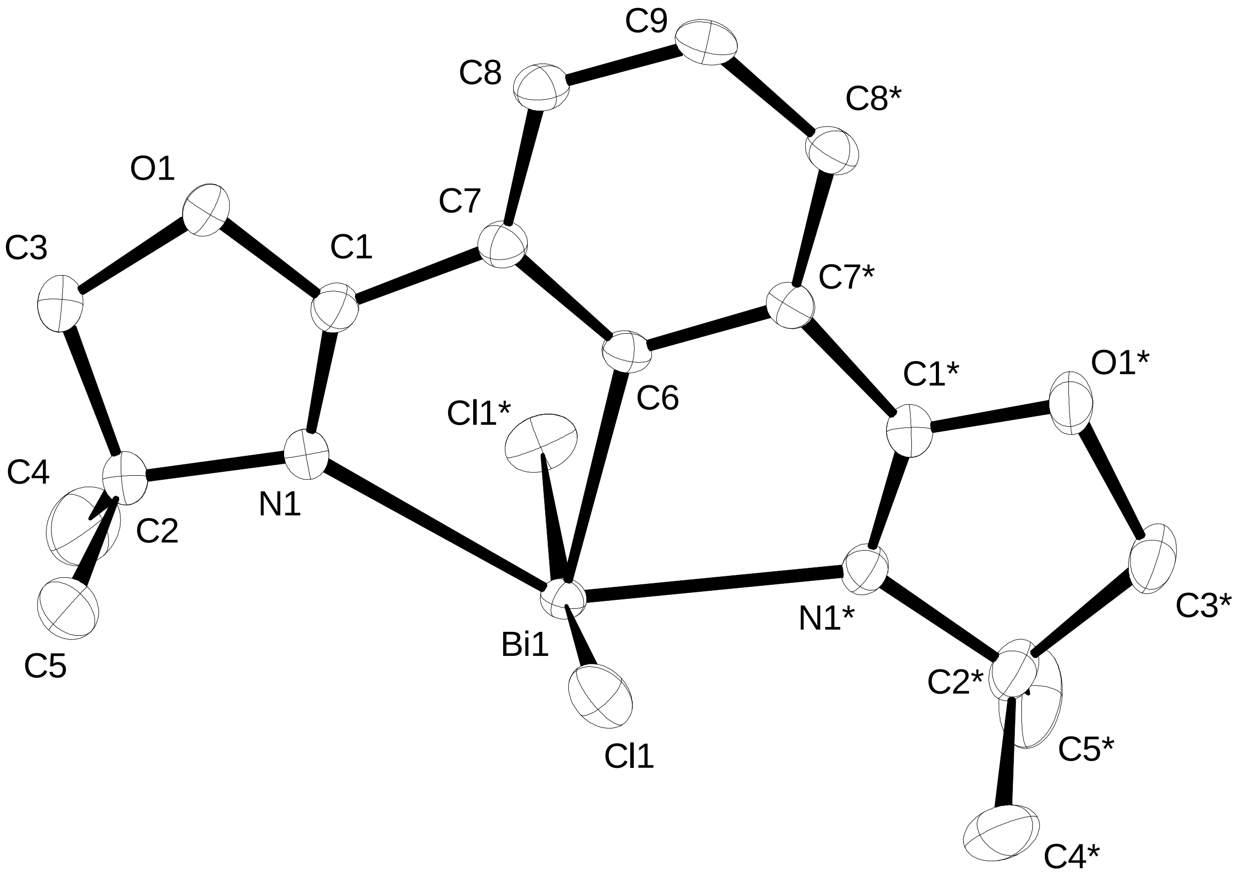

Figure S8. The molecular structure of complex **6**. H atoms have been removed for clarity.

X-ray Crystal Structure Analysis of complex 6: $C_{16}H_{19}BiCl_2N_2O_2$, M_r = 551.21 g mol^{-1}, colourless prism, crystal size 0.11 x 0.09 x 0.05 mm^3, monoclinic, *C*2/c [15], a = 18.871(4) Å, b = 11.829(2) Å, c = 8.9135(18) Å, β= 115.10(3)°, *V* = 1801.8(7) Å^3, *T* = 100(2) K, *Z* = 4, D_{calc} = 2.032 g·cm^3, λ = 0.71073 Å, $\mu(Mo\text{-}K_\alpha)$= 10.091 mm^{-1}, Gaussian absorption correction (T_{min} = 0.34379, T_{max} = 0.61896), Bruker AXS Enraf-Nonius KappaCCD diffractometer with a FR591 rotating Mo-anode X-ray source, 2.864 < θ < 30.508°, 17679 measured reflections, 2753 independent reflections, 2702 reflections with $I > 2\sigma(I)$, R_{int} = 0.0278]. The structure was solved by *SHELXT* and refined by full-matrix least-squares (*SHELXL*) against F^2 to R_1 = 0.0145 [$I > 2\sigma(I)$], wR_2 = 0.0358, 108 parameters.

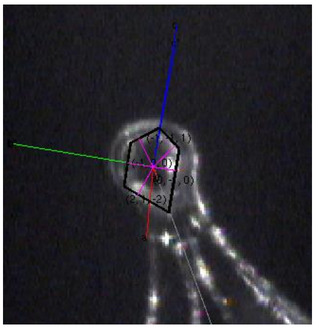

	h	k	l	distance (mm)
1.	0	1	0	0.045
2.	0	-1	0	0.04
3.	-1	1	1	0.055
4.	-1	-1	1	0.055
5.	-1	0	0	0.025
6.	1	0	0	0.025
7.	2	1	-2	0.055

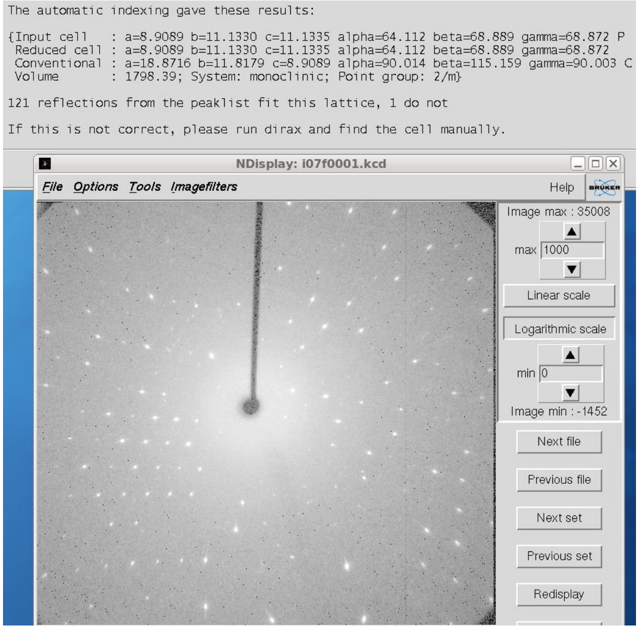

Figure S9. Crystal faces and unit cell determination of complex **6**.

INTENSITY STATISTICS FOR DATASET

Resolution	#Data	#Theory	%Complete	Redundancy	Mean I	Mean I/s	Rmerge	Rsigma
Inf - 2.35	277	287	96.5	12.79	127.89	90.95	0.0346	0.0105
2.35 - 1.56	660	660	100.0	15.94	86.09	93.22	0.0304	0.0092
1.56 - 1.24	927	927	100.0	15.47	58.34	79.14	0.0323	0.0104
1.24 - 1.08	952	952	100.0	14.01	44.18	66.51	0.0321	0.0123
1.08 - 0.98	945	945	100.0	12.81	34.76	55.86	0.0356	0.0148
0.98 - 0.91	946	946	100.0	11.86	29.08	47.47	0.0396	0.0172
0.91 - 0.86	851	851	100.0	11.12	24.70	41.92	0.0454	0.0200
0.86 - 0.81	1104	1104	100.0	10.43	19.84	35.04	0.0543	0.0241
0.81 - 0.78	795	795	100.0	9.89	17.83	30.18	0.0603	0.0273
0.78 - 0.75	911	911	100.0	9.43	15.24	26.55	0.0691	0.0319
0.75 - 0.72	1114	1114	100.0	8.21	15.05	23.85	0.0748	0.0358
0.72 - 0.70	809	809	100.0	6.82	12.60	19.03	0.0876	0.0455
0.70 - 0.68	959	959	100.0	6.59	11.14	16.79	0.0974	0.0519
0.68 - 0.66	1032	1032	100.0	6.27	9.34	14.09	0.1153	0.0626
0.66 - 0.64	1214	1214	100.0	5.95	8.42	12.20	0.1331	0.0742
0.64 - 0.63	638	638	100.0	5.72	8.25	11.31	0.1463	0.0803
0.63 - 0.62	693	693	100.0	5.66	7.62	10.24	0.1511	0.0892
0.62 - 0.60	1544	1544	100.0	5.48	6.84	8.75	0.1639	0.1073
0.60 - 0.59	823	823	100.0	5.17	5.86	6.79	0.1997	0.1448
0.59 - 0.58	1269	1273	99.7	4.99	5.16	5.25	0.2310	0.1920
0.68 - 0.58	7213	7217	99.9	5.59	7.26	9.62	0.1549	0.1021
Inf - 0.58	18463	18477	99.9	8.87	22.19	30.93	0.0471	0.0285

A resolution cut off (SHEL 0.7 55) was applied to dataset to exclude poorly measured reflections at high diffraction angles. Complete .cif-data of the compound are available under the CCDC number **CCDC-2091964**.

Table S9. Crystal data and structure refinement of complex **6**.

Identification code	13499	
Empirical formula	$C_{16}H_{19}BiCl_2N_2O_2$	
Color	colourless	
Formula weight	551.21 g · mol^{-1}	
Temperature	100(2) K	
Wavelength	0.71073 Å	
Crystal system	monoclinic	
Space group	*C*2/c, (No. 15)	
Unit cell dimensions	a = 18.871(4) Å	α= 90°.
	b = 11.829(2) Å	β= 115.10(3)°.
	c = 8.9135(18) Å	γ = 90°.
Volume	1801.8(7) $Å^3$	
Z	4	
Density (calculated)	2.032 Mg · m^{-3}	
Absorption coefficient	10.091 mm^{-1}	
F(000)	1048 e	
Crystal size	0.11 x 0.09 x 0.05 mm^3	
θ range for data collection	2.864 to 30.508°.	
Index ranges	$-26 \leq h \leq 26$, $-16 \leq k \leq 16$, $-12 \leq l \leq 12$	
Reflections collected	17679	
Independent reflections	2753 [R_{int} = 0.0278]	
Reflections with I>2σ(I)	2702	
Completeness to θ = 25.242°	99.7 %	
Absorption correction	Gaussian	
Max. and min. transmission	0.62 and 0.34	
Refinement method	Full-matrix least-squares on F^2	
Data / restraints / parameters	2753 / 0 / 108	
Goodness-of-fit on F^2	1.171	
Final R indices [I>2σ(I)]	R_1 = 0.0145	wR^2 = 0.0358
R indices (all data)	R_1 = 0.0151	wR^2 = 0.0361
Largest diff. peak and hole	0.6 and -1.4 e · $Å^{-3}$	

Table S10. Bond lengths [Å] and angles [°] of complex **6**.

Bi(1)-Cl(1)	2.6716(8)	Bi(1)-Cl(1)*	2.6715(8)
Bi(1)-N(1)	2.4632(19)	Bi(1)-N(1)*	2.4633(19)
Bi(1)-C(6)	2.215(3)	O(1)-C(1)	1.338(2)
O(1)-C(3)	1.471(3)	N(1)-C(1)	1.281(2)
N(1)-C(2)	1.489(3)	C(1)-C(7)	1.469(3)
C(2)-C(3)	1.553(3)	C(2)-C(4)	1.530(4)
C(2)-C(5)	1.525(4)	C(6)-C(7)	1.388(2)
C(6)-C(7)*	1.388(2)	C(7)-C(8)	1.398(3)
C(8)-C(9)	1.397(2)		
Cl(1)*-Bi(1)-Cl(1)	168.83(2)	N(1)*-Bi(1)-Cl(1)	85.71(4)
N(1)-Bi(1)-Cl(1)*	85.70(5)	N(1)-Bi(1)-Cl(1)	90.77(5)
N(1)*-Bi(1)-Cl(1)*	90.77(4)	N(1)-Bi(1)-N(1)*	143.17(8)
C(6)-Bi(1)-Cl(1)	84.413(12)	C(6)-Bi(1)-Cl(1)*	84.412(12)
C(6)-Bi(1)-N(1)	71.58(4)	C(6)-Bi(1)-N(1)*	71.58(4)
C(1)-O(1)-C(3)	105.57(16)	C(1)-N(1)-Bi(1)	111.37(13)
C(1)-N(1)-C(2)	108.92(17)	C(2)-N(1)-Bi(1)	139.32(13)
O(1)-C(1)-C(7)	119.75(17)	N(1)-C(1)-O(1)	118.07(17)
N(1)-C(1)-C(7)	122.17(17)	N(1)-C(2)-C(3)	101.50(16)
N(1)-C(2)-C(4)	108.9(2)	N(1)-C(2)-C(5)	110.06(19)
C(4)-C(2)-C(3)	111.5(2)	C(5)-C(2)-C(3)	112.5(2)
C(5)-C(2)-C(4)	111.9(2)	O(1)-C(3)-C(2)	105.30(16)
C(7)*-C(6)-Bi(1)	119.77(12)	C(7)-C(6)-Bi(1)	119.77(12)
C(7)-C(6)-C(7)*	120.5(2)	C(6)-C(7)-C(1)	114.98(17)
C(6)-C(7)-C(8)	120.21(19)	C(8)-C(7)-C(1)	124.80(17)
C(9)-C(8)-C(7)	119.07(19)	C(8)*-C(9)-C(8)	121.0(2)

Symmetry transformations used to generate equivalent atoms: * -x+1,y,-z+1/2

5.4. Single Crystal Structure Analysis of Complex **7 · 0.5 THF**

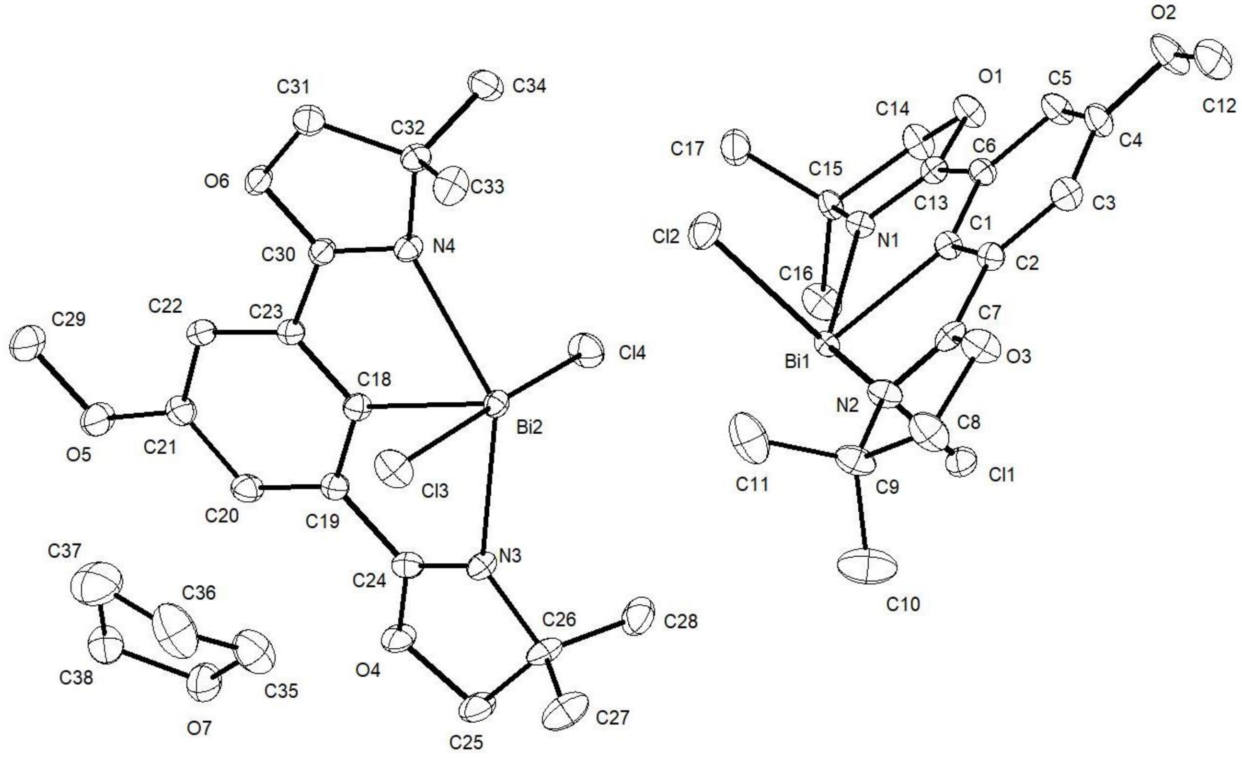

Figure S10. The molecular structure of complex **7 · 0.5 THF**. H atoms have been removed for clarity.

X-ray Crystal Structure Analysis of complex 7 · 0.5 THF: $C_{38}H_{50}Bi_2Cl_4N_4O_7$, M_r = 1234.58 g mol^{-1}, colourless prism, crystal size 0.10 x 0.05 x 0.05 mm^3, monoclinic, $P2_1/n$ [14], a = 8.6838(11) Å, b = 29.799(3) Å, c = 17.143(2) Å, β= 90.404(9)°, V = 4435.8(9) Å^3, T = 100(2) K, Z = 4, D_{calc} = 1.849 g·cm^3, λ = 0.71073 Å, μ(*Mo-K*$_\alpha$)= 8.214 mm^{-1}, Gaussian absorption correction ($T_{\min}$ = 0.51762, $T_{\max}$ = 0.69300), Bruker AXS Enraf-Nonius KappaCCD diffractometer with a FR591 rotating Mo-anode X-ray source, 2.622 < θ < 32.032°, 88449 measured reflections, 15427 independent reflections, 11397 reflections with $I > 2\sigma(I)$, R_{int} = 0.0536]. The structure was solved by *SHELXT* and refined by full-matrix least-squares (*SHELXL*) against F^2 to R_1 = 0.0297 [$I > 2\sigma(I)$], wR_2 = 0.0525, 506 parameters.

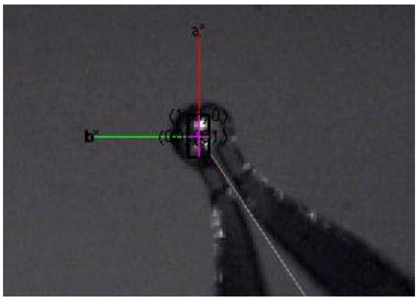

	h	k	l	distance (mm)
1.	0	0	1	0.025
2.	0	0	-1	0.025
3.	0	-1	0	0.025
4.	0	1	0	0.025
5.	1	0	0	0.05
6.	-1	0	0	0.05

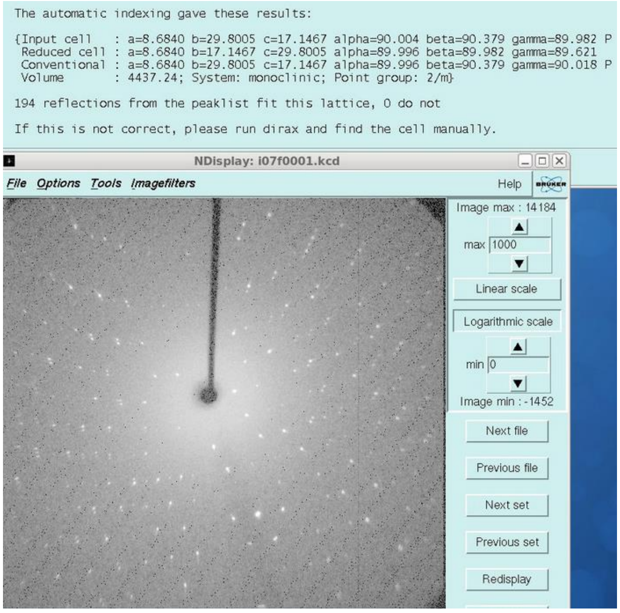

Figure S11. Crystal faces and unit cell determination of complex **7 · 0.5 THF**.

INTENSITY STATISTICS FOR DATASET

Resolution	#Data	#Theory	%Complete	Redundancy	Mean I	Mean I/s	Rmerge	Rsigma
Inf - 2.67	262	273	96.0	11.46	95.75	59.76	0.0359	0.0113
2.67 - 1.77	606	606	100.0	8.59	77.53	42.29	0.0337	0.0142
1.77 - 1.40	893	893	100.0	7.80	50.17	33.77	0.0356	0.0172
1.40 - 1.22	872	872	100.0	7.33	40.31	28.84	0.0386	0.0195
1.22 - 1.11	846	846	100.0	6.92	36.13	26.93	0.0382	0.0223
1.11 - 1.03	906	906	100.0	6.63	31.00	23.67	0.0421	0.0251
1.03 - 0.97	817	817	100.0	6.29	23.79	19.54	0.0492	0.0304
0.97 - 0.92	914	914	100.0	5.98	21.72	17.65	0.0549	0.0342
0.92 - 0.88	848	848	100.0	5.76	19.14	15.59	0.0613	0.0390
0.88 - 0.85	782	782	100.0	5.49	19.04	15.38	0.0636	0.0419
0.85 - 0.82	865	865	100.0	5.29	15.43	12.75	0.0740	0.0499
0.82 - 0.79	986	986	100.0	5.07	15.10	12.58	0.0785	0.0541
0.79 - 0.77	780	780	100.0	4.78	12.65	10.03	0.0910	0.0653
0.77 - 0.75	865	865	100.0	4.65	12.02	9.72	0.1011	0.0714
0.75 - 0.73	927	927	100.0	4.54	11.78	9.30	0.1080	0.0768
0.73 - 0.71	1051	1051	100.0	4.28	9.94	7.69	0.1208	0.0947
0.71 - 0.70	586	586	100.0	4.17	8.90	7.10	0.1409	0.1082
0.70 - 0.68	1230	1230	100.0	4.07	8.44	6.54	0.1458	0.1192
0.68 - 0.67	666	666	100.0	3.92	6.90	5.43	0.1752	0.1566
0.67 - 0.66	710	710	100.0	3.75	7.05	5.00	0.1762	0.1689
0.66 - 0.65	757	766	98.8	3.63	6.91	4.81	0.1892	0.1888
0.75 - 0.65	5927	5936	99.8	4.08	8.74	6.70	0.1406	0.1194
Inf - 0.65	17169	17189	99.9	5.52	22.21	16.17	0.0536	0.0403

A resolution cut off (SHEL 99 0.67) was applied to dataset to exclude poorly measured reflections at high diffraction angles. Complete .cif-data of the compound are available under the CCDC number **CCDC-2091967**.

Table S11. Crystal data and structure refinement of complex **7 · 0.5 THF**.

Identification code	13899	
Empirical formula	$C_{38}H_{50}Bi_2Cl_4N_4O_7$	
Color	colourless	
Formula weight	1234.58 g·mol^{-1}	
Temperature	100(2) K	
Wavelength	0.71073 Å	
Crystal system	Monoclinic	
Space group	$P2_1$/n, (No. 14)	
Unit cell dimensions	a = 8.6838(11) Å	α= 90°.
	b = 29.799(3) Å	β= 90.404(9)°.
	c = 17.143(2) Å	γ = 90°.
Volume	4435.8(9) Å^3	
Z	4	
Density (calculated)	1.849 Mg·m^{-3}	
Absorption coefficient	8.214 mm^{-1}	
F(000)	2384 e	
Crystal size	0.10 x 0.05 x 0.05 mm^3	
θ range for data collection	2.622 to 32.032°.	
Index ranges	-12 ≤ h ≤ 12, -42≤ k ≤ 44, -25 ≤ l ≤ 25	
Reflections collected	88449	
Independent reflections	15427 [R_{int} = 0.0536]	
Reflections with I>2σ(I)	11397	
Completeness to θ = 25.242°	99.9 %	
Absorption correction	Gaussian	
Max. and min. transmission	0.69300 and 0.51762	
Refinement method	Full-matrix least-squares on F^2	
Data / restraints / parameters	15427 / 0 / 506	
Goodness-of-fit on F^2	1.056	
Final R indices [I>2σ(I)]	R_1 = 0.0297	wR^2 = 0.0525
R indices (all data)	R_1 = 0.0534	wR^2 = 0.0586
Extinction coefficient	n/a	
Largest diff. peak and hole	0.924 and -1.221 e·Å^{-3}	

Table S12. Bond lengths [Å] and angles [°] of complex **7 · 0.5 THF**.

Bi(2)-Cl(4)	2.6578(8)	Bi(2)-Cl(3)	2.6775(8)
Bi(2)-C(18)	2.219(3)	Bi(2)-N(4)	2.469(3)
Bi(2)-N(3)	2.532(3)	Bi(1)-Cl(1)	2.6739(8)
Bi(1)-Cl(2)	2.6561(8)	Bi(1)-N(1)	2.469(2)
Bi(1)-N(2)	2.507(3)	Bi(1)-C(1)	2.227(3)
O(6)-C(30)	1.340(4)	O(6)-C(31)	1.477(4)
O(4)-C(24)	1.336(4)	O(4)-C(25)	1.464(4)
O(1)-C(13)	1.340(3)	O(1)-C(14)	1.467(4)
O(2)-C(4)	1.357(4)	O(2)-C(12)	1.430(4)
N(1)-C(13)	1.281(4)	N(1)-C(15)	1.489(4)
O(3)-C(8)	1.465(4)	O(3)-C(7)	1.343(4)
C(23)-C(18)	1.380(4)	C(23)-C(30)	1.470(4)
C(23)-C(22)	1.397(4)	O(5)-C(21)	1.360(4)
O(5)-C(29)	1.425(4)	O(7)-C(35)	1.427(5)
O(7)-C(38)	1.433(4)	C(17)-H(17A)	0.9800
C(17)-H(17B)	0.9800	C(17)-H(17C)	0.9800
C(17)-C(15)	1.513(5)	C(18)-C(19)	1.397(4)
N(4)-C(30)	1.279(4)	N(4)-C(32)	1.492(4)
N(3)-C(24)	1.280(4)	N(3)-C(26)	1.486(4)
C(13)-C(6)	1.473(4)	N(2)-C(9)	1.488(4)
N(2)-C(7)	1.275(4)	C(3)-H(3)	0.9500
C(3)-C(4)	1.393(4)	C(3)-C(2)	1.401(4)
C(8)-H(8A)	0.9900	C(8)-H(8B)	0.9900
C(8)-C(9)	1.545(5)	C(34)-H(34A)	0.9800
C(34)-H(34B)	0.9800	C(34)-H(34C)	0.9800
C(34)-C(32)	1.524(5)	C(1)-C(6)	1.389(4)
C(1)-C(2)	1.392(4)	C(6)-C(5)	1.390(4)
C(9)-C(10)	1.519(5)	C(9)-C(11)	1.524(5)
C(21)-C(22)	1.398(4)	C(21)-C(20)	1.398(5)
C(31)-H(31A)	0.9900	C(31)-H(31B)	0.9900
C(31)-C(32)	1.544(4)	C(22)-H(22)	0.9500
C(4)-C(5)	1.402(4)	C(12)-H(12A)	0.9800
C(12)-H(12B)	0.9800	C(12)-H(12C)	0.9800
C(32)-C(33)	1.523(4)	C(5)-H(5)	0.9500
C(7)-C(2)	1.466(4)	C(15)-C(16)	1.523(4)
C(15)-C(14)	1.551(4)	C(10)-H(10A)	0.9800
C(10)-H(10B)	0.9800	C(10)-H(10C)	0.9800

C(19)-C(24)	1.462(4)	C(19)-C(20)	1.392(4)
C(16)-H(16A)	0.9800	C(16)-H(16B)	0.9800
C(16)-H(16C)	0.9800	C(28)-H(28A)	0.9800
C(28)-H(28B)	0.9800	C(28)-H(28C)	0.9800
C(28)-C(26)	1.521(5)	C(36)-H(36A)	0.9900
C(36)-H(36B)	0.9900	C(36)-C(35)	1.481(6)
C(36)-C(37)	1.541(7)	C(35)-H(35A)	0.9900
C(35)-H(35B)	0.9900	C(26)-C(27)	1.522(5)
C(26)-C(25)	1.549(5)	C(27)-H(27A)	0.9800
C(27)-H(27B)	0.9800	C(27)-H(27C)	0.9800
C(33)-H(33A)	0.9800	C(33)-H(33B)	0.9800
C(33)-H(33C)	0.9800	C(14)-H(14A)	0.9900
C(14)-H(14B)	0.9900	C(20)-H(20)	0.9500
C(25)-H(25A)	0.9900	C(25)-H(25B)	0.9900
C(37)-H(37A)	0.9900	C(37)-H(37B)	0.9900
C(37)-C(38)	1.521(6)	C(11)-H(11A)	0.9800
C(11)-H(11B)	0.9800	C(11)-H(11C)	0.9800
C(38)-H(38A)	0.9900	C(38)-H(38B)	0.9900
C(29)-H(29A)	0.9800	C(29)-H(29B)	0.9800
C(29)-H(29C)	0.9800		

Cl(4)-Bi(2)-Cl(3)	174.28(3)	C(18)-Bi(2)-Cl(4)	87.33(8)
C(18)-Bi(2)-Cl(3)	87.99(8)	C(18)-Bi(2)-N(4)	71.21(10)
C(18)-Bi(2)-N(3)	70.35(10)	N(4)-Bi(2)-Cl(4)	95.57(6)
N(4)-Bi(2)-Cl(3)	86.02(6)	N(4)-Bi(2)-N(3)	141.56(8)
N(3)-Bi(2)-Cl(4)	82.72(6)	N(3)-Bi(2)-Cl(3)	92.60(6)
Cl(2)-Bi(1)-Cl(1)	172.48(3)	N(1)-Bi(1)-Cl(1)	88.87(6)
N(1)-Bi(1)-Cl(2)	96.27(6)	N(1)-Bi(1)-N(2)	141.70(9)
N(2)-Bi(1)-Cl(1)	89.17(7)	N(2)-Bi(1)-Cl(2)	83.40(7)
C(1)-Bi(1)-Cl(1)	88.73(8)	C(1)-Bi(1)-Cl(2)	87.77(8)
C(1)-Bi(1)-N(1)	70.99(9)	C(1)-Bi(1)-N(2)	70.73(10)
C(30)-O(6)-C(31)	105.2(2)	C(24)-O(4)-C(25)	105.2(2)
C(13)-O(1)-C(14)	104.9(2)	C(4)-O(2)-C(12)	118.0(3)
C(13)-N(1)-Bi(1)	111.66(19)	C(13)-N(1)-C(15)	108.3(2)
C(15)-N(1)-Bi(1)	139.23(19)	C(7)-O(3)-C(8)	105.2(3)
C(18)-C(23)-C(30)	114.8(3)	C(18)-C(23)-C(22)	121.6(3)
C(22)-C(23)-C(30)	123.6(3)	C(21)-O(5)-C(29)	116.7(3)
C(35)-O(7)-C(38)	109.6(3)	H(17A)-C(17)-H(17B)	109.5

H(17A)-C(17)-H(17C)	109.5	H(17B)-C(17)-H(17C)	109.5
C(15)-C(17)-H(17A)	109.5	C(15)-C(17)-H(17B)	109.5
C(15)-C(17)-H(17C)	109.5	C(23)-C(18)-Bi(2)	120.0(2)
C(23)-C(18)-C(19)	119.1(3)	C(19)-C(18)-Bi(2)	120.9(2)
C(30)-N(4)-Bi(2)	110.9(2)	C(30)-N(4)-C(32)	108.3(3)
C(32)-N(4)-Bi(2)	139.34(19)	C(24)-N(3)-Bi(2)	110.6(2)
C(24)-N(3)-C(26)	108.4(3)	C(26)-N(3)-Bi(2)	138.9(2)
O(1)-C(13)-C(6)	119.6(3)	N(1)-C(13)-O(1)	118.1(3)
N(1)-C(13)-C(6)	122.3(3)	C(9)-N(2)-Bi(1)	139.3(2)
C(7)-N(2)-Bi(1)	111.1(2)	C(7)-N(2)-C(9)	108.4(3)
C(4)-C(3)-H(3)	120.5	C(4)-C(3)-C(2)	119.0(3)
C(2)-C(3)-H(3)	120.5	O(6)-C(30)-C(23)	119.6(3)
N(4)-C(30)-O(6)	118.4(3)	N(4)-C(30)-C(23)	122.1(3)
O(3)-C(8)-H(8A)	110.6	O(3)-C(8)-H(8B)	110.6
O(3)-C(8)-C(9)	105.6(2)	H(8A)-C(8)-H(8B)	108.8
C(9)-C(8)-H(8A)	110.6	C(9)-C(8)-H(8B)	110.6
H(34A)-C(34)-H(34B)	109.5	H(34A)-C(34)-H(34C)	109.5
H(34B)-C(34)-H(34C)	109.5	C(32)-C(34)-H(34A)	109.5
C(32)-C(34)-H(34B)	109.5	C(32)-C(34)-H(34C)	109.5
C(6)-C(1)-Bi(1)	120.6(2)	C(6)-C(1)-C(2)	119.1(3)
C(2)-C(1)-Bi(1)	120.3(2)	C(1)-C(6)-C(13)	114.0(3)
C(1)-C(6)-C(5)	121.4(3)	C(5)-C(6)-C(13)	124.6(3)
N(2)-C(9)-C(8)	101.7(3)	N(2)-C(9)-C(10)	109.7(3)
N(2)-C(9)-C(11)	109.2(3)	C(10)-C(9)-C(8)	112.3(3)
C(10)-C(9)-C(11)	111.4(3)	C(11)-C(9)-C(8)	112.0(3)
O(5)-C(21)-C(22)	124.4(3)	O(5)-C(21)-C(20)	115.0(3)
C(22)-C(21)-C(20)	120.6(3)	O(6)-C(31)-H(31A)	110.8
O(6)-C(31)-H(31B)	110.8	O(6)-C(31)-C(32)	105.0(2)
H(31A)-C(31)-H(31B)	108.8	C(32)-C(31)-H(31A)	110.8
C(32)-C(31)-H(31B)	110.8	C(23)-C(22)-C(21)	118.6(3)
C(23)-C(22)-H(22)	120.7	C(21)-C(22)-H(22)	120.7
O(2)-C(4)-C(3)	124.2(3)	O(2)-C(4)-C(5)	115.1(3)
C(3)-C(4)-C(5)	120.8(3)	O(2)-C(12)-H(12A)	109.5
O(2)-C(12)-H(12B)	109.5	O(2)-C(12)-H(12C)	109.5
H(12A)-C(12)-H(12B)	109.5	H(12A)-C(12)-H(12C)	109.5
H(12B)-C(12)-H(12C)	109.5	N(4)-C(32)-C(34)	109.6(3)
N(4)-C(32)-C(31)	101.9(2)	N(4)-C(32)-C(33)	108.8(3)
C(34)-C(32)-C(31)	111.4(3)	C(33)-C(32)-C(34)	111.8(3)

C(33)-C(32)-C(31)	112.7(3)	C(6)-C(5)-C(4)	118.9(3)
C(6)-C(5)-H(5)	120.6	C(4)-C(5)-H(5)	120.6
O(3)-C(7)-C(2)	119.1(3)	N(2)-C(7)-O(3)	118.5(3)
N(2)-C(7)-C(2)	122.4(3)	N(1)-C(15)-C(17)	109.7(3)
N(1)-C(15)-C(16)	109.0(2)	N(1)-C(15)-C(14)	101.2(2)
C(17)-C(15)-C(16)	112.2(3)	C(17)-C(15)-C(14)	111.4(3)
C(16)-C(15)-C(14)	112.7(3)	C(9)-C(10)-H(10A)	109.5
C(9)-C(10)-H(10B)	109.5	C(9)-C(10)-H(10C)	109.5
H(10A)-C(10)-H(10B)	109.5	H(10A)-C(10)-H(10C)	109.5
H(10B)-C(10)-H(10C)	109.5	C(18)-C(19)-C(24)	115.2(3)
C(20)-C(19)-C(18)	120.7(3)	C(20)-C(19)-C(24)	124.0(3)
C(3)-C(2)-C(7)	124.0(3)	C(1)-C(2)-C(3)	120.8(3)
C(1)-C(2)-C(7)	115.2(3)	C(15)-C(16)-H(16A)	109.5
C(15)-C(16)-H(16B)	109.5	C(15)-C(16)-H(16C)	109.5
H(16A)-C(16)-H(16B)	109.5	H(16A)-C(16)-H(16C)	109.5
H(16B)-C(16)-H(16C)	109.5	H(28A)-C(28)-H(28B)	109.5
H(28A)-C(28)-H(28C)	109.5	H(28B)-C(28)-H(28C)	109.5
C(26)-C(28)-H(28A)	109.5	C(26)-C(28)-H(28B)	109.5
C(26)-C(28)-H(28C)	109.5	O(4)-C(24)-C(19)	119.5(3)
N(3)-C(24)-O(4)	118.3(3)	N(3)-C(24)-C(19)	122.2(3)
H(36A)-C(36)-H(36B)	109.4	C(35)-C(36)-H(36A)	111.6
C(35)-C(36)-H(36B)	111.6	C(35)-C(36)-C(37)	100.9(3)
C(37)-C(36)-H(36A)	111.6	C(37)-C(36)-H(36B)	111.6
O(7)-C(35)-C(36)	105.6(3)	O(7)-C(35)-H(35A)	110.6
O(7)-C(35)-H(35B)	110.6	C(36)-C(35)-H(35A)	110.6
C(36)-C(35)-H(35B)	110.6	H(35A)-C(35)-H(35B)	108.7
N(3)-C(26)-C(28)	109.5(3)	N(3)-C(26)-C(27)	110.0(3)
N(3)-C(26)-C(25)	101.3(3)	C(28)-C(26)-C(27)	111.4(3)
C(28)-C(26)-C(25)	112.0(3)	C(27)-C(26)-C(25)	112.2(3)
C(26)-C(27)-H(27A)	109.5	C(26)-C(27)-H(27B)	109.5
C(26)-C(27)-H(27C)	109.5	H(27A)-C(27)-H(27B)	109.5
H(27A)-C(27)-H(27C)	109.5	H(27B)-C(27)-H(27C)	109.5
C(32)-C(33)-H(33A)	109.5	C(32)-C(33)-H(33B)	109.5
C(32)-C(33)-H(33C)	109.5	H(33A)-C(33)-H(33B)	109.5
H(33A)-C(33)-H(33C)	109.5	H(33B)-C(33)-H(33C)	109.5
O(1)-C(14)-C(15)	104.8(2)	O(1)-C(14)-H(14A)	110.8
O(1)-C(14)-H(14B)	110.8	C(15)-C(14)-H(14A)	110.8
C(15)-C(14)-H(14B)	110.8	H(14A)-C(14)-H(14B)	108.9

C(21)-C(20)-H(20)	120.3	C(19)-C(20)-C(21)	119.4(3)
C(19)-C(20)-H(20)	120.3	O(4)-C(25)-C(26)	105.1(2)
O(4)-C(25)-H(25A)	110.7	O(4)-C(25)-H(25B)	110.7
C(26)-C(25)-H(25A)	110.7	C(26)-C(25)-H(25B)	110.7
H(25A)-C(25)-H(25B)	108.8	C(36)-C(37)-H(37A)	111.4
C(36)-C(37)-H(37B)	111.4	H(37A)-C(37)-H(37B)	109.3
C(38)-C(37)-C(36)	101.7(4)	C(38)-C(37)-H(37A)	111.4
C(38)-C(37)-H(37B)	111.4	C(9)-C(11)-H(11A)	109.5
C(9)-C(11)-H(11B)	109.5	C(9)-C(11)-H(11C)	109.5
H(11A)-C(11)-H(11B)	109.5	H(11A)-C(11)-H(11C)	109.5
H(11B)-C(11)-H(11C)	109.5	O(7)-C(38)-C(37)	105.9(3)
O(7)-C(38)-H(38A)	110.6	O(7)-C(38)-H(38B)	110.6
C(37)-C(38)-H(38A)	110.6	C(37)-C(38)-H(38B)	110.6
H(38A)-C(38)-H(38B)	108.7	O(5)-C(29)-H(29A)	109.5
O(5)-C(29)-H(29B)	109.5	O(5)-C(29)-H(29C)	109.5
H(29A)-C(29)-H(29B)	109.5	H(29A)-C(29)-H(29C)	109.5
H(29B)-C(29)-H(29C)	109.5		

5.5. Single Crystal Structure Analysis of Complex **8b**

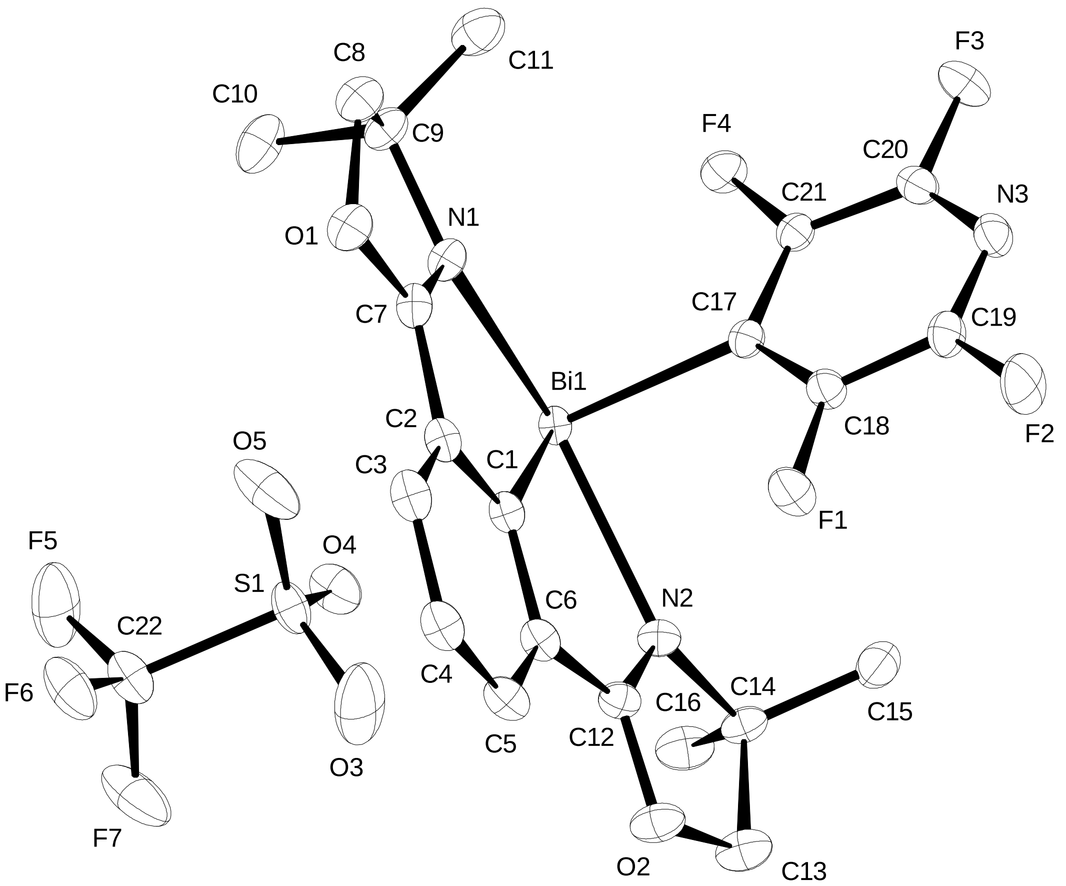

Figure S12. The molecular structure of complex **8b**. H atoms have been removed for clarity.

X-ray Crystal Structure Analysis of complex 8b: $C_{22}H_{19}Bi\,F_7N_3O_5S$, M_r = 779.44 g mol^{-1}, colourless plate, crystal size 0.10 x 0.05 x 0.05 mm^3, monoclinic, $P2_1/n$ [14], a = 10.5269(15) Å, b = 19.8254(12) Å, c = 13.0239(3) Å, β= 112.721(5)°, V = 2507.2(4) Å^3, T = 100(2) K, Z = 4, D_{calc} = 2.065 g·cm^3, λ = 0.71073 Å, $\mu(Mo\text{-}K_\alpha)$= 7.208 mm^{-1}, Gaussian absorption correction ($T_{\min}$ = 0.40907, $T_{\max}$ = 0.72684), Bruker AXS Enraf-Nonius KappaCCD diffractometer with a FR591 rotating Mo-anode X-ray source, 2.664 < θ < 33.098°, 59463 measured reflections, 9520 independent reflections, 7997 reflections with $I > 2\sigma(I)$, R_{int} = 0.0531]. The structure was solved by *SHELXT* and refined by full-matrix least-squares (*SHELXL*) against F^2 to R_1 = 0.0252 [$I > 2\sigma(I)$], wR_2 = 0.0546, 356 parameters.

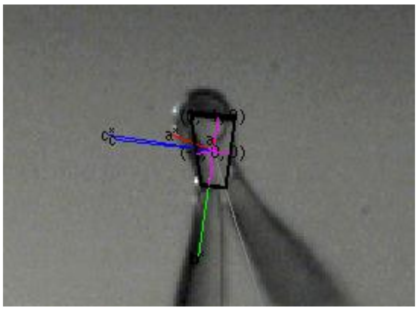

	h	k	l	distance (mm)
1.	0	-1	0	0.08
2.	0	1	0	0.08
3.	0	0	-1	0.04
4.	0	1	2	0.04
5.	-1	0	0	0.025
6.	1	0	0	0.025

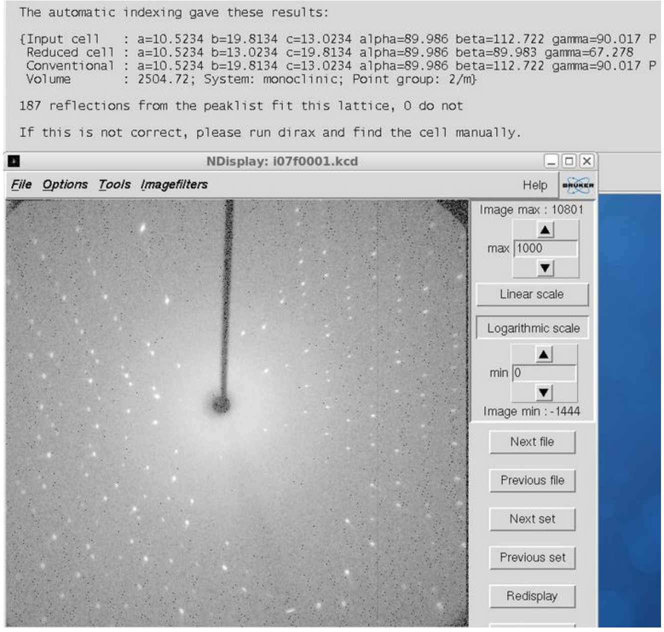

Figure S13. Crystal faces and unit cell determination of complex **8b**.

INTENSITY STATISTICS FOR DATASET

Resolution	#Data	#Theory	%Complete	Redundancy	Mean I	Mean I/s	Rmerge	Rsigma
Inf - 2.63	148	156	94.9	12.49	125.53	58.55	0.0449	0.0140
2.63 - 1.78	349	349	100.0	9.42	88.47	45.40	0.0454	0.0176
1.78 - 1.41	486	486	100.0	8.55	68.81	40.32	0.0439	0.0189
1.41 - 1.23	498	498	100.0	8.18	52.26	36.37	0.0441	0.0203
1.23 - 1.11	522	522	100.0	7.78	41.77	32.51	0.0411	0.0223
1.11 - 1.03	492	492	100.0	7.38	38.57	29.98	0.0417	0.0239
1.03 - 0.97	489	489	100.0	7.15	34.55	27.57	0.0436	0.0253
0.97 - 0.92	520	520	100.0	6.72	26.40	24.03	0.0494	0.0292
0.92 - 0.88	467	467	100.0	6.46	24.77	22.20	0.0508	0.0312
0.88 - 0.85	448	448	100.0	6.14	23.05	21.11	0.0554	0.0336
0.85 - 0.82	478	478	100.0	5.97	20.26	18.98	0.0605	0.0367
0.82 - 0.79	597	598	99.8	5.72	18.31	17.56	0.0658	0.0403
0.79 - 0.77	423	423	100.0	5.47	16.27	16.09	0.0724	0.0452
0.77 - 0.75	490	490	100.0	5.25	16.49	15.70	0.0781	0.0472
0.75 - 0.73	540	540	100.0	5.06	13.75	13.46	0.0849	0.0544
0.73 - 0.71	602	602	100.0	4.89	13.59	12.77	0.0960	0.0590
0.71 - 0.70	302	302	100.0	4.74	12.29	11.79	0.1058	0.0663
0.70 - 0.68	720	720	100.0	4.58	11.00	10.46	0.1121	0.0775
0.68 - 0.67	395	395	100.0	4.33	10.14	9.38	0.1176	0.0892
0.67 - 0.66	384	384	100.0	4.08	10.51	9.00	0.1157	0.0966
0.66 - 0.65	417	422	98.8	3.82	8.47	7.04	0.1364	0.1269
0.75 - 0.65	3360	3365	99.9	4.55	11.55	10.76	0.1048	0.0758
Inf - 0.65	9767	9781	99.9	6.17	28.32	21.50	0.0524	0.0321

Complete .cif-data of the compound are available under the CCDC number **CCDC-2091968**.

Table S13. Crystal data and structure refinement of complex **8b**.

Identification code	13833	
Empirical formula	$C_{22}H_{19}Bi\,F_7N_3O_5S$	
Color	colourless	
Formula weight	779.44 g · mol^{-1}	
Temperature	100(2) K	
Wavelength	0.71073 Å	
Crystal system	monoclinic	
Space group	$P2_1$/c, (No. 14)	
Unit cell dimensions	a = 10.5269(15) Å	α= 90°.
	b = 19.8254(12) Å	β= 112.721(5)°.
	c = 13.0239(3) Å	γ = 90°.
Volume	2507.2(4) Å^3	
Z	4	
Density (calculated)	2.065 Mg·m^{-3}	
Absorption coefficient	7.208 mm^{-1}	
F(000)	1496 e	
Crystal size	0.16 x 0.08 x 0.05 mm^3	
θ range for data collection	2.664 to 33.098°.	
Index ranges	$-16 \le h \le 16$, $-30 \le k \le 30$, $-20 \le l \le 20$	
Reflections collected	59463	
Independent reflections	9520 [R_{int} = 0.0531]	
Reflections with I>2σ(I)	7997	
Completeness to θ = 25.242°	99.9 %	
Absorption correction	Gaussian	
Max. and min. transmission	0.72684 and 0.40907	
Refinement method	Full-matrix least-squares on F^2	
Data / restraints / parameters	9520 / 0 / 356	
Goodness-of-fit on F^2	1.052	
Final R indices [I>2σ(I)]	R_1 = 0.0252	wR^2 = 0.0546
R indices (all data)	R_1 = 0.0359	wR^2 = 0.0582
Largest diff. peak and hole	1.5 and -2.2 e · Å^{-3}	

Table S14. Bond lengths [Å] and angles [°] of complex **8b**.

Bi(1)-N(1)	2.4779(19)	Bi(1)-N(2)	2.450(2)
Bi(1)-C(1)	2.225(2)	Bi(1)-C(17)	2.294(2)
F(1)-C(18)	1.345(3)	F(2)-C(19)	1.339(3)
F(3)-C(20)	1.335(3)	F(4)-C(21)	1.347(3)
O(1)-C(7)	1.341(3)	O(1)-C(8)	1.461(3)
O(2)-C(12)	1.332(3)	O(2)-C(13)	1.461(3)
N(1)-C(7)	1.286(3)	N(1)-C(9)	1.497(3)
N(2)-C(12)	1.280(3)	N(2)-C(14)	1.489(3)
N(3)-C(19)	1.312(3)	N(3)-C(20)	1.311(3)
C(1)-C(2)	1.390(3)	C(1)-C(6)	1.385(3)
C(2)-C(3)	1.401(3)	C(2)-C(7)	1.465(3)
C(3)-C(4)	1.395(4)	C(4)-C(5)	1.392(3)
C(5)-C(6)	1.397(3)	C(6)-C(12)	1.464(3)
C(8)-C(9)	1.537(3)	C(9)-C(10)	1.529(4)
C(9)-C(11)	1.521(4)	C(13)-C(14)	1.556(4)
C(14)-C(15)	1.519(4)	C(14)-C(16)	1.524(4)
C(17)-C(18)	1.377(3)	C(17)-C(21)	1.383(3)
C(18)-C(19)	1.383(3)	C(20)-C(21)	1.384(3)
S(1)-O(3)	1.436(3)	S(1)-O(4)	1.4399(19)
S(1)-O(5)	1.440(2)	S(1)-C(22)	1.818(3)
F(5)-C(22)	1.326(3)	F(6)-C(22)	1.335(3)
F(7)-C(22)	1.333(3)		
N(2)-Bi(1)-N(1)	141.31(6)	C(1)-Bi(1)-N(1)	70.90(8)
C(1)-Bi(1)-N(2)	71.51(8)	C(1)-Bi(1)-C(17)	93.60(8)
C(17)-Bi(1)-N(1)	86.39(7)	C(17)-Bi(1)-N(2)	87.16(8)
C(7)-O(1)-C(8)	104.12(18)	C(12)-O(2)-C(13)	105.85(19)
C(7)-N(1)-Bi(1)	111.73(15)	C(7)-N(1)-C(9)	107.16(19)
C(9)-N(1)-Bi(1)	140.00(14)	C(12)-N(2)-Bi(1)	111.01(15)
C(12)-N(2)-C(14)	108.8(2)	C(14)-N(2)-Bi(1)	139.84(15)
C(20)-N(3)-C(19)	116.5(2)	C(2)-C(1)-Bi(1)	120.48(18)
C(6)-C(1)-Bi(1)	119.04(16)	C(6)-C(1)-C(2)	120.5(2)
C(1)-C(2)-C(3)	120.0(2)	C(1)-C(2)-C(7)	114.7(2)
C(3)-C(2)-C(7)	125.2(2)	C(4)-C(3)-C(2)	119.0(2)
C(5)-C(4)-C(3)	121.1(2)	C(4)-C(5)-C(6)	119.2(2)
C(1)-C(6)-C(5)	120.2(2)	C(1)-C(6)-C(12)	114.7(2)
C(5)-C(6)-C(12)	124.9(2)	O(1)-C(7)-C(2)	120.2(2)

N(1)-C(7)-O(1)	117.6(2)	N(1)-C(7)-C(2)	122.1(2)
O(1)-C(8)-C(9)	104.28(19)	N(1)-C(9)-C(8)	99.93(19)
N(1)-C(9)-C(10)	108.0(2)	N(1)-C(9)-C(11)	111.2(2)
C(10)-C(9)-C(8)	111.9(2)	C(11)-C(9)-C(8)	112.7(2)
C(11)-C(9)-C(10)	112.3(2)	O(2)-C(12)-C(6)	119.0(2)
N(2)-C(12)-O(2)	118.4(2)	N(2)-C(12)-C(6)	122.5(2)
O(2)-C(13)-C(14)	105.6(2)	N(2)-C(14)-C(13)	101.37(19)
N(2)-C(14)-C(15)	109.2(2)	N(2)-C(14)-C(16)	110.2(2)
C(15)-C(14)-C(13)	112.2(2)	C(15)-C(14)-C(16)	111.6(2)
C(16)-C(14)-C(13)	111.8(2)	C(18)-C(17)-Bi(1)	126.89(17)
C(18)-C(17)-C(21)	115.1(2)	C(21)-C(17)-Bi(1)	117.74(16)
F(1)-C(18)-C(17)	122.0(2)	F(1)-C(18)-C(19)	117.7(2)
C(17)-C(18)-C(19)	120.3(2)	F(2)-C(19)-C(18)	119.6(2)
N(3)-C(19)-F(2)	116.4(2)	N(3)-C(19)-C(18)	124.0(2)
F(3)-C(20)-C(21)	119.8(2)	N(3)-C(20)-F(3)	116.7(2)
N(3)-C(20)-C(21)	123.5(2)	F(4)-C(21)-C(17)	120.5(2)
F(4)-C(21)-C(20)	119.0(2)	C(17)-C(21)-C(20)	120.6(2)
O(3)-S(1)-O(4)	115.64(15)	O(3)-S(1)-O(5)	113.84(17)
O(3)-S(1)-C(22)	103.61(14)	O(4)-S(1)-O(5)	114.37(12)
O(4)-S(1)-C(22)	103.78(12)	O(5)-S(1)-C(22)	103.53(13)
F(5)-C(22)-S(1)	111.88(19)	F(5)-C(22)-F(6)	106.9(2)
F(5)-C(22)-F(7)	107.8(2)	F(6)-C(22)-S(1)	111.13(17)
F(7)-C(22)-S(1)	111.53(19)	F(7)-C(22)-F(6)	107.3(2)

5.6. Clarification for Compound **3**, **4** and **5**

The structure **3** VUSVIQ (CCDC: 1409228) contains two molecules in the asymmetric unit of non-centrosymmetric space group Pna2_1 that differ in the conformation of the tert-butyl groups. In the solid the molecules are related by axial chirality as shown in Figure S14:

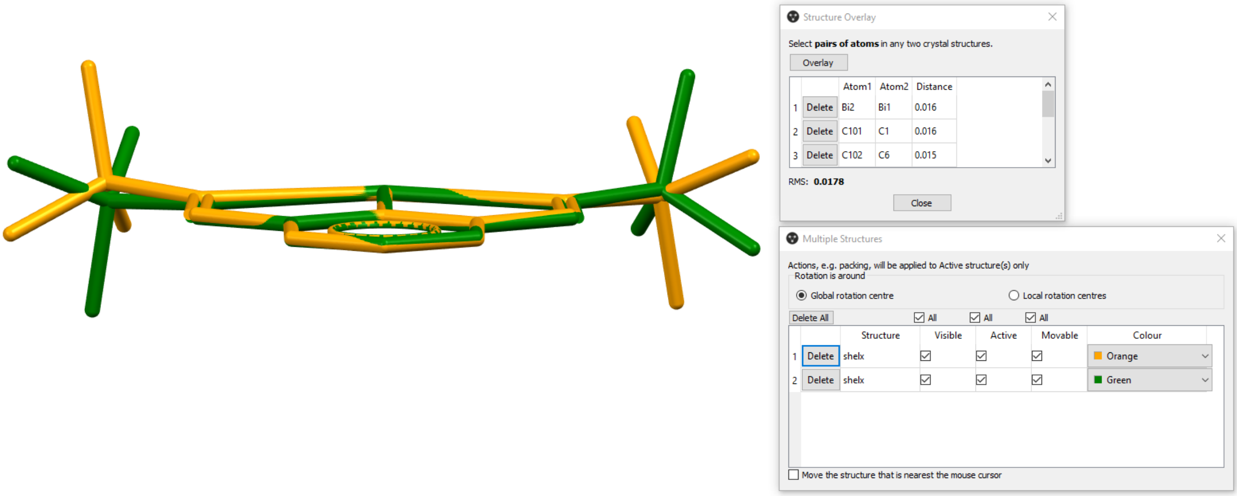

Figure S14. Two conformers in the asymmetric unit of **3** VUSVIQ.

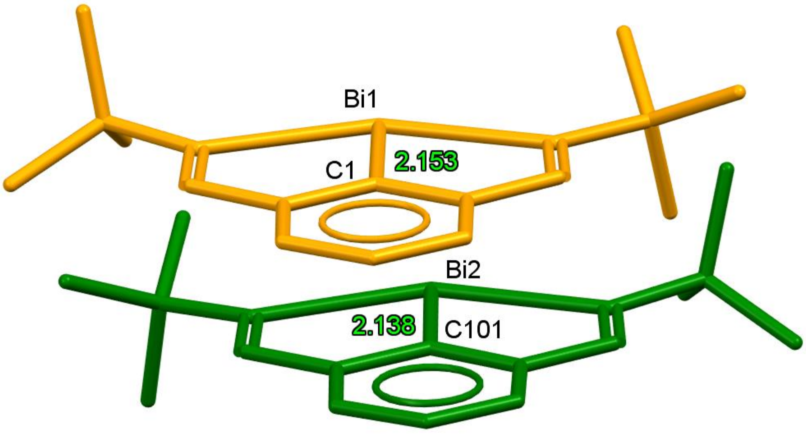

Figure S15. Bi-C bond distances of two indipendent molecules of **3** VUSVIQ.

For discussion, the averaged Bi-C distance of both independent molecules ($\bar{d}_{Bi-C}$ = 2.146 Å) was used and the standard uncertainties were summed (18).

The structure **4** (**CCDC-2091966**) contains two molecules in the asymmetric unit of non-centrosymmetric space group P$2_1 2_1 2_1$. The conformers are shown in Figure S16:

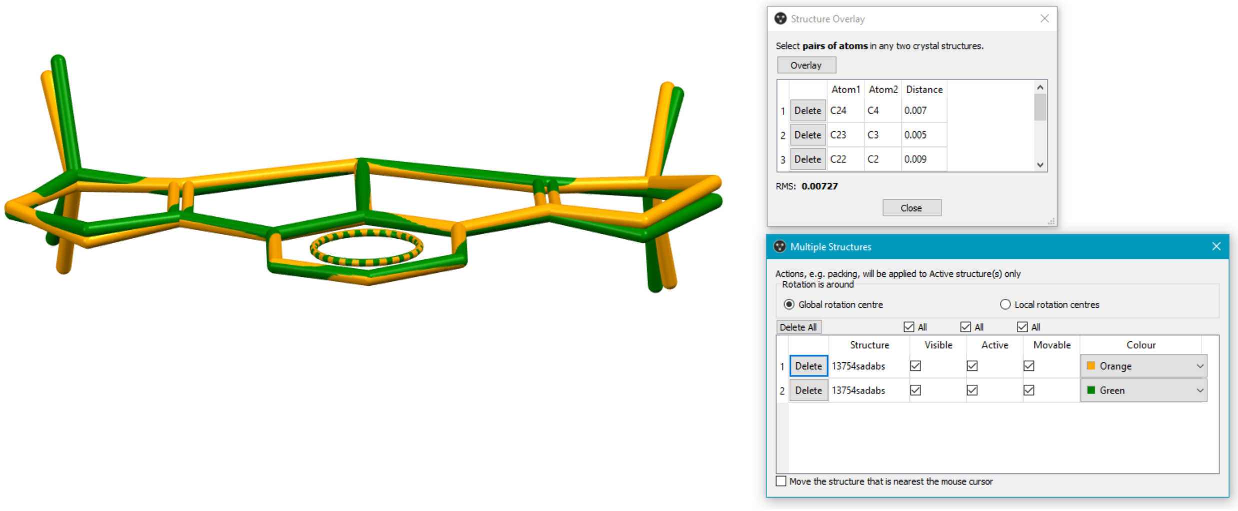

Figure S16. The two conformers in the asymmetric unit of **4**.

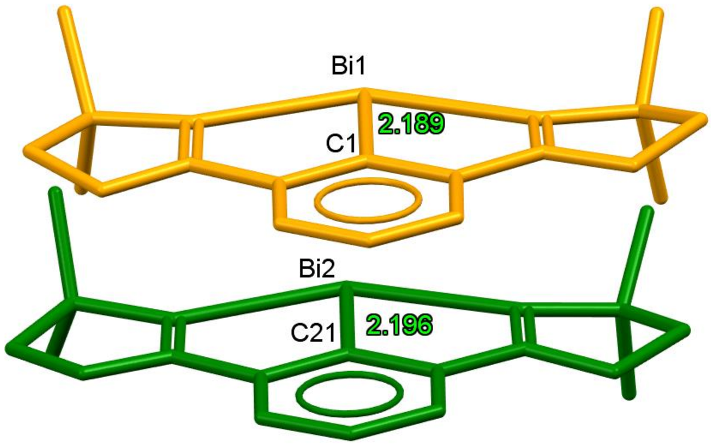

Figure S17. Bi-C bond distances of two indipendent molecules of **4**.

For discussion, the averaged Bi-C distance of both independent molecules ($\bar{d}_{Bi-C}$ = 2.193 Å) was used and the standard uncertainties were summed (6).

Compound **5** (**CCDC-2091965**) was found to crystallize in a centrosymmetric space group by checking the dataset using XPREP and ADDSYM in PLATON program.

6. NMR Spectra

6.1. NMR Spectra of Bismuth Compounds and the Precursors

2-Bromo-*N*,*N'*-bis(1-hydroxy-2-methylpropan-2-yl)isophthalamide (**S1**)

^{1}H NMR (DMSO-d_6, 400 MHz)

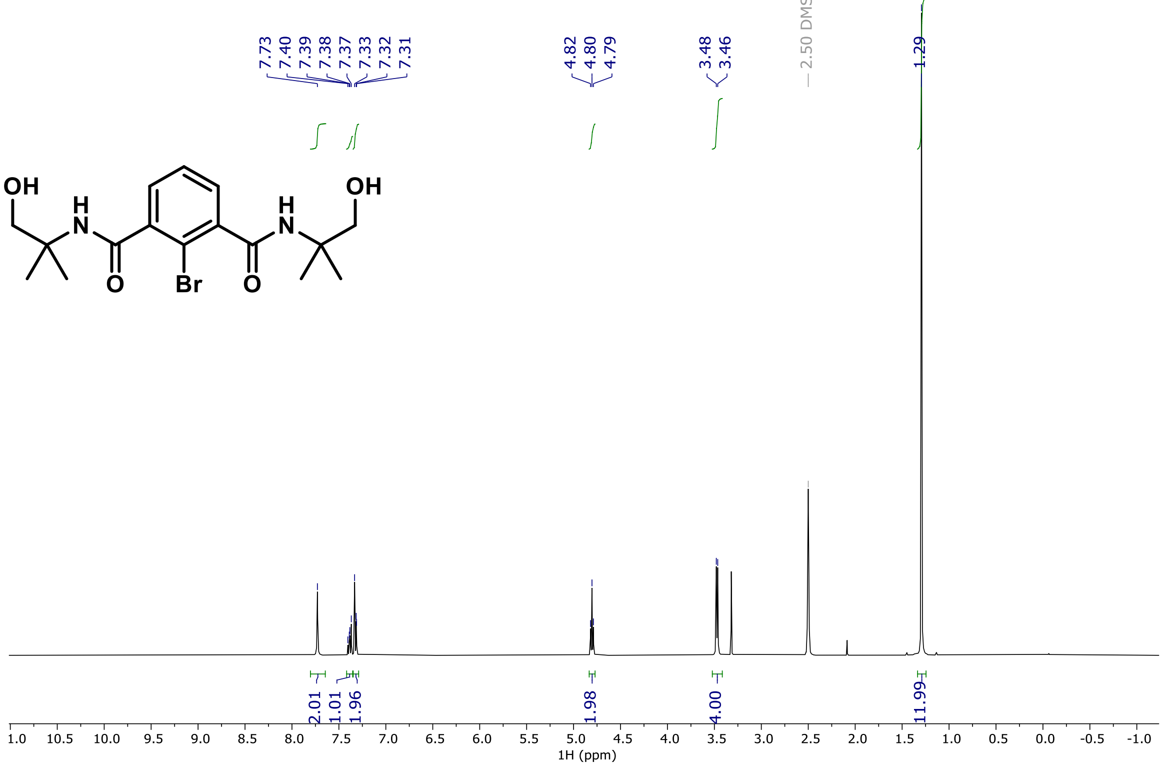

^{13}C NMR (DMSO-d_6, 101 MHz)

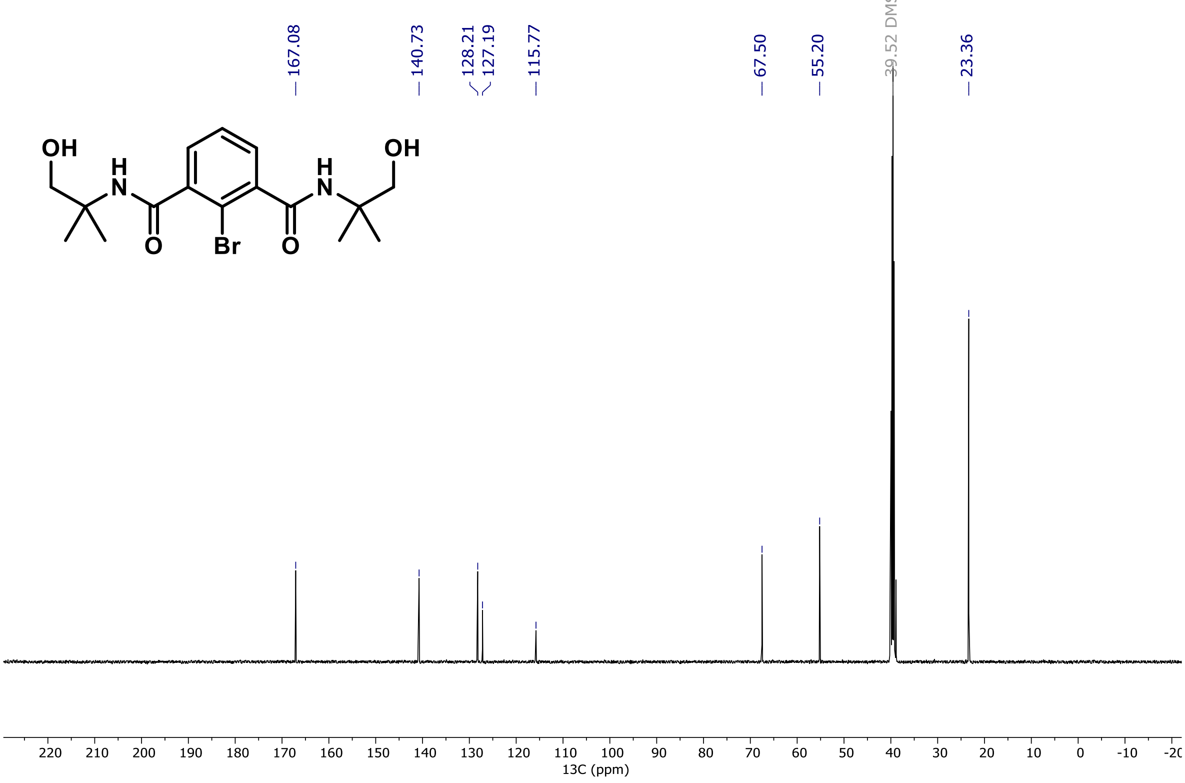

Phebox-Br (**10**)

^{1}H NMR ($CDCl_3$, 300 MHz)

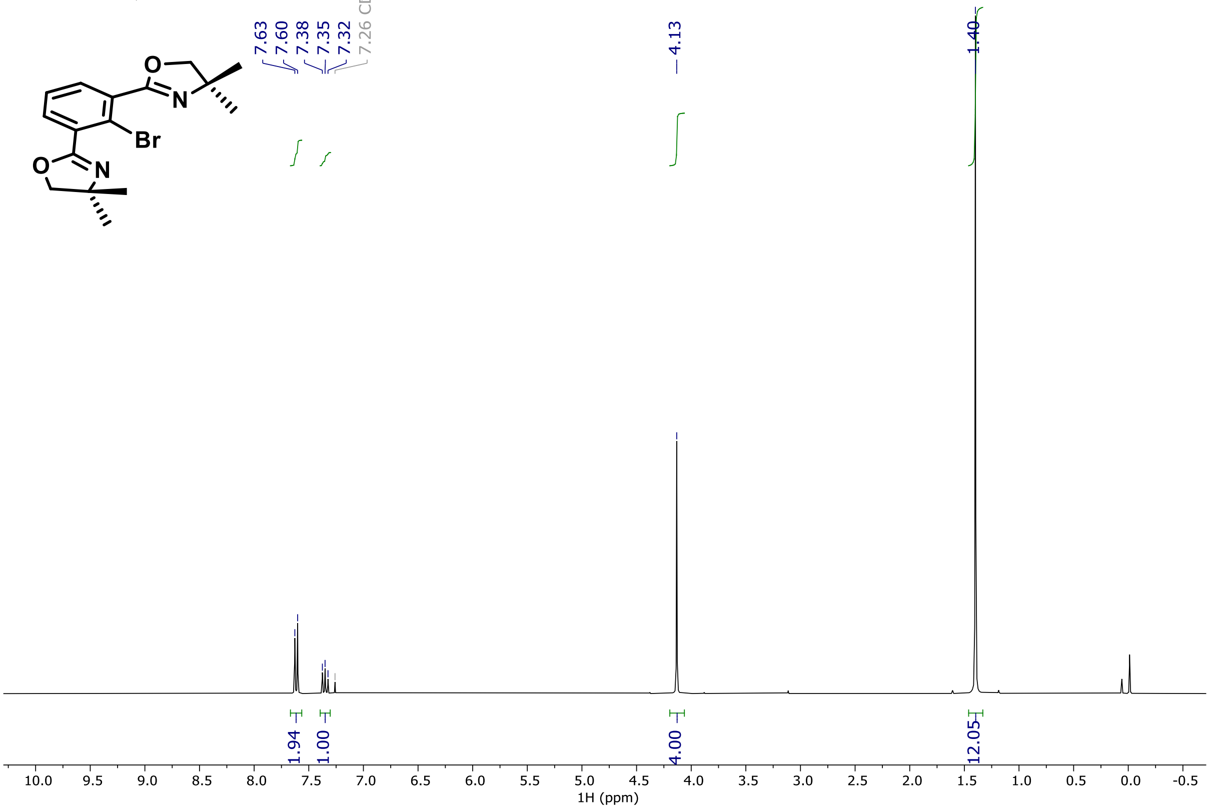

^{13}C NMR ($CDCl_3$, 75 MHz)

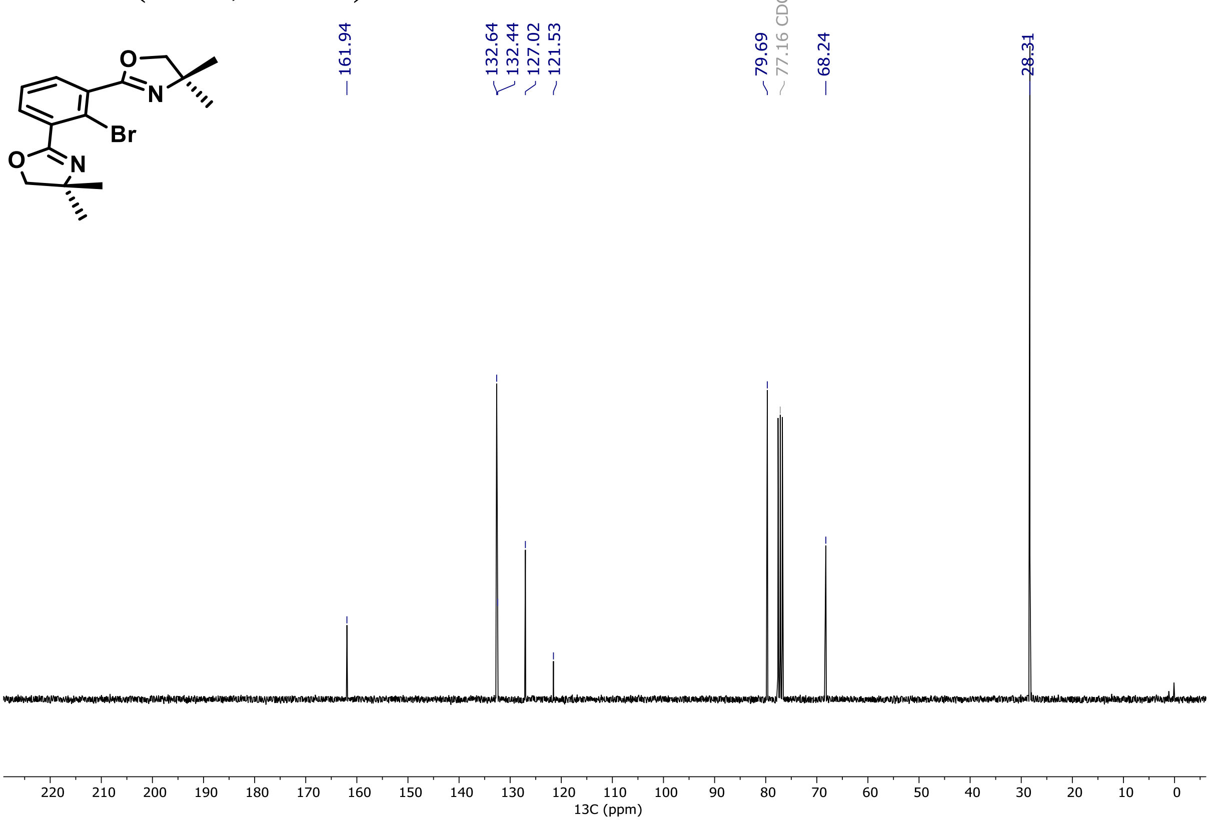

Phebox-$BiCl_2$ (**6**)

^{1}H NMR ($CDCl_3$, 400 MHz)

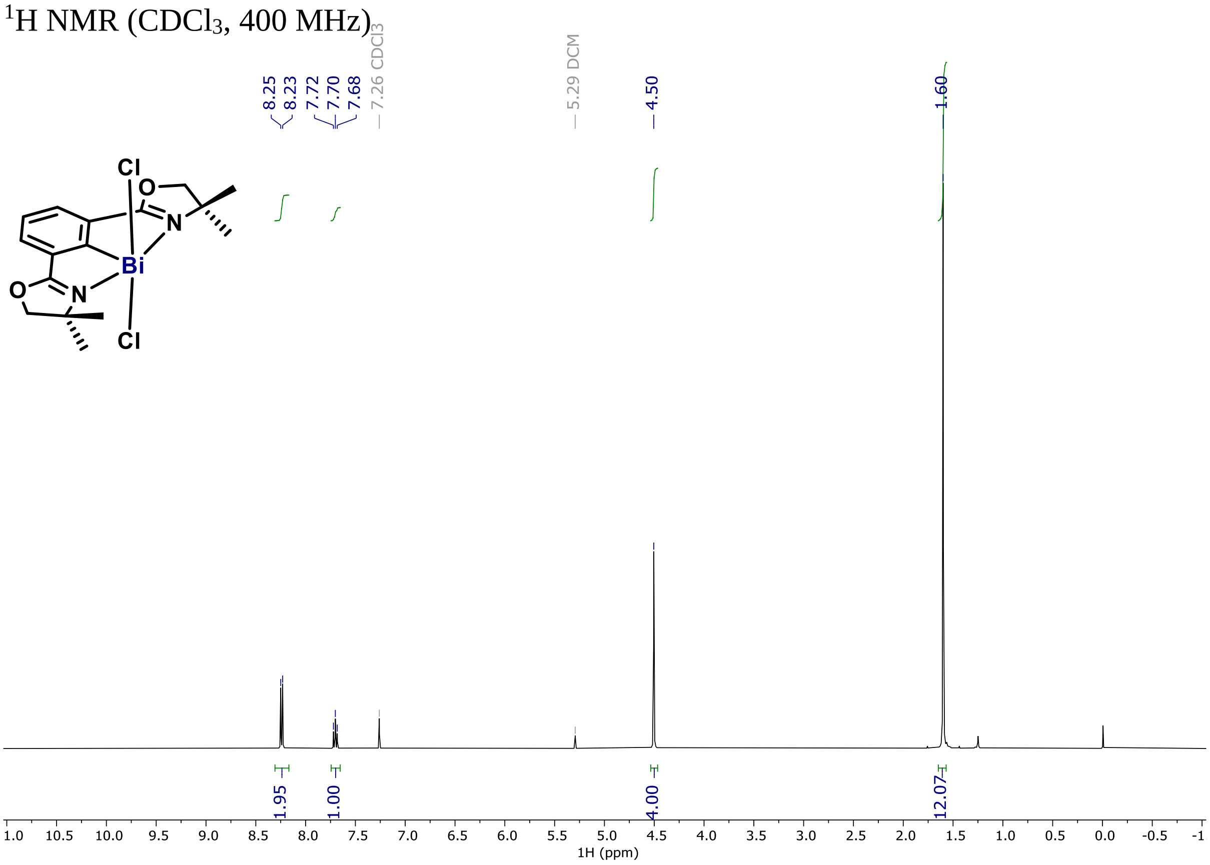

^{13}C NMR ($CDCl_3$, 101 MHz)

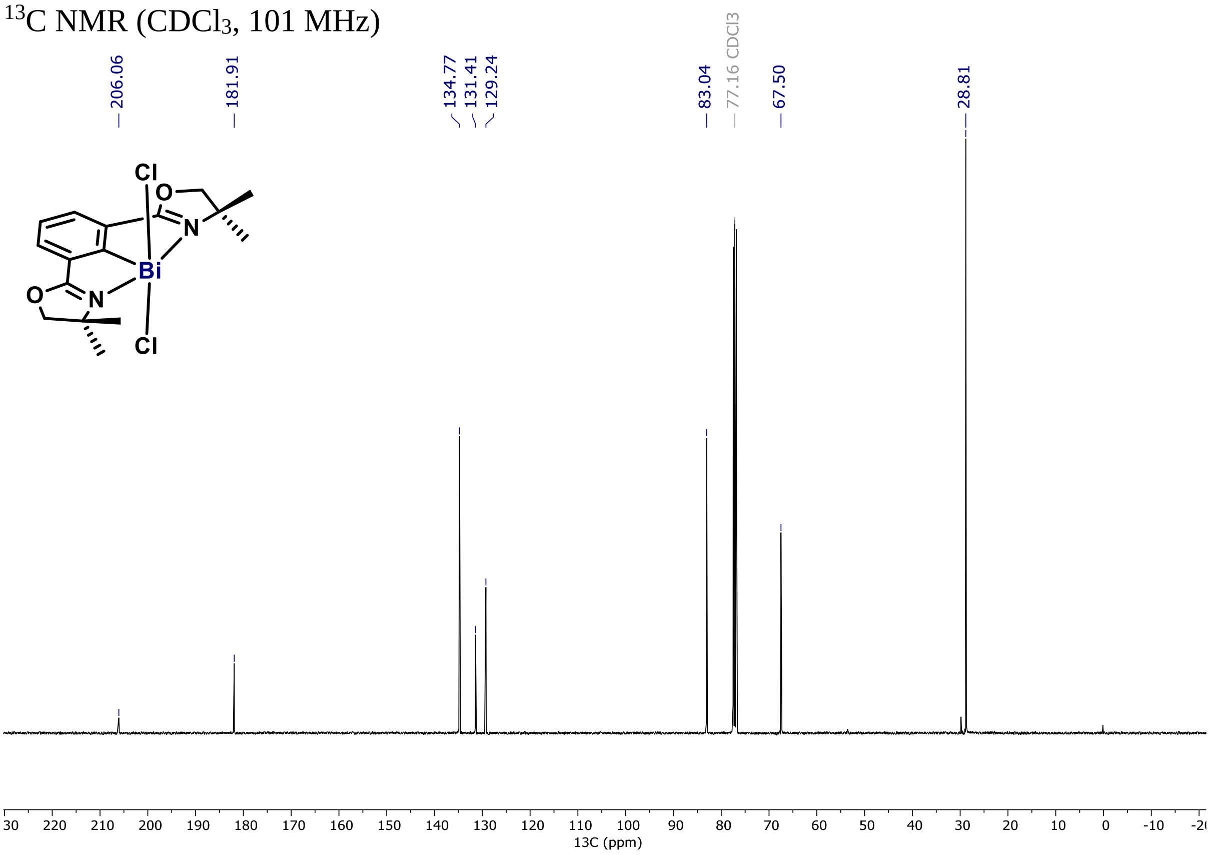

Phebox-Bi(I) (**4**)

^{1}H NMR (THF-d_8, 400 MHz)

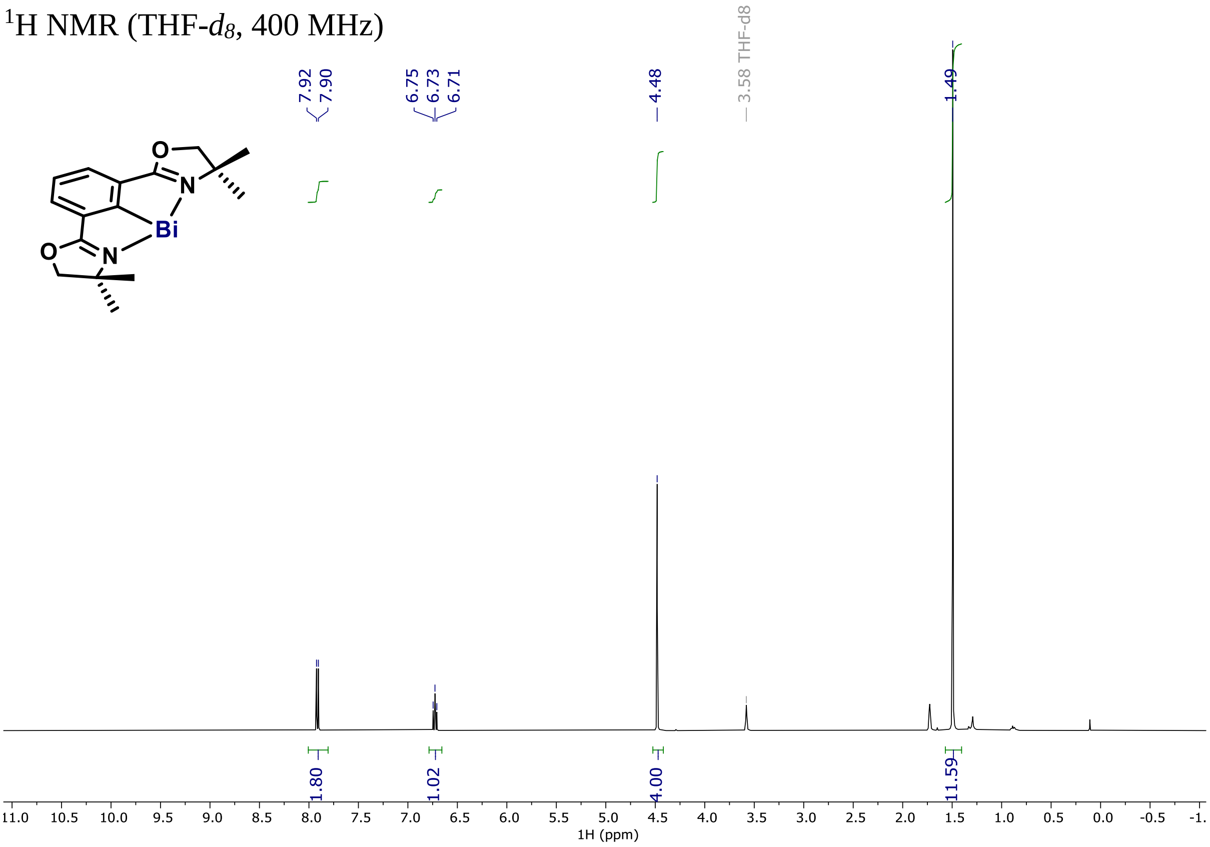

^{13}C NMR (THF-d_8, 101 MHz)

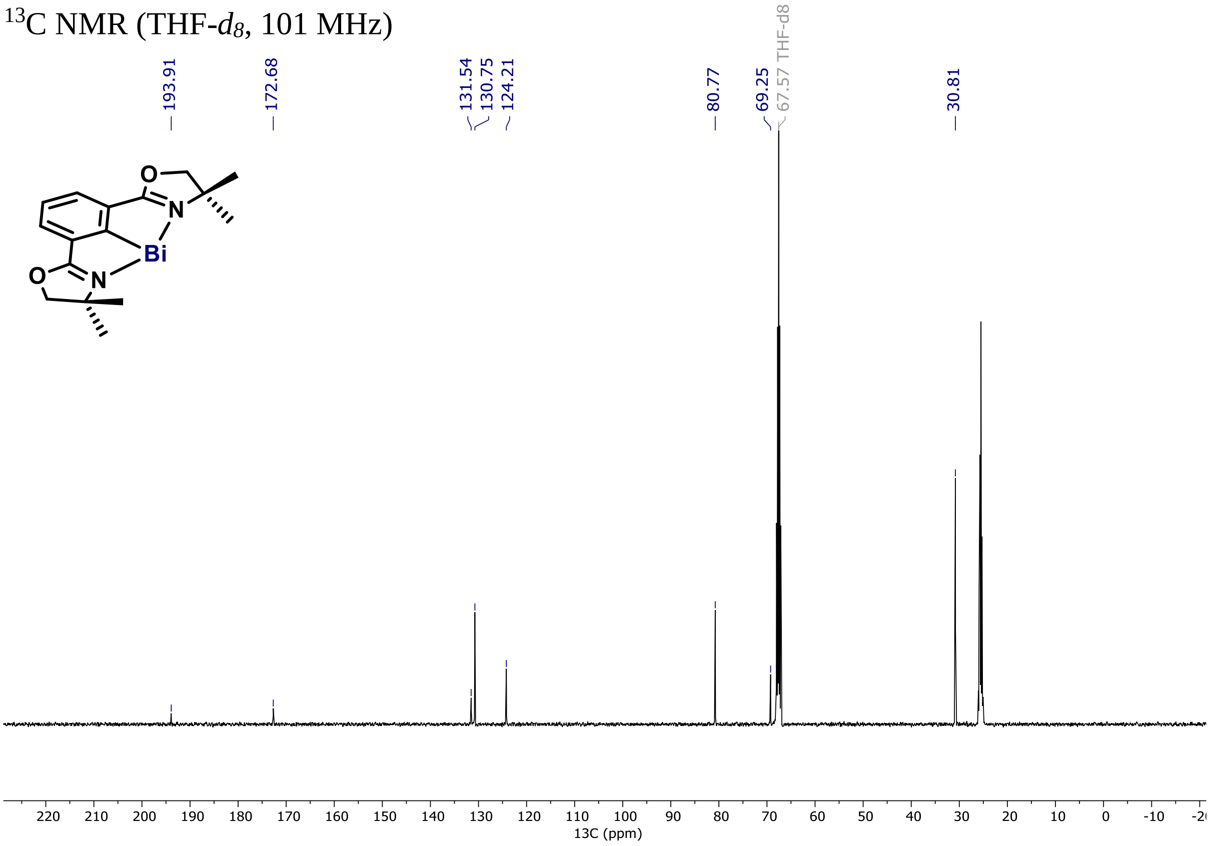

2-Bromo-*N*,*N'*-bis(1-hydroxy-2-methylpropan-2-yl)-5-methoxyisophthalamide (**S2**)

^{1}H NMR (DMSO-d_6, 300 MHz)

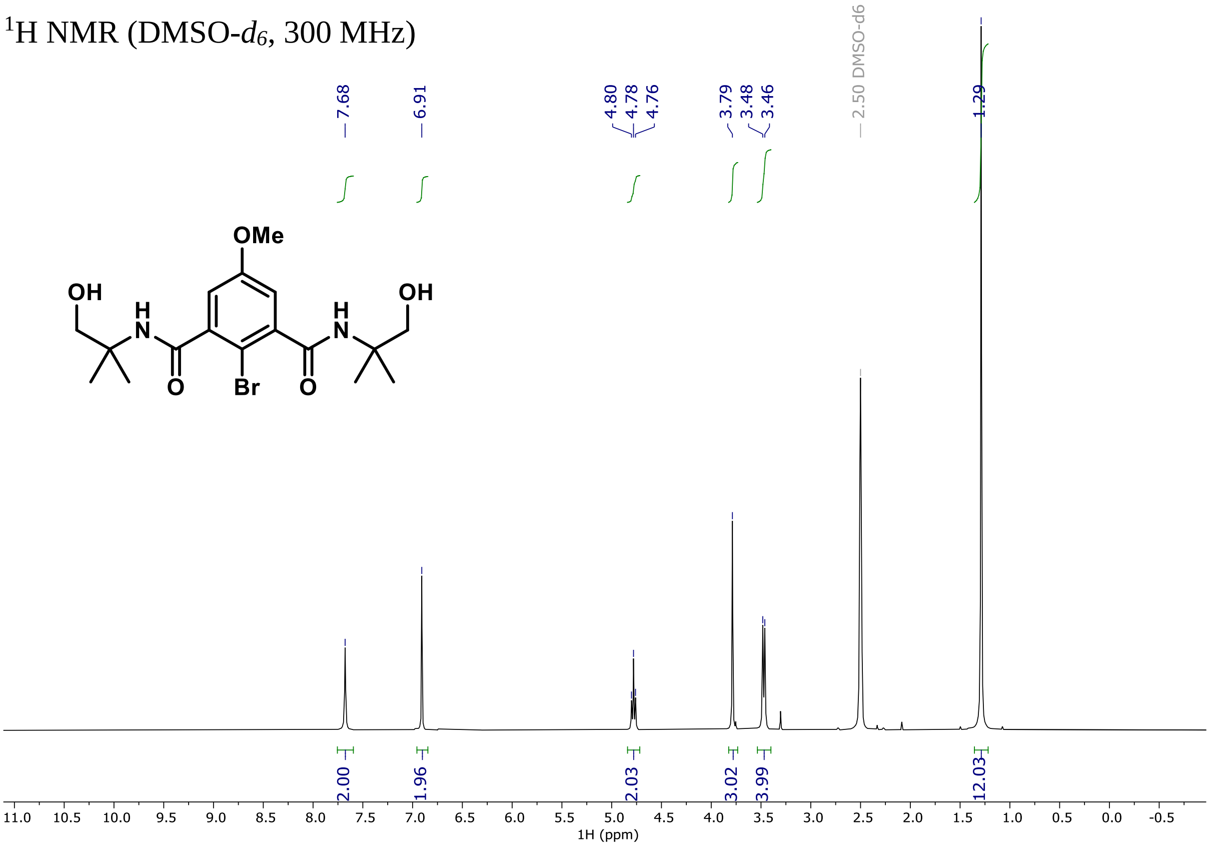

^{13}C NMR (DMSO-d_6, 75 MHz)

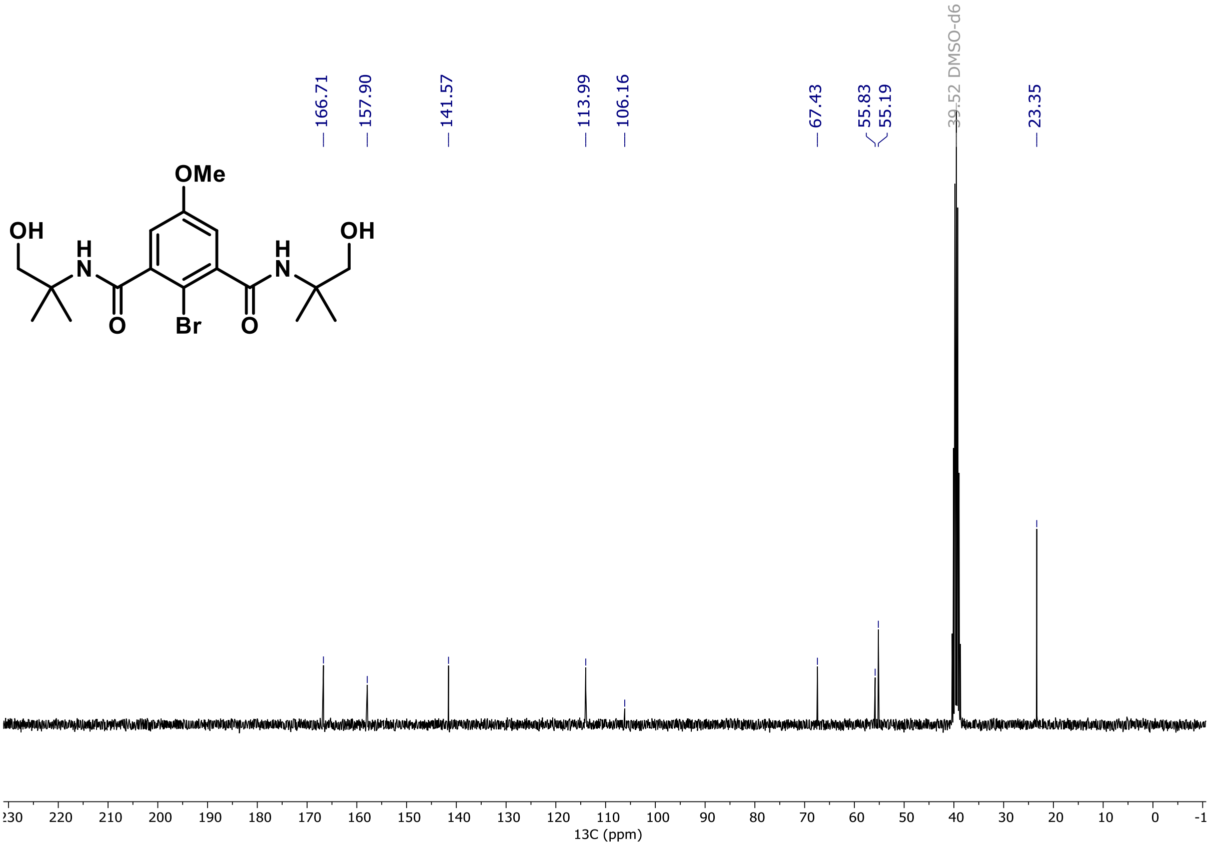

OMe-Phebox-Br (**S3**)

^{1}H NMR ($CDCl_3$, 300 MHz)

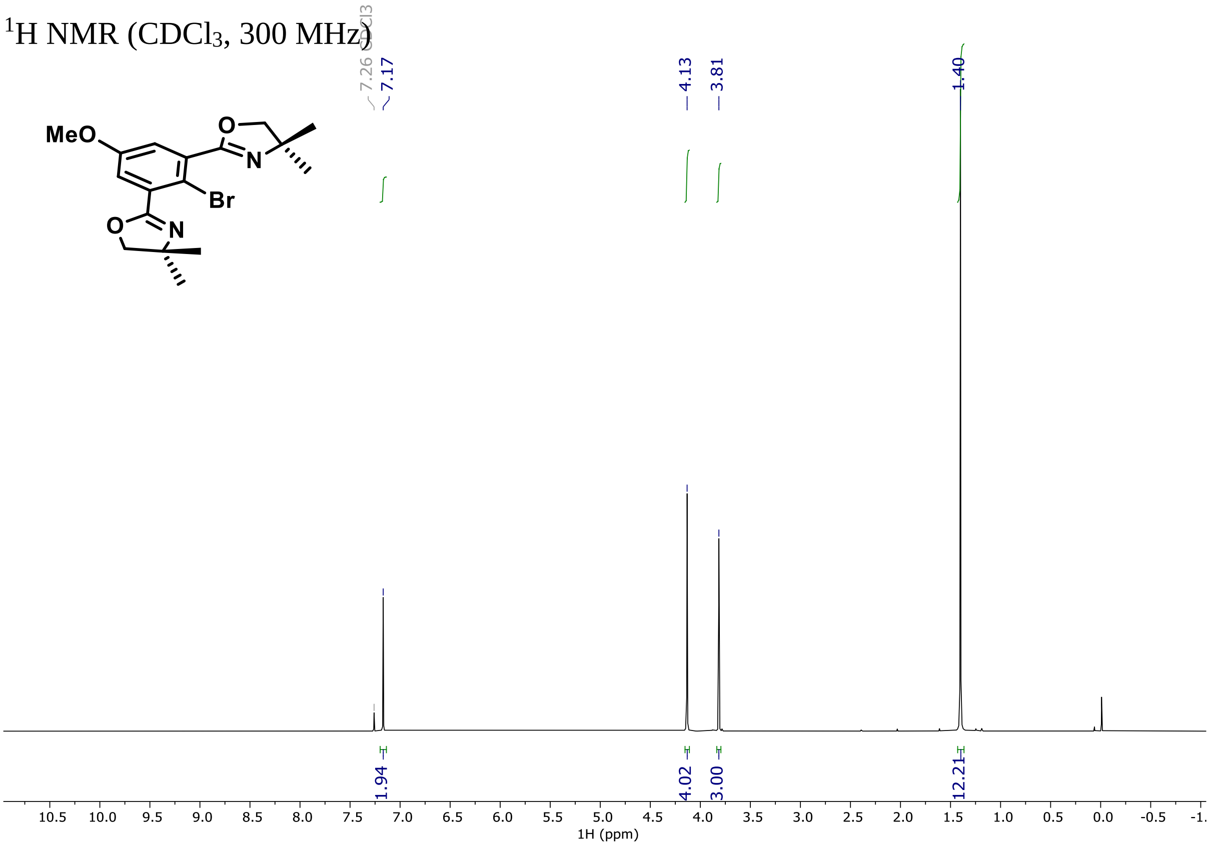

^{13}C NMR ($CDCl_3$, 75 MHz)

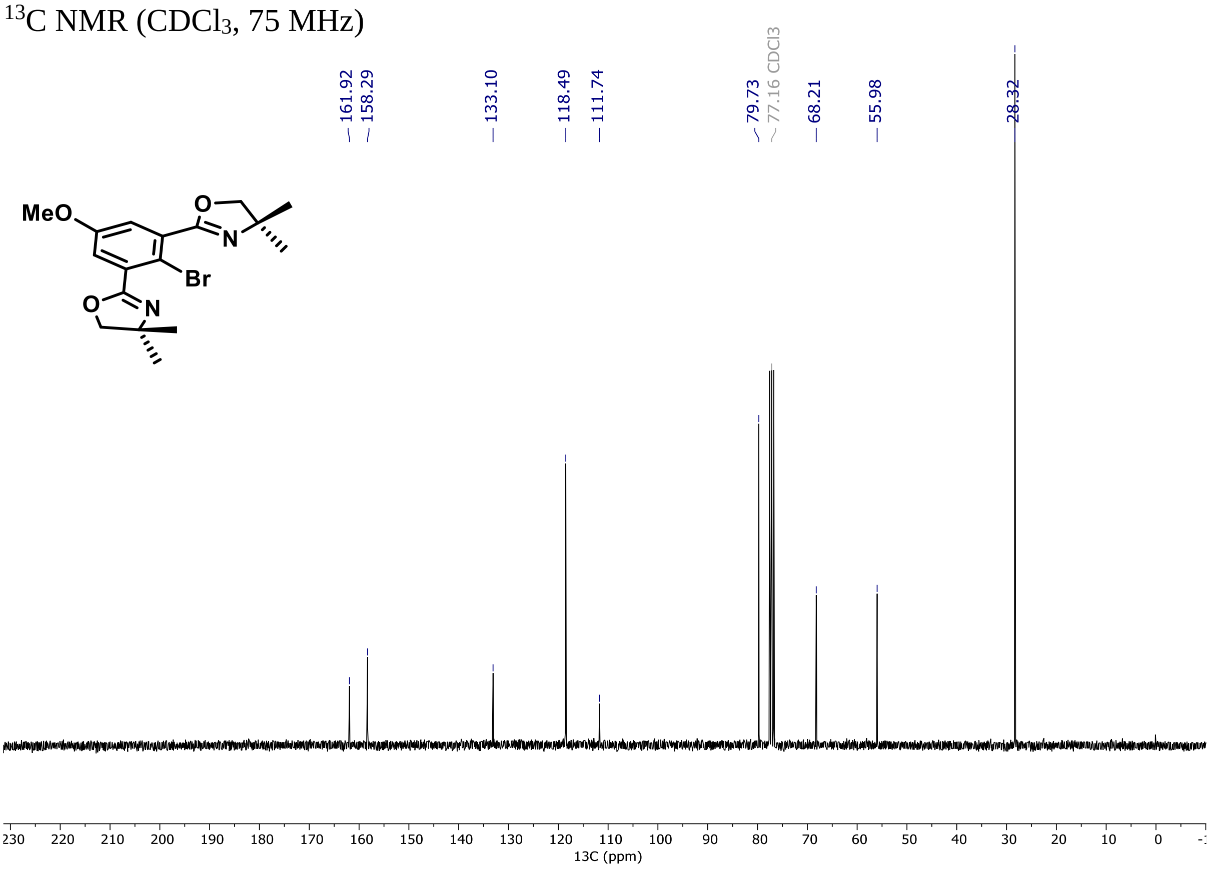

OMe-Phebox-BiCl$_2$ (**7**)

^{1}H NMR ($CDCl_3$, 400 MHz)

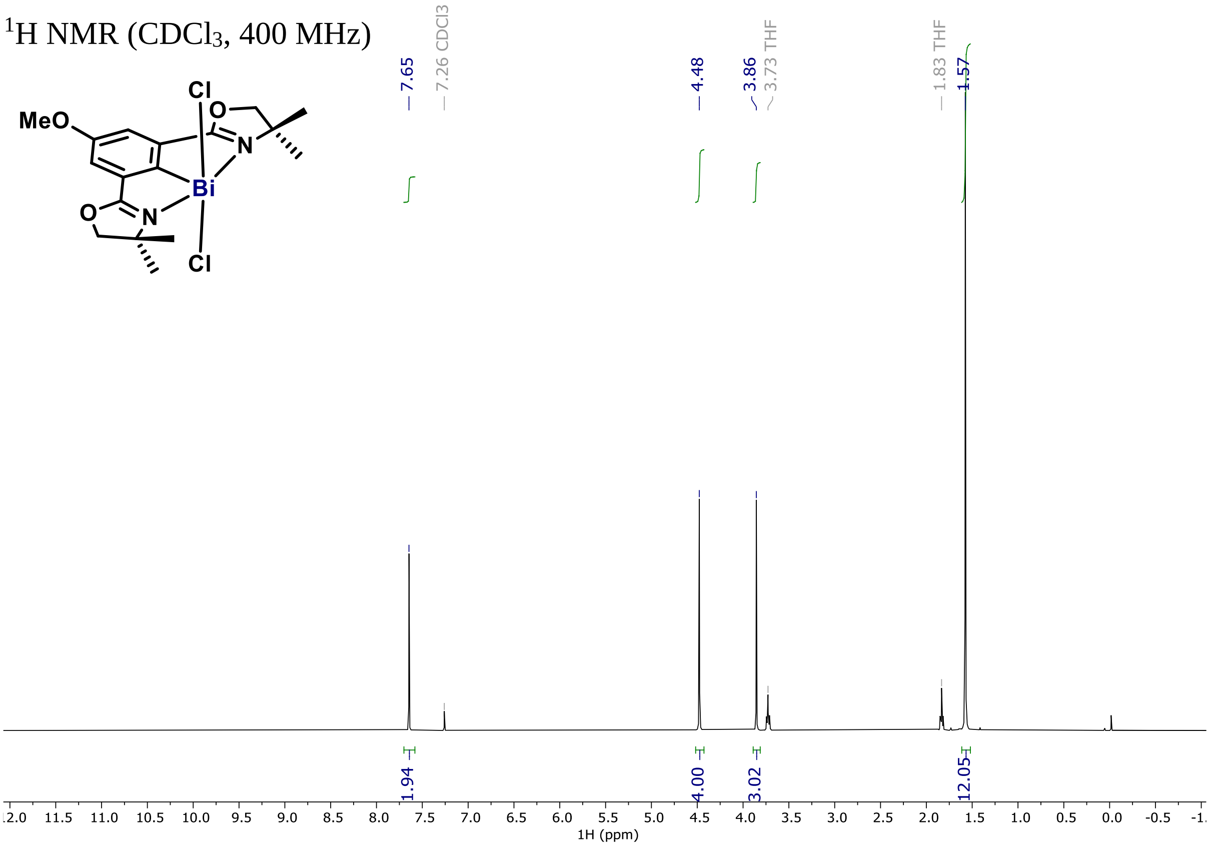

^{13}C NMR ($CDCl_3$, 101 MHz)

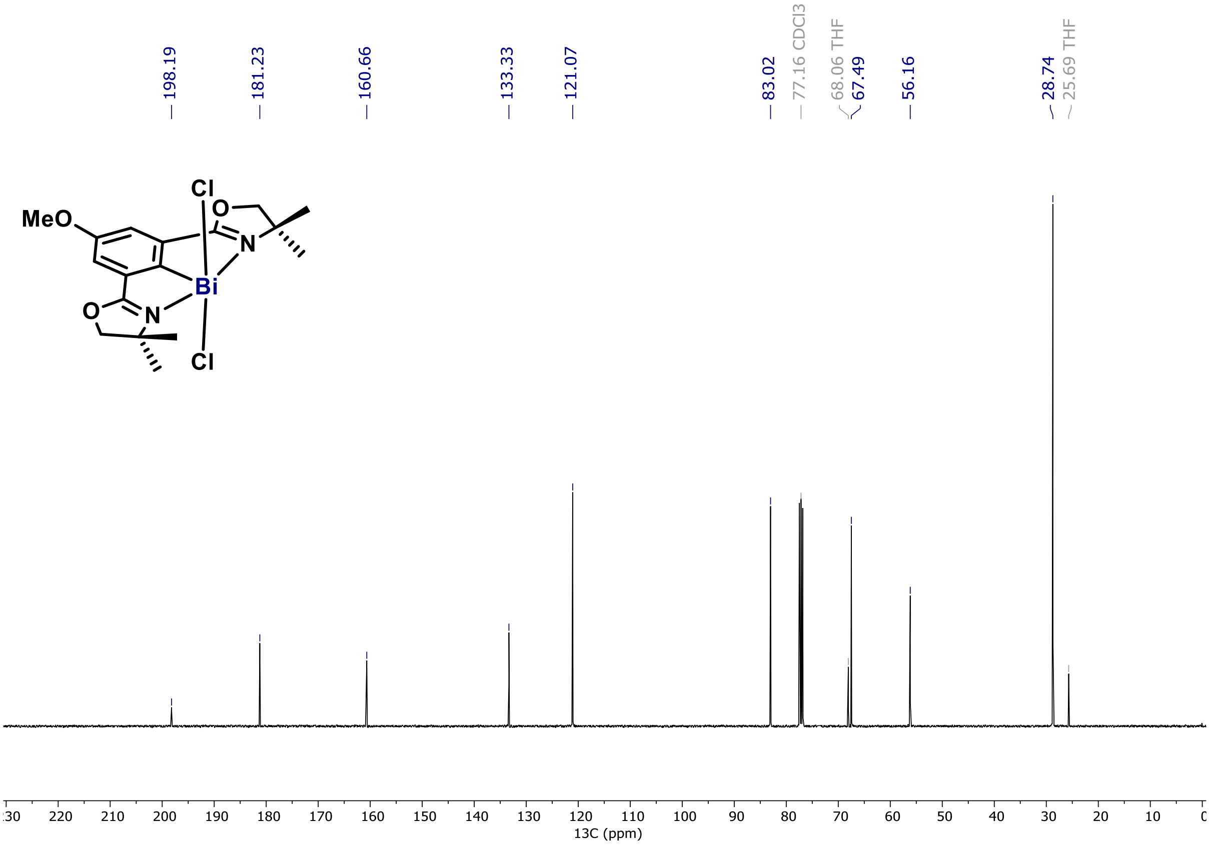

OMe-Phebox-Bi(I) (**5**)

^{1}H NMR (THF-d_8, 400 MHz)

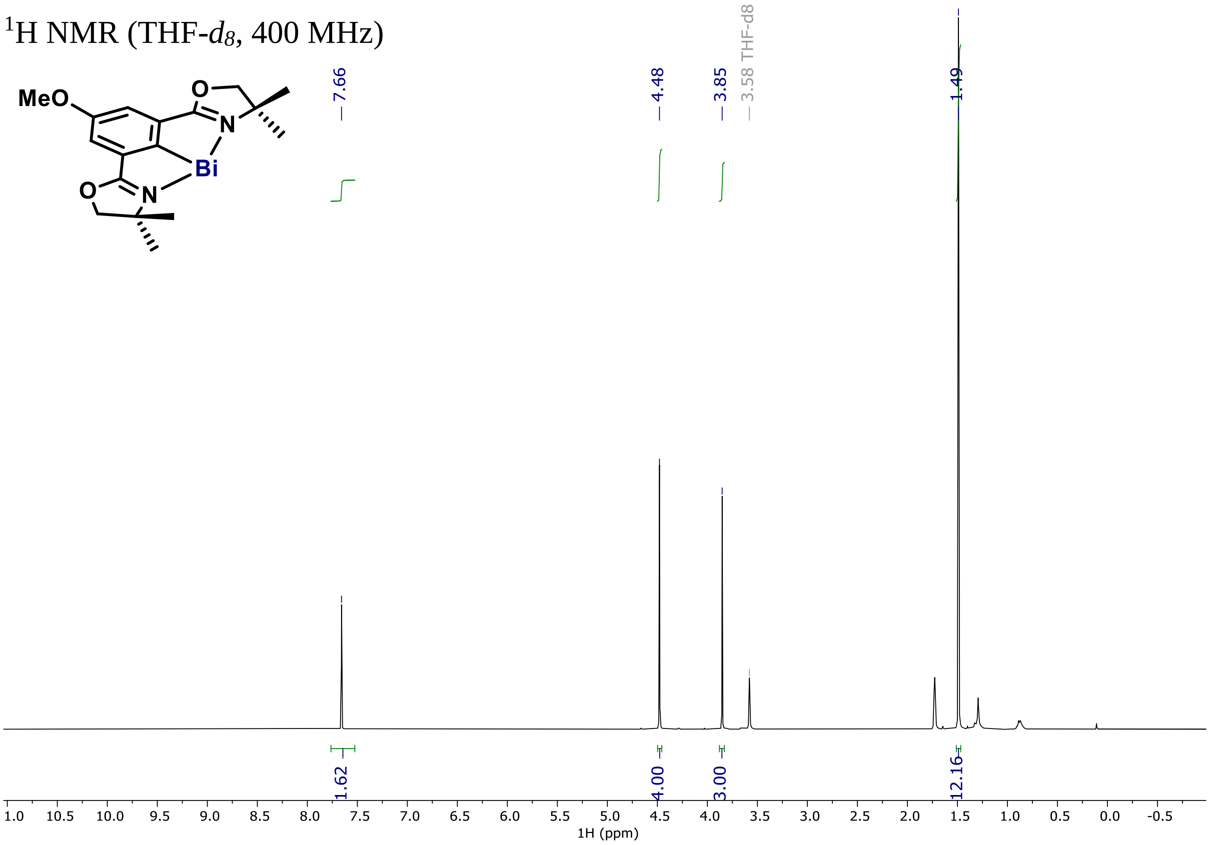

^{13}C NMR (THF-d_8, 101 MHz)

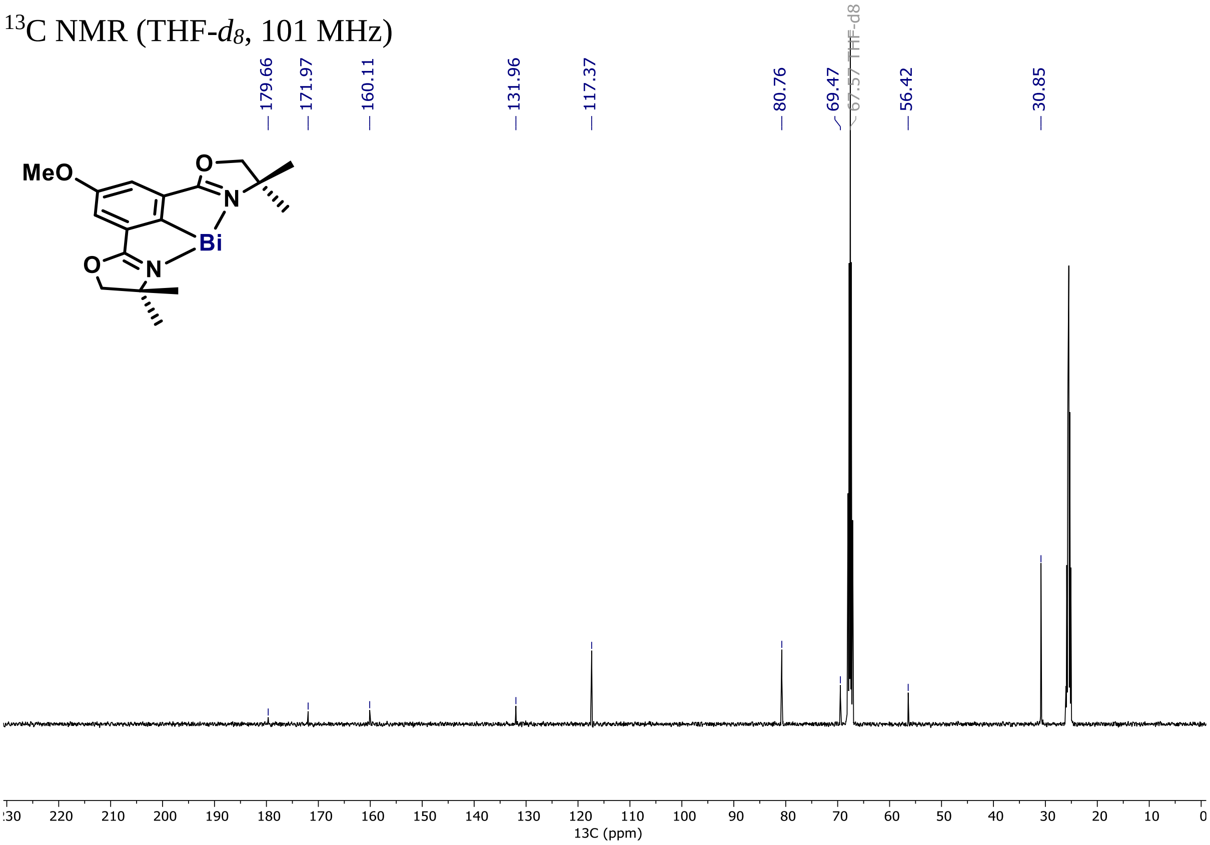

Phebox-BiF$_2$ (**S5**)

^{1}H NMR (CD$_3$CN, 400 MHz)

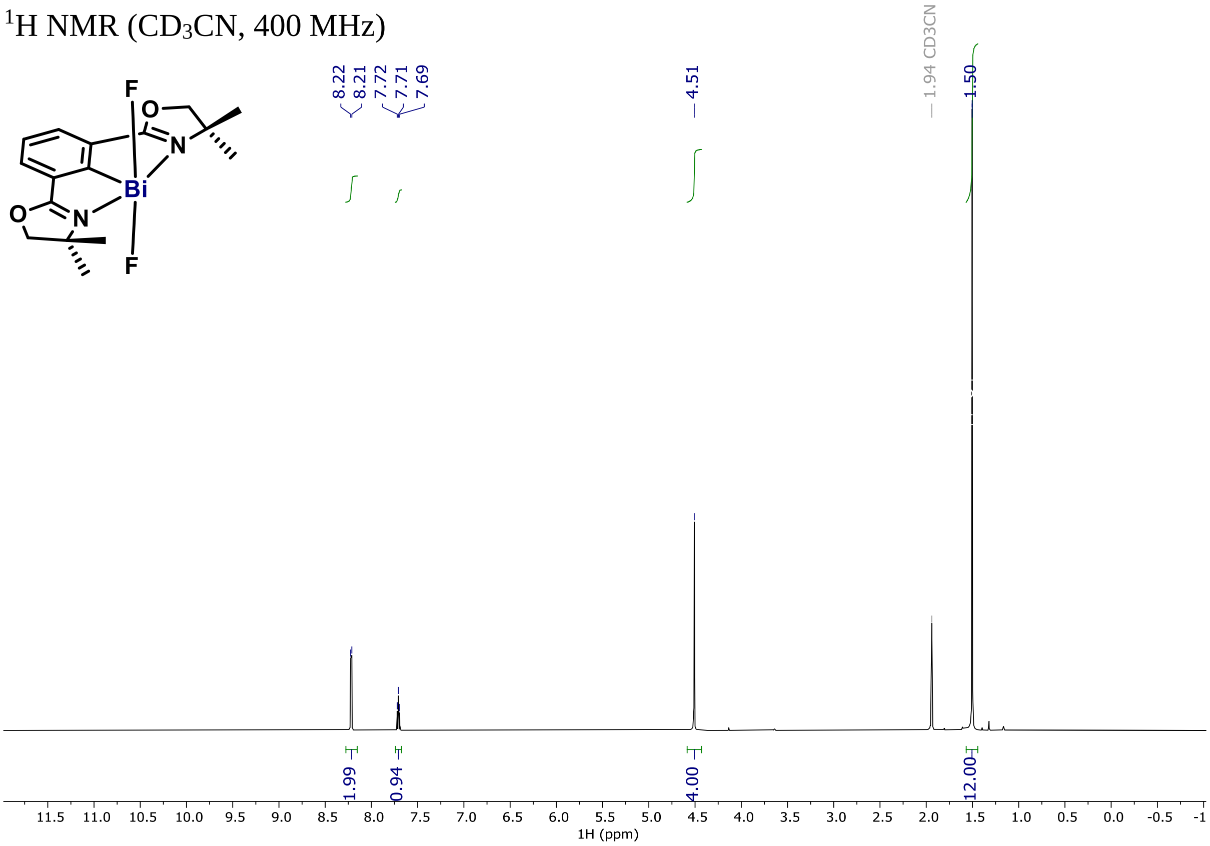

^{13}C NMR (CD$_3$CN, 151 MHz)

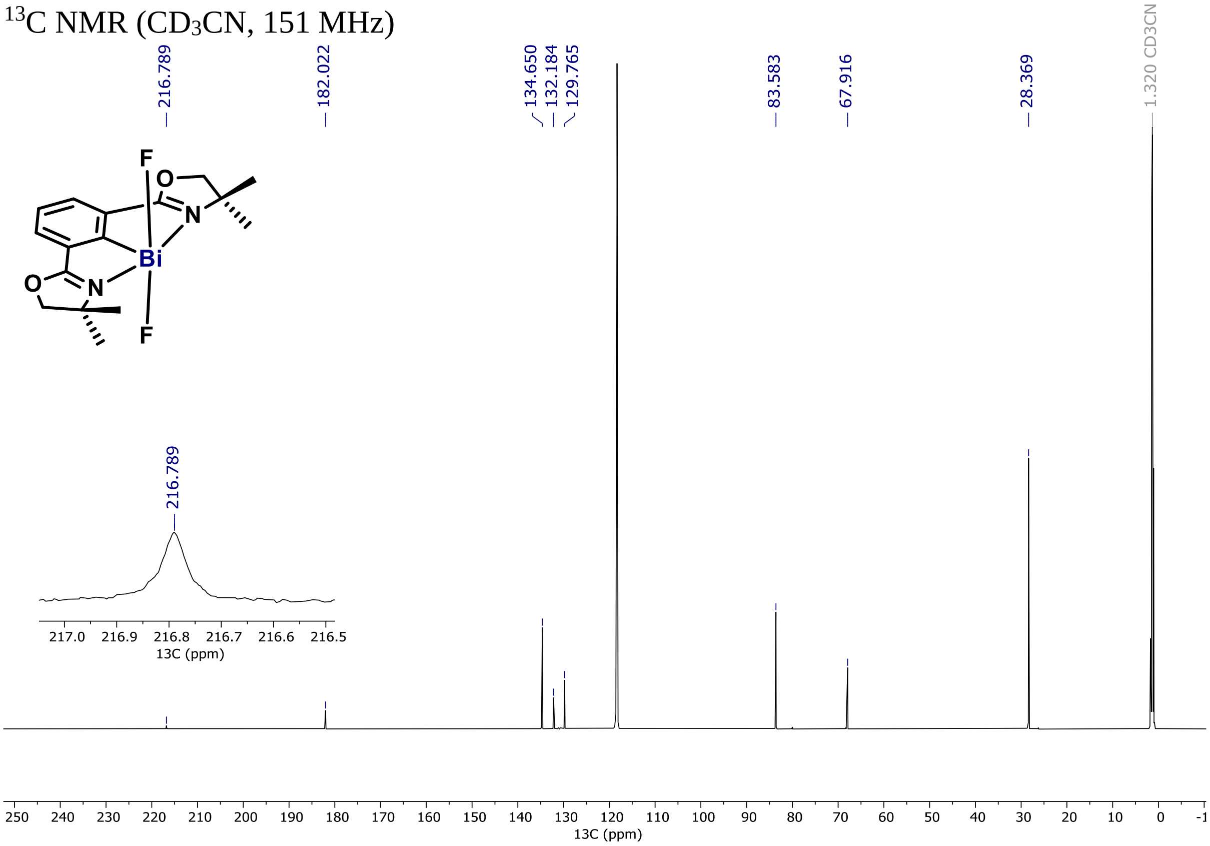

^{19}F NMR (CD_3CN, 565 MHz)

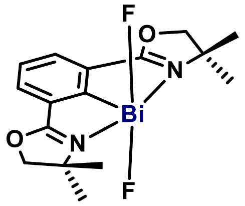

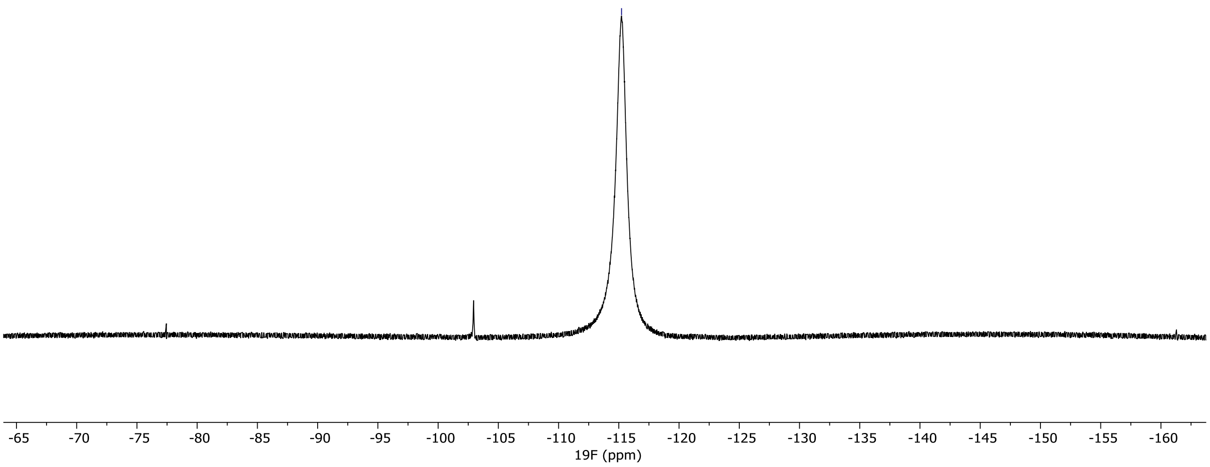

Phebox-Bi(4-tetrafluoropyridyl) triflate (**8b**)

^{1}H NMR (THF-d_8, 600 MHz)

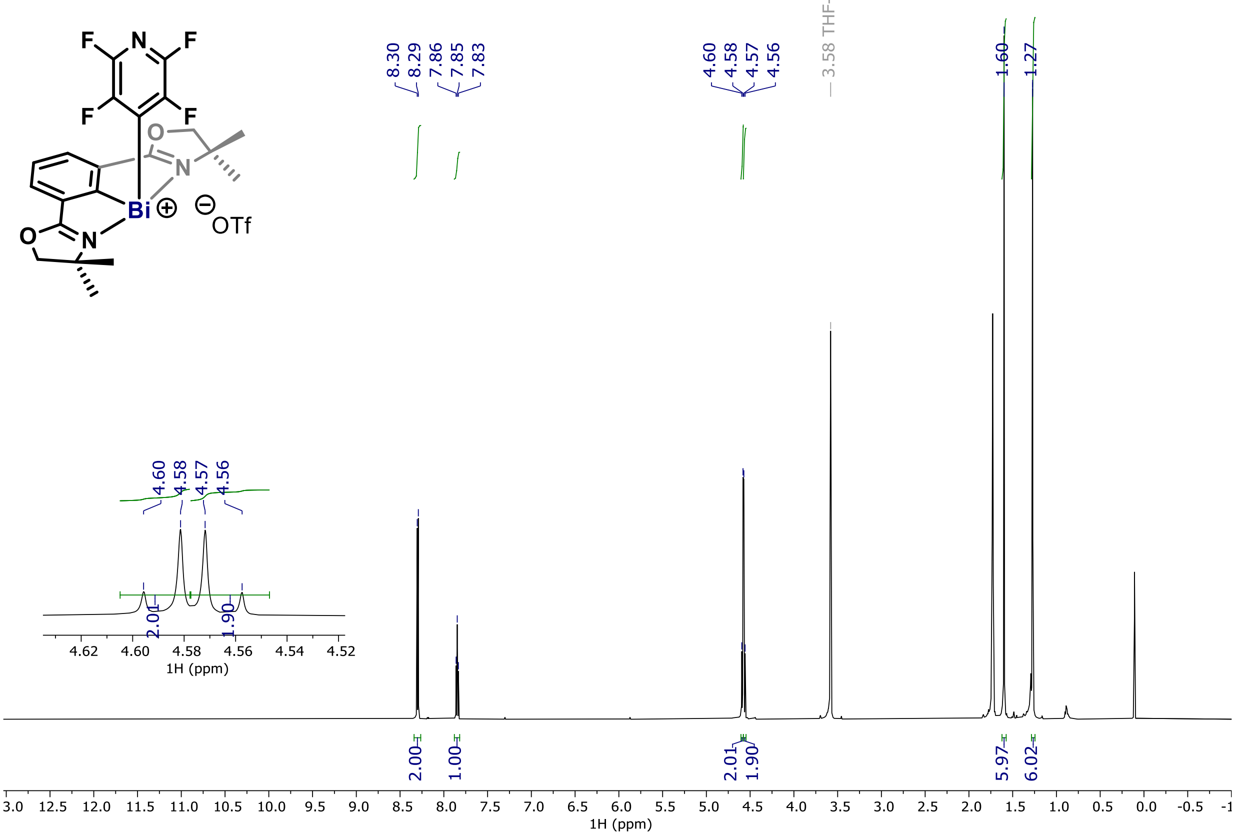

^{13}C NMR (THF-d_8, 151 MHz)

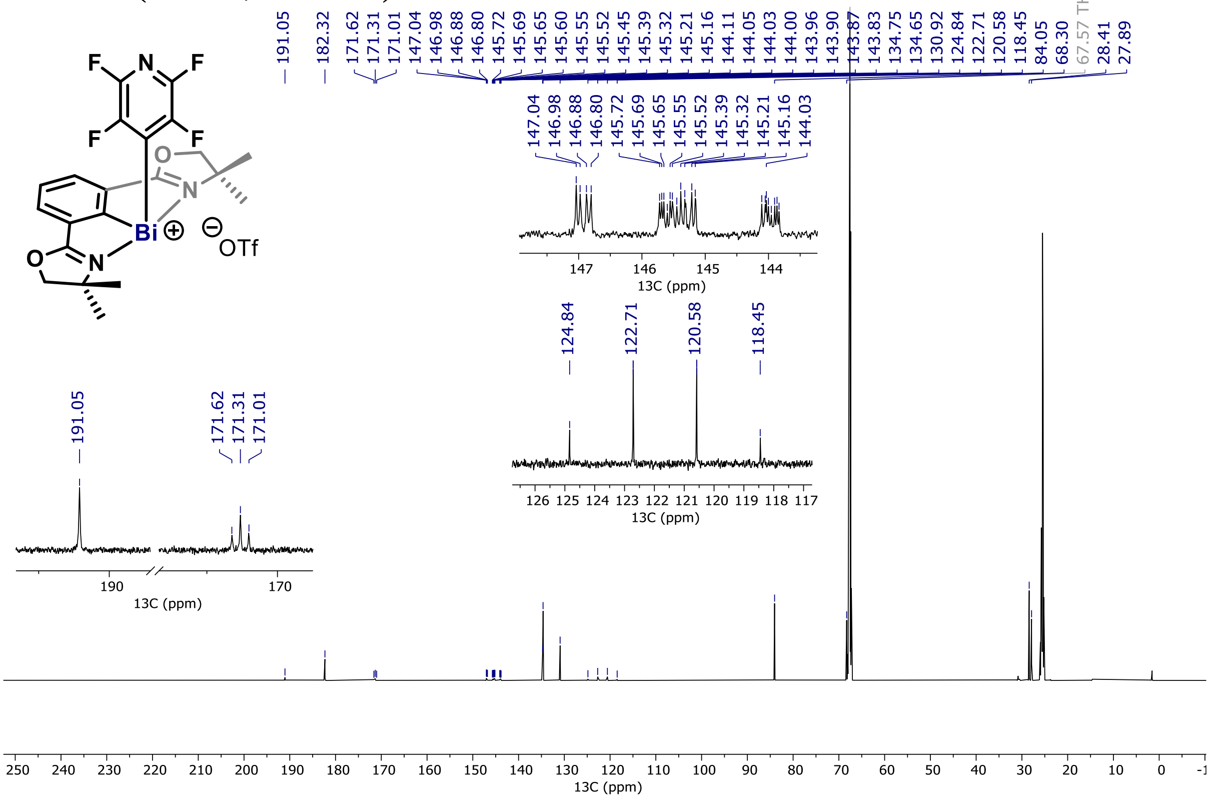

^{19}F NMR (THF-d_8, 565 MHz)

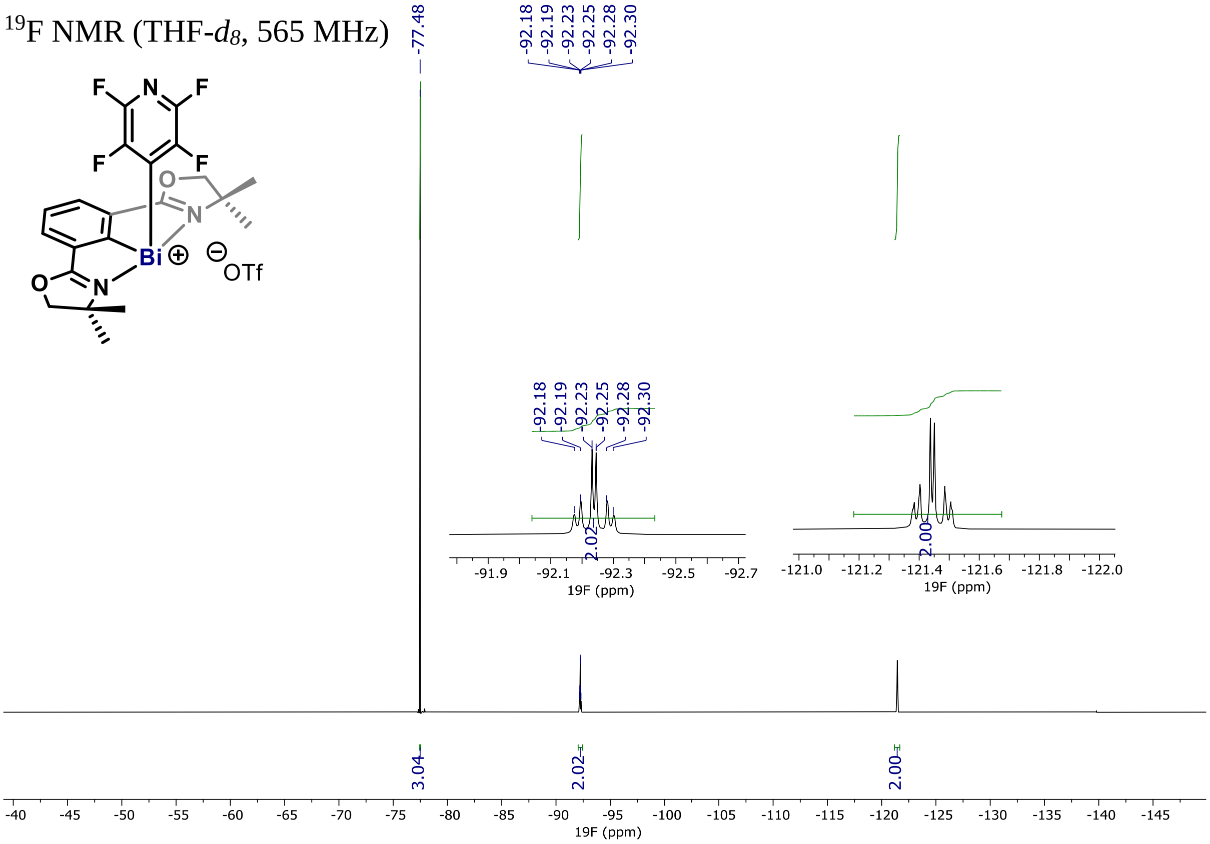

HSQC

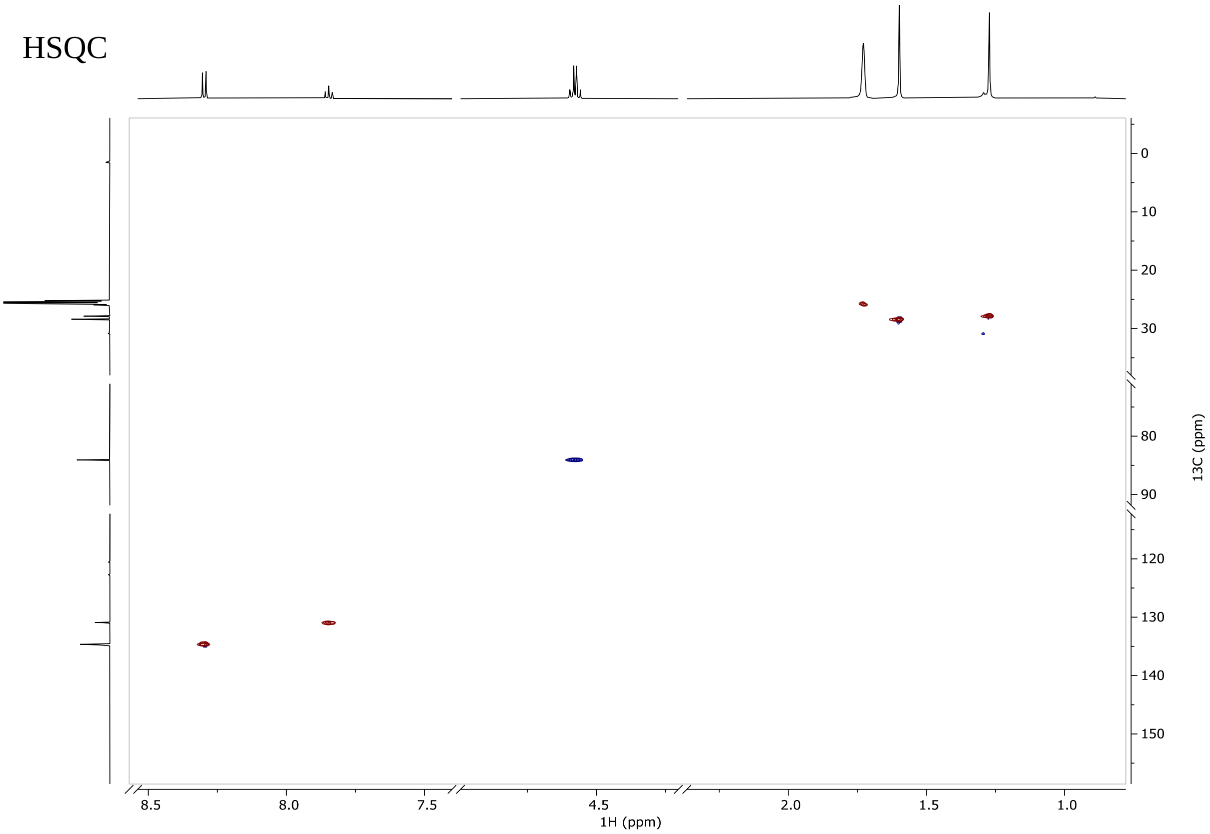

HMBC

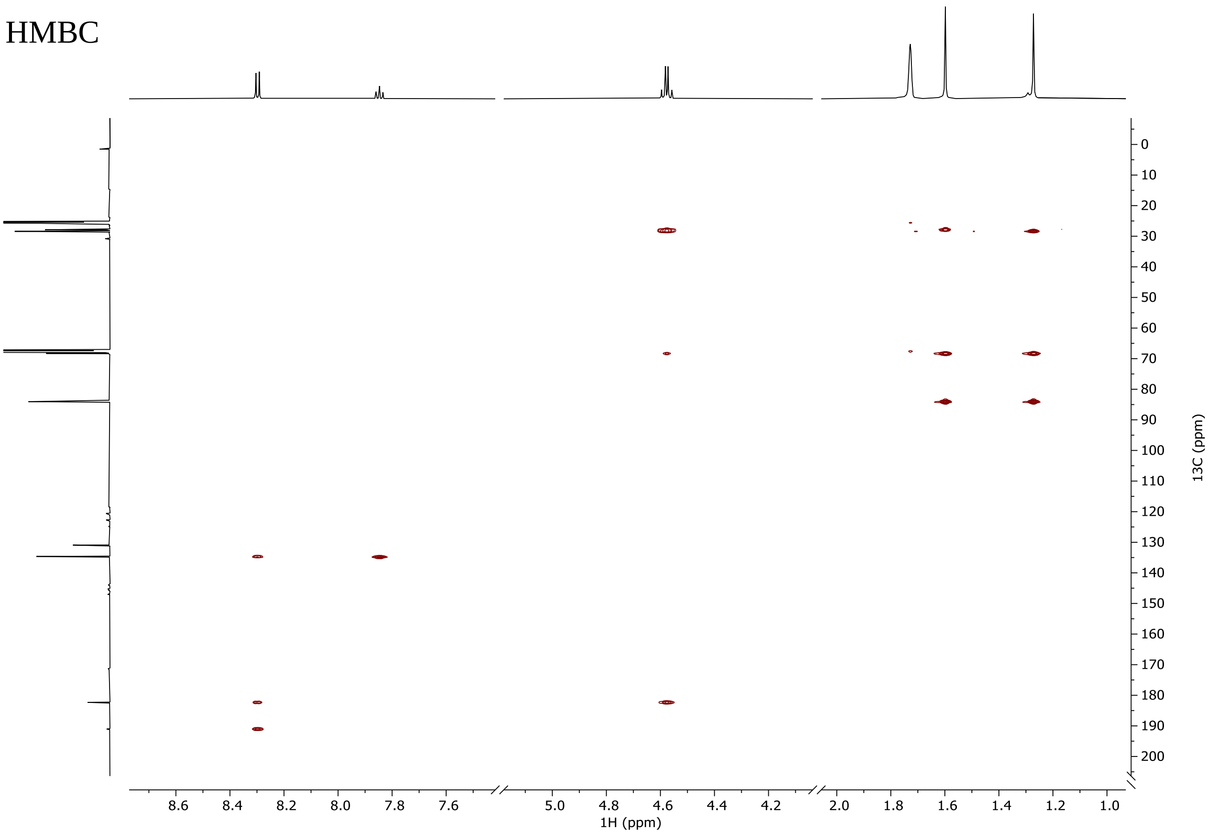

6.2. ^{19}F NMR Spectra for Determining the NMR Yields of HDF Reactions

HDF of hexafluorobenzene (**1a**) using **5** as catalyst

^{19}F NMR (THF with a sealed glass capillary containing C_6D_6, 470 MHz)

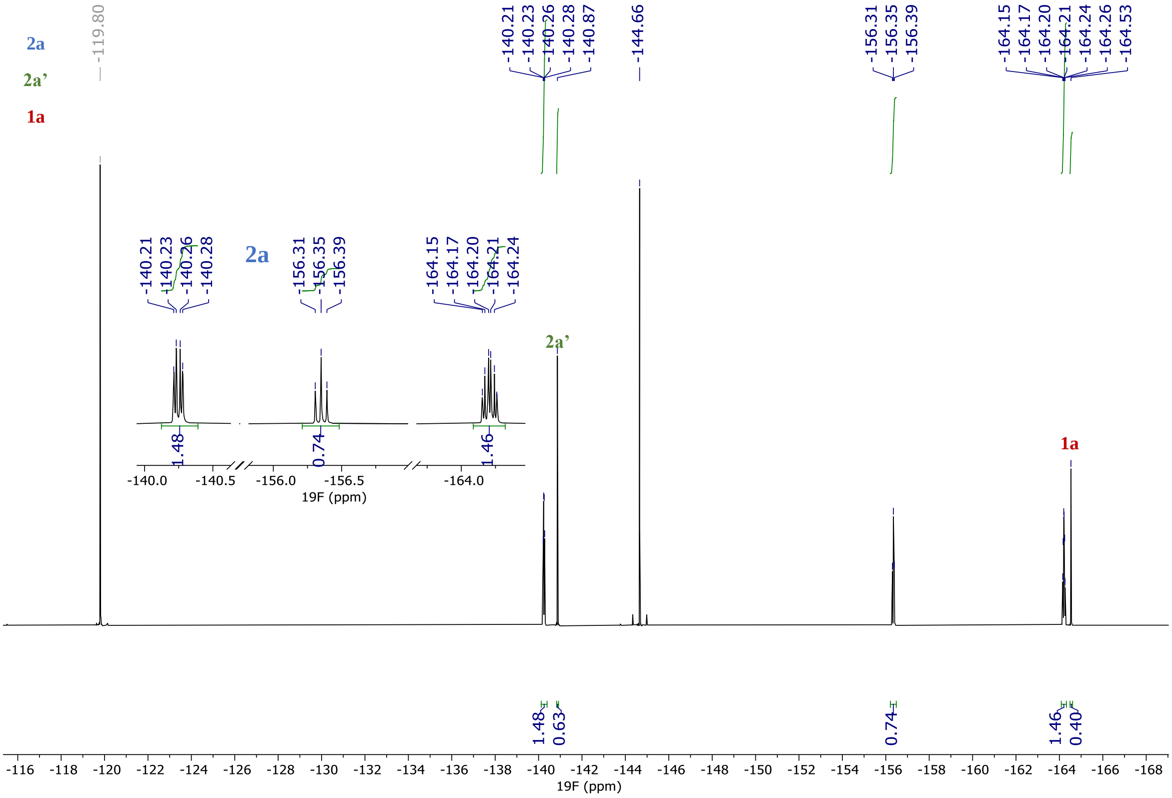

HDF of pentafluoropyridine (**1b**)

^{19}F NMR (THF with a sealed glass capillary containing C_6D_6, 470 MHz)

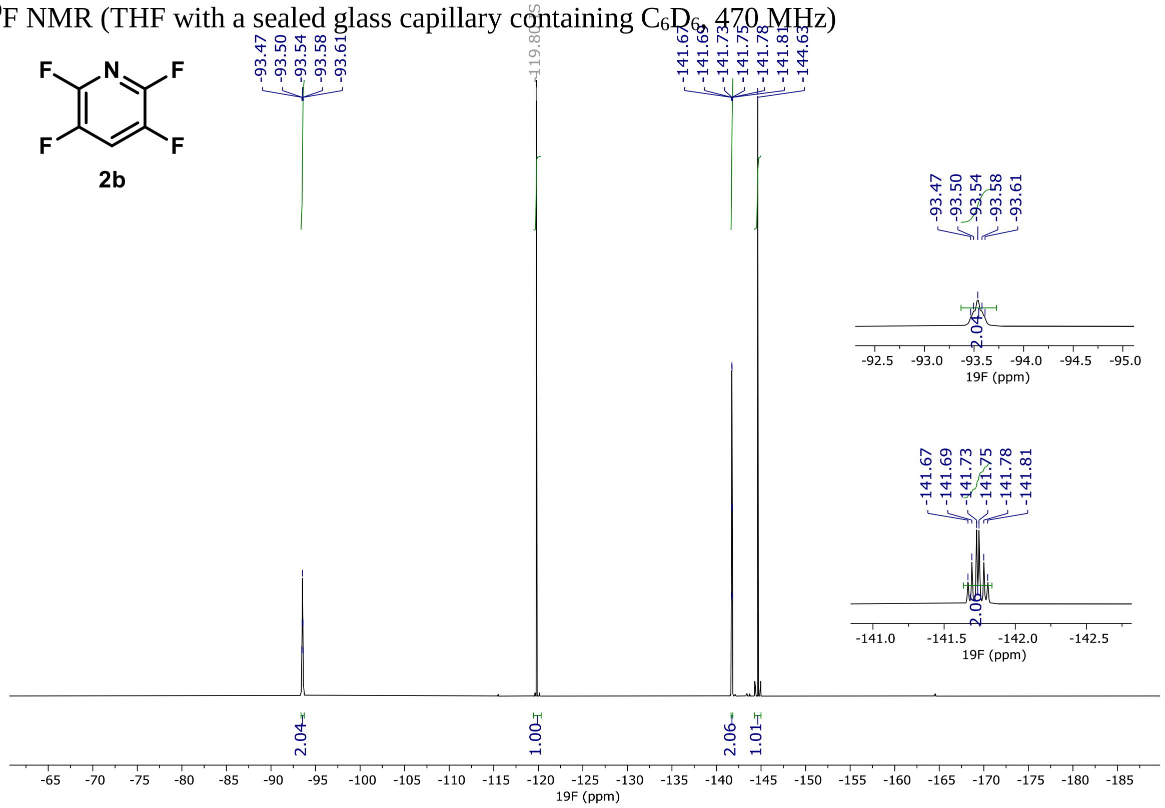

HDF of octafluorotoluene (**1c**)

^{19}F NMR (THF with a sealed glass capillary containing C_6D_6, 470 MHz)

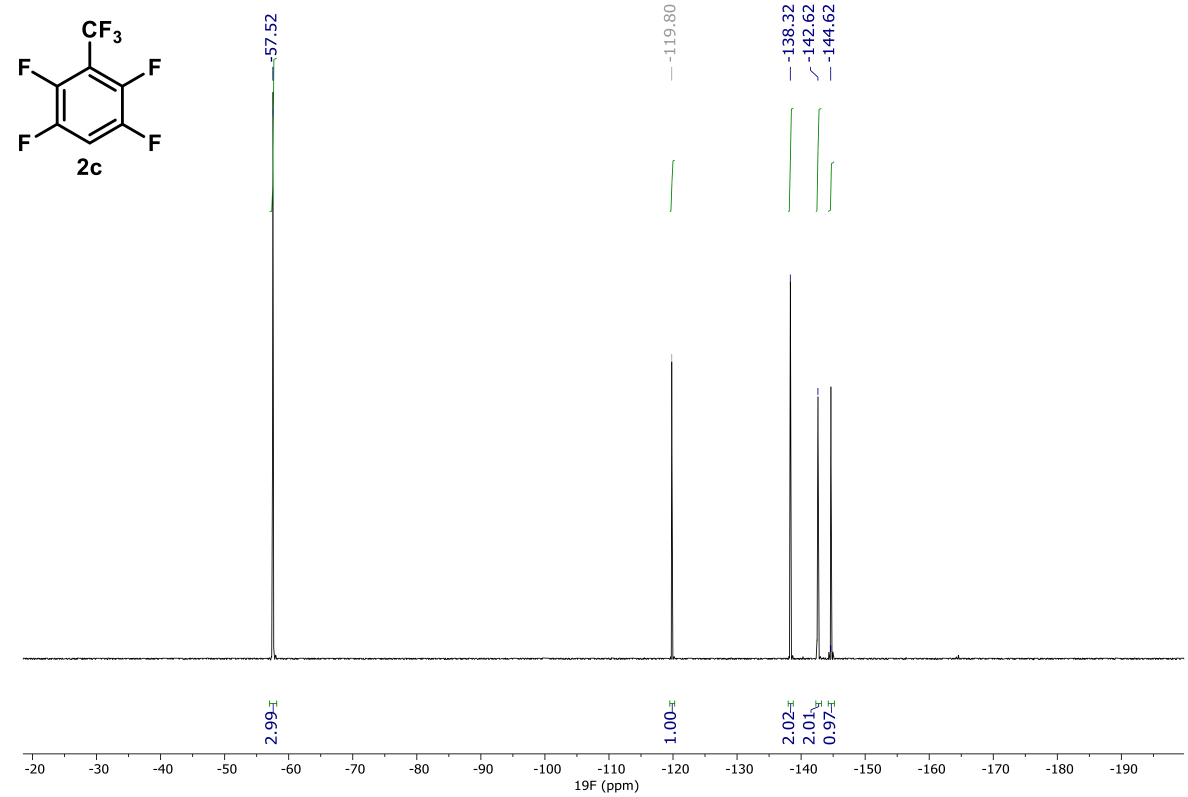

HDF of methyl 2,3,4,5,6-pentafluorobenzoate (**1d**)

^{19}F NMR (THF with a sealed glass capillary containing C_6D_6, 470 MHz)

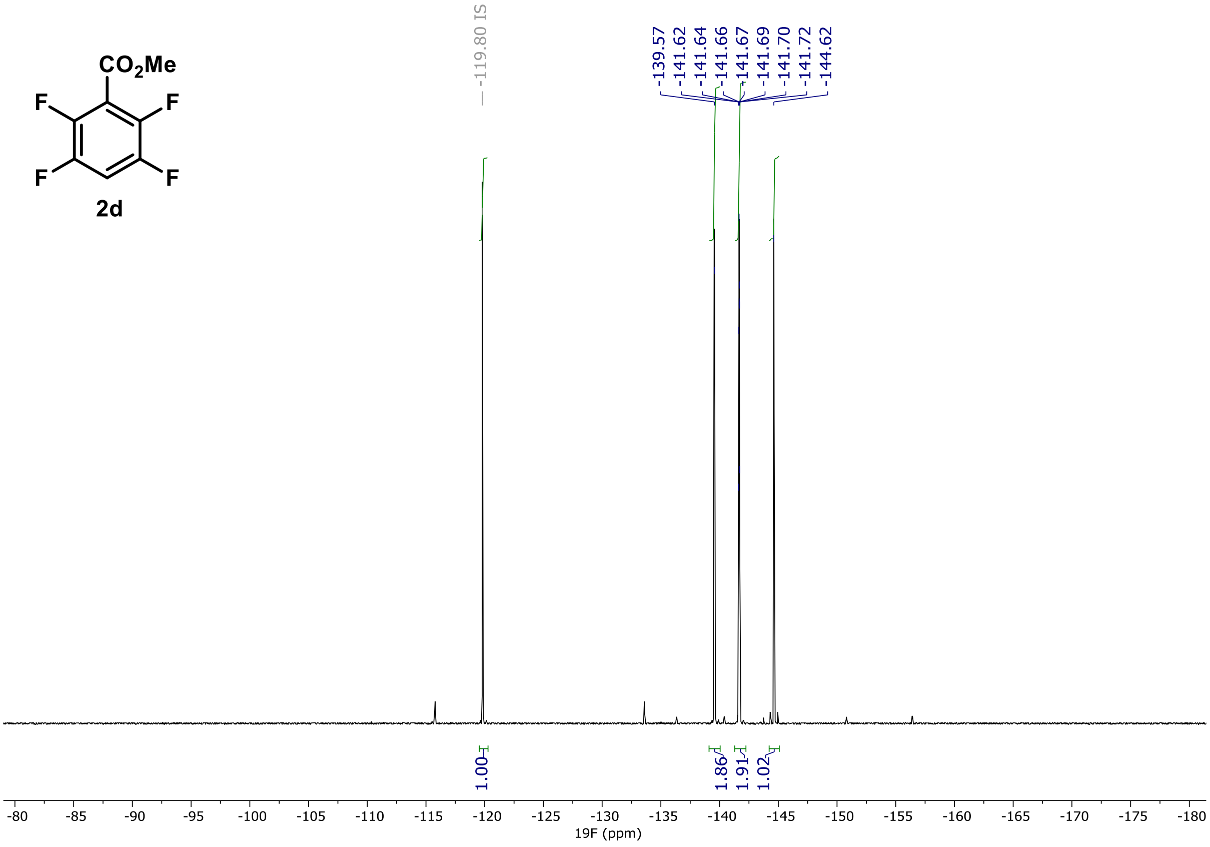

HDF of 2,3,4,5,6-pentafluorocyanobenzene (**1e**)

^{19}F NMR (THF with a sealed glass capillary containing C_6D_6, 470 MHz)

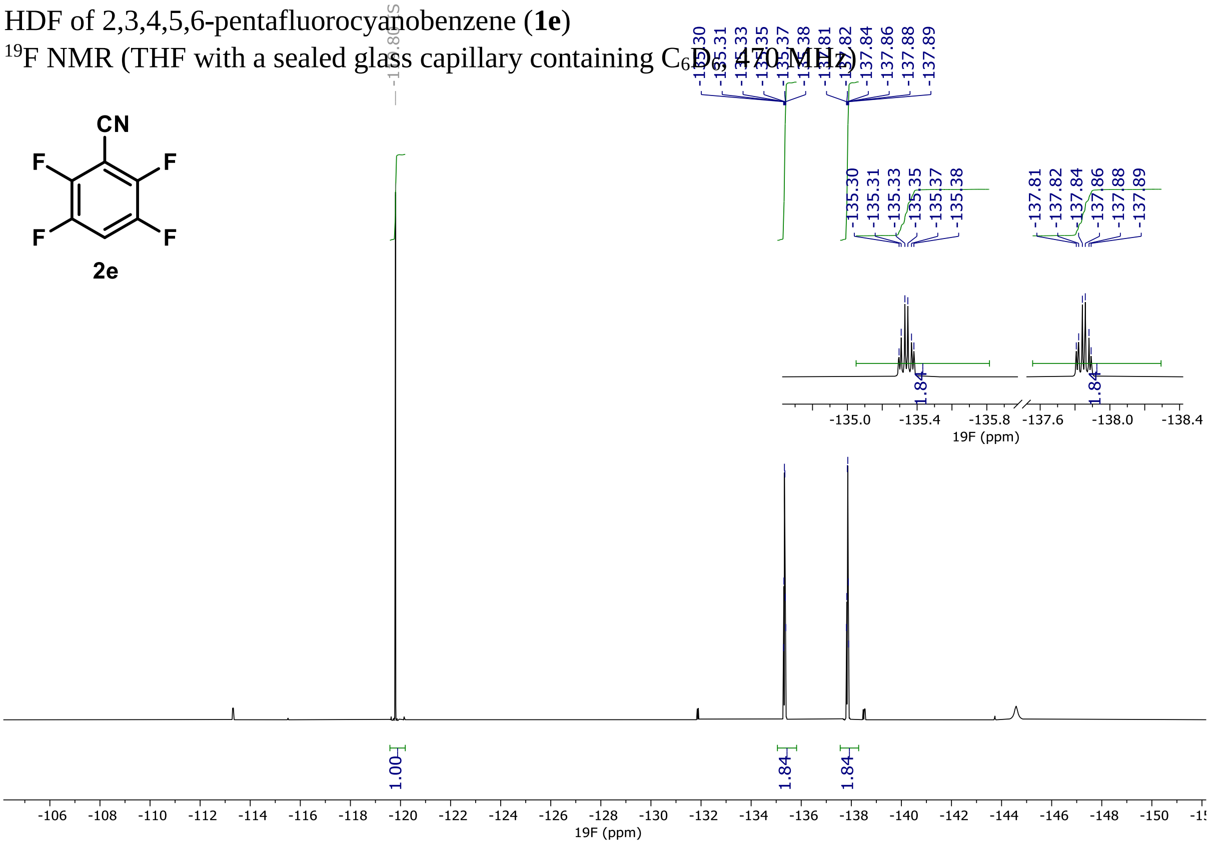

HDF of octafluoronaphthalene (**1h**)

^{19}F NMR (THF with a sealed glass capillary containing C_6D_6, 470 MHz)

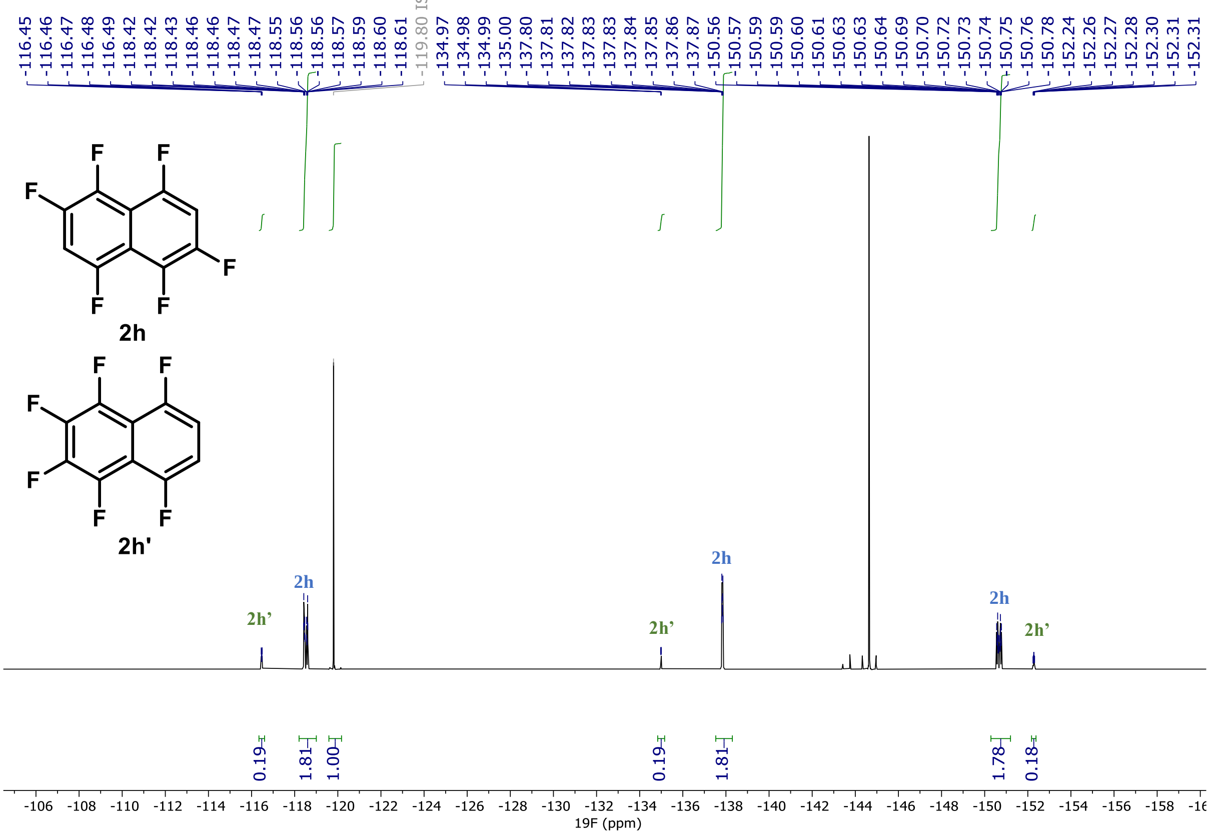

HDF of pentafluorobenzene (**2a**)

^{19}F NMR (THF with a sealed glass capillary containing C_6D_6, 470 MHz)

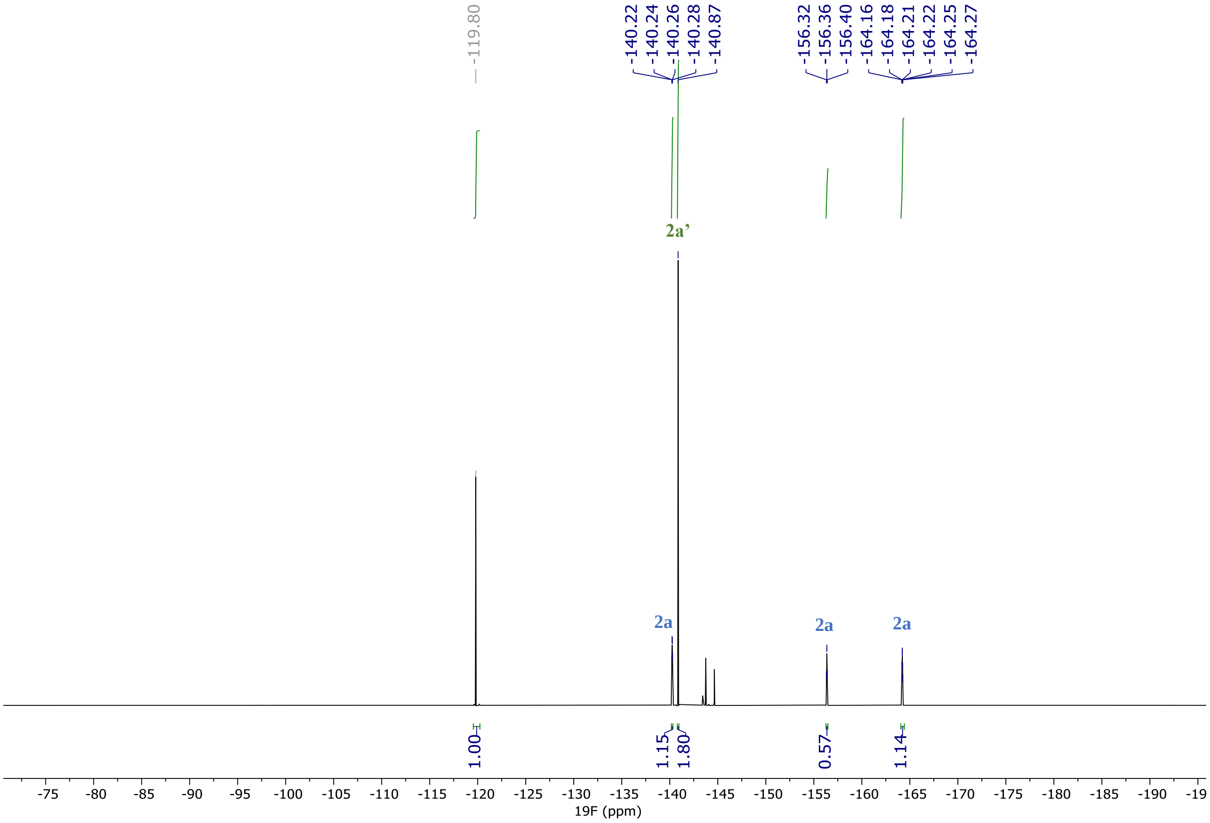

HDF of 2,4,6-trifluorobenzonitrile (**1n**)

^{19}F NMR (THF with a sealed glass capillary containing C_6D_6, 470 MHz)

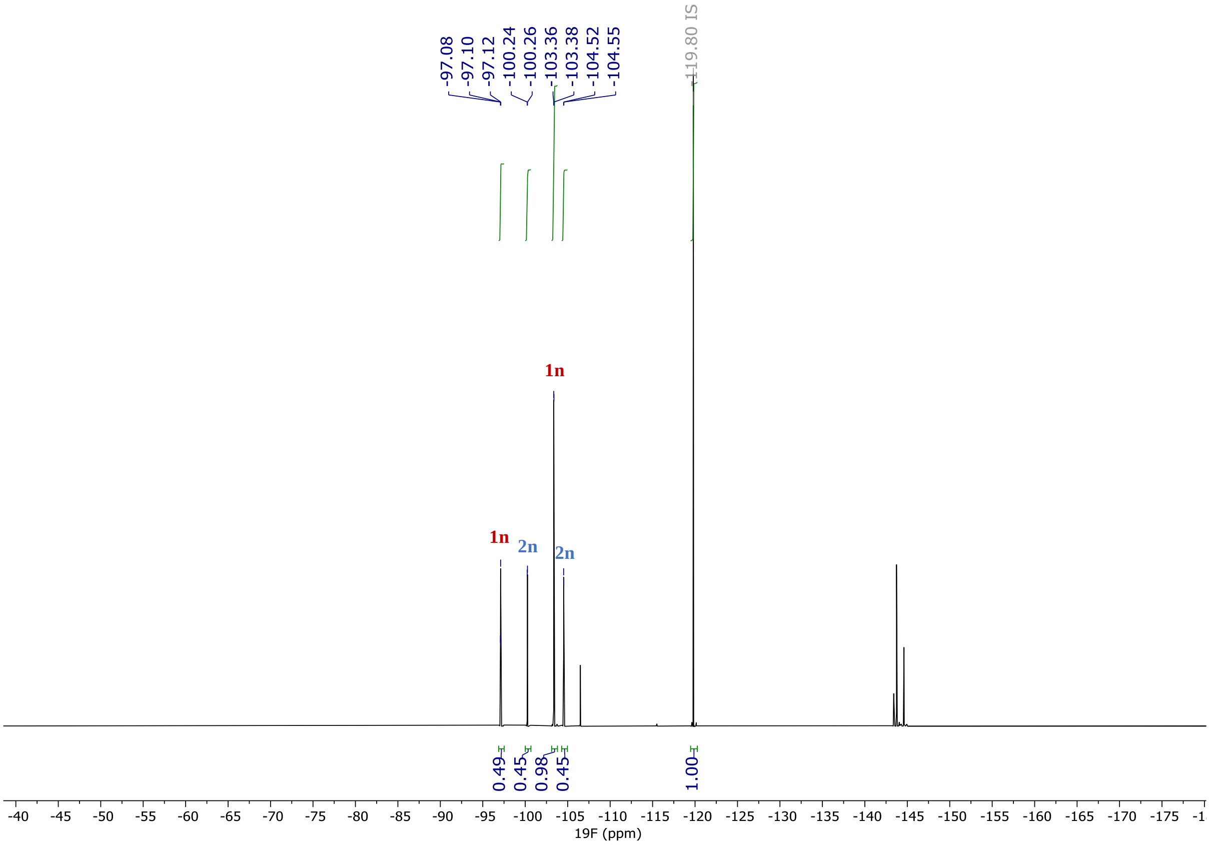

6.3. NMR Spectra of the Isolated HDF Products

2,5-Difluoroisophthalonitrile (**2f**)

^{1}H NMR ($CDCl_3$, 400 MHz)

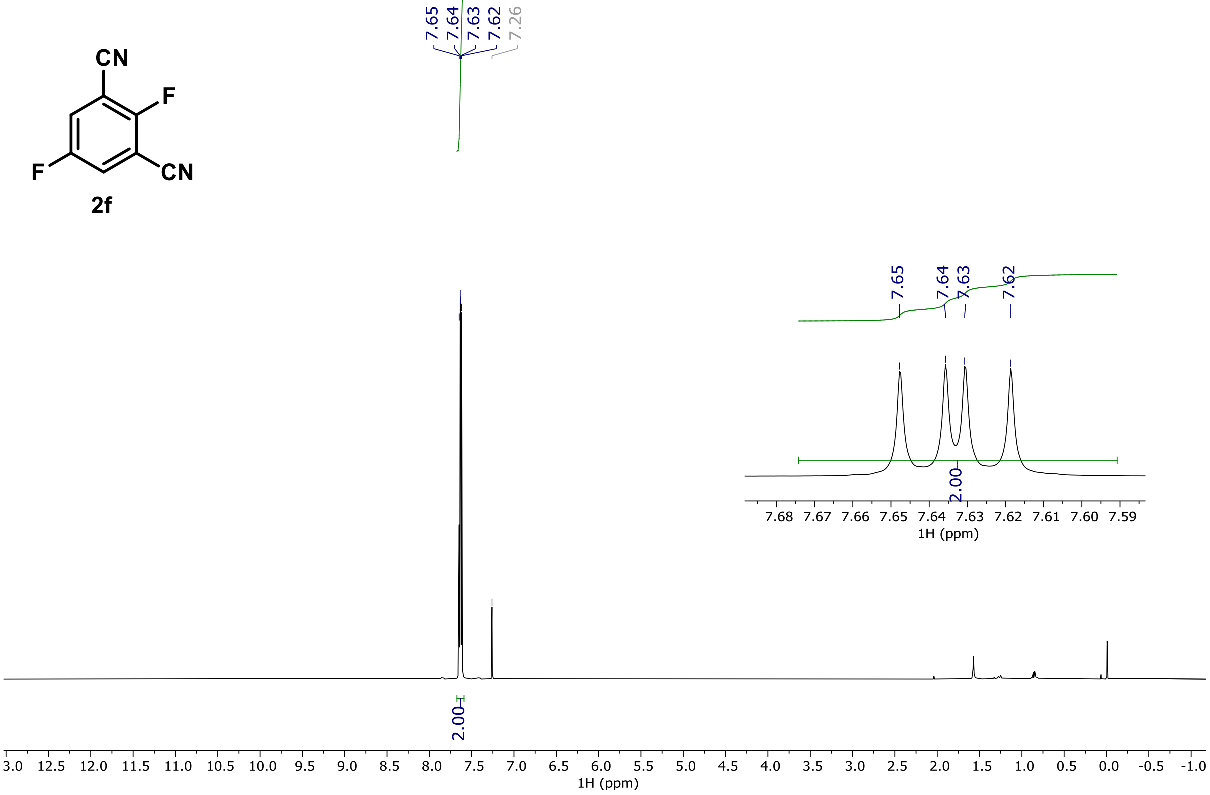

^{13}C NMR ($CDCl_3$, 101 MHz)

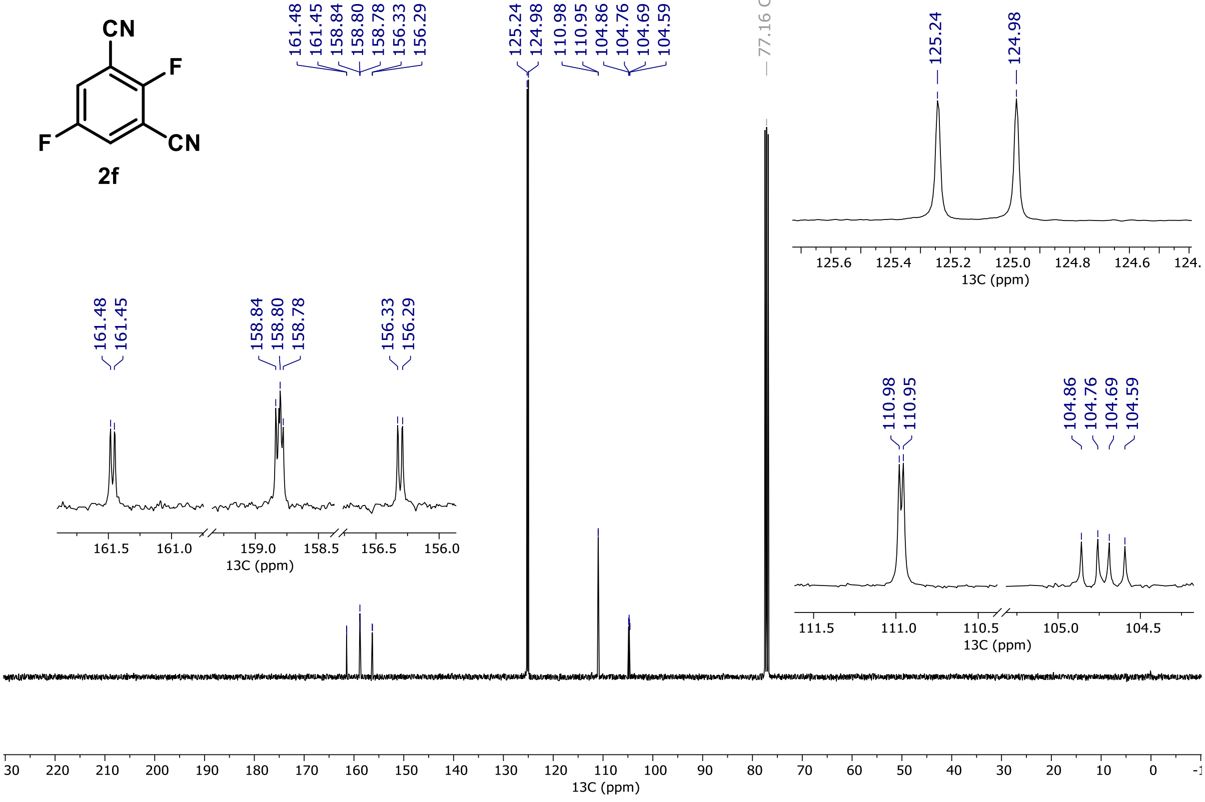

^{19}F NMR ($CDCl_3$, 282 MHz)

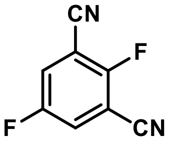

2f

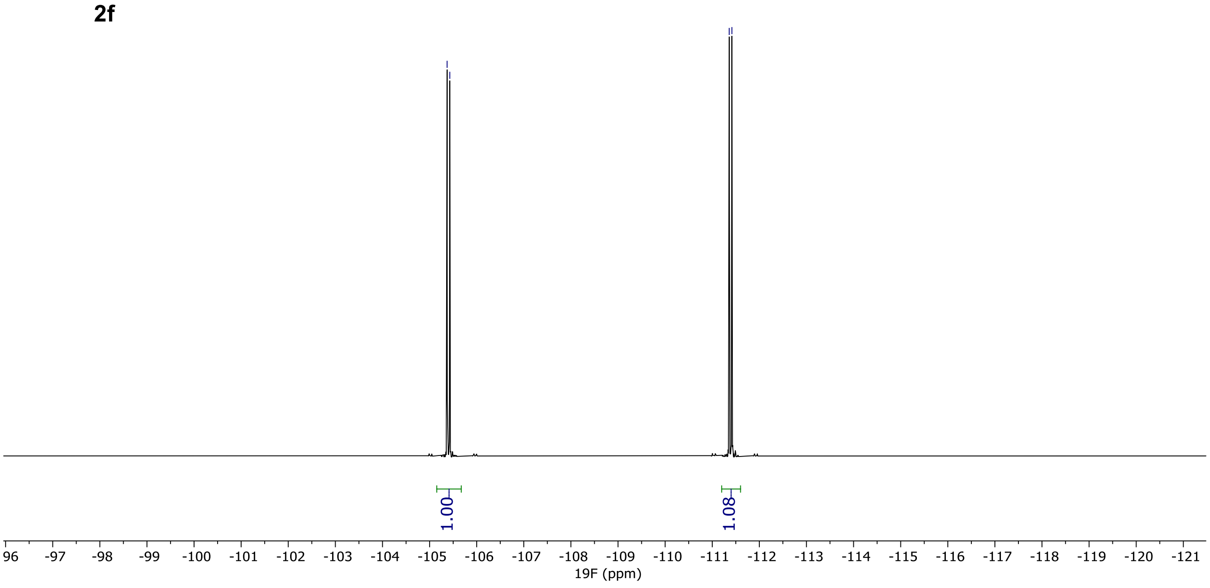

2,2',3,3',5,5',6,6'-Octafluoro-1,1'-biphenyl (**2g**)

^{1}H NMR ($CDCl_3$, 400 MHz)

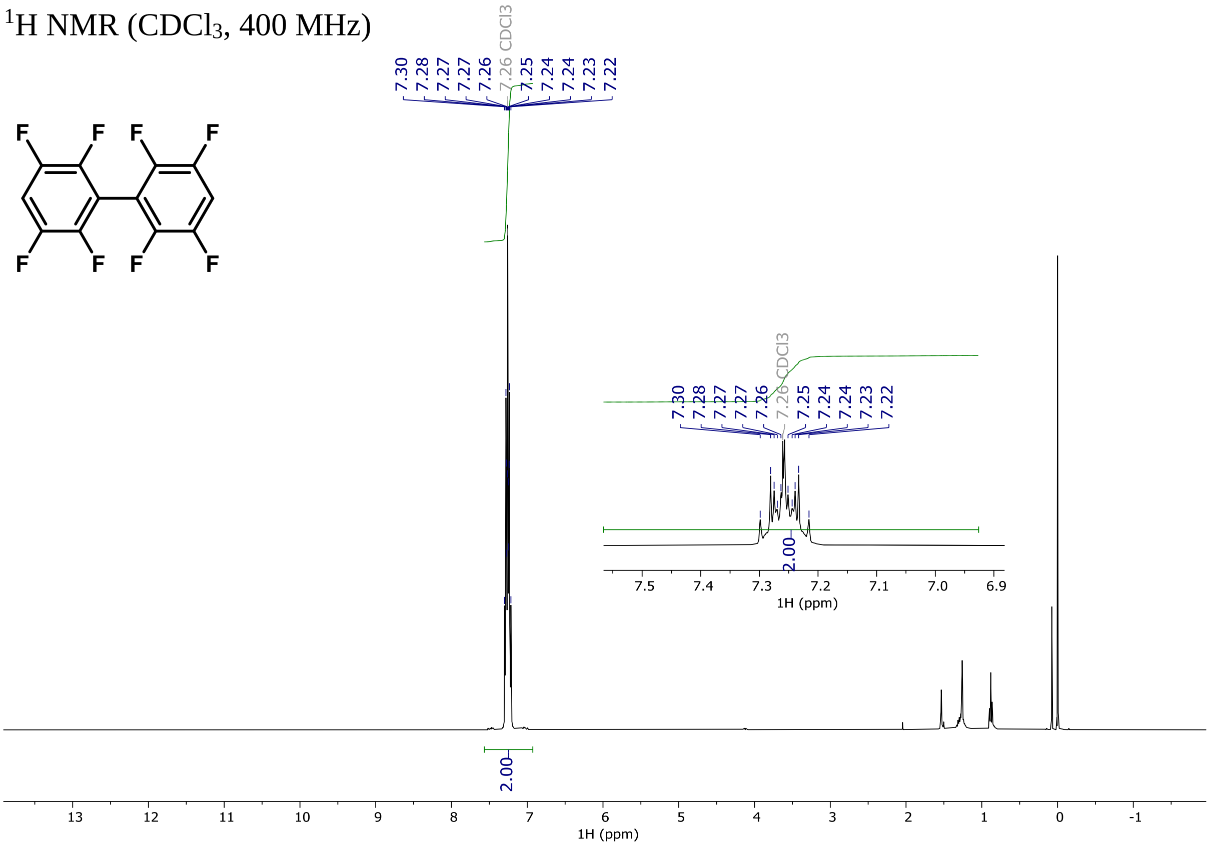

^{13}C NMR ($CDCl_3$, 151 MHz)

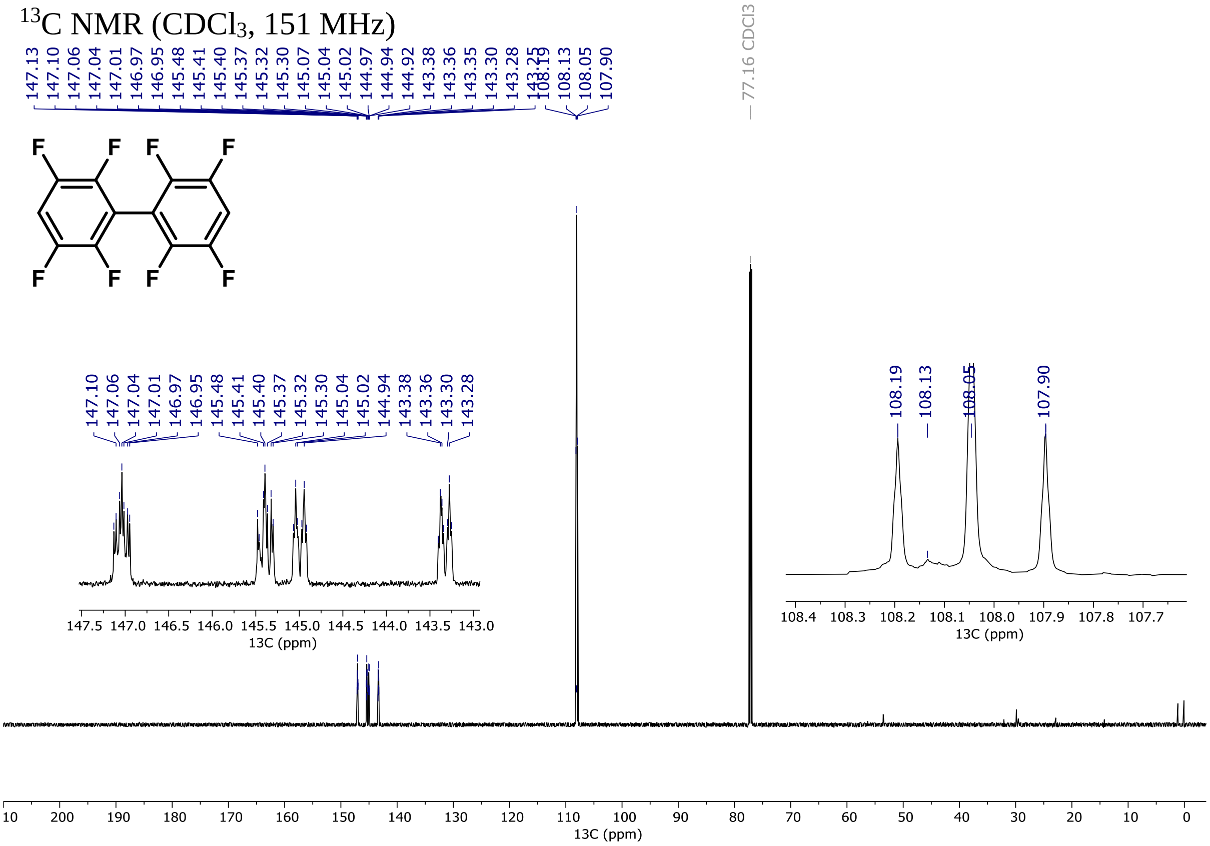

$^{13}C\{^{19}F\}$ NMR ($CDCl_3$, 151 MHz)

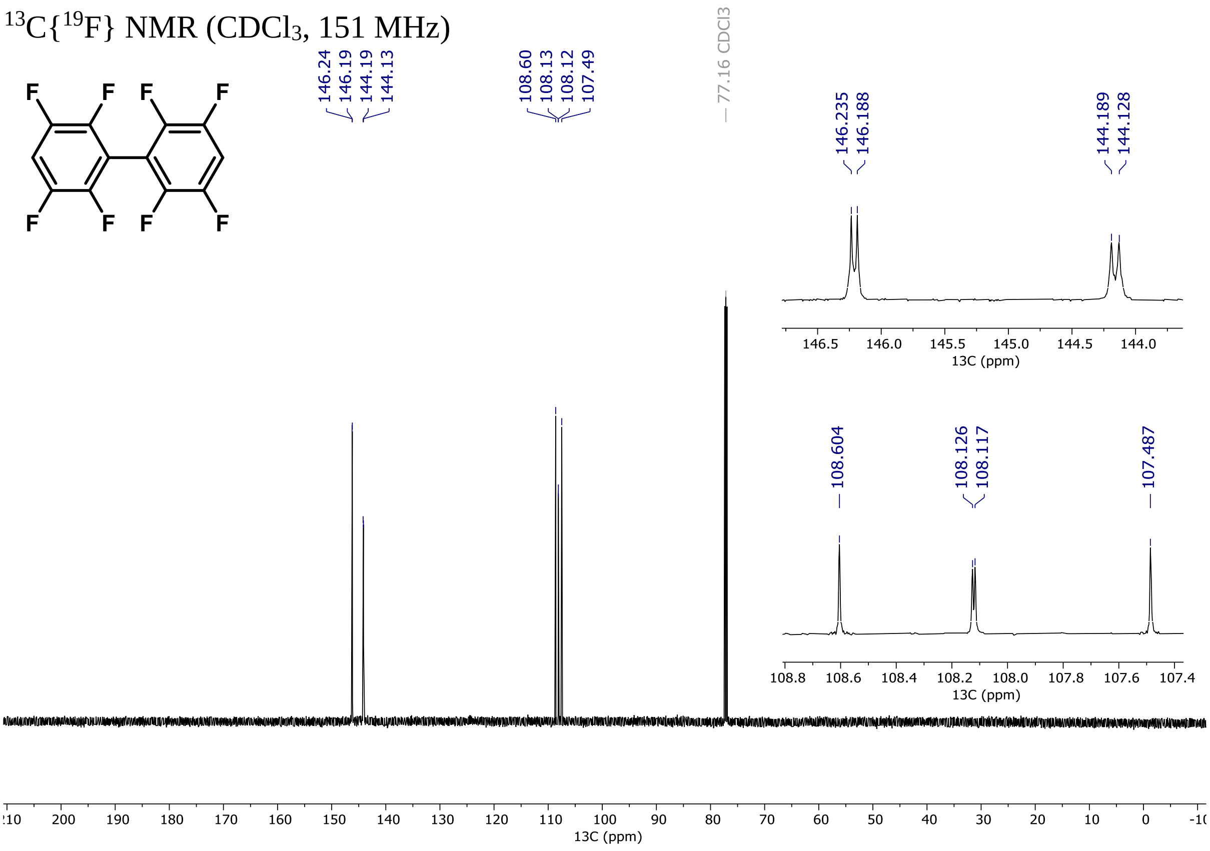

^{19}F NMR ($CDCl_3$, 565 MHz)

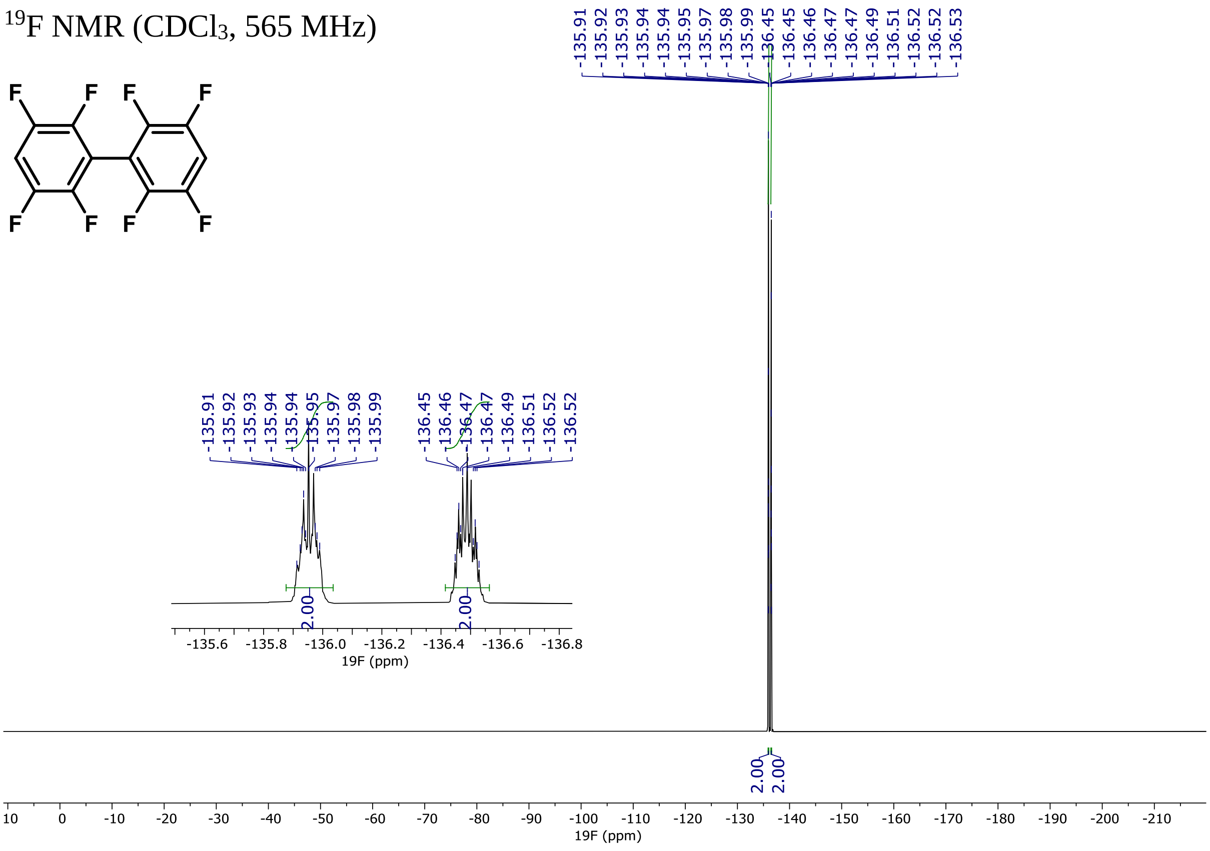

Diphenyl(2,3,5,6-tetrafluorophenyl)phosphane (**2i**)

^{1}H NMR ($CDCl_3$, 400 MHz)

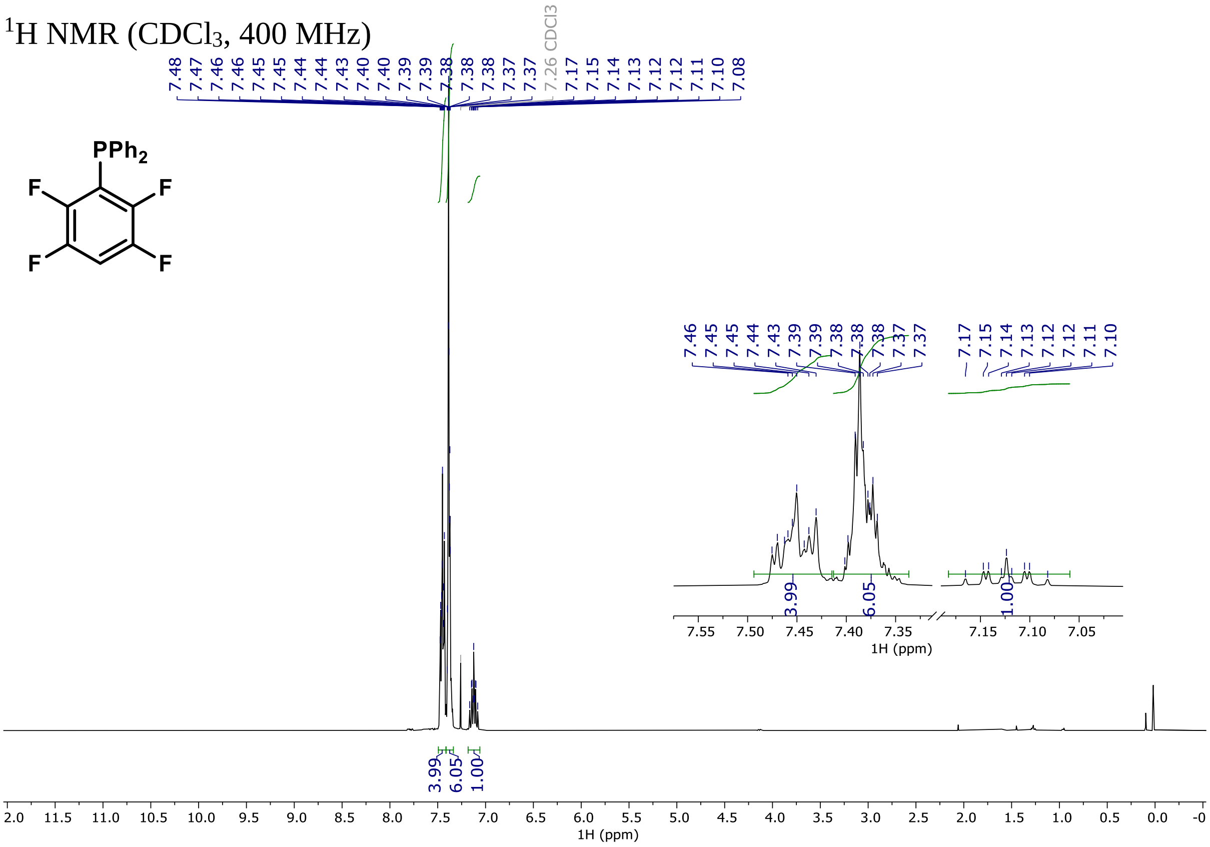

^{13}C NMR ($CDCl_3$, 101 MHz)

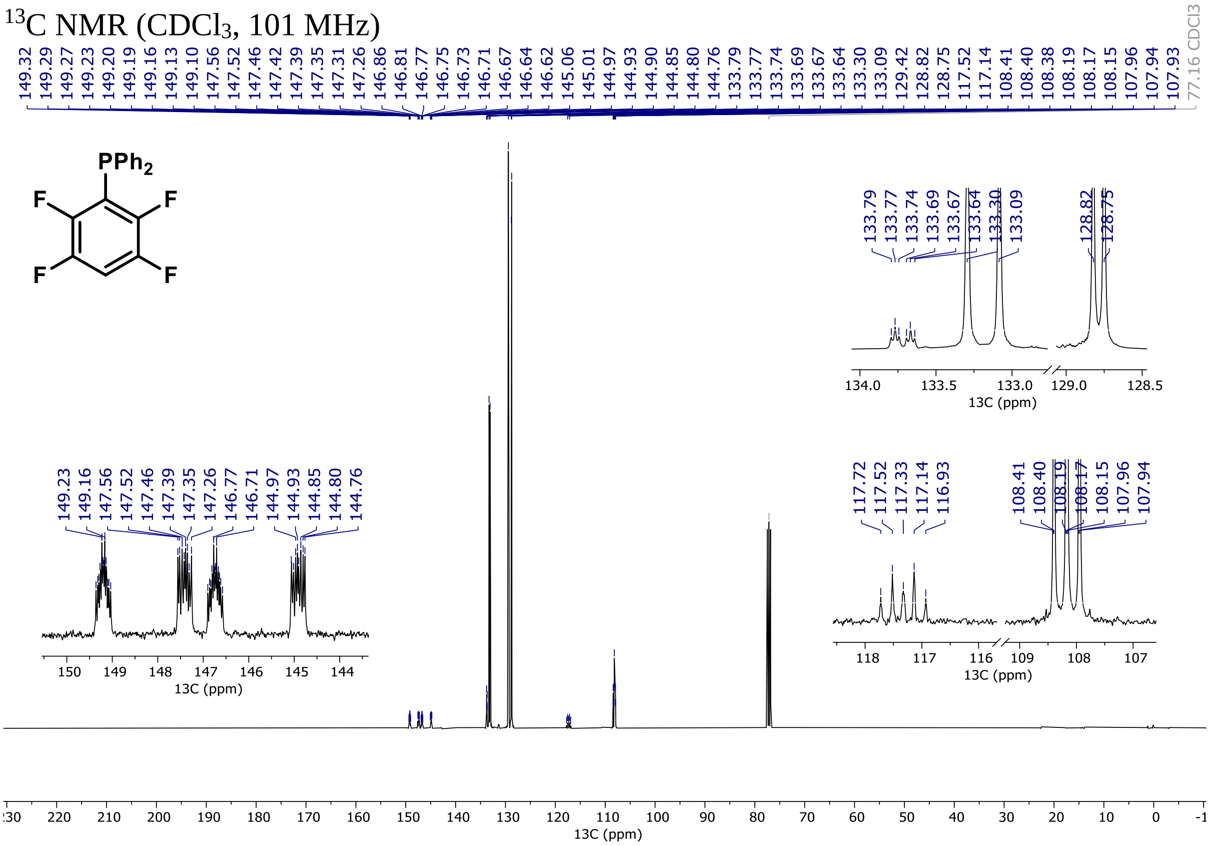

^{31}P NMR ($CDCl_3$, 162 MHz)

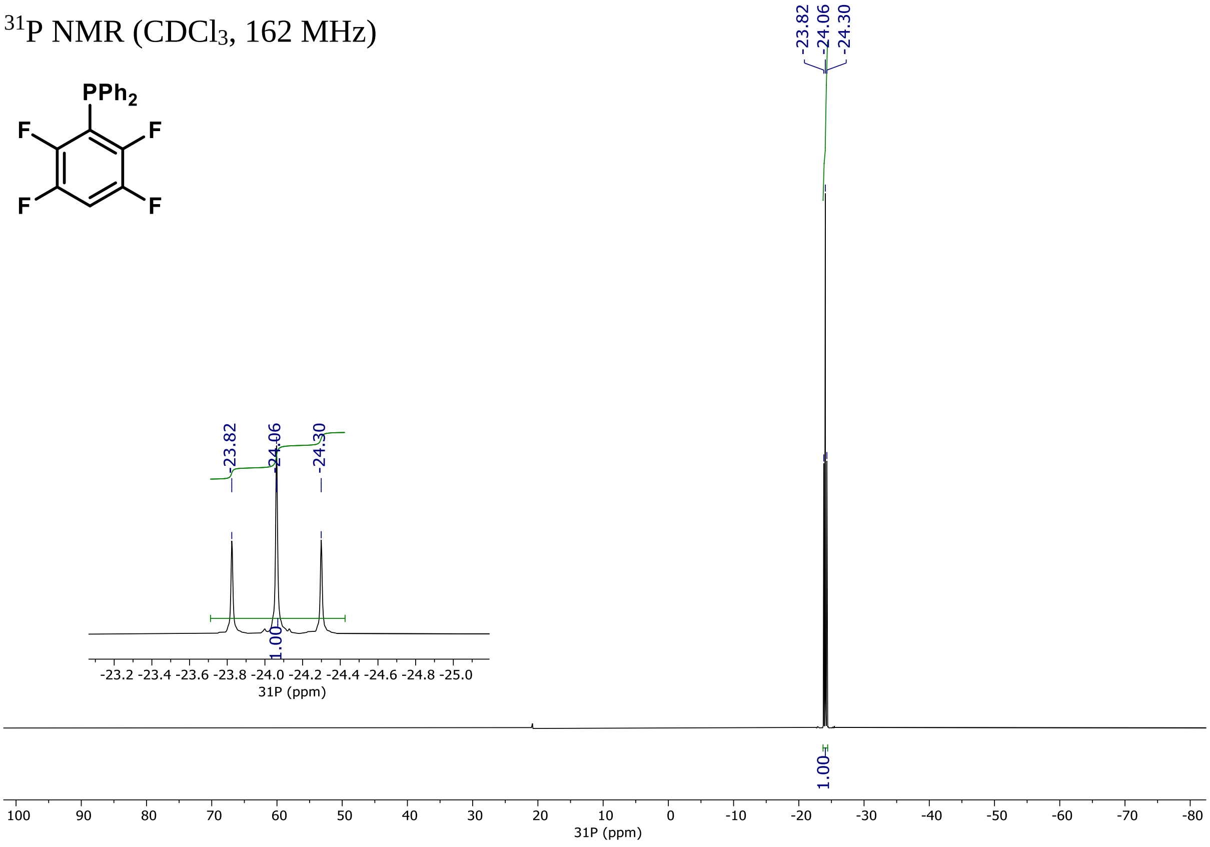

^{19}F NMR ($CDCl_3$, 282 MHz)

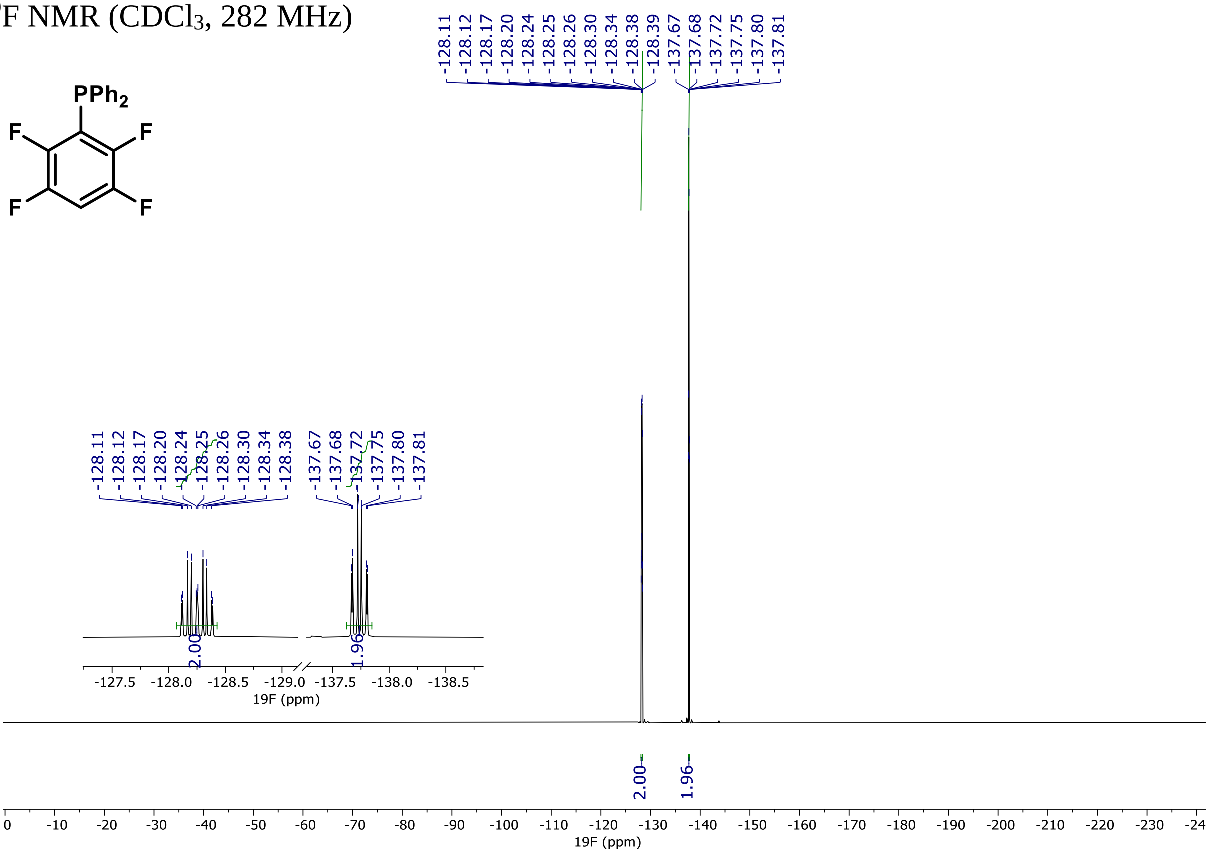

Tris(2,3,5,6-tetrafluorophenyl)phosphane (**2j**)

^{1}H NMR ($CDCl_3$, 400 MHz)

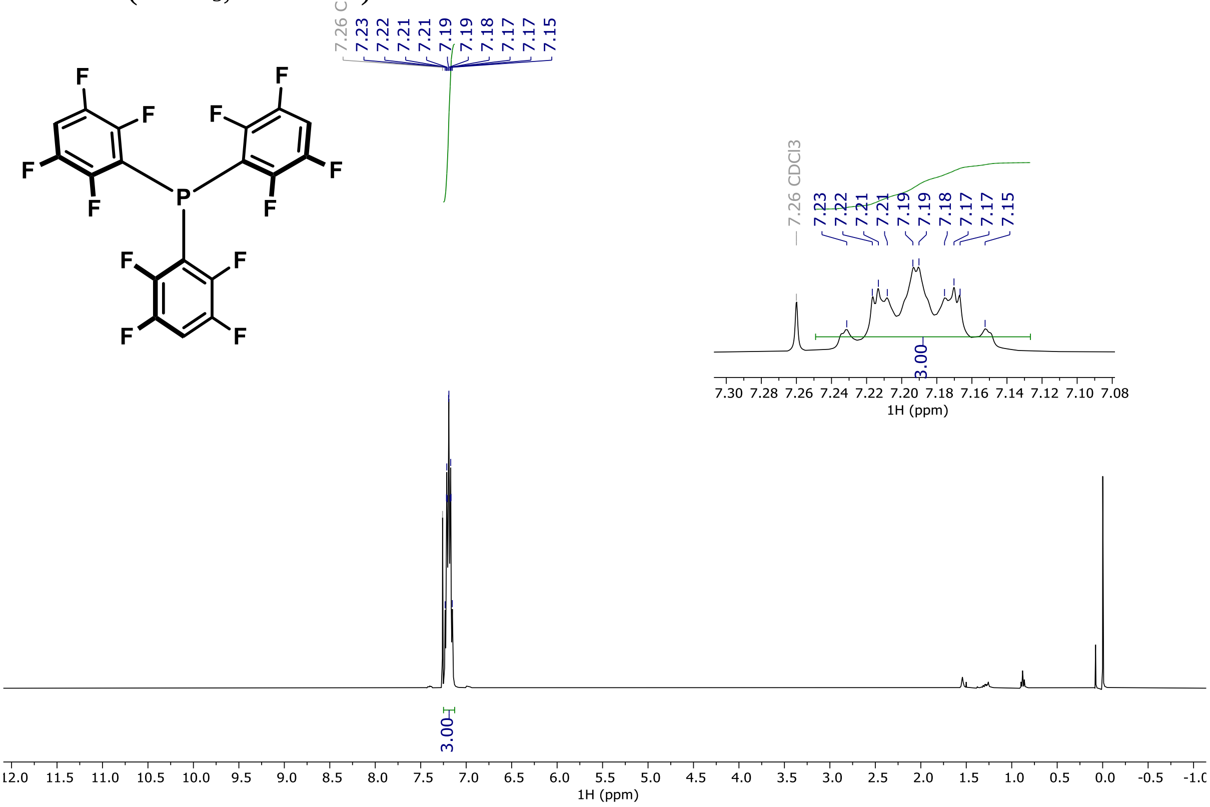

^{13}C NMR ($CDCl_3$, 101 MHz)

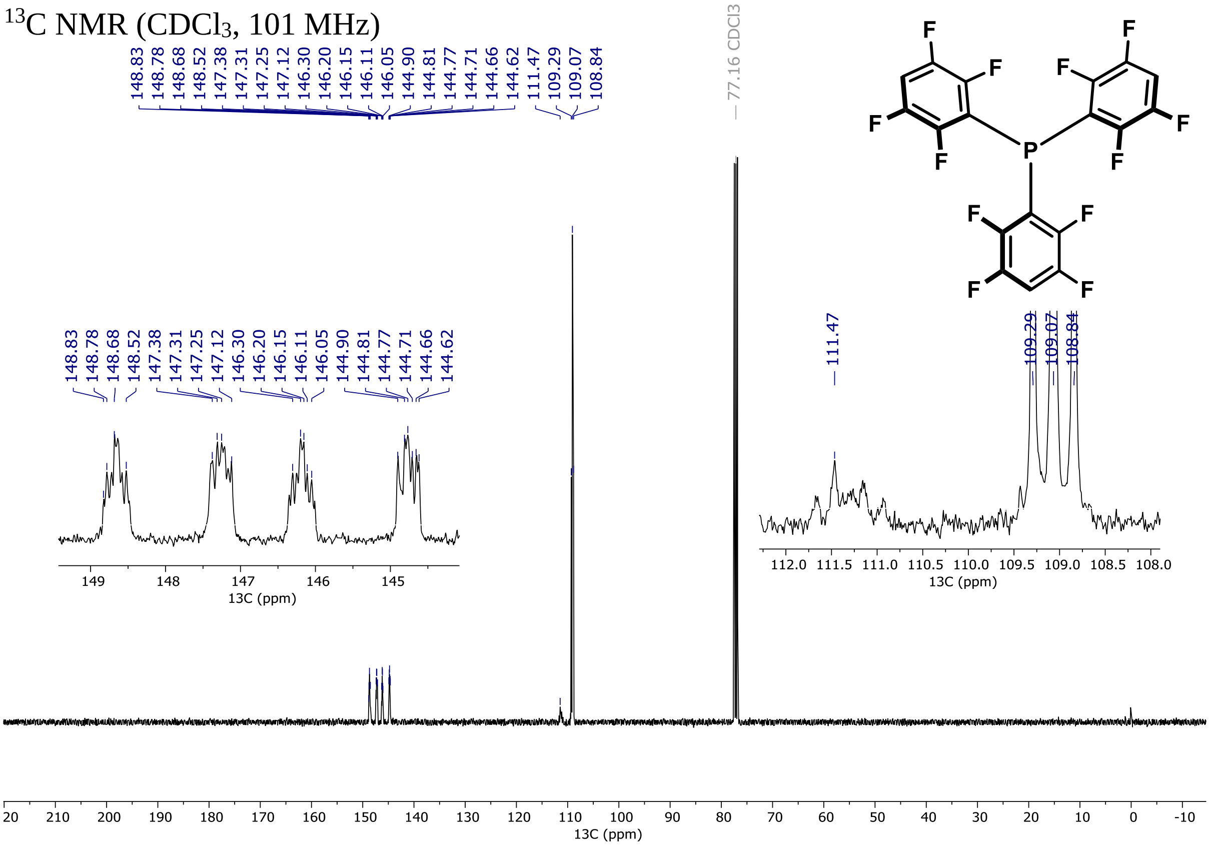

^{31}P NMR ($CDCl_3$, 162 MHz)

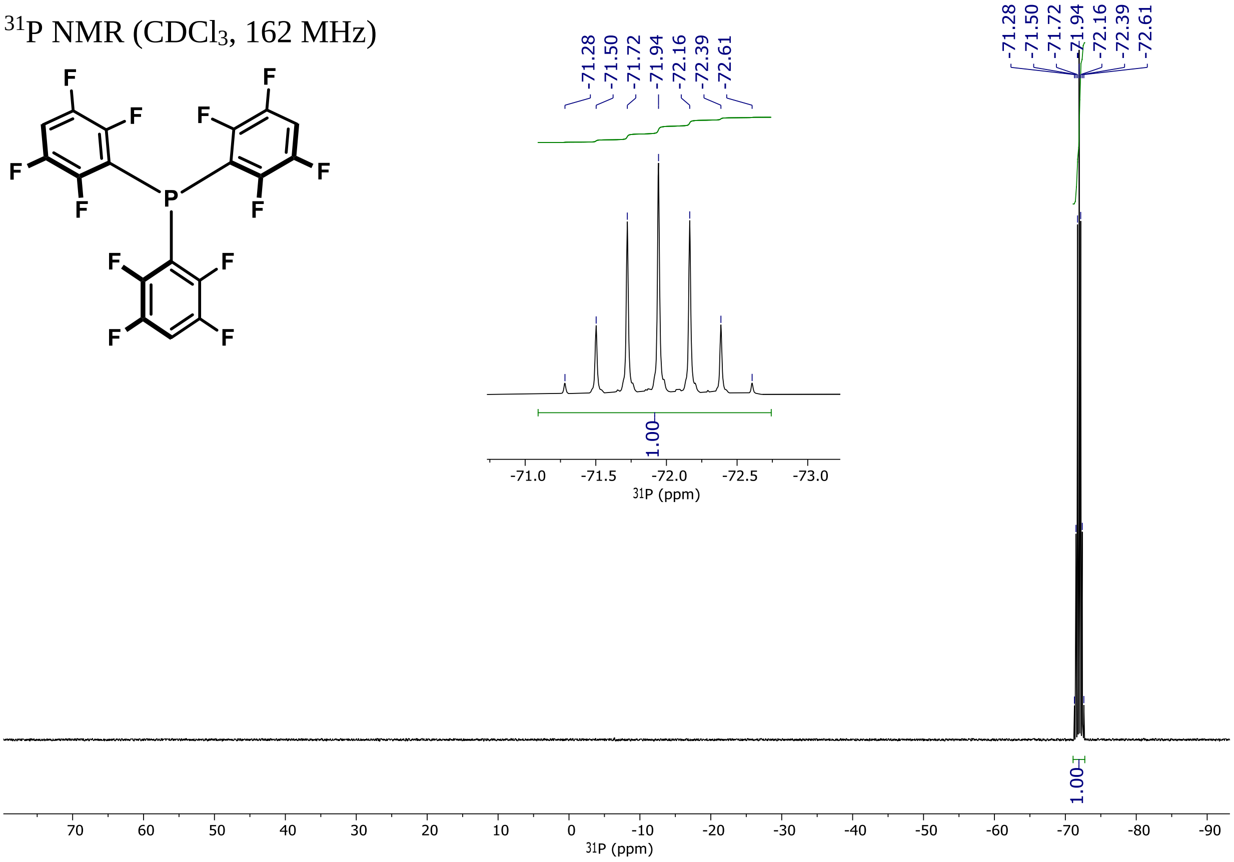

^{19}F NMR ($CDCl_3$, 282 MHz)

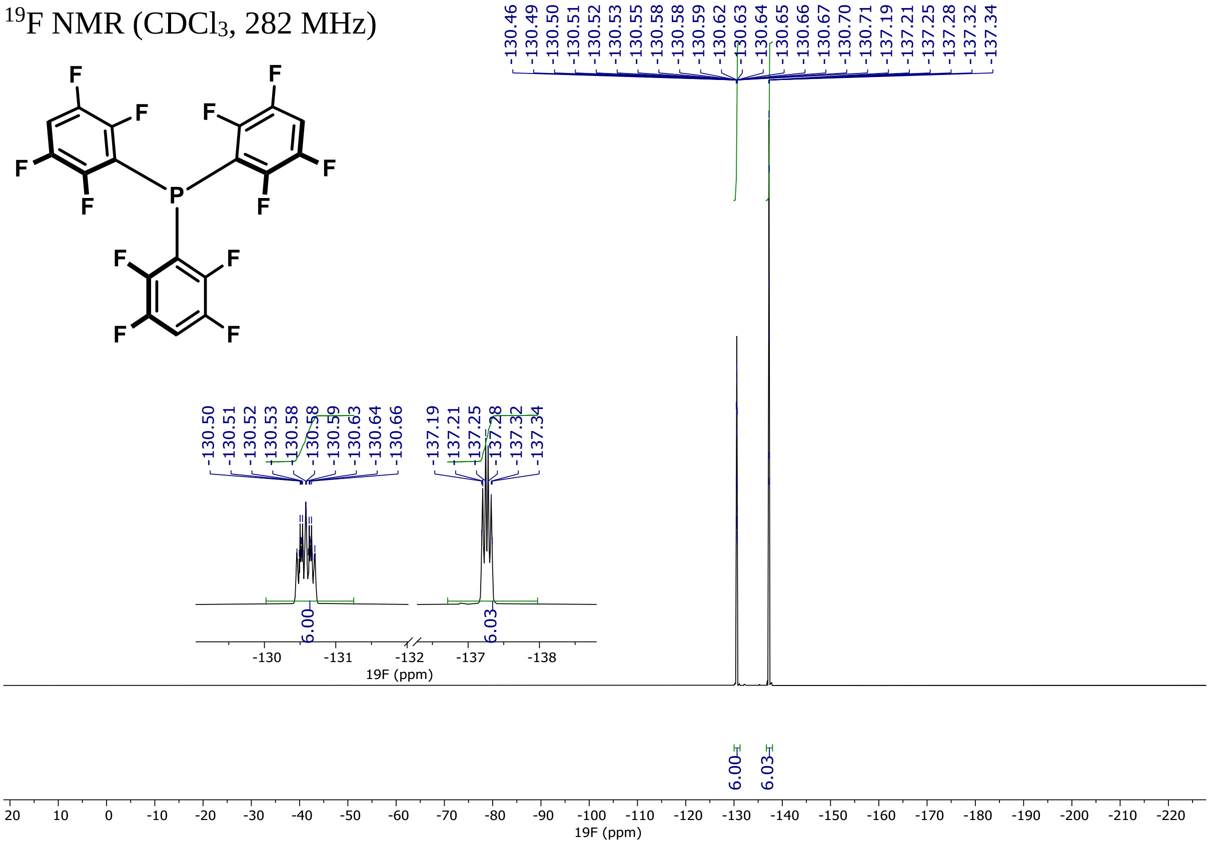

1,2-Bis-[bis-(2,3,5,6-trifluorophenyl)phosphino]ethane (**2k**)

^{1}H NMR ($CDCl_3$, 600 MHz)

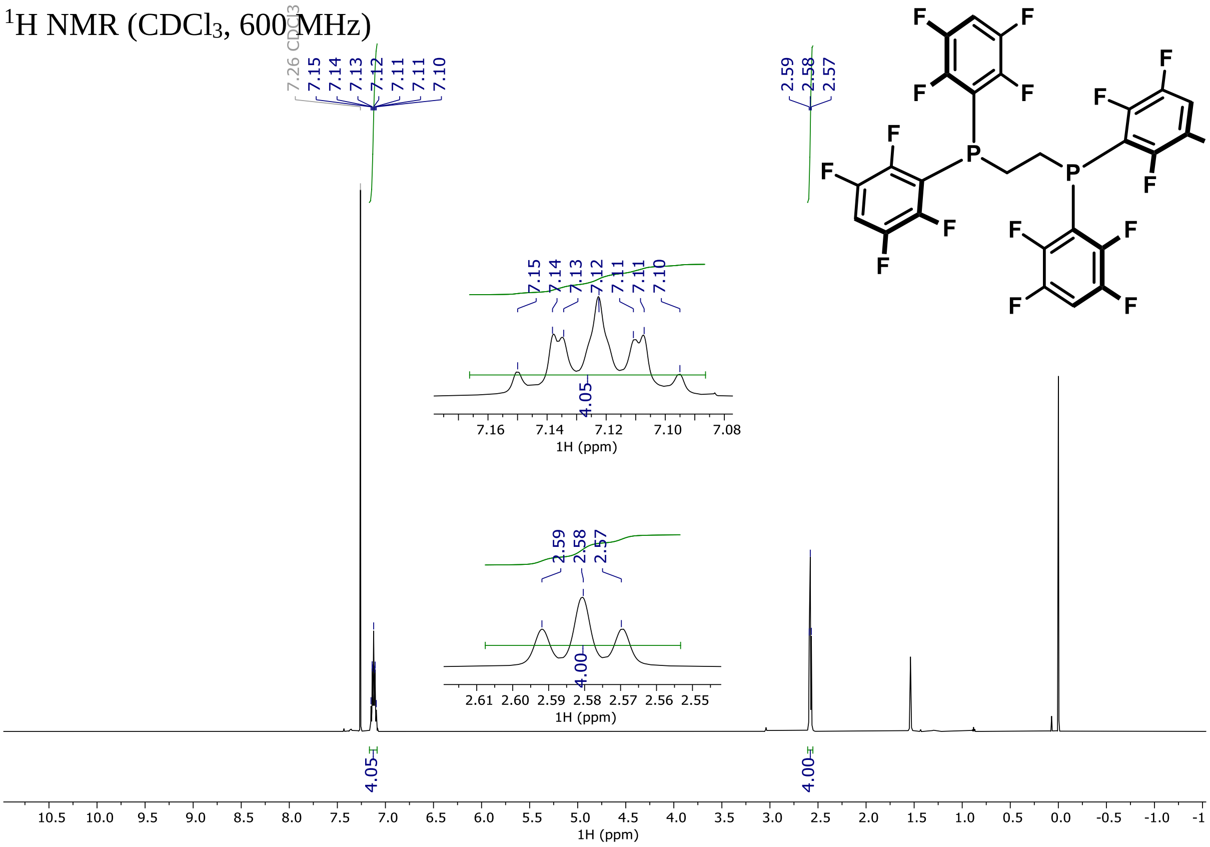

^{13}C NMR ($CDCl_3$, 151 MHz)

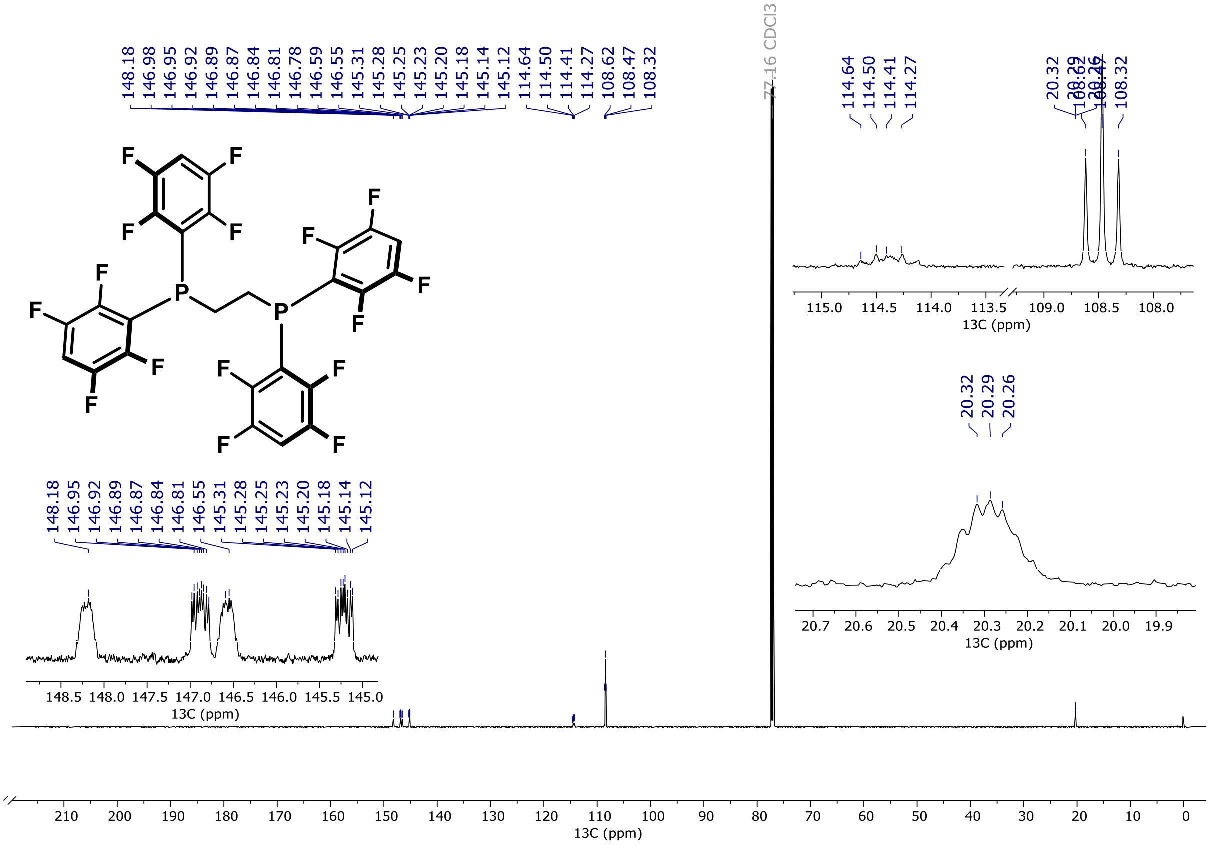

$^{13}C\{^{19}F\}$ NMR ($CDCl_3$, 151 MHz)

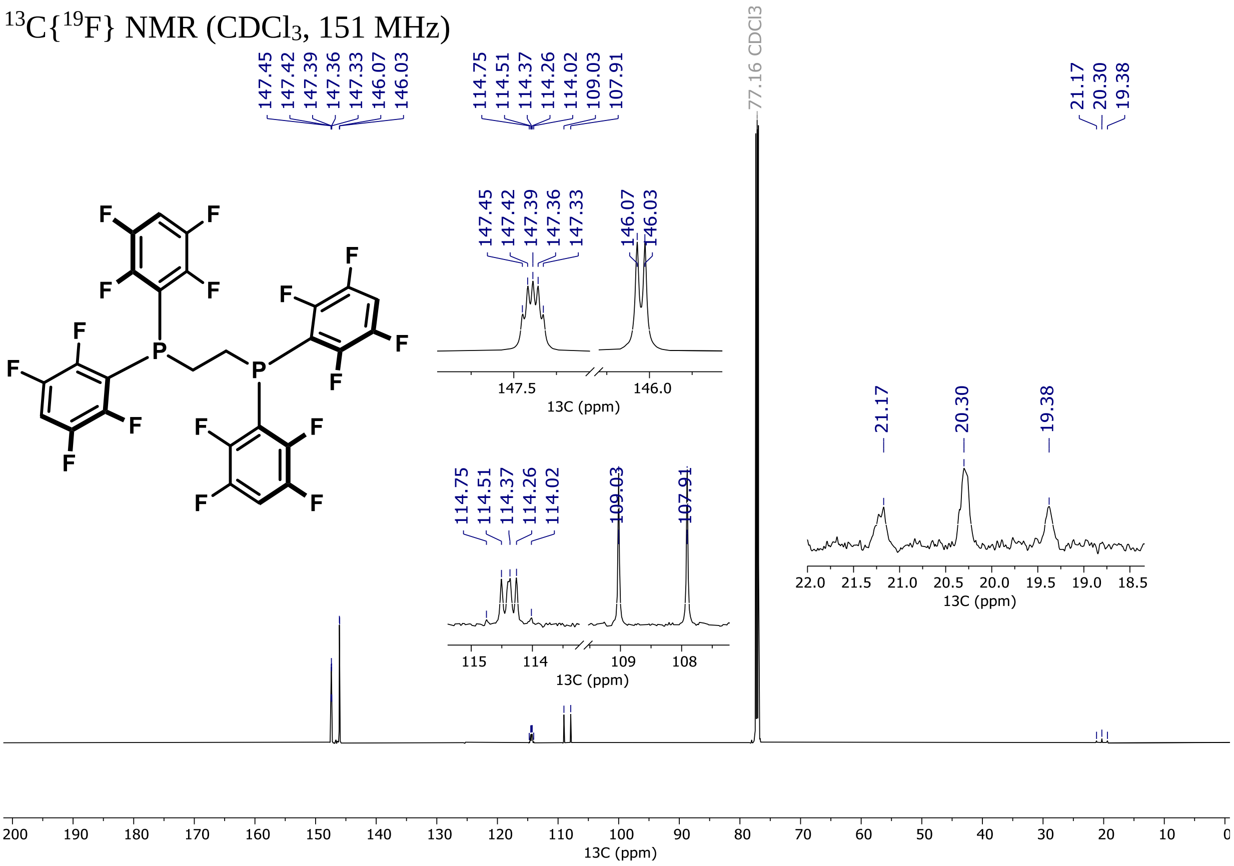

^{31}P NMR ($CDCl_3$, 243 MHz)

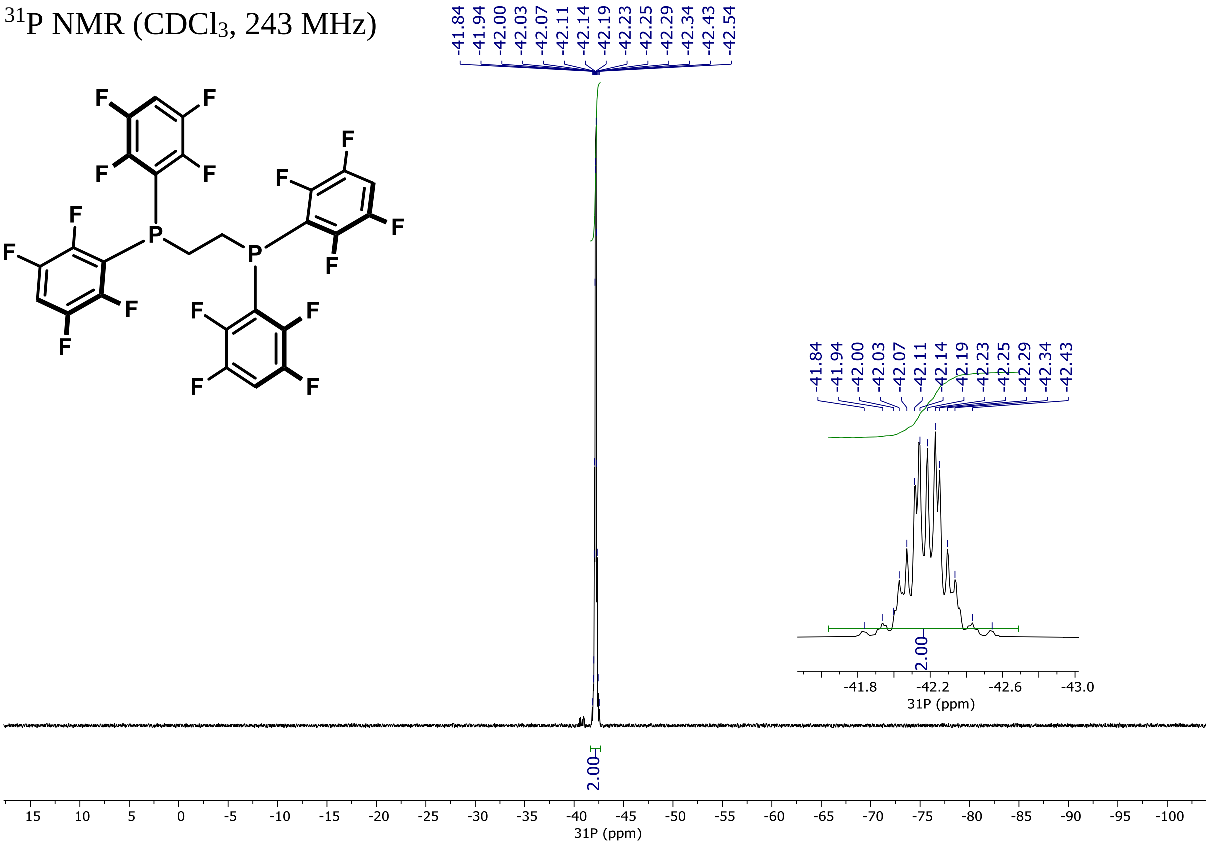

^{19}F NMR ($CDCl_3$, 565 MHz)

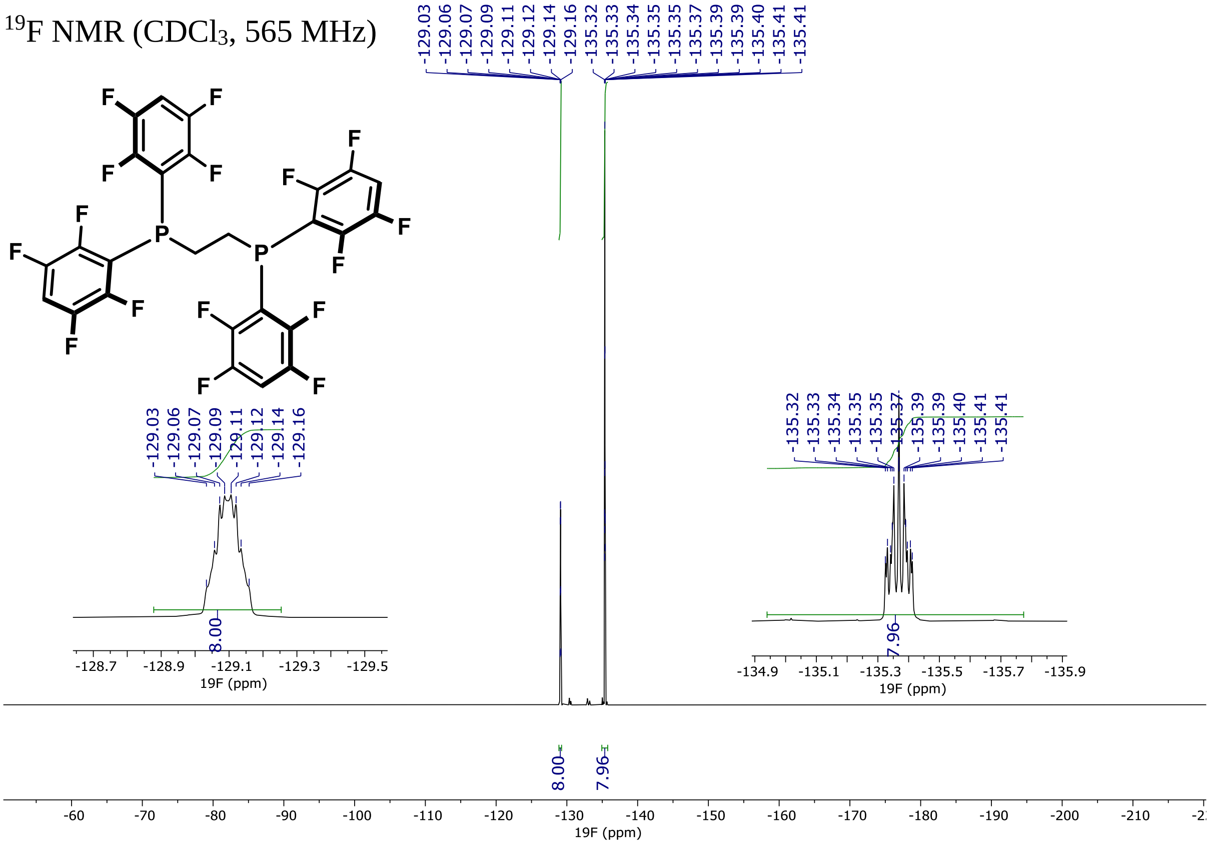

2,3,5,6-Tetrafluoro-1,1'-biphenyl (**2l**)

^{1}H NMR ($CDCl_3$, 400 MHz)

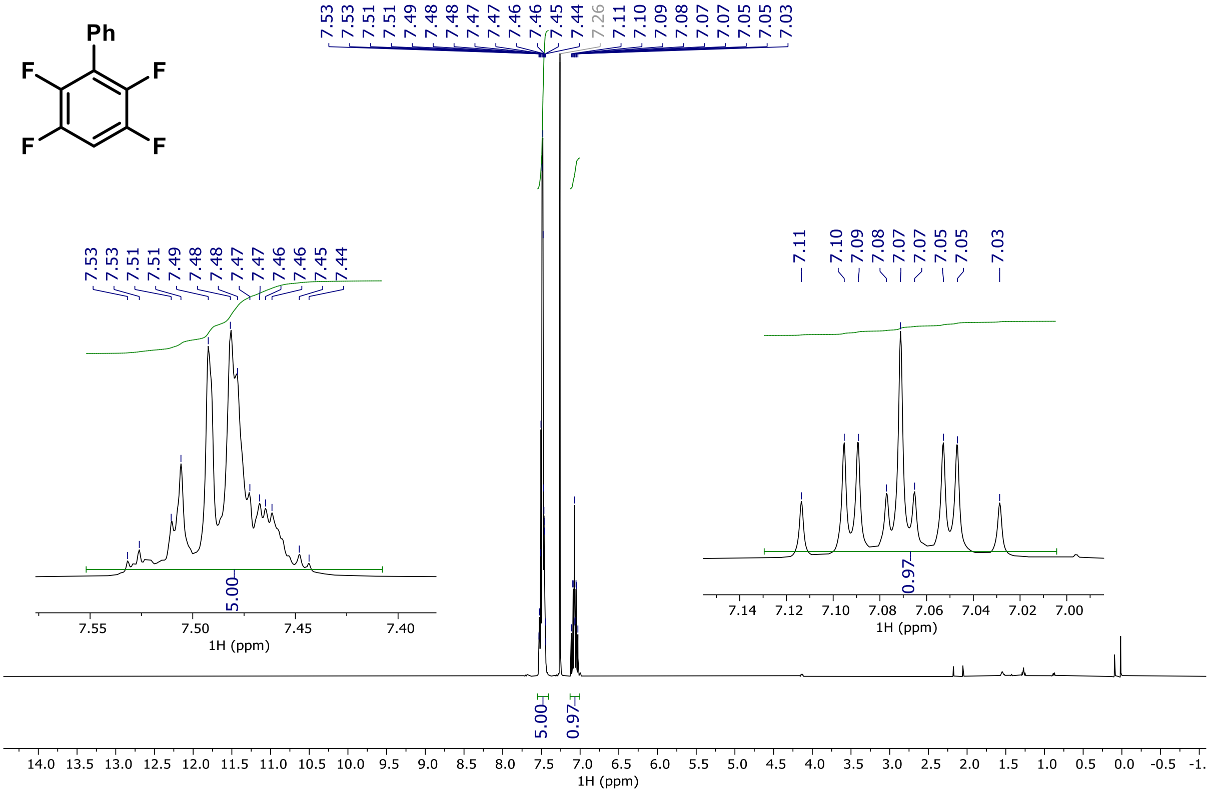

^{13}C NMR ($CDCl_3$, 101 MHz)

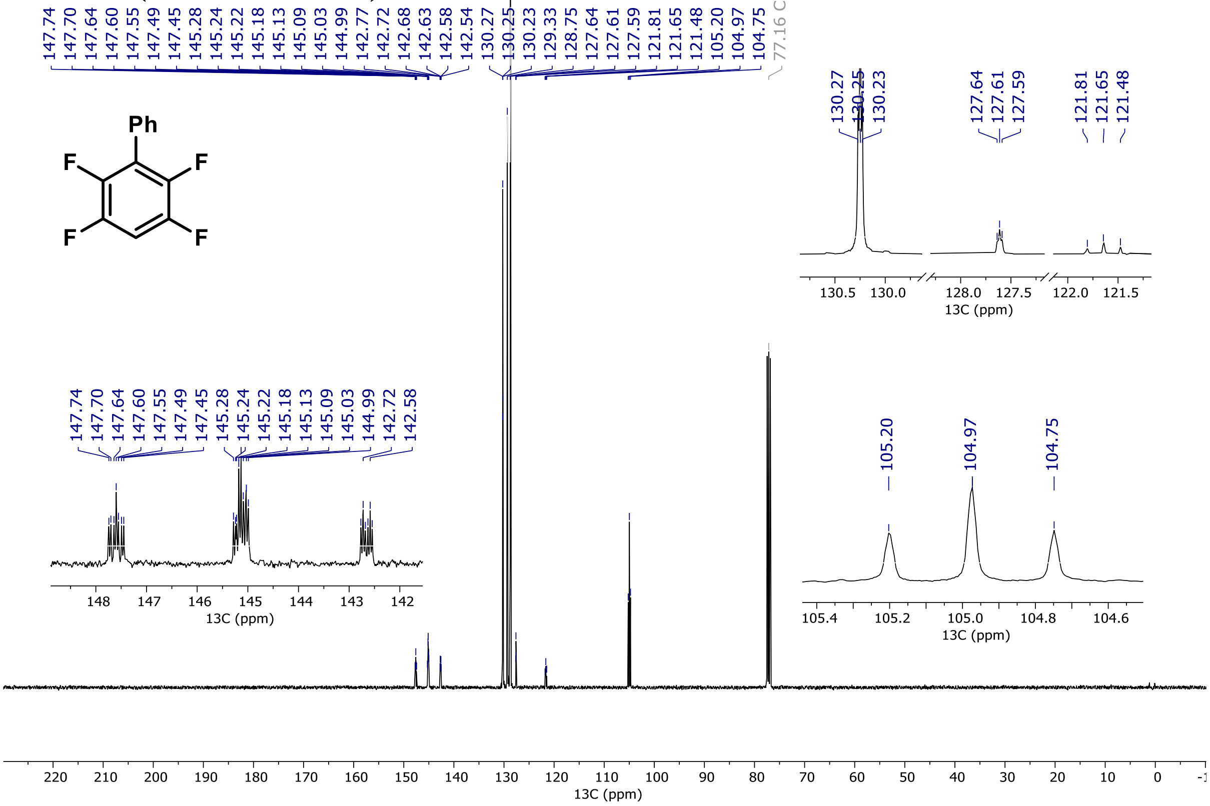

^{19}F NMR ($CDCl_3$, 282 MHz)

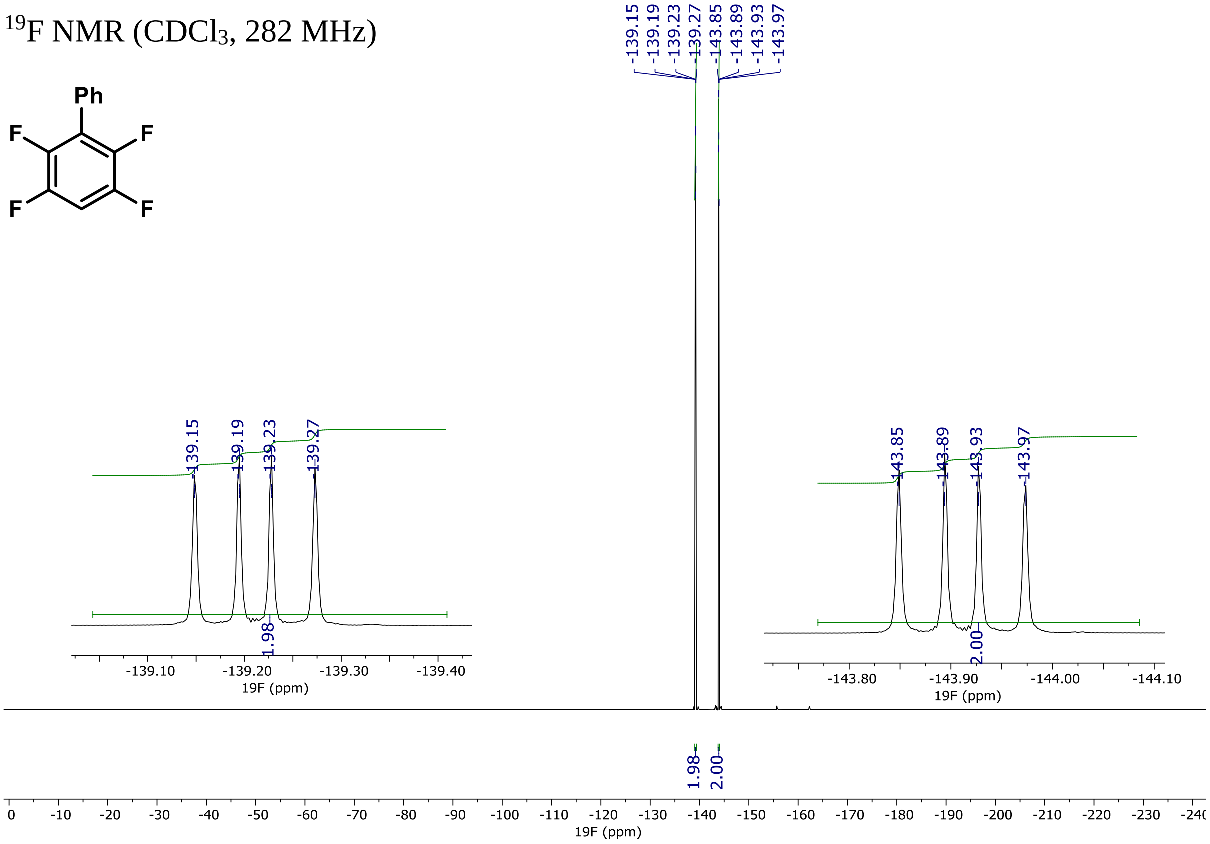

2-(Perfluorophenyl)pyridine (**1m**)

^{1}H NMR ($CDCl_3$, 400 MHz)

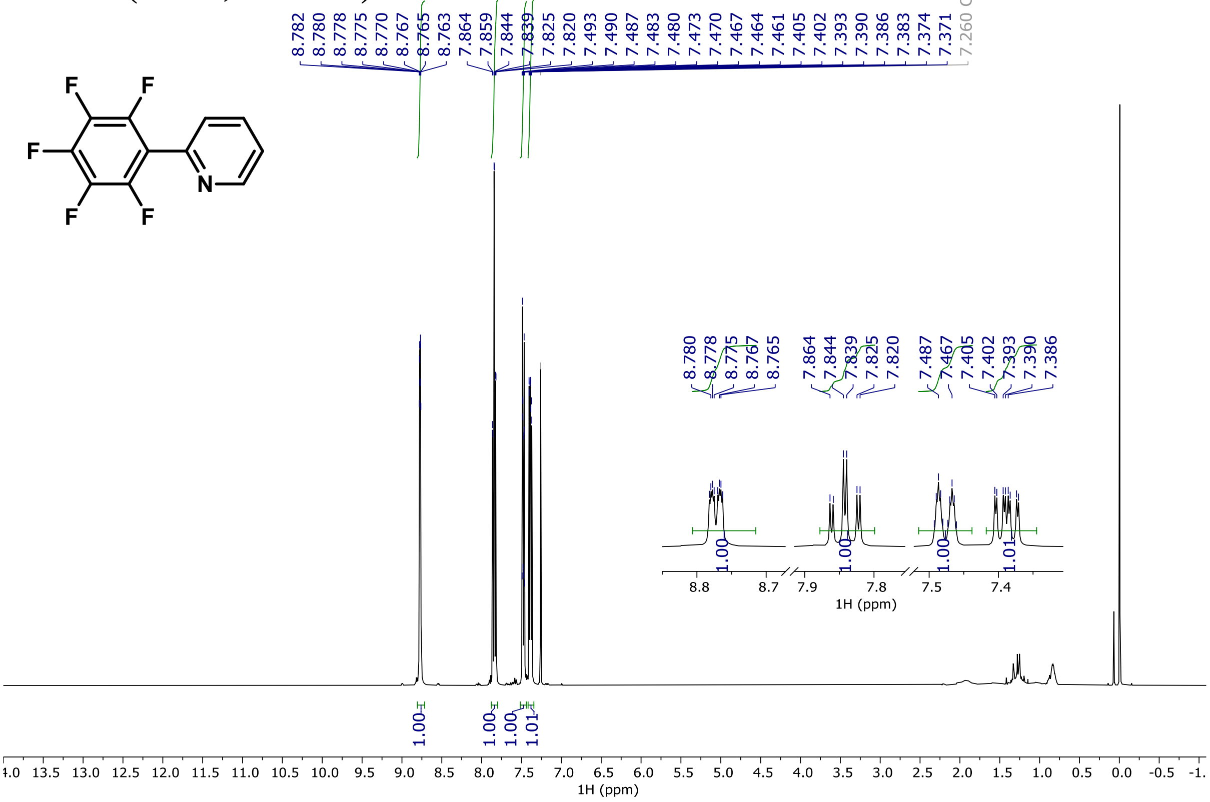

^{13}C NMR ($CDCl_3$, 101 MHz)

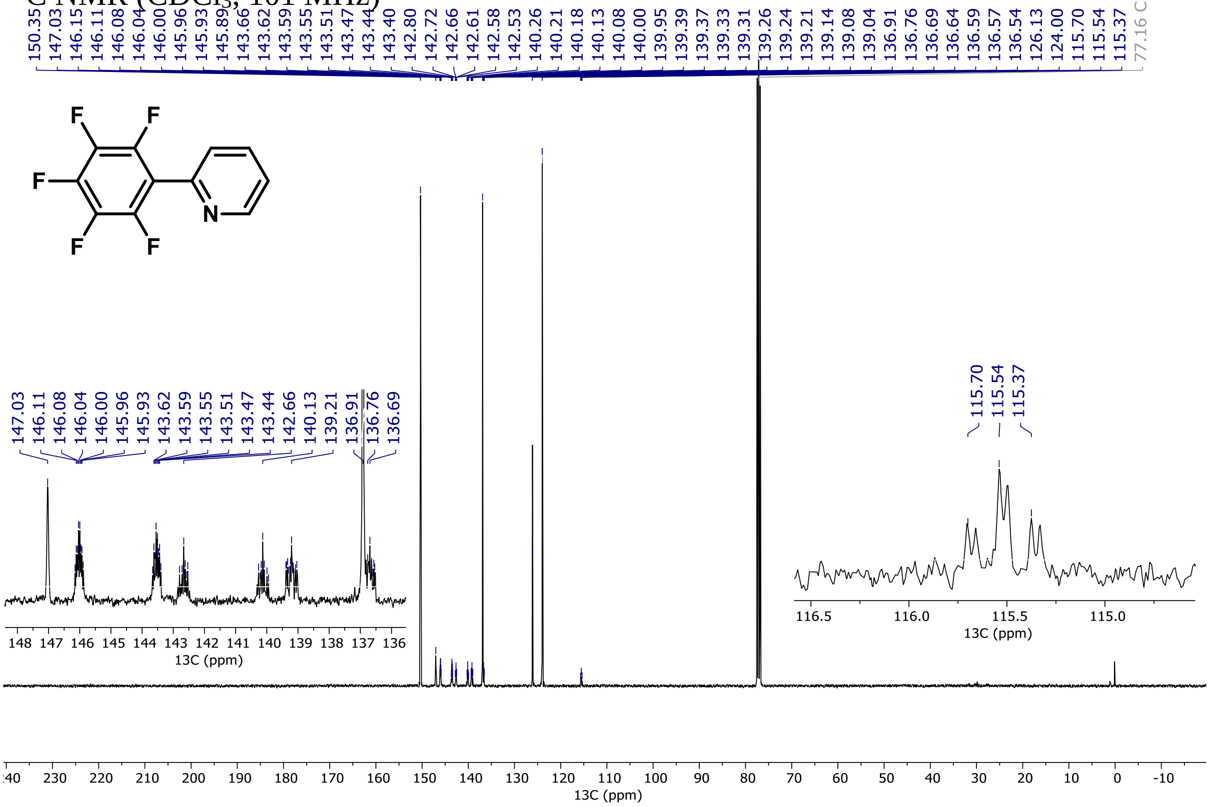

^{19}F NMR (CDCl$_3$, 282 MHz)

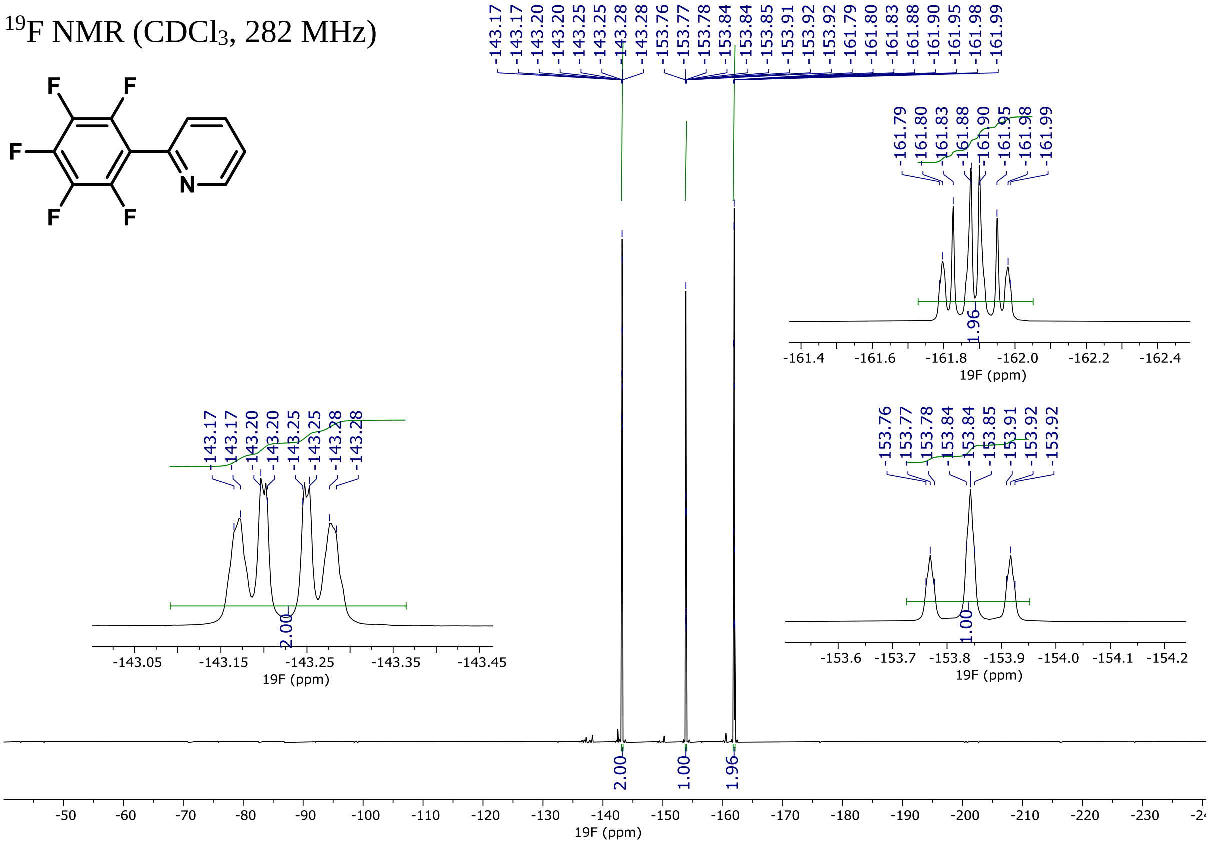

2-(2,3,5,6-Tetrafluorophenyl)pyridine (**2m**)

^{1}H NMR ($CDCl_3$, 400 MHz)

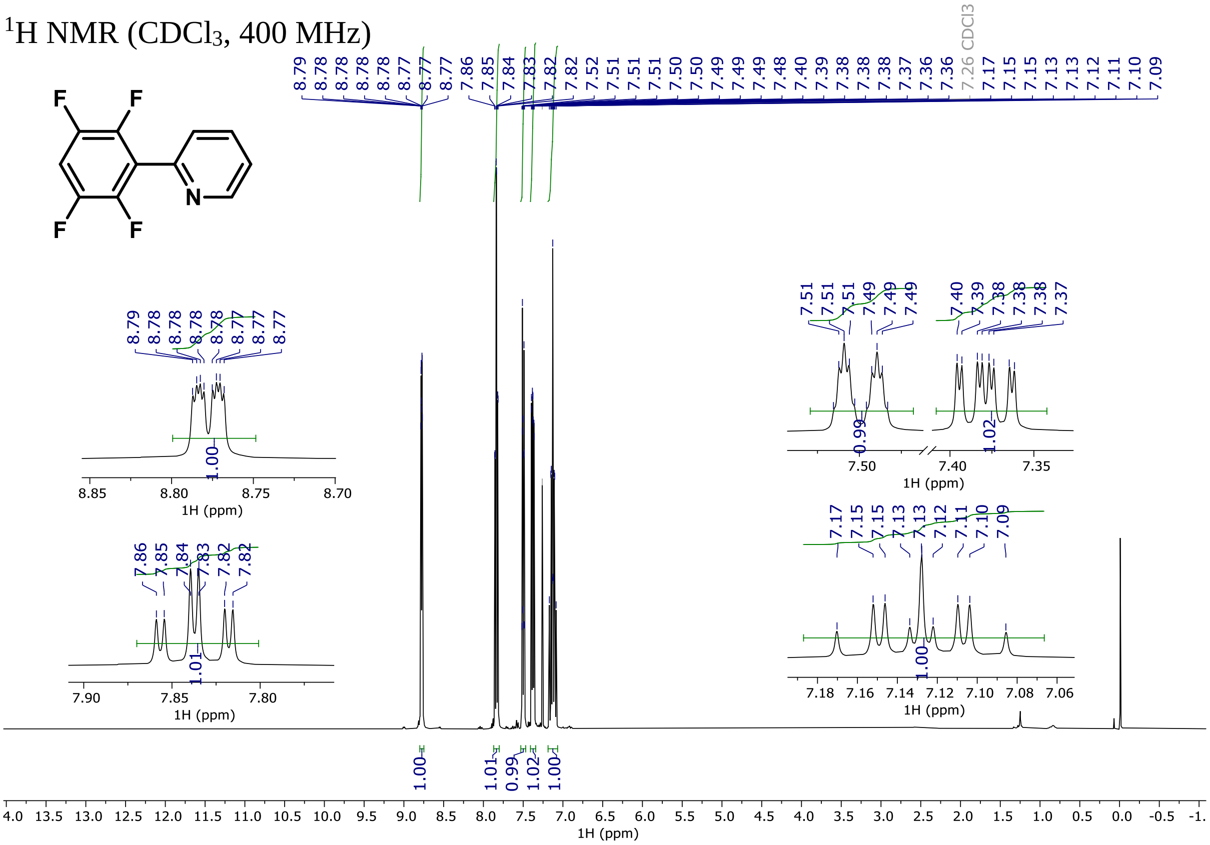

^{13}C NMR ($CDCl_3$, 101 MHz)

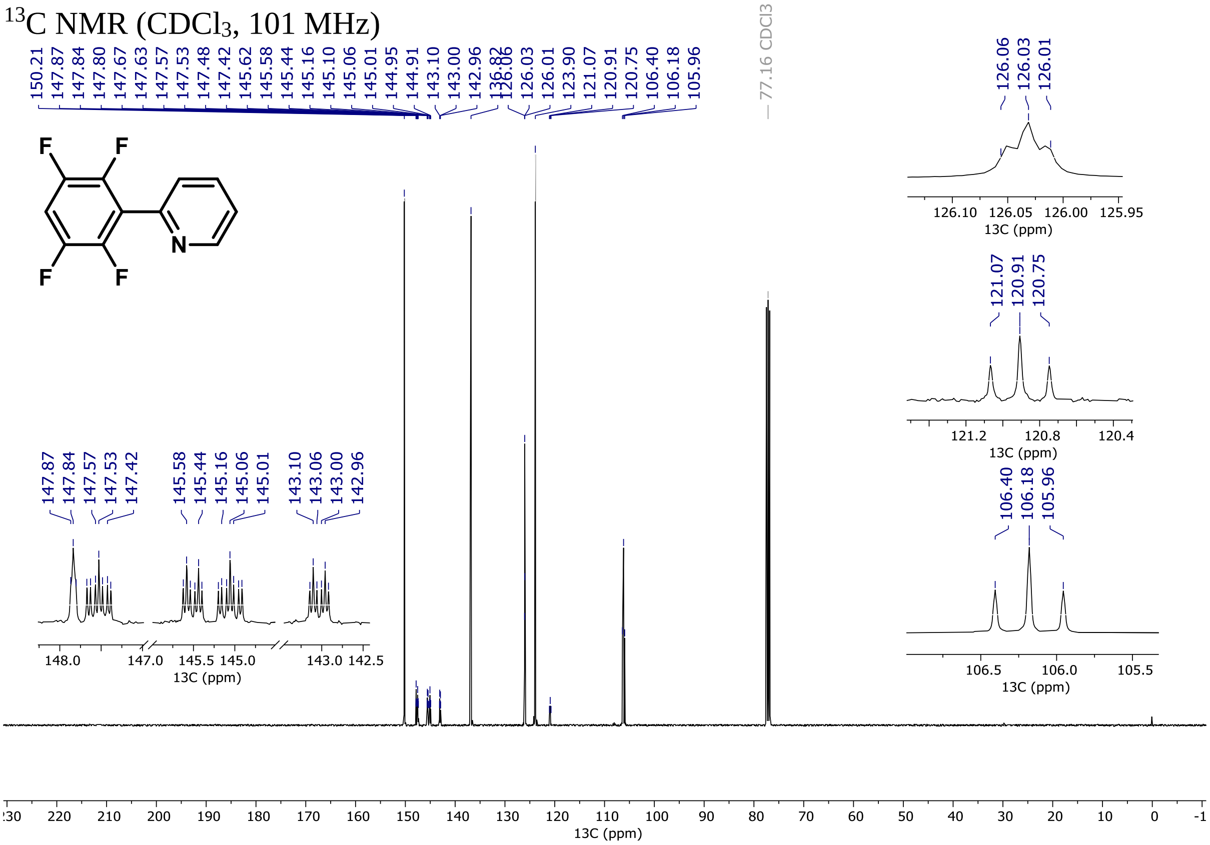

^{19}F NMR ($CDCl_3$, 282 MHz)

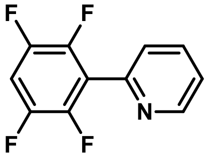

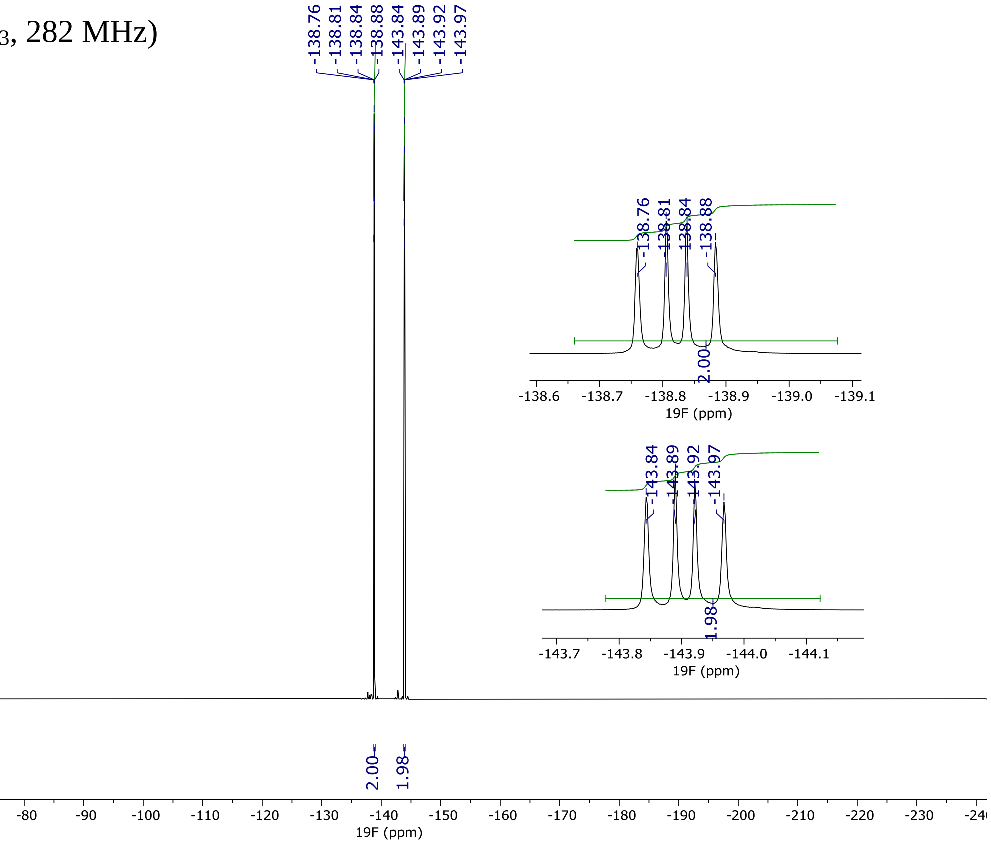

Diethylsilane-d_2

^{1}H NMR ($CDCl_3$, 300 MHz)

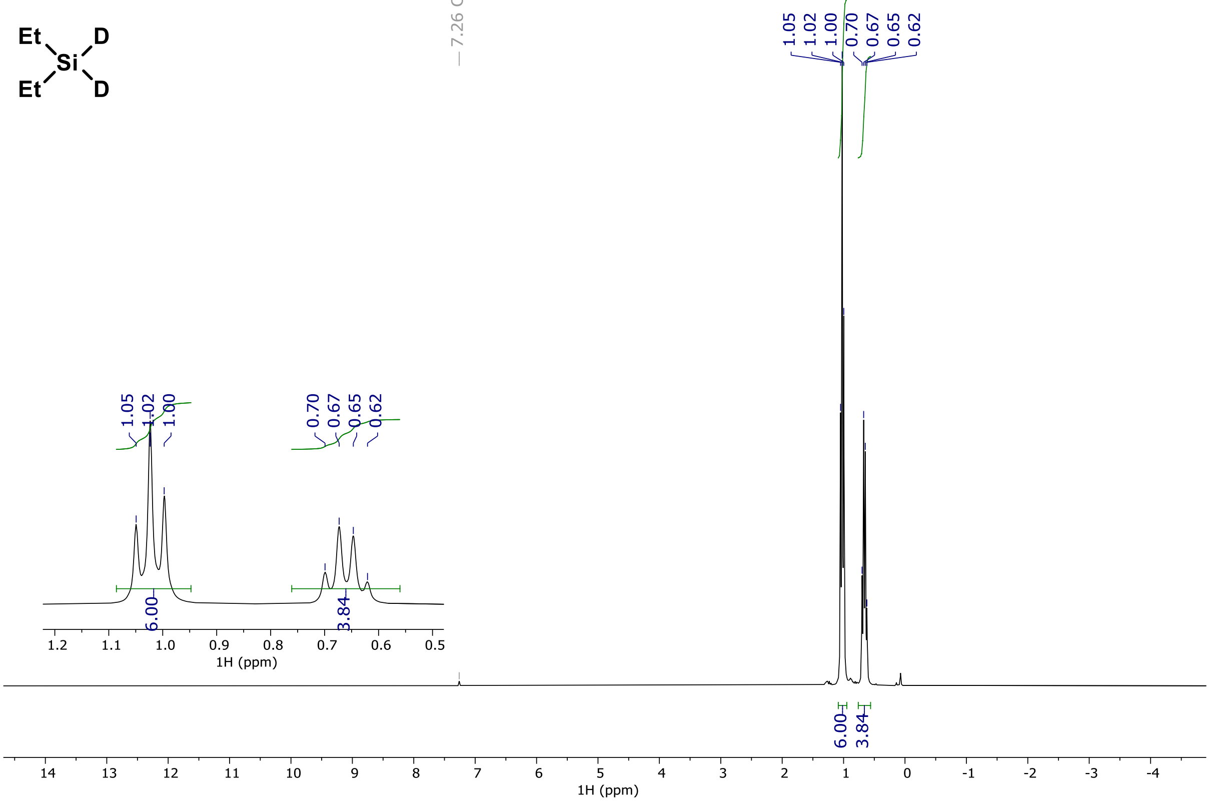

^{13}C NMR ($CDCl_3$, 75 MHz)

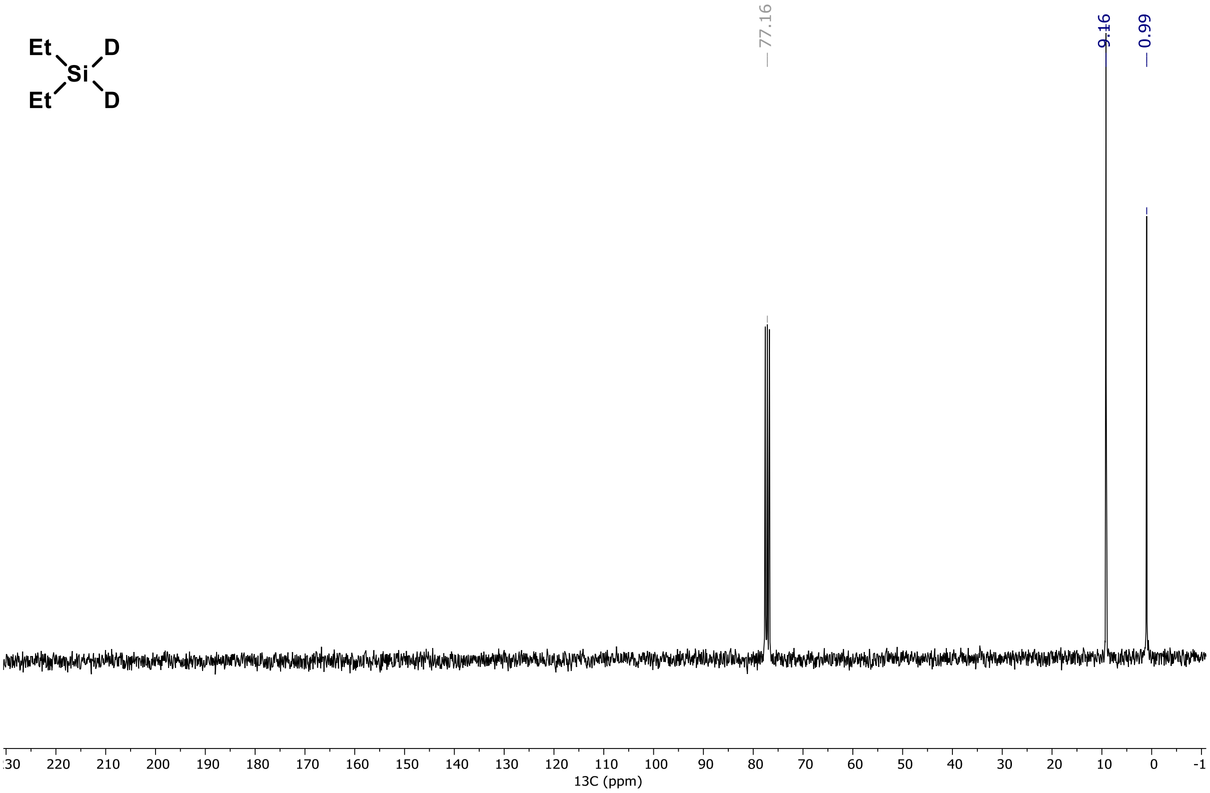

7. References

1. Harris, R. K.; Becker, E. D.; Cabral De Menezes, S. M.; Granger, P.; Hoffman, R. E.; Zilm, K. W., Further Conventions for NMR Shielding and Chemical Shifts (IUPAC Recommendations 2008). *Pure Appl. Chem.* **2008,** *80*, 59-84.
2. (a) Vránová, I.; Alonso, M.; Lo, R.; Sedlák, R.; Jambor, R.; Růžička, A.; Proft, F. D.; Hobza, P.; Dostál, L., From Dibismuthenes to Three- and Two-Coordinated Bismuthinidenes by Fine Ligand Tuning: Evidence for Aromatic BiC_3N Rings through a Combined Experimental and Theoretical Study. *Chem. Eur. J.* **2015,** *21*, 16917-16928; (b) Wang, F.; Planas, O.; Cornella, J., Bi(I)-Catalyzed Transfer-Hydrogenation with Ammonia-Borane. *J. Am. Chem. Soc.* **2019,** *141*, 4235-4240.
3. Pang, Y.; Leutzsch, M.; Nothling, N.; Cornella, J., Catalytic Activation of N_2O at a Low-Valent Bismuth Redox Platform. *J. Am. Chem. Soc.* **2020,** *142*, 19473-19479.
4. (a) Stol, M.; Snelders, D. J. M.; Godbole, M. D.; Havenith, R. W. A.; Haddleton, D.; Clarkson, G.; Lutz, M.; Spek, A. L.; van Klink, G. P. M.; van Koten, G., 2,6-Bis(oxazolinyl)phenylnickel(II) Bromide and 2,6-Bis(ketimine)phenylnickel(II) Bromide: Synthesis, Structural Features, and Redox Properties. *Organometallics* **2007,** *26*, 3985-3994; (b) Nishimoto, Y.; Nakao, S.; Machinaka, S.; Hidaka, F.; Yasuda, M., Synthesis and Characterization of Pheox– and Phebox–Aluminum Complexes: Application as Tunable Lewis Acid Catalysts in Organic Reactions. *Chem. Eur. J.* **2019,** *25*, 10792-10796.
5. Vránová, I.; Jambor, R.; Růžička, A.; Jirásko, R.; Dostál, L., Reactivity of *N,C,N*-Chelated Antimony(III) and Bismuth(III) Chlorides with Lithium Reagents: Addition vs Substitution. *Organometallics* **2015,** *34*, 534-541.
6. (a) Bugarin, A.; Connell, B. T., Chiral Nickel(II) and Palladium(II) NCN-Pincer Complexes Based on Substituted Benzene: Synthesis, Structure, and Lewis Acidity. *Organometallics* **2008,** *27*, 4357-4369; (b) Bugarin, A.; Connell, B. T., A highly active and selective palladium pincer catalyst for the formation of α-aryl ketonesvia cross-coupling. *Chem. Commun.* **2011,** *47*, 7218-7220.
7. (a) Lv, H.; Cai, Y.-B.; Zhang, J.-L., Copper-Catalyzed Hydrodefluorination of Fluoroarenes by Copper Hydride Intermediates. *Angew. Chem., Int. Ed.* **2013,** *52*, 3203-3207; (b) Senaweera, S. M.; Singh, A.; Weaver, J. D., Photocatalytic Hydrodefluorination: Facile Access to Partially Fluorinated Aromatics. *J. Am. Chem. Soc.* **2014,** *136*, 3002-3005; (c) Kikushima, K.; Grellier, M.; Ohashi, M.; Ogoshi, S., Transition-Metal-Free Catalytic Hydrodefluorination of Polyfluoroarenes by Concerted Nucleophilic Aromatic Substitution with a Hydrosilicate. *Angew. Chem., Int. Ed.* **2017,** *56*, 16191-16196.
8. Lv, H.; Zhan, J.-H.; Cai, Y.-B.; Yu, Y.; Wang, B.; Zhang, J.-L., π–π Interaction Assisted Hydrodefluorination of Perfluoroarenes by Gold Hydride: A Case of Synergistic Effect on C–F Bond Activation. *J. Am. Chem. Soc.* **2012,** *134*, 16216-16227.
9. Schoch, T. D.; Mondal, M.; Weaver, J. D., Catalyst-Free Hydrodefluorination of Perfluoroarenes with $NaBH_4$. *Org. Lett.* **2021**.
10. Zhivetyeva, S. I.; Goryunov, L. I.; Bagryanskaya, I. Y.; Grobe, J.; Shteingarts, V. D.; Würthwein, E. U., Phosphinodefluorination of polyfluorobenzenes by silylphosphines $Ph(R)PSiMe_3$ (R=Me, Ph): Further experimental and computational evidences for the concerted ANDN mechanism of aromatic nucleophilic substitution. *J. Fluor. Chem.* **2014,** *164*, 58-69.
11. Korenaga, T.; Abe, K.; Ko, A.; Maenishi, R.; Sakai, T., Ligand Electronic Effect on Reductive Elimination of Biphenyl from *cis*-$[Pt(Ph)_2(diphosphine)]$ Complexes Bearing Electron-Poor Diphosphine: Correlation Study between Experimental and Theoretical Results. *Organometallics* **2010,** *29*, 4025-4035.
12. (a) Wei, Y.; Kan, J.; Wang, M.; Su, W.; Hong, M., Palladium-Catalyzed Direct Arylation of Electron-Deficient Polyfluoroarenes with Arylboronic Acids. *Org. Lett.* **2009,** *11*,

3346-3349; (b) Do, H.-Q.; Daugulis, O., A General Method for Copper-Catalyzed Arene Cross-Dimerization. *J. Am. Chem. Soc.* **2011,** *133*, 13577-13586.

13. (a) Do, H.-Q.; Daugulis, O., Copper-Catalyzed Arylation and Alkenylation of Polyfluoroarene C−H Bonds. *J. Am. Chem. Soc.* **2008,** *130*, 1128-1129; (b) Yu, D.; Lu, L.; Shen, Q., Palladium-Catalyzed Coupling of Polyfluorinated Arenes with Heteroarenes via C–F/C–H Activation. *Org. Lett.* **2013,** *15*, 940-943.

14. Chen, J.; Cammers-Goodwin, A., 2-(Fluorophenyl)pyridines by the Suzuki–Miyaura method: Ag_2O accelerates coupling over undesired *ipso* substitution (SNAr) of fluorine. *Tetrahedron Lett.* **2003,** *44*, 1503-1506.

15. Tyrra, W.; Aboulkacem, S.; Hoge, B.; Wiebe, W.; Pantenburg, I., Silver compounds in synthetic chemistry: Part 4. 4-Tetrafluoropyridyl silver(I), AgC_5F_4N in redox transmetallations—possibilities and limitations in reactions with group 15 elements. *J. Fluor. Chem.* **2006,** *127*, 213-217.

16. (a) Tyrra, W.; Naumann, D., On pentavalent perfluoroorgano bismuth compounds. *Can. J. Chem.* **1989,** *67*, 1949-1951; (b) Olaru, M.; Nema, M. G.; Soran, A.; Breunig, H. J.; Silvestru, C., Mixed triorganobismuthines RAr_2Bi [Ar = C_6F_5, 2,4,6-$(C_6F_5)_3C_6H_2$] and hypervalent racemic Bi-chiral diorganobismuth(III) bromides RArBiBr (Ar = C_6F_5, Mes, Ph) with the ligand R = 2-$(Me_2NCH_2)C_6H_4$. Influences of the organic substituent. *Dalton Trans.* **2016,** *45*, 9419-9428; (c) Solyntjes, S.; Bader, J.; Neumann, B.; Stammler, H.-G.; Ignat'ev, N.; Hoge, B., Pentafluoroethyl Bismuth Compounds. *Chem. Eur. J.* **2017,** *23*, 1557-1567.

17. Aldridge, S.; Downs, A. J., Hydrides of the Main-Group Metals: New Variations on an Old Theme. *Chem. Rev.* **2001,** *101*, 3305-3366.

18. Amberger, E., Hydride des Wismuts. *Chem. Ber.* **1961,** *94*, 1447-1452.

19. Šimon, P.; de Proft, F.; Jambor, R.; Růžička, A.; Dostál, L., Monomeric Organoantimony(I) and Organobismuth(I) Compounds Stabilized by an NCN Chelating Ligand: Syntheses and Structures. *Angew. Chem., Int. Ed.* **2010,** *49*, 5468-5471.

20. (a) Balázs, G.; Breunig, H. J.; Lork, E., Synthesis and Characterization of R_2SbH, R_2BiH, and $R_2Bi{-}BiR_2$ [R = $(Me_3Si)_2CH$]. *Organometallics* **2002,** *21*, 2584-2586; (b) Schwamm, R. J.; Lein, M.; Coles, M. P.; Fitchett, C. M., Catalytic oxidative coupling promoted by bismuth TEMPOxide complexes. *Chem. Commun.* **2018,** *54*, 916-919; (c) Ramler, J.; Krummenacher, I.; Lichtenberg, C., Well-Defined, Molecular Bismuth Compounds: Catalysts in Photochemically Induced Radical Dehydrocoupling Reactions. *Chem. Eur. J.* **2020,** *26*, 14551-14555.

21. (a) Hardman, N. J.; Twamley, B.; Power, P. P., $(2,6\text{-}Mes_2H_3C_6)_2BiH$, a Stable, Molecular Hydride of a Main Group Element of the Sixth Period, and Its Conversion to the Dibismuthene $(2,6\text{-}Mes_2H_3C_6)BiBi(2,6\text{-}Mes_2C_6H_3)$. *Angew. Chem., Int. Ed.* **2000,** *39*, 2771-2773; (b) Olaru, M.; Duvinage, D.; Lork, E.; Mebs, S.; Beckmann, J., Heavy Carbene Analogues: Donor-Free Bismuthenium and Stibenium Ions. *Angew. Chem., Int. Ed.* **2018,** *57*, 10080-10084.

22. Ashe, A. J.; Ludwig, E. G.; Oleksyszyn, J., Preparation and properties of dibismuthines. *Organometallics* **1983,** *2*, 1859-1866.

23. Vícha, J.; Novotný, J.; Komorovsky, S.; Straka, M.; Kaupp, M.; Marek, R., Relativistic Heavy-Neighbor-Atom Effects on NMR Shifts: Concepts and Trends Across the Periodic Table. *Chem. Rev.* **2020,** *120*, 7065-7103.

24. Ebisawa, K.; Izumi, K.; Ooka, Y.; Kato, H.; Kanazawa, S.; Komatsu, S.; Nishi, E.; Shigehisa, H., Catalyst- and Silane-Controlled Enantioselective Hydrofunctionalization of Alkenes by Cobalt-Catalyzed Hydrogen Atom Transfer and Radical-Polar Crossover. *J. Am. Chem. Soc.* **2020,** *142*, 13481-13490.
